# Photoinduced Autopromoted Ni-Catalyzed Three-Component Arylsulfonation Inspired by Density Functional Theory/Time-Dependent Density Functional Theory-Simulated Photoactive Nickel Species

**DOI:** 10.1021/acs.orglett.4c04222

**Published:** 2024-12-23

**Authors:** Feng Zhang, Xiu-Fen Cheng, Xiaolin Liang, Duo-Duo Hu, Qian Gao, Hongliang Wang, Peng Wu, Yan Li

**Affiliations:** ‡ Chemical Biology Center, School of Pharmaceutical Sciences & Institute of Materia Medica, Shandong First Medical University & Shandong Academy of Medical Sciences, Jinan, Shandong 250117, China; § Department of Chemical Biology, Max Planck Institute of Molecular Physiology, 44227 Dortmund, Germany; ∥ Department of Chemistry, University of Science and Technology of China, 96 Jinzhai Road, Hefei, Anhui 230026, China; ⊥ Chemical Genomics Centre, Max Planck Institute of Molecular Physiology, 44227 Dortmund, Germany

## Abstract

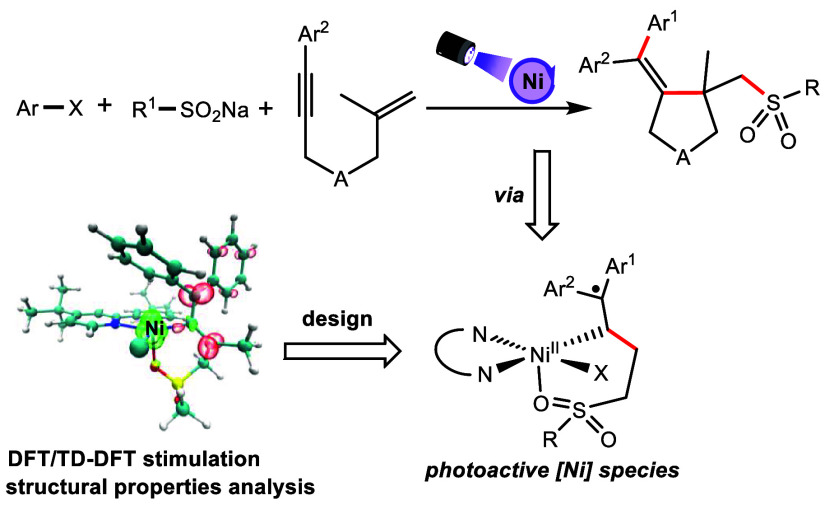

The structure of the novel photoactive nickel species was simulated by density functional theory (DFT)/time-dependent density functional theory (TD-DFT) calculations. The application of the simplified photoactive nickel catalyst was demonstrated in a photoinduced nickel-catalyzed three-component arylsulfonation of 1,6-enynes. This reaction was autopromoted and proceeded in the absence of an additional photocatalyst. This methodology exhibited mild conditions, a broad substrate scope, and high efficiency.

The visible-light catalysis strategy has gradually become one of the most powerful tools in organic synthetic chemistry.^1^ The introduction of visible light makes the reaction green and mild, and the photocatalysis strategy can be well-applied with continuous-flow technology.^2^ The photocatalytic reaction undergoes an excited state, a highly active species, to access diverse building blocks from readily available raw materials in an atom- and step-economic manner. Recently, a combination of photocatalysis with transition metal catalysis makes it possible to synthesize complex molecular motifs with high selectivity and high efficiency.^3^ In particular, photo-nickel dual catalysis, which provides a mild method to construct carbon–carbon bonds and carbon–heteroatom bonds, is often used for multi-component coupling reactions. Free radical species are generated by the visible photocatalytic cycle, which can be captured by unsaturated bonds and then coupled with counterparts in the transition metal catalytic cycle. However, such a method heavily relies on the usage of noble-metal-based photocatalysts (such as Ir and Ru), which greatly limits the large-scale application of this technology due to sustainability and cost issues.

In addition to the traditional photo-nickel dual catalytic system, the photoinduced nickel-catalyzed cross coupling,^4^ which has been pioneered by Doyle et al.,^5^ Miyake et al.,^6^ Alcázar et al.,^7^ since 2018, brought the combination of visible photocatalysis and nickel catalysis into a new stage.^8^ In such a method, the nickel complex is allowed to serve as both a cross-coupling catalyst and a photoactive substance. Different from the conventional synergistic catalytic system of photosensitizer and transition metal, photoinduced transition metal catalysis does not need an additional photosensitizer, thus achieving a more green and sustainable reaction. However, to the best of our knowledge, all of the photoinduced nickel-catalyzed cross couplings are two-component couplings, and most of the photoactive substances in the reactions are the complexes of nickel catalysts and ligands or their adducts with substrates (Scheme 1a).^4^

**Scheme 1:**
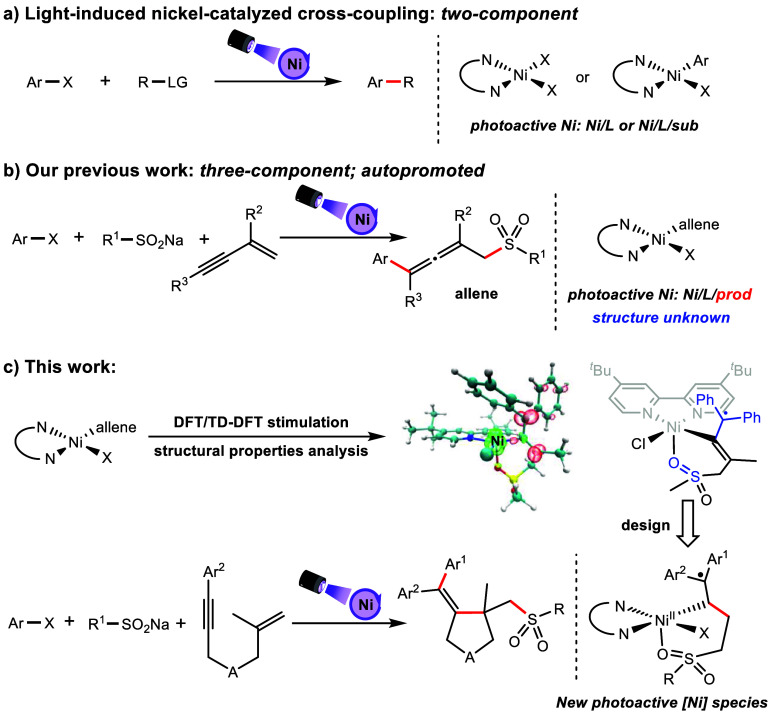
Light-Induced Nickel-Catalyzed Coupling Reactions and Their Photoactive Nickel Catalysts

In 2022, we reported the first photoinduced nickel-catalyzed three-component coupling reaction to synthesize sulfonyl allenes.^9^ The detailed mechanism investigation demonstrated that the photoactive substance is the complex of nickel catalyst, ligand, and sulfonyl allene product, leading to the characteristics of autocatalysis (Scheme 1b).^10^ Given that it is impossible for the two cumulative double bonds of allene to coordinate with a nickel center simultaneously, the ligating atoms and their combination modes may influence the reactivity and selectivity of the reactions. Therefore, we propose that it will be helpful to develop new catalysts through theoretical calculation and simulation of the catalysts in known reactions. Herein, we performed density functional theory (DFT) and time-dependent density functional theory (TD-DFT) calculations to investigate the potential structure of the photoactive nickel complex. The calculated results suggested a novel coordination mode of sulfonyl allene and nickel center. On the basis of the hypothetic photoinitiator structure, we described a three-component tandem cyclization of 1,6-enyne with sulfinate by photoinduced nickel catalysis (Scheme 1c). This method exhibited mild conditions, a broad substrate scope, and high efficiency. The mechanism study showed similar autopromoted characteristics to our previous reports, which verified the structure of the photoactive nickel catalyst.

Our investigation was initiated with the exploration of photoactive substances in the previous reports. It had been proven that the coordination compound of the sulfonyl allene product and the dtbpy-ligated Ni(I) complex was the key photoactive catalyst by the transient absorption kinetic research. To verify which combination mode plays a substantial role in the light absorption as a virtual photoinitiator, we performed structure optimization with DFT and ultraviolet–visible (UV–vis) spectrum simulation with TD-DFT calculations (Figure S1 of the Supporting Information). The calculated results showed that complex A may serve as the virtual photoinitiator in the system, exhibiting a maximum absorption wavelength of 400 nm, which corresponds to the purple light spectrum (395–415 nm).

The previous mechanistic studies^9^ proved that the addition of sulfonyl allene could accelerate the reaction, but sulfone or allene alone could not. Therefore, we were intrigued to identify which moiety of the sulfonyl allene structure played a pivotal role in promoting the photoinduced reaction. Consequently, a comprehensive analysis involving consideration of the structural properties (Figure 1a), interaction region indicator (IRI) analysis^11^ (Figure 1b), and spin population analysis (Figure 1c) was performed for complex **A**. The Mayer bond orders of Ni–O1 and Ni–C1, as depicted in Figure 1a, were calculated to be 0.31 and 0.68, respectively, with corresponding bond lengths of 2.15 and 2.05 Å. The higher bond order and shorter bond length indicated a stronger Ni–C1 bond compared to that of the Ni–O1 coordination bond, suggesting that the former is a bonding interaction. The IRI analysis (Figure 1b) revealed a pronounced attractive interaction between Ni–O1 and Ni–C1 in complex **A**. The analyses suggested that the valence state of Ni in complex **A** may potentially be bivalent. Spin population analysis was performed to further understand the electronic structure characteristics of complex **A** (Figure 1c). The calculated spin density on the Ni atom exceeded one electron (1.491) and was compensated for by the negative spin density observed on the sulfonyl allene ligand. Notably, most of the negative spin density was located on the diphenylmethylene moiety [C2 (−0.231) versus C3 (−0.320), C4 (−0.091), C5 (−0.103), and C6 (−0.092)]. As reflected by the Mayer bond orders of C1–C2 (1.61) and C1–C3 (1.38) and corresponding bond lengths of 1.36 and 1.40 Å, the bonding interaction of C1–C3 is weaker than that of the C1=C2 double bond, which further supported the carbon radical (C3) on the diphenylmethylene moiety. On the basis of the above analysis on the photoactive Ni(II) complex **A**, we speculated that the sulfonyl and diphenyl vinyl groups, instead of the sulfone and allene structures, may be the key components to coordinate with Ni(I). On the basis of the results of theoretical calculation, a three-component coupling reaction was designed with 1,6-enyne as the substrate, and the corresponding product would include diphenyl vinyl and sulfonyl groups.

**Figure 1:**
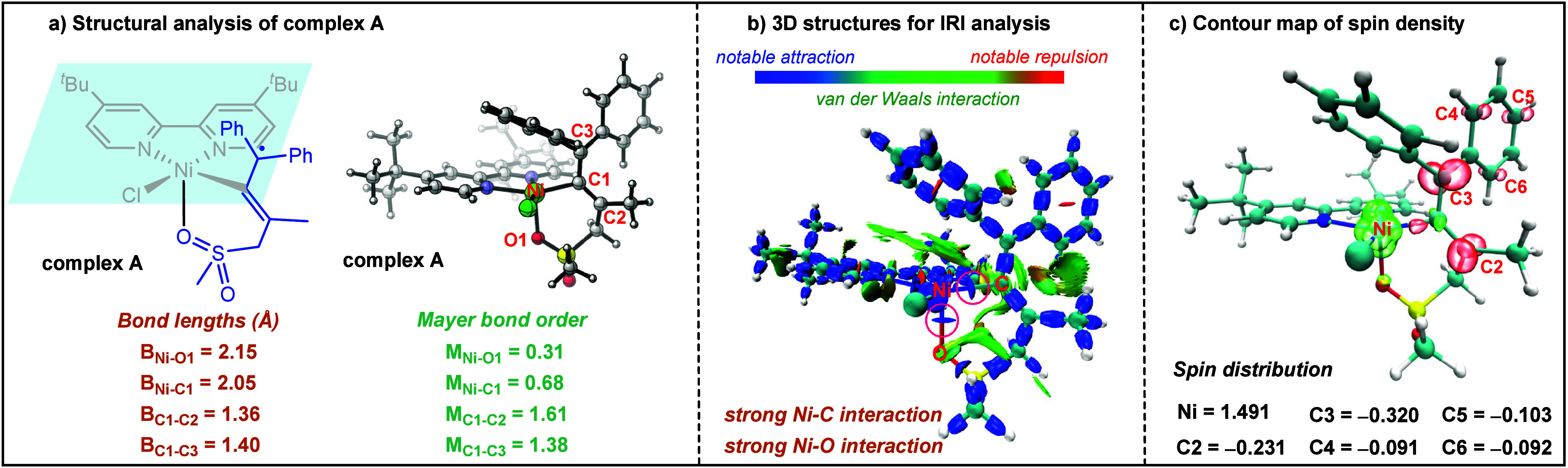
(a) Optimized geometry information, including bond lengths (Å) and Mayer bond orders for complex **A**, (b) three-dimensional structures for IRI analysis of complex **A**, with the isosurface of 1.0, and (c) contour map of spin density for complex **A**, with the isosurface of 0.1.

Considering the influence of the Thorpe–Ingold effect on the cyclization process, compound **1a** was chosen as the pilot substrate, sodium methylsulfinate **2a** was chosen as the sulfonyl radical source, and 4-iodobenzonitrile **3a** was chosen as the coupling partner. The investigation was commenced under the identical reaction conditions outlined in our previous report. To our delight, the three-component cyclized product could be obtained in 46% yield (entry 1 in Table 1). Solvent screening revealed that this photoinduced transformation was carried out successfully in *N,N-*dimethylacetamide (DMA) and dimethyl sulfoxide (DMSO) (entries 2 and 3 in Table 1). The use of DMSO gave the best yield of 64%, while most other solvents did not lead to any product. A survey of ligands showed that dmbpy gave a slightly better result (71%; entry 6 in Table 1). The screening of nickel catalysts demonstrated that other nickel salts led to lower yields (entry 11 in Table 1), and precoordinated NiCl_2_·dmbpy gave a slightly lower yield of 69% (entry 12 in Table 1). After adjustment of the amount of the three substrates, the corresponding product was obtained in 89% yield using 1.0 equiv of 1,6-enyne and 2.0 equiv of sulfinate and iodoarene (entry 13 in Table 1). Furthermore, control experiments suggested that the nickel catalyst, ligand, and light irradiation were all crucial to the three-component cyclization, as no product formation was observed in the absence of either of them (entries 14–16 in Table 1). A 1 mmol scale reaction can afford the cyclized product in 77% yield under the optimized conditions (entry 17 in Table 1).

**Table 1:**
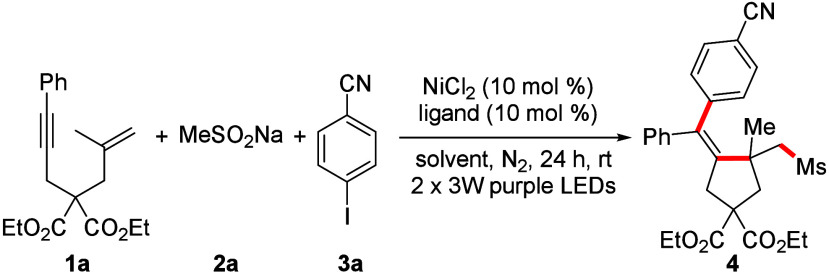
Optimization of Conditions^a^

entry	ligand	solvent	yield (%)
1	dtbpy	DMF	46
2	dtbpy	DMA	47
3	dtbpy	DMSO	64
4	dtbpy	MeCN	0
5	dtbpy	DCM	0
6	dmbpy	DMSO	71
7	**L1**	DMSO	53
8	**L2**	DMSO	58
9	dombpy	DMSO	66
10	**L3**	DMSO	32
11^b^	dmbpy	DMSO	16–63
12^c^		DMSO	69
13^d^	dmbpy	DMSO	89
14^d^^,^^e^	dmbpy	DMSO	0
15^d^^,^^f^	dmbpy	DMSO	0
16^d^		DMSO	0
17^d^^,^^g^	dmbpy	DMSO	77

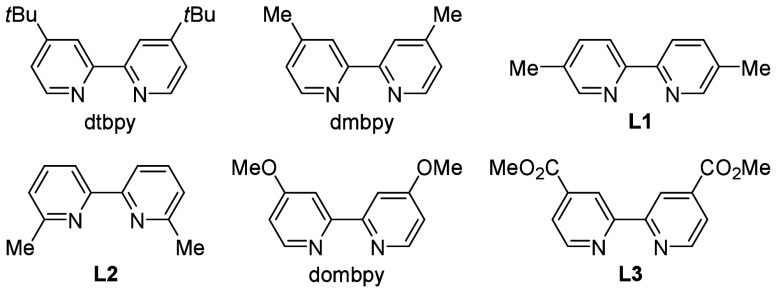

a Reaction conditions: compound **1a** (0.4 mmol, 2.0 equiv), compound **2a** (0.2 mmol), compound **3a** (0.4 mmol, 2.0 equiv), NiCl_2_ (10 mol %), ligand (10 mol %), solvent (2 mL), N_2_, room temperature, 2 × 3 W purple light-emitting diodes (LEDs), and 24 h.

b NiBr_2_, NiI_2_, or NiCl_2_·DME as the nickel catalyst.

c NiCl_2_·dmbpy as the nickel catalyst.

d Compound **1a** (0.2 mmol), compound **2a** (0.4 mmol, 2.0 equiv), and compound **3a** (0.4 mmol, 2.0 equiv).

e Without a nickel catalyst.

f In the dark.

g A 1 mmol scale reaction.

We then proceeded to examine the substrate scope of the three-component reaction with the optimized reaction condition (Table 2). First, a series of iodoarenes embedding electron-withdrawing substituents, such as cyano (**4**), trifluoromethyl (**5**), acetyl (**6**), and ester (**7**), at the *para* position of the phenyl rings provided the corresponding products with good to excellent yields. As for electron-neutral or electron-donating substitutions (**8**–**11**), relatively lower yields were obtained. The *meta*-substituted aryl iodides gave the products in good to excellent yields. Notably, this catalytic transformation could be smoothly applied to yield fused aromatic or heteroaromatic product (**22** and **23**). Next, the scope of 1,6-enynes was explored using 4-iodobenzonitrile as the coupling partner to show the scope versatility. An array of 1,6-enynes revealed little impact imposed by the electronic and steric effects. Electron-withdrawing and electron-donating substituents all underwent the three-component cyclization smoothly (**27**–**47**), delivering the desired products in good to excellent yields (up to 95%, **33**). Of note, in contrast to our previous report, the *ortho*-substituted enyne substrates were tolerated in this transformation and gave the corresponding products (**42**–**44**) in high yields. This arylsulfonation reaction was carried out successfully for the 1,6-enynes bearing fused arene (**45**) or heterocyclic arenes (**46** and **47**). When the O-tethered enyne substrate was used, the corresponding product was obtained in a low yield (35% for compound **48**) due to the absence of the Thorpe–Ingold effect. Finally, cyclopropylsufinate was suitable for this transformation and gave the cyclopropylsulfonyl product in 75% yield (**49**).

**Table 2:**
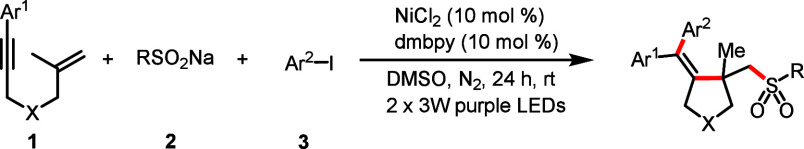

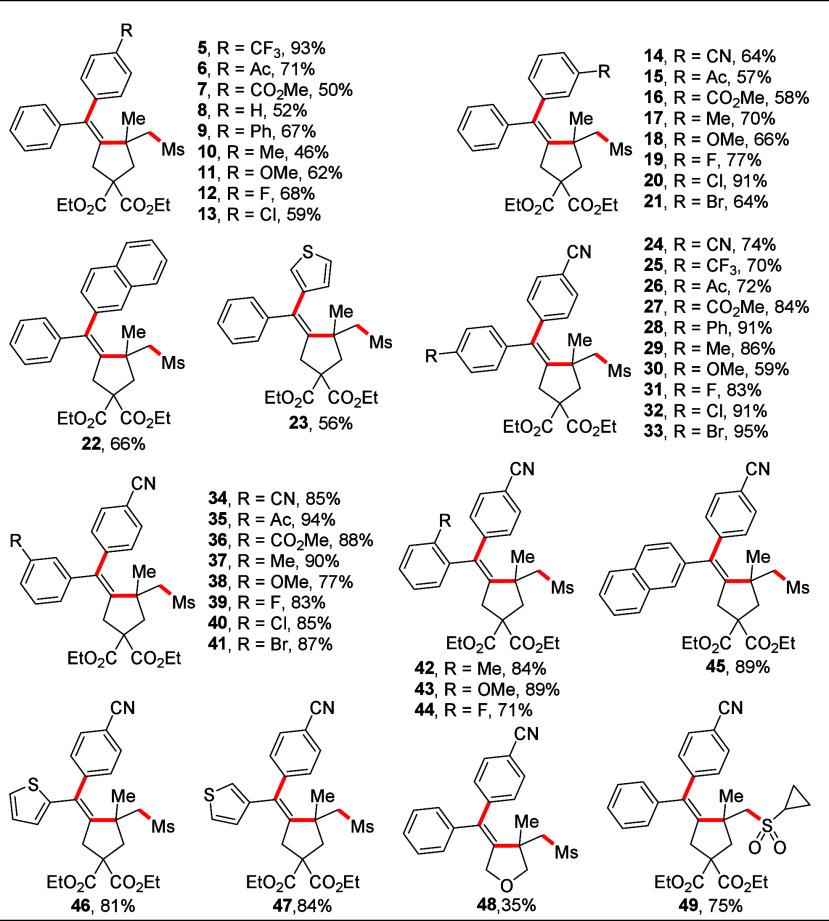
Substrate Scope^a^

a Reaction conditions: compound **1** (0.4 mmol, 2.0 equiv), compound **2** (0.2 mmol), compound **3** (0.4 mmol, 2.0 equiv), NiCl_2_ (10 mol %), dmbpy (10 mol %), DMSO (2 mL), N_2_, room temperature, 2 × 3 W purple LEDs, and 24 h.

To verify the reaction mechanism, a radical clock experiment was carried out using compound **1aa** as a radical scavenger. A sulfonyl radical adduct can be obtained under the optimized conditions, which suggests the generation of a sulfonyl radical. Then, an intermittent irradiation experiment showed that this annulation required constant irradiation and does not involve a light-initiated radical chain process (Figure 2b). At the same time, it can be found that this reaction was accelerated in the next “light-on” period. Additionally, a time-dependent experiment was executed (Figure 2c), and this photoredox process behaved at an initial stage at the earliest 6 h (7.9% of compound **4**). Next, the yield rose to 70.6% within 14 h in the next acceleration stage. Finally, the yield of compound **4** was only increased by 8.5% in the last 4 h due to the consumption of the reactants. The result indicated that this reaction involved an autopromotion step.

**Figure 2:**
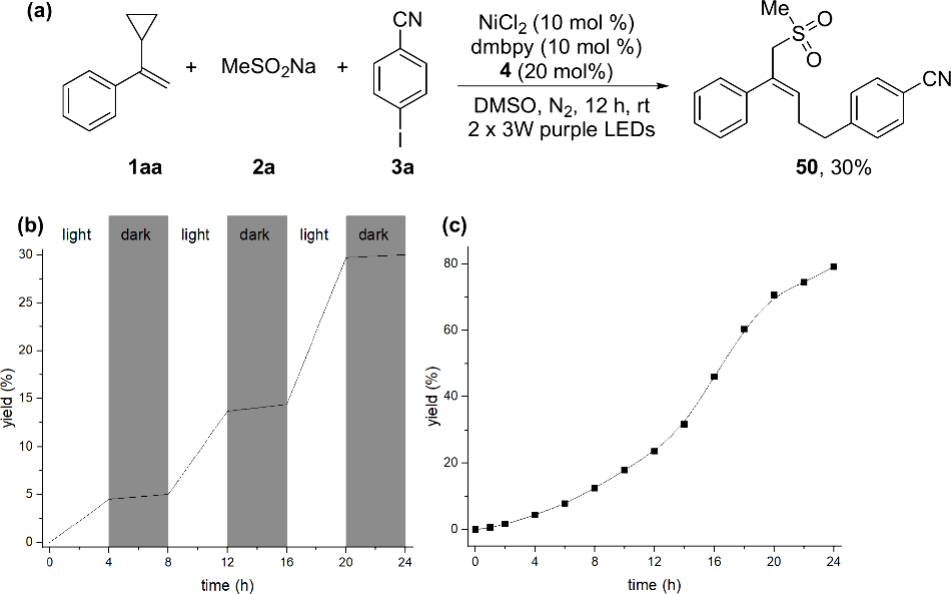
Mechanism studies: (a) radical trap experiment, (b) light on/off experiment, and (c) reaction progress monitoring the yield.

In summary, we studied the photoactive sulfonylallene–nickel complex structure through DFT/TD-DFT calculations, structural analysis, IRI analysis, and spin population analysis. The Ni(II)–diphenylmethylene radical species, which was generated by the coordination of diphenylvinyl and sulfonyl groups with Ni(I), was the simulated photoinitiator. The structure of this photoactive species guided us to develop a photocatalyst-free, nickel-catalyzed autopromoted three-component arylsulfonation of 1,6-enynes. This methodology demonstrated high catalytic reactivity, mild reaction conditions, and good tolerance toward substrates of different functional groups. Further elaboration of the synthetic application and modification of this novel photoactive nickel catalyst structure is currently underway in our laboratory.

## Data Availability

The data underlying this study are available in the published article and its Supporting Information.

## Supporting Information Available

Experimental procedures, optimization of reaction conditions, mechanistic investigations, computational details, characterization data, and nuclear magnetic resonance (NMR) spectra (PDF)

## References

[ref1] aNarayanam J. M. R.; Stephenson C. R. J. Visible Light Photoredox Catalysis: Applications in Organic Synthesis. Chem. Soc. Rev. 2011, 40, 102–113. 10.1039/B913880N. 20532341

[ref2] aSu Y.; Straathof N. J. W.; Hessel V.; Noël T. Photochemical Transformations Accelerated in Continuous-Flow Reactors: Basic Concepts and Applications. Chem. - Eur. J. 2014, 20, 10562–10589. 10.1002/chem.201400283. 25056280

[ref3] aSkubi K. L.; Blum T. R.; Yoon T. P. Dual Catalysis Strategies in Photochemical Synthesis. Chem. Rev. 2016, 116, 10035–10074. 10.1021/acs.chemrev.6b00018. 27109441 PMC5083252

[ref4] aParasram M.; Gevorgyan V. Visible Light-Induced Transition Metal-Catalyzed Transformations: Beyond Conventional Photosensitizers. Chem. Soc. Rev. 2017, 46, 6227–6240. 10.1039/C7CS00226B. 28799591 PMC5643232

[ref5] Shields B. J.; Kudisch B.; Scholes G. D.; Doyle A. G. Long-Lived Charge-Transfer States of Nickel(II) Aryl Halide Complexes Facilitate Bimolecular Photoinduced Electron Transfer. J. Am. Chem. Soc. 2018, 140, 3035–3039. 10.1021/jacs.7b13281. 29400956 PMC6698182

[ref6] Lim C. H.; Kudisch M.; Liu B.; Miyake G. M. C–N Cross-Coupling via Photoexcitation of Nickel–Amine Complexes. J. Am. Chem. Soc. 2018, 140, 7667–7673. 10.1021/jacs.8b03744. 29787252 PMC6034616

[ref7] Abdiaj I.; Fontana A.; Gomez M. V.; de la Hoz A.; Alcázar J. Visible-Light-Induced Nickel-Catalyzed Negishi Cross-Couplings by Exogenous-Photosensitizer-Free Photocatalysis. Angew. Chem., Int. Ed. 2018, 57, 8473–8477. 10.1002/anie.201802656. 29566297

[ref8] Yang L.; Lu H.-H.; Lai C.-H.; Li G.; Zhang W.; Cao R.; Liu F.; Wang C.; Xiao J.; Xue D. Light-Promoted Nickel Catalysis: Etherification of Aryl Electrophiles with Alcohols Catalyzed by a NiII-Aryl Complex. Angew. Chem., Int. Ed. 2020, 59, 12714–12719. 10.1002/anie.202003359. 32281220

[ref9] Hu D.-D.; Gao Q.; Dai J.-C.; Cui R.; Li Y.-B.; Li Y.-M.; Zhou X.-G.; Bian K.-J.; Wu B.-B.; Zhang K.-F.; Wang X.-S.; Li Y. Visible-Light-Induced, Autopromoted Nickel-Catalyzed Three-Component Arylsulfonation of 1,3-Enynes and Mechanistic Insights. Sci. China Chem. 2022, 65, 753–761. 10.1007/s11426-021-1193-5.

[ref10] aPavan M. J.; Fridman H.; Segalovich G.; Shames A. I.; Lemcoff N. G.; Mokari T. Photoxidation of Benzyl Alcohol with Heterogeneous Photocatalysts in the UV Range: The Complex Interplay with the Autoxidative Reaction. ChemCatChem. 2018, 10, 2541–2545. 10.1002/cctc.201800284.

[ref11] Lu T.; Chen Q. Interaction Region Indicator: A Simple Real Space Function Clearly Revealing Both Chemical Bonds and Weak Interactions. Chem. Methods 2021, 1, 231–239. 10.1002/cmtd.202100007.

