# Supplementary material for: Photoinduced Autopromoted Ni-Catalyzed Three-Component Arylsulfonation Inspired by Density Functional Theory/Time-Dependent Density Functional Theory-Simulated Photoactive Nickel Species
Source: Org Lett. 2024 Dec 23;27(1):217–22. doi: 10.1021/acs.orglett.4c04222 (PMC11731393; doi:10.1021/acs.orglett.4c04222)

Supporting Information

Photoinduced Autopromoted Ni-Catalyzed Three-Component Arylsulfonation Inspired by Density Functional Theory/Time- Dependent Density Functional Theory-Simulated Photoactive Nickel Species

Feng Zhang,[a,‡] Xiu-Fen Cheng,[bc,‡] Xiaolin Liang,[a] Duo-Duo Hu,[c] Qian Gao,[c] Hongliang Wang,[a,*] Peng Wu,[bd,*] Yan Li[ac,*]

[a]Chemical Biology Center, School of Pharmaceutical Sciences & Institute of Materia Medica, Shandong First Medical University & Shandong Academy of Medical Sciences, Jinan 250117, Shandong, China

[b]Department of Chemical Biology, Max Planck Institute of Molecular Physiology, Dortmund 44227, Germany

[c]Department of Chemistry, University of Science and Technology of China, 96 Jinzhai Road, Hefei, Anhui 230026, China

[d]Chemical Genomics Centre, Max Planck Institute of Molecular Physiology, Dortmund 44227, Germany

‡These authors contributed equally.

*Email: hliang_18@sdfmu.edu.cn; peng.wu@mpi-dortmund.mpg.de; liyann@sdfmu.edu.cn.

Contents

General information: S3

Experimental procedure S4

General procedure for preparation of 1,6-enynes S4

General procedure for visible light induced arylsulfonation of 1,6-enynes S8

Radical trap experiments S27

Light on/off experiment S27

Time-dependent experiment S28

Computational Details S29

References S47

NMR spectra of new compounds (^{1}H, ^{13}C and ^{19}F NMR) S48

General information:

NMR spectra were recorded on Bruker-400 (400 MHz for ^{1}H; 100 MHz for ^{13}C) or Bruker AVANCE III 800 (800 MHz for ^{1}H; 200 MHz for ^{13}C). High resolution mass spectra (HRMS) were recorded on P-SIMS-Gly of Bruker Daltonics Inc. using ESI-TOF (electrospray ionization-time of flight) or Micromass GCT using EI (electron impact). SuperDry dimethyl sulfoxide, 4,4-dimethyl-2,2'-bipyridine, nickel(II) chloride, sodium methanesulfinate and 4-iodobenzonitrile were purchased from Adamas and used as received. Unless otherwise stated, all other reagents and starting materials are purchased from commercial sources and used without further purification.

Experimental procedure

Substrates **1a**,[2] **1b**,[1] **1d**,[1] **1e**,[1] **1f**,[2] **1g**,[1] **1h**,[1] **1i**,[3] **1j**,[2] **1k**,[2] **1o**,[3] **1q**,[3] **1t**,[1] **1v**,[3] **1w**,[1] **1x**,[3] **1z**[4] are known compounds and have been synthesized according to the previously reported methods. Substrates **1c**, **1l**, **1m**, **1n**, **1p**, **1r**, **1s**, **1u**, **1y** are synthesized by the following method.[5]

General procedure for preparation of 1,6-enynes

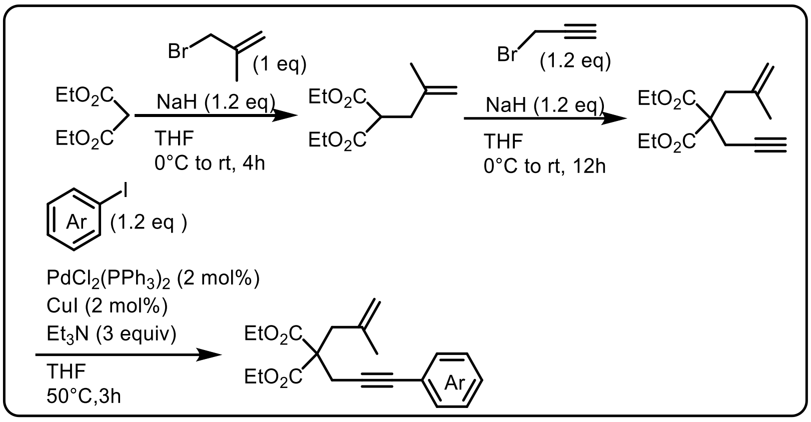

A 100ml round-bottom flask containing a magneton was charged with NaH (60% in oil, 1.0 g, 24 mmol, 1.2 equiv) and THF (40 mL). The solution was cooled to 0 °C by an ice/water bath. Diethyl malonate (4.8 g, 30 mmol, 1.5 equiv) was added to the flask, and the mixture was stirred for 30 min at 0 °C. 3-Bromo-2-methylprop-1-ene (2.7 g, 20 mmol, 1.0 equiv) was added to the flask. The resulting mixture was warmed to room temperature and stirred for 4 h. Water (20 mL) was added to the flask, and the organic materials were extracted with EtOAc (50 mL x 3). The combined organic layer was washed with water (50 mL x 2) and brine (50 mL x 1), and dried over anhydrous Na_2SO_4. After removal of the insoluble materials by filtration，the product was purified by column chromatography on silica gel. Diethyl 2-(2-methylallyl)malonate (2.5 g, 59%) was obtained.

A 100ml round-bottom flask containing a magneton, was charged with NaH (60% in oil, 0.59 g, 14 mmol, 1.2 equiv) and THF (40 mL). The solution was cooled to 0 °C by an ice/water bath. Diethyl 2-(2-methylallyl)malonate (2.5 g, 12 mmol, 1.0 equiv) was added to the flask, and the mixture was stirred for 30 min at 0 °C. Propargyl bromide (1.7 g, 14 mmol, 1.2 equiv) was added to the flask. The resulting mixture was warmed to room temperature and stirred for 12 h. Saturated NH_4Cl (30 mL) was added to the flask, and the organic materials were extracted with EtOAc (50 mL x 3). The combined organic layer was washed with water (50 mL x 2) and brine (50 mL x 1), and dried over anhydrous Na_2SO_4. After removal of the insoluble materials by filtration，the product was purified by column chromatography on silica gel.

$PdCl_2(PPh_3)_2$ (140 mg, 0.2 mmol, 2 mol%) and CuI (38 mg, 0.2 mmol, 2 mol%) were added to a 100ml round-bottom flask containing magnetons to replace nitrogen,

THF (20 mL), Et_3N (2.9 g, 29 mmol, 2.9 equiv) and iodobenzene (12 mmol, 1.2 equiv) were added. The enyne (10 mmol, 1.0 equiv) was added dropwise to the flask and the resulting mixture was stirred at 50 °C for 3 hours. After cooling to room temperature, the insoluble material is removed by diatomite pad filtration. The filtrate was concentrated and purified by silica gel column chromatography.

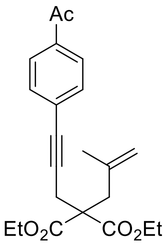

1c

The product **1c** was synthesized from 4-iodoacetophenone (2.95 g, 12 mmol) and purified with silica gel chromatography (PE/EA = 10:1) as a colorless oil. ^{1}H NMR (400 MHz, $CDCl_3$) δ 7.88 – 7.84 (m, 2H), 7.43 (d, *J* = 8.4 Hz, 2H), 4.95 – 4.86 (m, 2H), 4.28-4.19 (m, 4H), 3.06 (s, 2H), 2.89 (s, 2H), 2.58 (s, 3H), 1.69 (s, 3H), 1.26 (t, *J* = 7.1 Hz, 6H). ^{13}C NMR (151 MHz, $CDCl_3$) δ 197.5, 170.3, 140.0, 136.1, 131.8, 128.3 (2C), 116.5, 88.7, 83.2, 61.8, 56.6, 39.8, 26.7, 23.7, 23.4, 14.2. HRMS ESI (m/z): $[M+Na]^+$ calcd. for $C_{22}H_{26}O_5Na$: 393.1672, found: 393.1682.

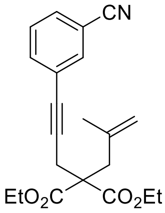

1l

The product **1l** was synthesized from 3-iodobenzonitrile (2.75 g, 12 mmol) and purified with silica gel chromatography (PE/EA = 10:1) as a colorless oil. ^{1}H NMR (400 MHz, $CDCl_3$) δ 7.63 – 7.60 (m, 1H), 7.59 – 7.53 (m, 2H), 7.39 (t, *J* = 7.8 Hz, 1H), 4.95 – 4.84 (m, 2H), 4.28-4.19 (m, 4H), 3.04 (s, 2H), 2.87 (s, 2H), 1.68 (s, 3H), 1.26 (t, *J* = 7.1 Hz, 6H). ^{13}C NMR (151 MHz, $CDCl_3$) δ 170.2, 140.0, 135.8, 135.0, 131.3, 129.3, 124.9, 118.2, 116.5, 112.8, 87.9, 81.6, 61.9, 56.5, 39.8, 23.5, 23.4, 14.2. HRMS ESI (m/z): $[M+Na]^+$ calcd. for $C_{21}H_{23}NO_4Na$: 376.1519, found: 376.1519.

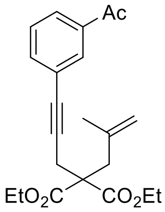

1m

The product **1m** was synthesized from 3-iodoacetophenone (2.95 g, 12 mmol) and purified with silica gel chromatography (PE/EA = 10:1) as a colorless oil. ^{1}H NMR (400 MHz, $CDCl_3$) δ 7.92 (t, *J* = 1.5 Hz, 1H), 7.88 – 7.85 (m, 1H), 7.56 – 7.53 (m, 1H), 7.38 (t, *J* = 7.8 Hz, 1H), 4.95 – 4.88 (m, 2H), 4.28 – 4.17 (m, 4H), 3.06 (s, 2H),

2.90 (s, 2H), 2.59 (s, 3H), 1.70 (s, 3H), 1.27 (t, J = 7.1 Hz, 6H). ^{13}C NMR (151 MHz, $CDCl_3$) δ 197.6, 170.3, 140.1, 137.2, 136.1, 131.5, 128.7, 127.7, 124.0, 116.5, 86.2, 82.8, 61.8, 56.6, 39.7, 26.8, 23.6, 23.4, 14.2. HRMS ESI (m/z): $[M+Na]^+$ calcd. for $C_{22}H_{26}O_5Na$: 393.1672, found: 393.1671.

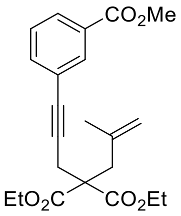

1n

The product **1n** was synthesized from methyl 3-iodobenzoate (3.14 g, 12 mmol) and purified with silica gel chromatography (PE/EA = 10:1) as a colorless oil. ^{1}H NMR (400 MHz, $CDCl_3$) δ 8.02 (s, 1H), 7.94 (d, J = 7.8 Hz, 1H), 7.53 (d, J = 7.7 Hz, 1H), 7.35 (t, J = 7.8 Hz, 1H), 4.91 (d, J = 18.0 Hz, 2H), 4.28-4.19 (m, 4H), 3.92 – 3.89 (m, 3H), 3.05 (s, 2H), 2.89 (s, 2H), 1.70 (s, 3H), 1.27 (t, J = 7.1 Hz, 6H). ^{13}C NMR (151 MHz, $CDCl_3$) δ 170.3, 166.5, 140.1, 136.0, 132.7, 130.4, 129.1, 128.5, 123.8, 116.4, 86.0, 82.8, 61.8, 56.7, 52.4, 39.7, 23.6, 23.4, 14.2. HRMS ESI (m/z): $[M+Na]^+$ calcd. for $C_{22}H_{26}O_6Na$: 409.1622, found: 409.1623.

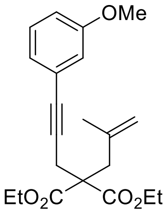

1p

The product **1p** was synthesized from 3-iodoanisole (2.81g, 12 mmol) and purified with silica gel chromatography (PE/EA = 20:1) as a colorless oil. ^{1}H NMR (400 MHz, $CDCl_3$) δ 7.18 (t, J = 7.9 Hz, 1H), 6.96 (d, J = 7.6 Hz, 1H), 6.90 – 6.88 (m, 1H), 6.83 (dd, J = 8.3, 2.5 Hz, 1H), 4.94 – 4.88 (m, 2H), 4.27-4.17 (m, 4H), 3.78 (s, 3H), 3.04 (s, 2H), 2.89 (s, 2H), 1.70 (s, 3H), 1.27 (t, J = 7.1 Hz, 6H). ^{13}C NMR (151 MHz, $CDCl_3$) δ 170.4, 159.3, 140.2, 129.4, 124.4, 124.3, 116.6, 116.4, 114.4, 84.8, 83.7, 61.7, 56.7, 55.3, 39.7, 23.6, 23.4, 14.2. HRMS ESI (m/z): $[M+Na]^+$ calcd. for $C_{21}H_{26}O_5Na$: 381.1672, found: 381.1675.

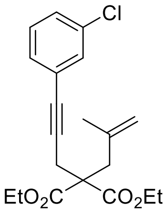

1r

The product **1r** was synthesized from 3-chloroiodobenzene (2.86 g, 12 mmol) and purified with silica gel chromatography (PE/EA = 20:1) as a colorless oil. ^{1}H NMR (400 MHz, $CDCl_3$) δ 7.33 (d, J = 1.6 Hz, 1H), 7.24 (dt, J = 5.9, 1.5 Hz, 2H), 7.21 (d, J

= 7.5 Hz, 1H), 4.94 – 4.87 (m, 2H), 4.29 – 4.15 (m, 4H), 3.04 (s, 2H), 2.88 (s, 2H), 1.69 (s, 3H), 1.27 (t, *J* = 7.1 Hz, 6H). ^{13}C NMR (151 MHz, $CDCl_3$) δ 170.3, 140.1, 134.1, 131.5, 129.9, 129.5, 128.3, 125.0, 116.4, 86.3, 82.5, 61.8, 56.6, 39.7, 23.5, 23.4, 14.1. HRMS ESI (m/z): $[M+Na]^+$ calcd. for $C_{20}H_{23}ClO_4Na$: 385.1177, found: 385.1176.

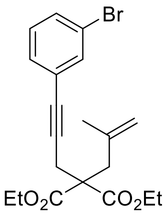

1s

The product **1s** was synthesized from 1-bromo-3-iodobenzene (3.39 g, 12 mmol) and purified with silica gel chromatography (PE/EA = 20:1) as a colorless oil. ^{1}H NMR (400 MHz, $CDCl_3$) δ 7.49 (t, *J* = 1.6 Hz, 1H), 7.42 – 7.38 (m, 1H), 7.28 (d, *J* = 7.8 Hz, 1H), 7.13 (t, *J* = 7.9 Hz, 1H), 4.94 – 4.86 (m, 2H), 4.28 – 4.15 (m, 4H), 3.03 (s, 2H), 2.88 (s, 2H), 1.69 (s, 3H), 1.26 (t, *J* = 7.1 Hz, 6H). ^{13}C NMR (151 MHz, $CDCl_3$) δ 170.3, 140.0, 134.4, 131.2, 130.3, 129.8, 125.3, 122.1, 116.4, 86.5, 82.3, 61.8, 56.6, 39.7, 23.5, 23.4, 14.2. HRMS ESI (m/z): $[M+Na]^+$ calcd. for $C_{20}H_{23}BrO_4Na$: 429.0672, found: 429.0671.

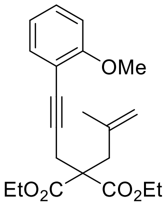

1u

The product **1u** was synthesized from 2-iodoanisole(2.81 g, 12 mmol) and purified with silica gel chromatography (PE/EA = 20:1) as a colorless oil. ^{1}H NMR (400 MHz, $CDCl_3$) δ 7.33 (dd, *J* = 7.5, 1.7 Hz, 1H), 7.23 (dd, *J* = 8.0, 1.4 Hz, 1H), 6.88 – 6.81 (m, 2H), 4.98 – 4.92 (m, 2H), 4.27-4.17 (m, 4H), 3.84 (s, 3H), 3.10 (s, 2H), 2.93 (s, 2H), 1.70 (s, 3H), 1.26 (t, *J* = 7.1 Hz, 6H). ^{13}C NMR (151 MHz, $CDCl_3$) δ 170.4, 160.2, 140.3, 133.6, 129.4, 120.4, 116.4, 112.6, 110.6, 89.0, 80.0, 61.7, 56.8, 55.7, 39.6, 23.9, 23.4, 14.1. HRMS ESI (m/z): $[M+H]^+$ calcd. for $C_{21}H_{27}O_5$: 359.1853, found: 359.1859.

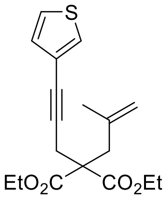

1y

The product **1y** was synthesized from 3-iodothiophene (2.52 g, 12 mmol) and purified with silica gel chromatography (PE/EA = 20:1) as a colorless oil. ^{1}H NMR (400 MHz, $CDCl_3$) δ 7.34 (dd, *J* = 3.0, 1.1 Hz, 1H), 7.22 (dd, *J* = 5.0, 3.0 Hz, 1H), 7.03 (dd, *J* = 5.0, 1.1 Hz, 1H), 4.94 – 4.87 (m, 2H), 4.27-4.17 (m, 4H), 3.02 (s, 2H),

2.88 (s, 2H), 1.69 (s, 3H), 1.26 (t, *J* = 7.1 Hz, 6H). ^{13}C NMR (151 MHz, $CDCl_3$) δ 170.4, 140.2, 130.0, 128.3, 125.2, 122.4, 116.3, 84.4, 78.8, 61.7, 56.7, 39.7, 23.6, 23.4, 14.1. HRMS ESI (m/z): $[M+Na]^+$ calcd. for $C_{18}H_{22}O_4SNa$: 357.1131, found: 357.1151.

The experiment setup

The light source was purchased from Wuhan GeAo Chemical Technology Co., Ltd. (Four-hole photoreactor). The nominal wavelength is 395-415 nm. The emission spectrum of this purple LEDs was shown below. The material of the irradiation vessel is borosilicate glass, and the distance from the light source to the irradiation vessel is 4 mm in average without filter.

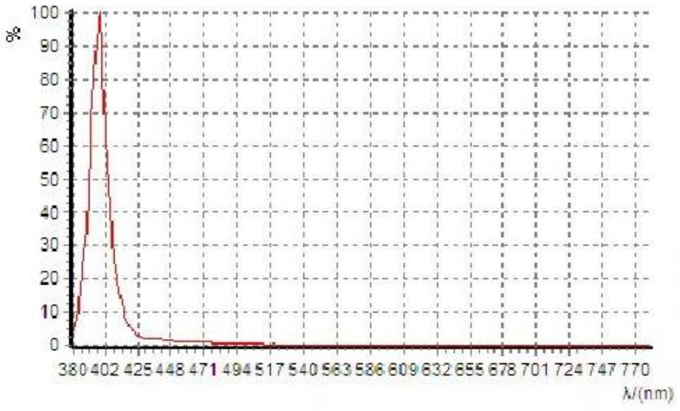

General procedure for visible light induced arylsulfonation of 1,6-enynes

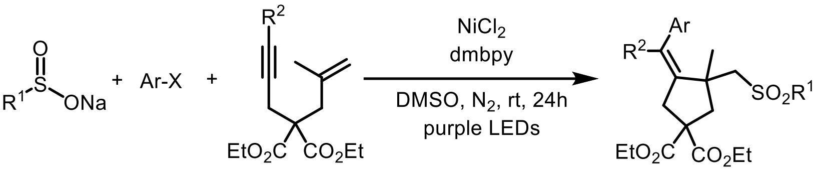

In a 10 mL oven-dried Schlenk tube, 2.0 mL DMSO was added to a mixture of $NiCl_2$ (1.3 mg, 0.01 mmol, 10 mol%), dmbpy(1.8 mg, 0.01 mmol, 10 mol%), sodium sulfinate (0.2 mmol, 2.0 equiv), aryl halide (0.2 mmol, 2.0 equiv) and 1,6-enyne 1 (0.1 mmol, 1.0 equiv) under N_2 atmosphere. The tube was sealed with a Teflon lined cap and the reaction was stirred and irradiated with 6 W purple LED lamps (395-415 nm, with cooling fan to keep the reaction temperature near 25 °C) for 24 h. The reaction mixture was quenched with water and extracted with EtOAc (10 mL x 2). The combined organic phase was washed with brine (20 mL), dried over anhydrous Na_2SO_4, concentrated in vacuo. The residue as purified by flash column chromatography to give the product.

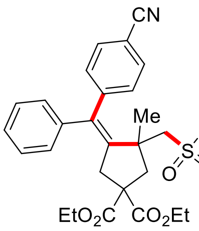

4

The product **4** was purified with silica gel chromatography (PE/EA = 2:1) as a white solid (0.2 mmol scaled, 90.6 mg, 89%); m.p. 108.6-110.5 °C. ^{1}H NMR (400 MHz, $CDCl_3$) δ 7.60 (d, *J* = 8.2 Hz, 2H), 7.36 (dd, *J* = 15.3, 8.0 Hz, 4H), 7.29 (d, *J* = 7.1 Hz, 1H), 7.26 – 7.23 (m, 2H), 4.24 – 4.15 (m, 4H), 3.24 (d, *J* = 14.3 Hz, 1H), 3.13 (d, *J* = 14.0 Hz, 1H), 3.03 – 2.94 (m, 3H), 2.75 (s, 3H), 2.50 (d, *J* = 14.3 Hz, 1H), 1.31 (s, 3H), 1.28 – 1.23 (m, 6H). ^{13}C NMR (101 MHz, $CDCl_3$) δ 171.5, 171.4, 148.2, 144.9, 139.7, 136.4, 132.6, 129.2, 128.7, 128.6, 127.8, 118.8, 110.5, 62.8, 62.0, 61.8, 57.5, 45.9, 44.7, 44.1, 40.6, 28.7, 14.0 (2C). HRMS ESI (m/z): $[M+Na]^+$ calcd. for $C_{28}H_{31}NO_6SNa$: 532.1764, found: 532.1768.

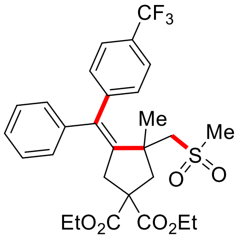

5

The product **5** was purified with silica gel chromatography (PE/EA = 2:1) as a white solid (0.2 mmol scaled, 102.6mg, 93%); m.p. 122.8-124.2 °C. ^{1}H NMR (500 MHz, $CDCl_3$) δ 7.55 (d, *J* = 8.2 Hz, 2H), 7.35 (dd, *J* = 17.4, 7.9 Hz, 4H), 7.27 – 7.23 (m, 3H), 4.23-4.16 (m, 4H), 3.22 (d, *J* = 14.3 Hz, 1H), 3.15 (d, *J* = 14.0 Hz, 1H), 3.06 (d, *J* = 16.9 Hz, 1H), 3.01 – 2.94 (m, 2H), 2.74 (s, 3H), 2.51 (d, *J* = 14.3 Hz, 1H), 1.34 (s, 3H), 1.27 – 1.23 (m, 6H). ^{13}C NMR (126 MHz, $CDCl_3$) δ 171.6, 171.5, 147.2, 144.6, 140.2, 136.8, 129.2, 128.9 (q, *J* = 32.4 Hz), 128.6, 128.2, 127.7, 125.7 (q, *J* = 3.7 Hz), 124.1 (q, *J* = 272.5 Hz), 63.0, 62.0, 61.8, 57.6, 46.1, 44.7, 44.1, 40.5, 28.6, 14.0 (2C). ^{19}F NMR (376 MHz, $CDCl_3$) δ -62.5 (s, 3F). HRMS ESI (m/z): $[M+Na]^+$ calcd. for $C_{28}H_{31}F_3O_6SNa$: 575.1686, found: 575.1698.

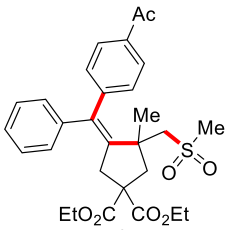

6

The product **6** was purified with silica gel chromatography (PE/EA = 2:1) as a white s olid (0.2 mmol scaled, 74.6 mg, 71%); m.p. 104.5-106.2 °C. ^{1}H NMR (500 MHz, CD Cl$_3$) δ 7.89 (d, *J* = 8.2 Hz, 2H), 7.33 (t, *J* = 7.6 Hz, 4H), 7.25 (d, *J* = 8.2 Hz, 3H), 4.21 -4.15 (m, 4H), 3.21 (d, *J* = 14.3 Hz, 1H),3.16 (d, *J* = 14.0 Hz, 1H), 3.07 (d, *J* = 16.9 H

z, 1H), 2.99 (dd, J = 15.5, 5.8 Hz, 2H), 2.74 (s, 3H), 2.56 (s, 3H), 2.51 (d, J = 14.3 Hz, 1H), 1.35 (s, 3H), 1.27 – 1.22 (m, 6H).^{13}C NMR (126 MHz, $CDCl_3$) δ 197.7, 171.6, 171.5, 148.5, 144.5, 140.1, 137.1, 135.5, 129.2, 128.8, 128.5, 128.1, 127.7, 63.0, 61.9, 61.8, 57.6, 46.1, 44.7, 44.1, 40.5, 28.5, 26.6, 14.1, 14.0. HRMS ESI (m/z): $[M+Na]^+$ calcd. for $C_{29}H_{34}O_7SNa$: 549.1917, found: 549.1919.

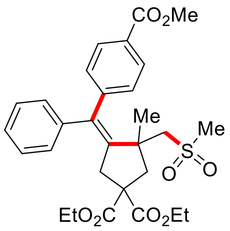

7

The product **7** was purified with silica gel chromatography (PE/EA = 2:1) as a white solid (0.2 mmol scaled, 54.2 mg, 50%); m.p. 110.2-112.8 °C. ^{1}H NMR (400 MHz, $CDCl_3$) δ 7.97 (d, J = 8.3 Hz, 2H), 7.36 – 7.28 (m, 5H), 7.26 – 7.23 (m, 2H), 4.22 – 4.15 (m, 4H), 3.89 (s, 3H), 3.18 (dd, J = 17.7, 14.2 Hz, 2H), 3.07 (d, J = 17.0 Hz, 1H), 3.01 – 2.94 (m, 2H), 2.73 (s, 3H), 2.50 (d, J = 14.3 Hz, 1H), 1.35 (s, 3H), 1.27-1.21 (m, 6H). ^{13}C NMR (101 MHz, $CDCl_3$) δ 171.6, 171.5, 166.8, 148.3, 144.4, 140.2, 137.2, 130.0, 129.2, 128.5, 127.9, 127.6, 63.0, 61.9, 61.7, 57.6, 52.1, 46.0, 44.6, 44.1, 40.4, 28.5, 14.0 (2C). HRMS ESI (m/z): $[M+Na]^+$ calcd. for $C_{29}H_{34}O_8SNa$: 565.1867, found: 565.1876.

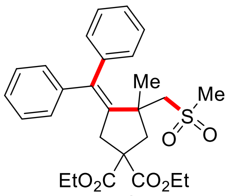

8

The product **8** was purified with silica gel chromatography (PE/EA = 4:1) as a white solid (0.2 mmol scaled, 48.4 mg, 52%); m.p. 120.8-122.6 °C. ^{1}H NMR (800 MHz, $CDCl_3$) δ 7.32 – 7.30 (m, 2H), 7.28 (t, J = 7.7 Hz, 2H), 7.25 – 7.20 (m, 5H), 7.18 (t, J = 7.4 Hz, 1H), 4.22 – 4.15 (m, 4H), 3.18 (dd, J = 14.2, 9.1 Hz, 2H), 3.12 – 3.09 (m, 1H), 3.00 – 2.97 (m, 2H), 2.71 (s, 3H), 2.50 (d, J = 14.4 Hz, 1H), 1.37 (s, 3H), 1.27-1.21 (m, 6H). ^{13}C NMR (201 MHz, $CDCl_3$) δ 171.8, 171.6, 143.7, 141.0, 138.1, 129.1, 128.6, 128.4, 127.7, 127.3, 126.7, 63.3, 61.8, 61.7, 57.6, 46.2, 44.6, 44.1, 40.4, 28.4, 14.0 (2C). HRMS ESI (m/z): $[M+Na]^+$ calcd. for $C_{27}H_{32}O_6SNa$: 507.1812, found: 507.1823.

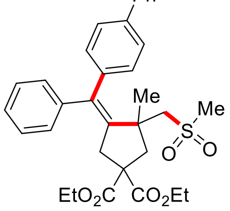

9

The product **9** was purified with silica gel chromatography (PE/EA = 3:1) as a white solid (0.2 mmol scaled, 75.0mg, 67%); m.p. 128.2-130.3 °C. ^{1}H NMR (800 MHz, $CDCl_3$) δ 7.54 (d, *J* = 7.3 Hz, 2H), 7.51 (d, *J* = 8.2 Hz, 2H), 7.41 (t, *J* = 7.7 Hz, 2H), 7.35 – 7.31 (m, 3H), 7.29 – 7.26 (m, 5H), 4.22 – 4.17 (m, 4H), 3.21 – 3.16 (m, 3H), 3.06 (d, *J* = 17.1 Hz, 1H), 3.01 (d, *J* = 14.0 Hz, 1H), 2.72 (s, 3H), 2.52 (d, *J* = 14.3 Hz, 1H), 1.38 (s, 3H), 1.27 – 1.23 (m, 6H). ^{13}C NMR (201 MHz, $CDCl_3$) δ 171.8, 171.6, 143.9, 142.6, 141.0, 140.7, 139.5, 137.7, 129.2, 128.7, 128.4, 128.2, 127.4, 127.3 (2C), 127.0, 63.3, 61.9, 61.7, 57.7, 46.2, 44.6, 44.1, 40.5, 28.4, 14.0 (2C). HRMS ESI (m/z): $[M+Na]^+$ calcd. for $C_{33}H_{36}O_6SNa$: 583.2125, found: 583.2134.

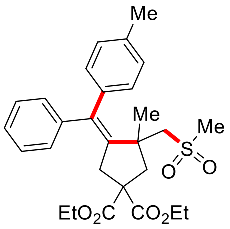

10

The product **10** was purified with silica gel chromatography (PE/EA = 3:1) as a white solid (0.2 mmol scaled, 45.8 mg, 46%); m.p. 108.9-110.0 °C. ^{1}H NMR (400 MHz, $CDCl_3$) δ 7.33 – 7.28 (m, 2H), 7.22 (t, *J* = 6.7 Hz, 3H), 7.09 (s, 4H), 4.22 – 4.14 (m, 4H), 3.19 – 3.10 (m, 3H), 3.02 – 2.96 (m, 2H), 2.71 (s, 3H), 2.50 (d, *J* = 14.3 Hz, 1H), 2.29 (s, 3H), 1.36 (s, 3H), 1.27 – 1.22 (m, 6H). ^{13}C NMR (101 MHz, $CDCl_3$) δ 171.9, 171.6, 143.5, 141.2, 140.8, 138.1, 136.3, 129.3, 129.1, 128.3, 127.6, 127.2, 63.3, 61.8, 61.7, 57.6, 46.2, 44.5, 44.1, 40.4, 28.3, 21.1, 14.1, 14.0. HRMS ESI (m/z): $[M+Na]^+$ calcd. for $C_{28}H_{34}O_6SNa$: 521.1968, found: 521.1975.

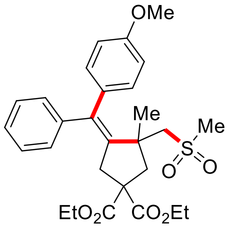

11

The product **11** was purified with silica gel chromatography (PE/EA = 3:1) as a white solid (0.2 mmol scaled, 63.7 mg, 62%); m.p. 100.3-102.2 °C. ^{1}H NMR (400 MHz, $CDCl_3$) δ 7.31 (t, *J* = 7.3 Hz, 2H), 7.25 – 7.19 (m, 3H), 7.13 (d, *J* = 8.6 Hz, 2H), 6.82 (d, *J* = 8.7 Hz, 2H), 4.22-4.16 (m, 4H), 3.76 (s, 3H), 3.19 – 3.11 (m, 3H), 3.04 – 2.96 (m, 2H), 2.71 (s, 3H), 2.50 (d, *J* = 14.2 Hz, 1H), 1.35 (s, 3H), 1.27 – 1.23 (m, 6H). ^{13}C NMR (101 MHz, $CDCl_3$) δ 171.9, 171.7, 158.2, 143.4, 141.4, 137.7, 136.2, 129.1, 129.0, 128.3, 127.2, 113.9, 63.3, 61.9, 61.7, 57.6, 55.2, 46.2, 44.5, 44.1, 40.5, 28.4, 14.1, 14.0. HRMS ESI (m/z): $[M+Na]^+$ calcd. for $C_{28}H_{34}O_7SNa$: 537.1917, found: 537.1920.

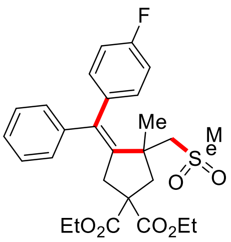

12

The product **12** was purified with silica gel chromatography (PE/EA = 2:1) as a white solid (0.2 mmol scaled, 68.2 mg, 68%); m.p. 98.2-100.7 °C. ^{1}H NMR (800 MHz, $CDCl_3$) δ 7.32 (t, *J* = 7.6 Hz, 2H), 7.25 (s, 1H), 7.23 – 7.21 (m, 2H), 7.20 – 7.18 (m, 2H), 6.97 (t, *J* = 8.7 Hz, 2H), 4.22 – 4.16 (m, 4H), 3.19 (d, *J* = 14.4 Hz, 1H), 3.14 (d, *J* = 14.0 Hz, 1H), 3.08 (dd, *J* = 17.1, 1.2 Hz, 1H), 2.99 – 2.96 (m, 2H), 2.72 (s, 3H), 2.50 (dd, *J* = 14.4, 1.2 Hz, 1H), 1.34 (s, 3H), 1.26 – 1.23 (m, 6H). ^{13}C NMR (201 MHz, $CDCl_3$) δ 171.7, 171.6, 161.5 (d, *J* = 245.9 Hz), 144.0, 140.9, 139.6 (d, *J* = 3.4 Hz), 137.0, 129.4 (d, *J* = 7.9 Hz), 129.1, 128.4, 127.4, 115.5 (d, *J* = 21.3 Hz), 63.1, 61.9, 61.7, 46.1, 44.5, 44.0, 40.5, 28.5, 14.0 (2C). ^{19}F NMR (376 MHz, $CDCl_3$) δ -115.7 (s, 1F). HRMS ESI (m/z): $[M+Na]^+$ calcd. for $C_{27}H_{31}FO_6SNa$: 525.1718, found: 525.1720.

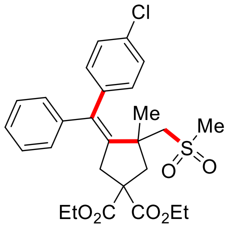

13

The product **13** was purified with silica gel chromatography (PE/EA = 3:1) as a white solid (0.2 mmol scaled, 61.1 mg, 59%); m.p. 110.5-112.2 °C. ^{1}H NMR (500 MHz, C DCl3) δ 7.32 (t, *J* = 7.3 Hz, 2H), 7.25 (d, *J* = 6.0 Hz, 3H), 7.22 (d, *J* = 8.0 Hz, 2H), 7.1 7 (d, *J* = 8.3 Hz, 2H), 4.22-4.16 (m, 4H), 3.20 (d, *J* = 14.3 Hz, 1H), 3.14 (d, *J* = 14.0 Hz, 1H), 3.08 (d, *J* = 17.0 Hz, 1H), 3.00 – 2.95 (m, 2H), 2.72 (s, 3H), 2.50 (d, *J* = 14.3 Hz, 1H), 1.33 (s, 3H), 1.27-1.24 (m, 6H). ^{13}C NMR (126 MHz, $CDCl_3$) δ 171.7, 171. 6, 144.2, 142.1, 140.6, 136.9, 132.6, 129.2, 129.1, 128.8, 128.5, 127.5, 63.1, 61.9, 61. 7, 57.6, 46.1, 44.6, 44.1, 40.5, 28.5, 14.1, 14.0. HRMS ESI (m/z): $[M+Na]^+$ calcd. for $C_{27}H_{31}ClO_6SNa$: 541.1422, found: 541.1423.

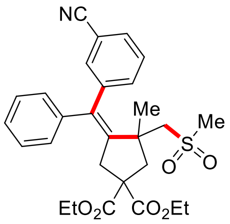

14

The product **14** was purified with silica gel chromatography (PE/EA = 2:1) as a white solid (0.2 mmol scaled, 65.2 mg, 64%); m.p. 101.2-103.5 °C. ^{1}H NMR (400 MHz, $CDCl_3$) δ 7.54 – 7.51 (m, 2H), 7.50 – 7.47 (m, 1H), 7.44 – 7.39 (m, 1H), 7.35 (dd, *J* =

7.9, 6.5 Hz, 2H), 7.31 – 7.28 (m, 1H), 7.26 – 7.23 (m, 2H), 4.25 – 4.16 (m, 4H), 3.24 (d, *J* = 14.3 Hz, 1H), 3.13 (d, *J* = 14.0 Hz, 1H), 3.04 (dd, *J* = 16.8, 1.0 Hz, 1H), 3.00 – 2.94 (m, 2H), 2.75 (s, 3H), 2.49 (dd, *J* = 14.3, 1.0 Hz, 1H), 1.32 (s, 3H), 1.28 – 1.24 (m, 6H). ^{13}C NMR (101 MHz, $CDCl_3$) δ 171.5, 171.4, 145.2, 144.8, 139.9, 135.9, 132.5, 131.5, 130.5, 129.6, 129.2, 128.7, 127.9, 118.7, 112.7, 62.8, 62.0, 61.9, 57.6, 46.0, 44.6, 44.1, 40.6, 28.7, 14.1, 14.0. HRMS ESI (m/z): $[M+Na]^+$ calcd. for $C_{28}H_{31}NO_6SNa$: 532.1764, found: 532.1761.

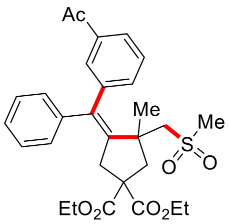

15

The product **15** was purified with silica gel chromatography (PE/EA = 2:1) as a white solid (0.2 mmol scaled, 59.9 mg, 57%); m.p. 100.8-102.6 °C. ^{1}H NMR (400 MHz, $CDCl_3$) δ 7.83 – 7.77 (m, 2H), 7.46 (d, *J* = 7.7 Hz, 1H), 7.41 (d, *J* = 7.6 Hz, 1H), 7.36 – 7.31 (m, 2H), 7.26 (d, *J* = 3.8 Hz, 3H), 4.23-4.16 (m, 4H), 3.19 (dd, *J* = 14.1, 10.0 Hz, 2H), 3.06 (d, *J* = 16.9 Hz, 1H), 3.02 – 2.93 (m, 2H), 2.74 (s, 3H), 2.58 (s, 3H), 2.52 (d, *J* = 14.2 Hz, 1H), 1.37 (s, 3H), 1.27 – 1.22 (m, 6H). ^{13}C NMR (101 MHz, $CDCl_3$) δ 198.0, 171.7, 171.5, 144.5, 144.2, 140.4, 137.4, 137.1, 132.6, 129.1, 129.0, 128.5, 127.6, 127.4, 126.9, 63.0, 61.9, 61.7, 57.6, 46.1, 44.6, 44.0, 40.4, 28.4, 26.7, 14.0 (2C). HRMS ESI (m/z): $[M+Na]^+$ calcd. for $C_{29}H_{34}O_7SNa$: 549.1917, found: 549.1933.

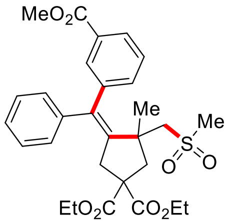

16

The product **16** was purified with silica gel chromatography (PE/EA = 2:1) as a white solid (0.2 mmol scaled, 62.8 mg, 58%); m.p. 103.2-105.9 °C. ^{1}H NMR (400 MHz, $CDCl_3$) δ 7.89 – 7.85 (m, 2H), 7.46 – 7.43 (m, 1H), 7.38 (t, *J* = 7.5 Hz, 1H), 7.35 – 7.30 (m, 2H), 7.26 (d, *J* = 5.2 Hz, 3H), 4.23 – 4.16 (m, 4H), 3.89 (s, 3H), 3.19 (dd, *J* = 14.2, 5.8 Hz, 2H), 3.06 (d, *J* = 17.1 Hz, 1H), 2.99 (d, *J* = 14.0 Hz, 1H), 2.94 (d, *J* = 17.0 Hz, 1H), 2.73 (s, 3H), 2.50 (d, *J* = 14.2 Hz, 1H), 1.38 (s, 3H), 1.27-1.23 (m, 6H). ^{13}C NMR (101 MHz, $CDCl_3$) δ 171.7, 171.5, 166.9, 144.6, 144.0, 140.5, 137.2, 132.4, 130.6, 129.1, 128.9, 128.8, 128.5, 128.0, 127.6, 63.1, 61.9, 61.7, 57.6, 52.2, 46.1, 44.6, 44.1, 40.4, 28.4, 14.0 (2C). HRMS ESI (m/z): $[M+Na]^+$ calcd. for $C_{29}H_{34}O_8SNa$: 565.1867, found: 565.1879.

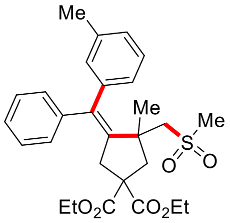

17

The product **17** was purified with silica gel chromatography (PE/EA = 4:1) as a white solid (0.2 mmol scaled, 69.7 mg, 70%); m.p. 120.7-122.2 °C. ^{1}H NMR (800 MHz, $CDCl_3$) δ 7.31 (t, *J* = 7.5 Hz, 2H), 7.23 (d, *J* = 7.6 Hz, 3H), 7.17 (d, *J* = 7.2 Hz, 1H), 7.00 (q, *J* = 7.3, 6.9 Hz, 3H), 4.22 – 4.15 (m, 4H), 3.17 (dd, *J* = 14.3, 3.2 Hz, 2H), 3.10 (d, *J* = 17.0 Hz, 1H), 2.99 – 2.96 (m, 2H), 2.70 (s, 3H), 2.49 (d, *J* = 14.4 Hz, 1H), 2.30 (s, 3H), 1.37 (s, 3H), 1.26-1.22 (m, 6H). ^{13}C NMR (201 MHz, $CDCl_3$) δ 171.9, 171.6, 143.7, 143.5, 141.1, 138.3, 138.2, 129.1, 128.5, 128.3, 128.2, 127.5, 127.2, 124.6, 63.3, 61.8, 61.6, 57.6, 46.2, 44.5, 44.1, 40.3, 28.3, 21.5, 14.0 (2C). HRMS ESI (m/z): $[M+Na]^+$ calcd. for $C_{28}H_{34}O_6SNa$: 521.1968, found: 521.1974.

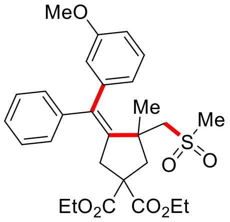

18

The product **18** was purified with silica gel chromatography (PE/EA = 4:1) as a white solid (0.2 mmol scaled, 67.8 mg, 66%); m.p. 130.9-132.8 °C. ^{1}H NMR (400 MHz, $CDCl_3$) δ 7.34 – 7.29 (m, 2H), 7.26 – 7.18 (m, 4H), 6.80 (d, *J* = 7.7 Hz, 1H), 6.76 – 6.70 (m, 2H), 4.23 – 4.15 (m, 4H), 3.77 (s, 3H), 3.20 – 3.10 (m, 3H), 3.02 – 2.95 (m, 2H), 2.71 (s, 3H), 2.50 (d, *J* = 14.3 Hz, 1H), 1.36 (s, 3H), 1.27-1.22 (m, 6H). ^{13}C NMR (101 MHz, $CDCl_3$) δ 171.8, 171.59, 159.6, 145.0, 143.7, 140.8, 137.9, 129.7, 129.0, 128.3, 127.3, 120.0, 113.6, 111.7, 63.2, 61.9, 61.7, 57.6, 55.2, 46.1, 44.5, 44.0, 40.3, 28.3, 14.0 (2C). HRMS ESI (m/z): $[M+Na]^+$ calcd. for $C_{28}H_{34}O_7SNa$: 537.1917, found: 537.1922.

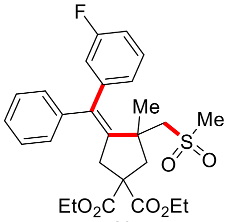

19

The product **19** was purified with silica gel chromatography (PE/EA = 3:1) as a white solid (0.2 mmol scaled, 77.3 mg, 77%); m.p. 102.5-104.2 °C. ^{1}H NMR (500 MHz, $CDCl_3$) δ 7.35 – 7.30 (m, 2H), 7.24 (dd, *J* = 10.8, 4.3 Hz, 4H), 7.01 (d, *J* = 7.5 Hz, 1H), 6.89 (dd, *J* = 18.1, 9.2 Hz, 2H), 4.23-4.15 (m, 4H), 3.20 (d, *J* = 14.3 Hz, 1H), 3.15 (d, *J* = 14.1 Hz, 1H), 3.10 (d, *J* = 17.1 Hz, 1H), 3.01 – 2.94 (m, 2H), 2.72 (d, *J* = 5.1 Hz, 3H), 2.49 (d, *J* = 14.3 Hz, 1H), 1.34 (d, *J* = 5.2 Hz, 3H), 1.27 – 1.24 (m, 6H).

^{13}C NMR (126 MHz, $CDCl_3$) δ 171.7, 171.5, 162.8 (d, *J* = 246.9 Hz), 145.7 (d, *J* = 6.9 Hz), 144.3, 140.4, 136.9, 130.2 (d, *J* = 8.8 Hz), 129.1, 128.5, 127.6, 123.5 (d, *J* = 2.8 Hz), 114.8 (d, *J* = 21.3 Hz), 113.7 (d, *J* = 21.1 Hz), 63.1, 61.9, 61.7, 57.6, 46.1, 44.6, 44.1, 40.4, 28.5, 14.0 (2C). ^{19}F NMR (753 MHz, $CDCl_3$) δ -112.7 (m, J = 9.3, 6.5 Hz, 1F). HRMS ESI (m/z): $[M+Na]^+$ calcd. for $C_{27}H_{31}FO_6SNa$: 525.1718, found: 525.1724.

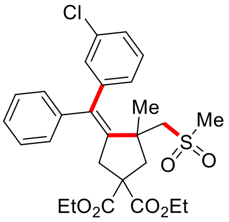

20

The product **20** was purified with silica gel chromatography (PE/EA = 3:1) as a white solid (0.2 mmol scaled, 94.2 mg, 91%); m.p. 110.0-112.9 °C. ^{1}H NMR (500 MHz, $CDCl_3$) δ 7.33 (t, *J* = 7.4 Hz, 2H), 7.28 (s, 1H), 7.25-7.21 (m, 3H), 7.19 – 7.15 (m, 2H), 7.12 (dd, *J* = 7.5, 1.3 Hz, 1H), 4.24 – 4.17 (m, 4H), 3.20 (d, *J* = 14.3 Hz, 1H), 3.15 (d, *J* = 14.0 Hz, 1H), 3.09 (d, *J* = 17.0 Hz, 1H), 2.99 – 2.94 (m, 2H), 2.72 (s, 3H), 2.49 (d, *J* = 14.3 Hz, 1H), 1.35 (s, 3H), 1.27-1.24 (m, 6H). ^{13}C NMR (126 MHz, $CDCl_3$) δ 171.7, 171.5, 145.3, 144.5, 140.3, 136.8, 134.3, 130.0, 129.1, 128.5, 127.8, 127.6, 126.9, 126.0, 63.0, 61.9, 61.7, 57.6, 46.0, 44.6, 44.1, 40.4, 28.5, 14.0 (2C). HRMS ESI (m/z): $[M+Na]^+$ calcd. for $C_{27}H_{31}ClO_6SNa$: 541.1422, found: 541.1429.

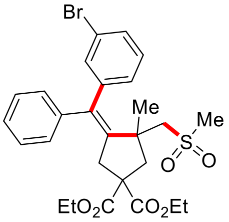

21

The product **21** was purified with silica gel chromatography (PE/EA = 3:1) as a white solid (0.2 mmol scaled, 71.9mg, 64%); m.p. 100.8-102.0 °C. ^{1}H NMR (400 MHz, $CDCl_3$) δ 7.36 – 7.30 (m, 4H), 7.28 (s, 1H), 7.23 (d, *J* = 7.0 Hz, 2H), 7.17 (d, *J* = 4.9 Hz, 2H), 4.23 – 4.16 (m, 4H), 3.20 (d, *J* = 14.3 Hz, 1H), 3.15 (d, *J* = 14.1 Hz, 1H), 3.08 (d, *J* = 16.9 Hz, 1H), 2.98 (d, *J* = 5.2 Hz, 1H), 2.94 (s, 1H), 2.72 (s, 3H), 2.48 (d, *J* = 14.4 Hz, 1H), 1.34 (d, *J* = 4.8 Hz, 3H), 1.28-1.23 (m, 6H). ^{13}C NMR (101 MHz, $CDCl_3$) δ 171.7, 171.5, 145.7, 144.6, 140.3, 136.7, 130.6, 130.3, 129.8, 129.1, 128.5, 127.6, 126.5, 122.6, 63.0, 61.9, 61.8, 57.6, 46.1, 44.6, 44.1, 40.4, 28.5, 14.1, 14.0. HRMS ESI (m/z): $[M+Na]^+$ calcd. for $C_{27}H_{31}BrO_6SNa$: 585.0917, found: 585.0924.

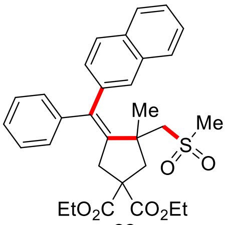

22

The product **22** was purified with silica gel chromatography (PE/EA = 3:1) as a white solid (0.2 mmol scaled, 70.4 mg, 66%); m.p. 108.2-110.6 °C. ^{1}H NMR (800 MHz, $CDCl_3$) δ 7.79 – 7.76 (m, 3H), 7.67 (s, 1H), 7.46 – 7.42 (m, 2H), 7.35 (dd, *J* = 8.4, 1.4 Hz, 1H), 7.32 (d, *J* = 6.6 Hz, 4H), 7.25 – 7.23 (m, 1H), 4.20 – 4.14 (m, 4H), 3.23 – 3.20 (m, 2H), 3.14 (d, *J* = 17.2 Hz, 1H), 3.04 (d, *J* = 16.1 Hz, 2H), 2.74 (s, 3H), 2.53 (d, *J* = 14.4 Hz, 1H), 1.41 (s, 3H), 1.26-1.20 (m, 6H). ^{13}C NMR (201 MHz, $CDCl_3$) δ 171.8, 171.6, 144.1, 141.2, 140.9, 138.0, 133.5, 132.2, 129.3, 128.4, 128.3, 128.0, 127.6, 127.4, 126.3, 126.2, 126.1, 125.9, 63.3, 61.8, 61.7, 57.6, 46.2, 44.7, 44.1, 40.5, 28.5, 14.0 (2C). HRMS ESI (m/z): $[M+Na]^+$ calcd. for $C_{31}H_{34}O_6SNa$: 557.1968, found: 557.1967.

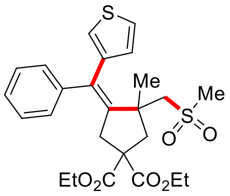

23

The product **23** was purified with silica gel chromatography (PE/EA = 4:1) as a white solid (0.2 mmol scaled, 54.8mg, 56%); m.p. 124.3-126.0 °C. ^{1}H NMR (800 MHz, $CDCl_3$) δ 7.34 (t, *J* = 7.6 Hz, 2H), 7.28 (d, *J* = 7.4 Hz, 1H), 7.22 – 7.19 (m, 3H), 7.04 – 7.02 (m, 1H), 6.92 – 6.90 (m, 1H), 4.23 – 4.18 (m, 4H), 3.33 (d, *J* = 17.1 Hz, 1H), 3.18 (d, *J* = 17.1 Hz, 1H), 3.16 (d, *J* = 14.4 Hz, 1H), 3.13 (d, *J* = 14.1 Hz, 1H), 2.94 (d, *J* = 14.1 Hz, 1H), 2.69 (s, 3H), 2.53 (d, *J* = 14.3 Hz, 1H), 1.32 (s, 3H), 1.27 – 1.25 (m, 6H). ^{13}C NMR (201 MHz, $CDCl_3$) δ 171.9, 171.6, 144.0, 143.5, 140.8, 132.8, 129.5, 128.3, 127.9, 127.4, 125.0, 122.7, 63.3, 61.9, 61.7, 57.8, 46.1, 45.0, 44.1, 40.8, 28.2, 14.0 (2C). HRMS ESI (m/z): $[M+Na]^+$ calcd. for $C_{25}H_{30}O_6S_2Na$: 513.1376, found: 513.1382.

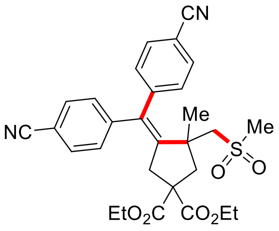

24

The product **24** was purified with silica gel chromatography (PE/EA = 2:1) as a white solid (0.2 mmol scaled, 79.0 mg, 74%); m.p. 110.2-112.6 °C. ^{1}H NMR (400 MHz, $CDCl_3$) δ 7.64 (dd, *J* = 15.0, 8.5 Hz, 4H), 7.45 (d, *J* = 8.1 Hz, 2H), 7.37 (d, *J* = 8.1 Hz, 2H), 4.24-4.15 (m, 4H), 3.30 (d, *J* = 14.3 Hz, 1H), 3.08 (d, *J* = 13.9 Hz, 1H), 3.04 – 2.97 (m, 3H), 2.86 (s, 3H), 2.44 (d, *J* = 14.2 Hz, 1H), 1.28 – 1.20 (m, 9H). ^{13}C NMR (101 MHz, $CDCl_3$) δ 171.5, 171.0, 147.2, 145.5, 144.8, 134.7, 132.8, 132.4, 130.2, 128.8, 118.5, 118.2, 111.8, 111.2, 62.7, 62.1, 61.9, 57.3, 46.0, 44.6, 44.3, 41.3, 29.2, 14.0 (2C). HRMS ESI (m/z): $[M+Na]^+$ calcd. for $C_{29}H_{30}N_2O_6SNa$: 557.1717, found: 557.1721.

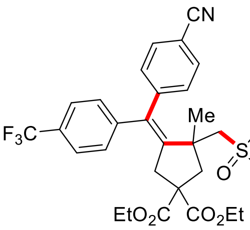

25

The product **25** was purified with silica gel chromatography (PE/EA = 2:1) as a white solid (0.2 mmol scaled, 80.7 mg, 70%); m.p. 118.2-120.0 °C. ^{1}H NMR (500 MHz, $CDCl_3$) δ 7.61 (d, *J* = 8.0 Hz, 4H), 7.43 (d, *J* = 8.0 Hz, 2H), 7.38 (d, *J* = 8.3 Hz, 2H), 4.23 – 4.16 (m, 4H), 3.31 (d, *J* = 14.3 Hz, 1H), 3.10 (d, *J* = 13.9 Hz, 1H), 3.04 – 2.95 (m, 3H), 2.82 (s, 3H), 2.46 (d, *J* = 14.3 Hz, 1H), 1.30 – 1.21 (m, 9H). ^{13}C NMR (126 MHz, $CDCl_3$) δ 171.5, 171.1, 147.5, 145.3, 143.6, 135.0, 132.7, 130.0 (q, *J* = 32.6 Hz), 129.7, 128.7, 125.6 (q, *J* = 3.6 Hz), 123.8 (q, *J* = 272.2 Hz), 118.6, 111.0, 62.7, 62.0, 61.8, 57.4, 45.9, 44.6, 44.2, 41.2, 29.1, 14.0 (2C). ^{19}F NMR (376 MHz, $CDCl_3$) δ -62.6 (s, 3F). HRMS ESI (m/z): $[M+Na]^+$ calcd. for $C_{29}H_{30}F_3NO_6SNa$: 600.1638, found: 600.1649.

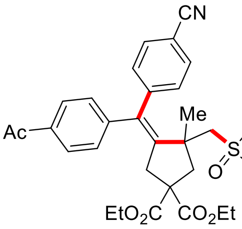

26

The product **26** was purified with silica gel chromatography (PE/EA = 2:1) as a white solid (0.2 mmol scaled, 79.3 mg, 72%); m.p. 118.3-120.8 °C. ^{1}H NMR (400 MHz, $CDCl_3$) δ 7.94 (dd, *J* = 8.3, 1.6 Hz, 2H), 7.60 (dd, *J* = 8.1, 1.5 Hz, 2H), 7.42 – 7.36 (m, 4H), 4.25 – 4.15 (m, 4H), 3.29 (d, *J* = 14.3 Hz, 1H), 3.13 (d, *J* = 13.9 Hz, 1H), 3.03 – 2.98 (m, 3H), 2.82 (s, 3H), 2.59 (d, *J* = 1.9 Hz, 3H), 2.47 (d, *J* = 14.3 Hz, 1H), 1.28 – 1.22 (m, 9H). ^{13}C NMR (101 MHz, $CDCl_3$) δ 197.3, 171.5, 171.2, 147.6, 145.2, 144.8, 136.3, 135.4, 132.7, 129.6, 128.8, 128.6, 118.6, 110.9, 62.8, 62.0, 61.8, 57.5, 45.9, 44.7, 44.2, 41.1, 29.0, 26.6, 14.0 (2C). HRMS ESI (m/z): $[M+Na]^+$ calcd. for $C_{30}H_{33}NO_7SNa$: 574.1870, found: 574.1877.

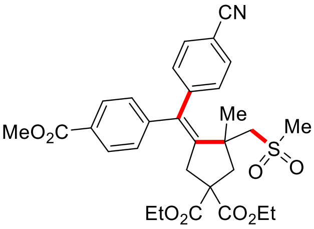

27

The product **27** was purified with silica gel chromatography (PE/EA = 2:1) as a white solid (0.2 mmol scaled, 95.2mg, 84%); m.p. 120.9-122.8 °C. ^{1}H NMR (500 MHz, $CDCl_3$) δ 8.02 (d, *J* = 8.3 Hz, 2H), 7.61 (d, *J* = 8.3 Hz, 2H), 7.38 (t, *J* = 7.7 Hz, 4H), 4.23 – 4.16 (m, 4H), 3.91 (s, 3H), 3.29 (d, *J* = 14.3 Hz, 1H), 3.11 (d, *J* = 13.9 Hz, 1H),

3.03 – 2.96 (m, 3H), 2.80 (s, 3H), 2.47 (d, *J* = 14.3 Hz, 1H), 1.27 – 1.23 (m, 9H). ^{13}C NMR (126 MHz, $CDCl_3$) δ 171.5, 171.2, 166.4, 147.6, 145.2, 144.6, 135.5, 132.7, 129.9, 129.6, 129.4, 128.8, 118.7, 110.9, 62.7, 62.0, 61.9, 57.5, 52.3, 45.9, 44.7, 44.2, 41.0, 29.0, 14.1, 14.0. HRMS ESI (m/z): $[M+Na]^+$ calcd. for $C_{30}H_{33}NO_8SNa$: 590.1819, found: 590.1818.

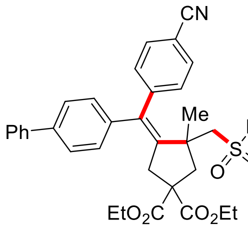

28

The product **28** was purified with silica gel chromatography (PE/EA = 2:1) as a white solid (0.2 mmol scaled, 106.4 mg, 91%); m.p. 110.8-112.2 °C. ^{1}H NMR (400 MHz, $CDCl_3$) δ 7.63 – 7.55 (m, 6H), 7.43 (q, *J* = 8.1, 7.7 Hz, 4H), 7.35 (dd, *J* = 19.0, 7.7 Hz, 3H), 4.23-4.17 (m, 4H), 3.27 (d, *J* = 14.3 Hz, 1H), 3.19 (d, *J* = 13.9 Hz, 1H), 3.05 (d, *J* = 14.0 Hz, 2H), 3.02 (d, 13.9 Hz, 1H), 2.77 (s, 3H), 2.51 (d, *J* = 14.2 Hz, 1H), 1.35 (s, 3H), 1.27 – 1.23 (m, 6H). ^{13}C NMR (201 MHz, $CDCl_3$) δ 171.5, 171.4, 148.3, 145.1, 140.7, 140.0, 138.7, 136.0, 142.6, 129.7, 128.9, 128.8, 127.7, 127.2, 127.0, 118.8, 110.6, 62.9, 62.0, 61.8, 57.6, 46.0, 44.7, 44.1, 40.8, 28.9, 14.1, 14.0. HRMS ESI (m/z): $[M+Na]^+$ calcd. for $C_{34}H_{35}NO_6SNa$: 608.2077, found: 608.2086.

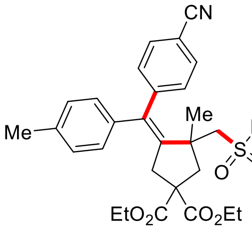

29

The product **29** was purified with silica gel chromatography (PE/EA = 2:1) as a white solid (0.2 mmol scaled, 89.9 mg, 86%); m.p. 115.2-117.5 °C. ^{1}H NMR (800 MHz, C DCl$_3$) δ 7.59 – 7.57 (m, 2H), 7.36 – 7.34 (m, 2H), 7.14 (d, *J* = 8.0 Hz, 2H), 7.11 (d, *J* = 8.1 Hz, 2H), 4.23 – 4.14 (m, 4H), 3.23 (d, *J* = 14.4 Hz, 1H), 3.15 (d, *J* = 14.0 Hz, 1 H), 3.03 – 3.01 (m, 2H), 2.96 (d, *J* = 17.0 Hz, 1H), 2.76 (s, 3H), 2.49 (dd, *J* = 14.4, 1. 3 Hz, 1H), 2.32 (s, 3H), 1.32 (s, 3H), 1.26-1.22 (m, 6H). ^{13}C NMR (201 MHz, $CDCl_3$) δ 171.5, 171.4, 148.6, 144.8, 137.6, 136.7, 136.4, 132.5, 129.3, 129.1, 128.7, 118.8, 1 10.4, 62.9, 62.0, 61.8, 57.6, 46.0, 44.7, 44.1, 40.7, 28.7, 21.2, 14.0 (2C). HRMS ESI (m/z): $[M+Na]^+$ calcd. for $C_{29}H_{33}NO_6SNa$: 546.1921, found: 546.1934.

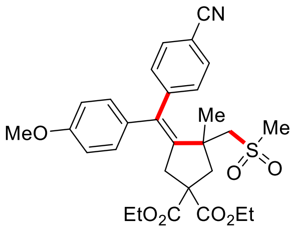

30

The product **30** was purified with silica gel chromatography (PE/EA = 2:1) as a white solid (0.2 mmol scaled, 63.6 mg, 59%); m.p. 120.0-122.2 °C. ^{1}H NMR (400 MHz, $CDCl_3$) δ 7.60 – 7.56 (m, 2H), 7.36 – 7.33 (m, 2H), 7.16 – 7.12 (m, 2H), 6.88 – 6.84 (m, 2H), 4.23 – 4.15 (m, 4H), 3.79 (s, 3H), 3.24 (d, *J* = 14.2 Hz, 1H), 3.14 (d, *J* = 14.0 Hz, 1H), 3.05 – 2.97 (m, 3H), 2.78 (s, 3H), 2.49 (dd, *J* = 14.2, 1.1 Hz, 1H), 1.32 (s, 3H), 1.26 – 1.23 (m, 6H). ^{13}C NMR (101 MHz, $CDCl_3$) δ 171.5, 171.4, 159.0, 148.7, 145.1, 136.1, 132.5, 131.8, 130.4 128.7, 118.8, 114.0, 110.4, 62.9, 62.0, 61.8, 57.6, 55.3, 46.0, 44.7, 44.2, 40.7, 28.8, 14.0 (2C). HRMS ESI (m/z): $[M+Na]^+$ calcd. for $C_{29}H_{33}NO_7SNa$: 562.1870, found: 562.1864.

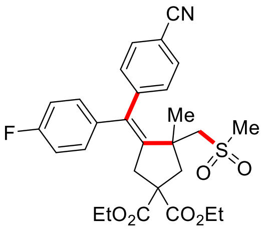

31

The product **31** was purified with silica gel chromatography (PE/EA = 2:1) as a white solid (0.2 mmol scaled, 87.4 mg, 83%); m.p. 108.2-110.6 °C. ^{1}H NMR (400 MHz, $CDCl_3$) δ 7.62 – 7.58 (m, 2H), 7.38 – 7.35 (m, 2H), 7.27 – 7.23 (m, 2H), 7.07 – 7.02 (m, 2H), 4.23-4.16 (m, 4H), 3.27 (d, *J* = 14.3 Hz, 1H), 3.15 – 3.10 (m, 1H), 3.00 (t, *J* = 7.0 Hz, 3H), 2.81 (s, 3H), 2.48 (d, *J* = 14.3 Hz, 1H), 1.28 (d, *J* = 2.0 Hz, 3H), 1.26 – 1.21 (m, 6H). ^{13}C NMR (101 MHz, $CDCl_3$) δ 171.5, 171.3, 162.09 (d, *J* = 248.3 Hz), 148.2, 145.4, 135.6 (d, *J* = 3.6 Hz), 135.4, 132.6, 131.0 (d, *J* = 7.9 Hz), 128.7, 118.7, 115.7 (d, *J* = 21.4 Hz), 110.7, 62.8, 62.0, 61.8, 57.5, 46.0, 44.7, 44.2, 41.0, 28.9, 14.0 (2C). ^{19}F NMR (376 MHz, CDCl3) δ -113.4 (s, 1F). HRMS ESI (m/z): $[M+Na]^+$ calcd. for $C_{28}H_{30}FNO_6SNa$: 550.1670, found: 550.1672.

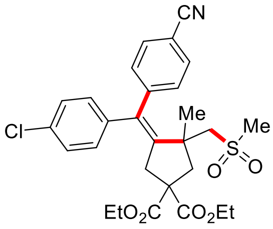

32

The product **32** was purified with silica gel chromatography (PE/EA = 2:1) as a white solid (0.2 mmol scaled, 98.8 mg, 91%); m.p. 120.9-122.8 °C. ^{1}H NMR (400 MHz, C DCl$_3$) δ 7.60 (d, *J* = 8.0 Hz, 2H), 7.34 (dd, *J* = 10.3, 8.2 Hz, 4H), 7.21 (d, *J* = 8.2 Hz, 2H), 4.23 – 4.12 (m, 4H), 3.28 (d, *J* = 14.3 Hz, 1H), 3.12 (d, *J* = 13.9 Hz, 1H), 3.02 – 2.98 (m, 3H), 2.82 (s, 3H), 2.47 (d, *J* = 14.3 Hz, 1H), 1.27-1.24 (m, 9H). ^{13}C NMR (1 01 MHz, $CDCl_3$) δ 171.5, 171.3, 147.9, 145.3, 138.2, 135.2, 133.9, 132.6, 130.6, 128. 9, 128.7, 118.7, 110.8, 62.7, 62.0, 61.9, 57.5, 45.9, 44.7, 44.2, 41.0, 29.1, 14.1, 14.0. HRMS ESI (m/z): $[M+Na]^+$ calcd. for $C_{28}H_{30}ClNO_6SNa$: 566.1375, found: 566.1375.

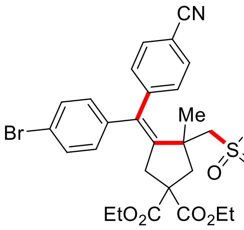

33

The product **33** was purified with silica gel chromatography (PE/EA = 2:1) as a white solid (0.2 mmol scaled, 113.8mg, 95%); m.p. 130.9-132.6 °C. ^{1}H NMR (400 MHz, $CDCl_3$) δ 7.60 (d, *J* = 8.4 Hz, 2H), 7.48 (d, *J* = 8.4 Hz, 2H), 7.35 (d, *J* = 8.3 Hz, 2H), 7.16 (d, *J* = 8.4 Hz, 2H), 4.24 – 4.14 (m, 4H), 3.29 (d, *J* = 14.3 Hz, 1H), 3.13 (d, *J* = 14.0 Hz, 1H), 3.03 – 2.99 (m, 3H), 2.83 (s, 3H), 2.47 (d, *J* = 14.3 Hz, 1H), 1.28 – 1.22 (m, 9H).^{13}C NMR (101 MHz, $CDCl_3$) δ 171.5, 171.2, 147.8, 145.2, 138.6, 135.1, 132.6, 131.8, 130.9, 128.7, 122.0, 118.7, 110.7, 62.7, 62.0, 61.8, 57.4, 45.9, 44.6, 44.2, 41.0, 29.1, 14.0 (2C). HRMS ESI (m/z): $[M+Na]^+$ calcd. for $C_{28}H_{30}BrNO_6SNa$: 610.0869, found: 610.0873.

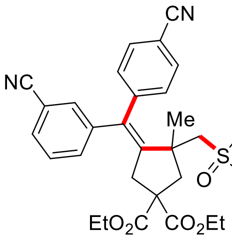

34

The product **34** was purified with silica gel chromatography (PE/EA = 2:1) as a white solid (0.2 mmol scaled, 90.7 mg, 85%); m.p. 138.8-140.1 °C. ^{1}H NMR (400 MHz, C DCl$_3$) δ 7.66 – 7.56 (m, 5H), 7.49 (d, *J* = 7.7 Hz, 1H), 7.38 (d, *J* = 8.3 Hz, 2H), 4.24-4. 16 (m, 4H), 3.32 (d, *J* = 14.2 Hz, 1H), 3.08 – 2.97 (m, 4H), 2.87 (s, 3H), 2.43 (d, *J* = 1 4.2 Hz, 1H), 1.28 – 1.22 (m, 9H). ^{13}C NMR (101 MHz, $CDCl_3$) δ 171.5, 171.0, 147.3, 145.8, 141.2, 134.2, 133.8, 132.8, 132.7, 131.4, 129.6, 128.8, 118.6, 118.1, 112.9, 11 1.2, 62.6, 62.1, 61.9, 57.3, 46.0, 44.6, 44.2, 41.4, 29.3, 14.1, 14.0. HRMS ESI (m/z): $[M+Na]^+$ calcd. for $C_{29}H_{30}N_2O_6SNa$: 557.1717, found: 557.1723.

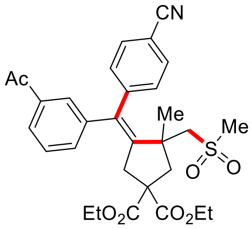

35

The product **35** was purified with silica gel chromatography (PE/EA = 2:1) as a white solid (0.2 mmol scaled, 103.5mg, 94%); m.p. 110.9-112.6 °C. ^{1}H NMR (400 MHz, $CDCl_3$) δ 7.90 (d, *J* = 23.8 Hz, 1H), 7.83 (d, *J* = 18.6 Hz, 1H), 7.63 (dd, *J* = 15.3, 8.0 Hz, 2H), 7.49 – 7.39 (m, 4H), 4.25 – 4.15 (m, 4H), 3.31 (d, *J* = 14.3 Hz, 1H), 3.14 (d, *J* = 13.9 Hz, 1H), 3.05 – 2.97 (m, 3H), 2.85 (s, 3H), 2.60 (s, 3H), 2.45 (d, *J* = 14.2 Hz, 1H), 1.27 – 1.19 (m, 9H). ^{13}C NMR (101 MHz, $CDCl_3$) δ 197.8, 171.5, 171.2, 147.8,

145.3, 140.4, 137.3, 135.3, 133.9, 132.7, 129.0, 128.8, 128.5, 127.9, 118.7, 110.8, 62.7, 62.0, 61.9, 57.5, 45.9, 44.6, 44.1, 41.2, 29.0, 26.8, 14.1, 14.0. HRMS ESI (m/z): $[M+H]^+$ calcd. for $C_{30}H_{33}NO_7S$: 552.2050, found: 552.2031.

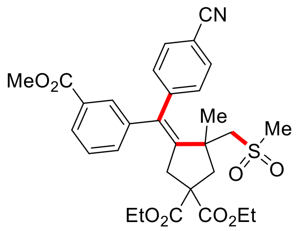

36

The product **36** was purified with silica gel chromatography (PE/EA = 2:1) as a white solid (0.2 mmol scaled, 99.7 mg, 88%); m.p. 106.8-108.3 °C. ^{1}H NMR (500 MHz, $CDCl_3$) δ 7.99 – 7.94 (m, 2H), 7.61 (d, *J* = 8.2 Hz, 2H), 7.48 – 7.39 (m, 4H), 4.23-4.16 (m, 4H), 3.92 (s, 3H), 3.32 (d, *J* = 14.3 Hz, 1H), 3.14 (d, *J* = 13.9 Hz, 1H), 3.04 – 2.96 (m, 3H), 2.82 (s, 3H), 2.47 (d, *J* = 14.3 Hz, 1H), 1.27 – 1.20 (m, 9H). ^{13}C NMR (126 MHz, $CDCl_3$) δ 171.5, 171.2, 166.5, 147.9, 145.5, 140.2, 135.2, 133.8, 132.6, 130.5, 130.1, 128.9, 128.8 (2C), 118.7, 110.8, 62.7, 62.0, 61.8, 57.5, 52.4, 45.9, 44.6, 44.0, 41.1, 29.0, 14.0 (2C). HRMS ESI (m/z): $[M+Na]^+$ calcd. for $C_{30}H_{33}NO_8SNa$: 590.1819, found: 590.1822.

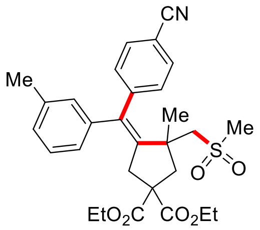

37

The product **37** was purified with silica gel chromatography (PE/EA = 2:1) as a white solid (0.2 mmol scaled, 94.1 mg, 90%); m.p. 118.0-120.8 °C. ^{1}H NMR (800 MHz, C DCl$_3$) δ 7.62 (d, *J* = 8.3 Hz, 2H), 7.40 (d, *J* = 8.3 Hz, 2H), 7.20 (d, *J* = 7.6 Hz, 1H), 7. 03 (d, *J* = 7.6 Hz, 1H), 6.99 (d, *J* = 7.7 Hz, 1H), 6.96 (s, 1H), 4.21 – 4.16 (m, 4H), 3.2 3 (d, *J* = 14.4 Hz, 1H), 3.16 (d, *J* = 13.9 Hz, 1H), 3.08 (d, *J* = 16.3 Hz, 1H), 3.04 (d, *J* = 13.9 Hz, 1H), 2.94 (d, *J* = 13.9 Hz, 1H), 2.81 (s, 3H), 2.47 (d, *J* = 14.4 Hz, 1H), 2.3 0 (s, 3H), 1.28 (s, 3H), 1.26-1.22 (m, 6H). ^{13}C NMR (201 MHz, $CDCl_3$) δ 171.6, 171. 5, 146.2, 144.4, 142.6, 138.6, 136.6, 132.2, 130.0, 128.8, 128.2, 128.0, 124.7, 118.5, 1 11.2, 63.1, 61.9, 61.7, 57.4, 46.1, 44.6, 44.2, 40.8, 28.7, 21.5, 14.0 (2C). HRMS ESI (m/z): $[M+Na]^+$ calcd. for $C_{29}H_{33}NO_6SNa$: 546.1921, found: 546.1926.

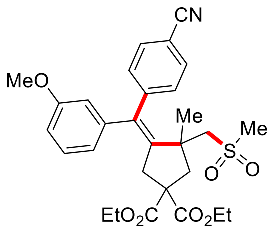

38

The product **38** was purified with silica gel chromatography (PE/EA = 2:1) as a white solid (0.2 mmol scaled, 83.0mg, 77%); m.p. 126.5-128.2 °C. ^{1}H NMR (400 MHz, $CDCl_3$) δ 7.59 (d, *J* = 8.3 Hz, 2H), 7.37 (d, *J* = 8.4 Hz, 2H), 7.24 (d, *J* = 8.0 Hz, 1H), 6.83 – 6.77 (m, 3H), 4.23 – 4.15 (m, 4H), 3.78 (s, 3H), 3.24 (d, *J* = 14.3 Hz, 1H), 3.17 (d, *J* = 14.0 Hz, 1H), 3.05 (d, *J* = 6.6 Hz, 1H), 3.01 (d, *J* = 8.9 Hz, 1H), 2.99 (d, *J* = 12.0 Hz, 1H), 2.78 (s, 3H), 2.49 (d, *J* = 14.3 Hz, 1H), 1.32 (s, 3H), 1.27-1.21 (m, 6H). ^{13}C NMR (201 MHz, $CDCl_3$) δ 171.5, 171.4, 159.6, 148.2, 144.7, 141.0, 136.1, 132.5, 129.7, 128.7, 121.5, 118.8, 115.5, 112.6, 110.6, 62.8, 62.0, 61.8, 57.6, 55.3, 46.0, 44.7, 44.1, 40.7, 28.8, 14.0 (2C). HRMS ESI (m/z): $[M+Na]^+$ calcd. for $C_{29}H_{33}NO_7SNa$: 562.1870, found: 562.1877.

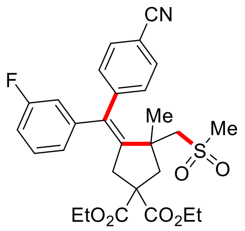

39

The product **39** was purified with silica gel chromatography (PE/EA = 2:1) as a white solid (0.2 mmol scaled, 87.4 mg, 83%); m.p. 120.5-122.2 °C. ^{1}H NMR (400 MHz, $CDCl_3$) δ 7.63 – 7.59 (m, 2H), 7.40 – 7.37 (m, 2H), 7.35 – 7.30 (m, 1H), 7.09 – 7.06 (m, 1H), 7.02 – 6.97 (m, 2H), 4.24 – 4.14 (m, 4H), 3.29 (d, *J* = 14.3 Hz, 1H), 3.15 (d, *J* = 13.9 Hz, 1H), 3.02 (d, *J* = 13.5 Hz, 3H), 2.82 (s, 3H), 2.48 (d, *J* = 14.2 Hz, 1H), 1.30 – 1.22 (m, 9H). ^{13}C NMR (101 MHz, $CDCl_3$) δ 171.4, 171.2, 163.5 (d, *J* = 248.7 Hz), 147.7, 145.3, 141.9 (d, *J* = 7.4 Hz), 135.0, 132.6, 130.3 (d, *J* = 8.4 Hz), 128.7, 125.0 (d, *J* = 2.9 Hz), 118.7, 116.3 (d, *J* = 21.2 Hz), 114.8 (d, *J* = 20.9 Hz), 110.8, 62.7, 62.0, 61.8, 57.5, 45.9, 44.7, 44.1, 40.9, 28.9, 14.0 (2C). ^{19}F NMR (376 MHz, $CDCl_3$) δ -111.3 (s, 1F). HRMS ESI (m/z): $[M+Na]^+$ calcd. for $C_{28}H_{30}FNO_6SNa$: 550.1670, found: 550.1674.

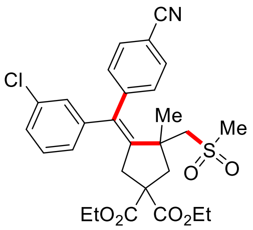

40

The product **40** was purified with silica gel chromatography (PE/EA = 2:1) as a white solid (0.2 mmol scaled, 92.3 mg, 85%); m.p. 138.3-140.2 °C. ^{1}H NMR (400 MHz, $CDCl_3$) δ 7.62 – 7.59 (m, 2H), 7.39 – 7.36 (m, 2H), 7.30 – 7.27 (m, 3H), 7.18-7.15 (m, 1H), 4.24 – 4.15 (m, 4H), 3.30 (d, *J* = 14.3 Hz, 1H), 3.12 (d, *J* = 13.9 Hz, 1H), 3.02 – 2.97 (m, 3H), 2.82 (s, 3H), 2.47 (d, *J* = 14.2 Hz, 1H), 1.28 – 1.24 (m, 9H). ^{13}C NMR (101 MHz, $CDCl_3$) δ 171.5, 171.2, 147.7, 145.4, 141.6, 134.9, 134.5, 132.7, 129.9, 129.3, 128.8, 128.0, 127.5, 118.7, 110.9, 62.6, 62.0, 61.8, 57.5, 45.9, 44.7, 44.1, 41.0,

29.1, 14.0 (2C). HRMS ESI (m/z): $[M+Na]^+$ calcd. for $C_{28}H_{30}ClNO_6SNa$: 566.1375, found: 566.1379.

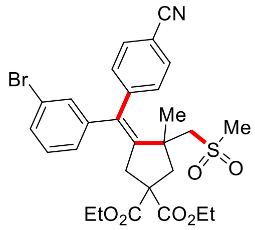

41

The product **41** was purified with silica gel chromatography (PE/EA = 2:1) as a white solid (0.2 mmol scaled, 102.1 mg, 87%); m.p. 118.5-120.2 °C. ^{1}H NMR (500 MHz, $CDCl_3$) δ 7.64 (d, *J* = 8.3 Hz, 2H), 7.42 (d, *J* = 8.3 Hz, 2H), 7.37 – 7.32 (m, 2H), 7.21 – 7.14 (m, 2H), 4.24-4.15 (m, 4H), 3.26 (d, *J* = 14.3 Hz, 1H), 3.12 (d, *J* = 13.9 Hz, 1H), 3.05 (d, *J* = 1.5 Hz, 2H), 2.95 (d, *J* = 13.8 Hz, 1H), 2.83 (s, 3H), 2.45 (d, *J* = 14.3 Hz, 1H), 1.28 – 1.22 (m, 9H). ^{13}C NMR (126 MHz, $CDCl_3$) δ 171.4, 171.4, 145.4, 145.3, 144.5, 135.0, 132.3, 130.6, 130.5, 130.4, 130.0, 126.5, 122.8, 118.3, 111.5, 62.8, 62.0, 61.8, 57.3, 46.0, 44.6, 44.2, 41.0, 28.9, 14.1, 14.0. HRMS ESI (m/z): $[M+Na]^+$ calcd. for $C_{28}H_{30}BrNO_6SNa$: 610.0869, found: 610.0878.

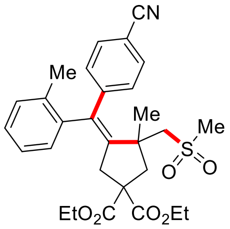

42

The product **42** was purified with silica gel chromatography (PE/EA = 2:1) as a white solid (0.2 mmol scaled, 87.8mg, 84%); m.p. 130.5-132.2 °C. ^{1}H NMR (800 MHz, $CDCl_3$) δ 7.59 – 7.57 (m, 2H), 7.43 (d, *J* = 8.4 Hz, 1H), 7.35 (d, *J* = 8.4 Hz, 1H), 7.31-7.29 (m, 0.5H), 7.28-7.27 (m, 0.5H), 7.25-7.24 (m, 1H), 7.2-7.21 (m, 1H), 7.18 – 7.15 (m, 1H), 4.26 – 4.15 (m, 4H), 3.31 – 3.26 (m, 1H), 3.25 (d, *J* = 13.9 Hz, 0.5H), 3.18 (d, *J* = 16.9 Hz, 0.5H), 3.13 (d, *J* = 13.5 Hz, 1H), 3.11 (d, *J* = 10.2 Hz, 0.5H), 3.02 – 2.99 (m, 0.5H), 2.90 (d, *J* = 16.6 Hz, 0.5H), 2.81 (d, *J* = 14.0 Hz, 0.5H), 2.79 (s, 1.5H), 2.66 (s, 1.5H), 2.58 (d, J = 8.0 Hz, 0.5H), 2.44 (d, *J* = 16.0 Hz, 1.1 Hz, 0.5H), 2.24 (s, 1.5H), 2.14 (s, 1.5H), 1.44 (s, 1.4H), 1.28 – 1.22 (m, 6H), 1.17 (s, 1.6H). ^{13}C NMR (201 MHz, $CDCl_3$) δ 171.8, 171.7, 171.3, 171.2, 147.1, 146.6, 146.3, 144.7, 138.9, 138.5, 136.5, 135.9, 135.7, 135.6, 132.2, 132.1, 131.0, 130.9, 130.7, 129.6, 129.5, 129.4, 128.6, 128.3, 125.8, 125.5, 118.8, 118.7, 110.5, 110.4, 63.5, 62.1, 61.9, 61.8 (2C), 61.6, 57.7, 57.6, 46.4, 45.5, 44.9, 44.8, 44.3, 44.0, 40.7, 40.0, 28.4, 25.8, 20.5, 20.3, 14.1, 14.0 (3C). HRMS ESI (m/z): $[M+Na]^+$ calcd. for $C_{29}H_{33}NO_6SNa$: 546.1921, found: 546.1930.

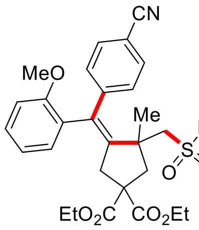

43

The product **43** was purified with silica gel chromatography (PE/EA = 2:1) as a white solid (0.2 mmol scaled, 95.9 mg, 89%); m.p. 128.8-130.2 °C. ^{1}H NMR (500 MHz, $CDCl_3$) δ 7.57 (d, *J* = 7.8 Hz, 2H), 7.44 (d, *J* = 8.0 Hz, 1H), 7.38 (d, *J* = 8.0 Hz, 1H), 7.30 (d, *J* = 8.0 Hz, 1H), 7.18 (d, *J* = 6.0 Hz, 1H), 6.95 (dd, *J* = 17.3, 7.9 Hz, 1H), 6.86 (d, *J* = 8.2 Hz, 1H), 4.21-4.16 (m, 4H), 3.79 (d, *J* = 3.6 Hz, 3H), 3.28 – 3.20 (m, 1.4H), 3.11 (d, *J* = 14.4 Hz, 1H), 3.06 – 2.98 (m, 1H), 2.96 (d, *J* = 7.8 Hz, 0.6H), 2.93 – 2.85 (m, 1H), 2.77 (s, 1.2H), 2.72 (s, 1.8H), 2.58 (d, *J* = 14.3 Hz, 0.6H), 2.49 (d, *J* = 14.3 Hz, 0.4H), 1.36 (s, 1.8H), 1.27 – 1.24 (m, 6H), 1.18 (s, 1.2H). ^{13}C NMR (201 MHz, CDCl3) δ 171.7, 171.6, 171.4, 171.3, 156.4, 155.9, 148.3, 147.9, 146.2, 145.0, 133.0, 132.9, 132.3 (2C), 131.2, 130.1, 129.8, 129.6, 129.0, 128.9, 128.6, 128.3, 120.8, 120.3, 119.0, 118.9, 111.3, 111.1, 110.4 (2C), 63.1, 62.1, 62.0 (2C), 61.8, 61.7, 57.7, 57.6, 55.3, 55.0, 46.1, 45.5, 44.7, 44.2, 43.9, 40.5, 40.1, 27.4, 25.7, 14.0 (4C). HRMS ESI (m/z): $[M+H]^+$ calcd. for $C_{29}H_{33}NO_7S$: 540.2050, found: 540.2062.

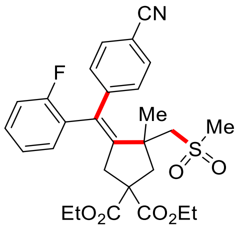

44

The product **44** was purified with silica gel chromatography (PE/EA = 2:1) as a white solid (0.2 mmol scaled, 74.8 mg, 71%); m.p. 126.4-128.3 °C. ^{1}H NMR (500 MHz, $CDCl_3$) δ 7.63 – 7.59 (m, 2H), 7.41 (dd, *J* = 24.2, 6.9 Hz, 2H), 7.31 (s, 2H), 7.16 (s, 1H), 7.08 (t, *J* = 8.9 Hz, 1H), 4.22-4.17 (m, 4H), 3.29 (t, *J* = 14.5 Hz, 1H), 3.24 – 3.16 (m, 1H), 3.11 – 3.01 (m, 2H), 2.93 (d, *J* = 15.9 Hz, 1H), 2.81 (d, *J* = 16.0 Hz, 3H), 2.53 (dd, *J* = 39.7, 14.2 Hz, 1H), 1.36 – 1.21 (m, 9H). ^{13}C NMR (201 MHz, $CDCl_3$) δ 171.4, 171.3, 171.2, 159.1 (d, *J* = 244.7 Hz), 158.8 (d, *J* = 244.0 Hz), 148.5, 147.5, 147.1, 146.9, 132.6, 131.7, 130.7, 130.4, 130.2, 130.0, 129.8, 128.8, 127.5 (d, *J* = 17.1 Hz), 127.0 (d, *J* = 17.4 Hz), 124.5, 124.3, 118.8, 118.7, 116.4 (d, *J* = 21.8 Hz), 116.0 (d, *J* = 21.5 Hz), 111.0, 110.8, 62.9, 62.0, 61.9, 57.6, 57.5, 45.8, 45.7, 44.9, 44.5, 44.4, 44.0, 41.1, 40.4, 27.6, 26.6, 14.0 (2C). ^{19}F NMR (753 MHz, $CDCl_3$) δ -110.5 (s, 0.5F), -112.1 (s, 0.5F). HRMS ESI (m/z): $[M+Na]^+$ calcd. for $C_{28}H_{30}FNO_6SNa$: 550.1670, found: 550.1678.

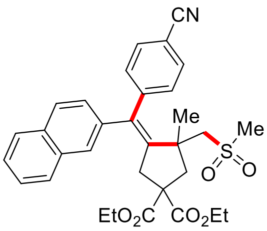

45

The product **45** was purified with silica gel chromatography (PE/EA = 2:1) as a white solid (0.2 mmol scaled, 99.5 mg, 89%); m.p. 108.4-110.5 °C. ^{1}H NMR (400 MHz, CDCl$_3$) δ 7.83 – 7.78 (m, 3H), 7.76 (s, 1H), 7.58 (d, *J* = 8.4 Hz, 2H), 7.52 – 7.47 (m, 2H), 7.45 (d, *J* = 8.1 Hz, 2H), 7.35 – 7.31 (m, 1H), 4.27 – 4.16 (m, 4H), 3.30 (d, *J* = 14.3 Hz, 1H), 3.16 (d, *J* = 14.0 Hz, 1H), 3.08 (d, *J* = 4.0 Hz, 2H), 2.99 (d, *J* = 14.0 Hz, 1H), 2.69 (s, 3H), 2.49 (d, *J* = 14.3 Hz, 1H), 1.31 (s, 3H), 1.29-1.22 (m, 6H). ^{13}C NMR (101 MHz, CDCl$_3$) δ 171.6, 171.3, 148.2, 145.1, 137.2, 136.3, 132.9, 132.5, 132.4, 128.9, 128.4, 128.0, 127.8 (2C), 127.1, 126.9, 126.6, 118.8, 110.6, 62.8, 62.0, 61.8, 57.6, 45.9, 44.7, 44.1, 41.0, 29.1, 14.1, 14.0. HRMS ESI (m/z): [M+Na]$^+$ calcd. for $C_{32}H_{33}NO_6SNa$: 582.1921, found: 582.1932.

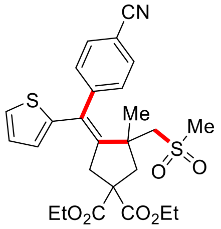

46

The product **46** was purified with silica gel chromatography (PE/EA = 2:1) as a white solid (0.2 mmol scaled, 83.4mg, 81%); m.p. 110.2-112.1 °C. ^{1}H NMR (800 MHz, CDCl$_3$) δ 7.62 – 7.60 (m, 2H), 7.38 – 7.37 (m, 2H), 7.31 (dd, *J* = 5.1, 1.0 Hz, 1H), 6.98 (dd, *J* = 5.1, 3.5 Hz, 1H), 6.95 (dd, *J* = 3.5, 1.1 Hz, 1H), 4.23 – 4.15 (m, 4H), 3.28 (dd, *J* = 14.1, 2.3 Hz, 2H), 3.19 (d, *J* = 13.9 Hz, 1H), 3.05 – 3.02 (m, 1H), 2.95 (d, *J* = 17.3 Hz, 1H), 2.83 (s, 3H), 2.54 (dd, *J* = 14.4, 1.2 Hz, 1H), 1.43 (s, 3H), 1.26 – 1.22 (m, 6H). ^{13}C NMR (201 MHz, CDCl$_3$) δ 171.2, 171.1, 149.5, 147.8, 140.1, 132.5, 128.7, 128.5, 127.5, 126.9, 126.7, 118.6, 110.7, 62.1, 61.9, 61.7, 57.3, 45.9, 44.8, 44.0, 41.0, 28.6, 13.9, 13.8. HRMS ESI (m/z): [M+Na]$^+$ calcd. for $C_{26}H_{29}NO_6S_2Na$: 538.1329, found: 538.1331.

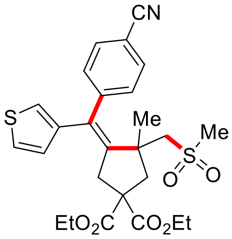

47

The product **47** was purified with silica gel chromatography (PE/EA = 2:1) as a white solid (0.2 mmol scaled, 86.5mg, 84%); m.p. 128.0-130.8 °C. ^{1}H NMR (500 MHz, CDCl$_3$) δ 7.62 – 7.58 (m, 2H), 7.34 (dd, *J* = 17.9, 5.3 Hz, 3H), 7.19 – 7.17 (m, 1H), 6.9

4 – 6.91 (m, 1H), 4.23-4.14 (m, 4H), 3.25 (d, *J* = 14.2 Hz, 1H), 3.16 (d, *J* = 14.0 Hz, 1 H), 3.08 – 3.00 (m, 2H), 2.96 (d, *J* = 17.2 Hz, 1H), 2.80 (s, 3H), 2.49 (d, *J* = 14.2 Hz, 1H), 1.33 (d, *J* = 3.9 Hz, 3H), 1.27-1.21 (m, 6H). ^{13}C NMR (126 MHz, $CDCl_3$) δ 171. 5, 171.3, 147.7, 146.5, 139.4, 132.5, 131.5, 128.8, 128.6, 126.4, 123.4, 118.8, 110.6, 6 2.6, 62.0, 61.8, 57.6, 46.0, 44.6, 44.2, 40.8, 28.8, 14.0 (2C). HRMS ESI (m/z): [M+N a]$^+$ calcd. for $C_{26}H_{29}NO_6S_2Na$: 538.1329, found: 538.1330.

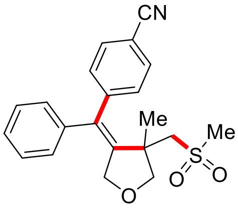

48

The product **48** was purified with silica gel chromatography (PE/EA = 2:1) as a white solid (0.2 mmol scaled, 25.6 mg, 35%); m.p. 130.3-132.2 °C. ^{1}H NMR (400 MHz, $CDCl_3$) δ 7.62 – 7.58 (m, 2H), 7.40 (dd, *J* = 7.9, 6.4 Hz, 2H), 7.36 – 7.31 (m, 3H), 7.28 (d, *J* = 1.6 Hz, 1H), 7.26 – 7.25 (m, 1H), 4.45 (d, *J* = 14.0 Hz, 1H), 4.34 – 4.25 (m, 2H), 3.84 (d, *J* = 9.3 Hz, 1H), 3.12 (d, *J* = 14.2 Hz, 1H), 2.98 (d, *J* = 14.3 Hz, 1H), 2.81 (s, 3H), 1.33 (s, 3H). ^{13}C NMR (101 MHz, $CDCl_3$) δ 146.7, 144.5, 138.8, 134.1, 132.5, 129.1, 128.7, 128.4, 128.1, 118.6, 111.0, 78.9, 71.9, 60.7, 45.4, 43.5, 24.8. HRMS ESI (m/z): [M+Na]$^+$ calcd. for $C_{21}H_{21}NO_3SNa$: 390.1134, found: 390.1141.

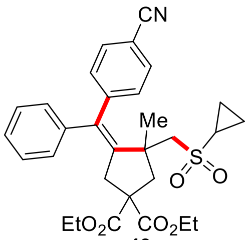

49

The product **49** was purified with silica gel chromatography (PE/EA = 2:1) as a white solid (0.2 mmol scaled, 80.4 mg, 75%); m.p. 130.5-132.3 °C. ^{1}H NMR (400 MHz, $CDCl_3$) δ 7.59 (d, *J* = 8.2 Hz, 2H), 7.38 (d, *J* = 8.3 Hz, 2H), 7.35 – 7.30 (m, 2H), 7.29 – 7.27 (m, 1H), 7.24 (d, *J* = 6.7 Hz, 2H), 4.24 – 4.13 (m, 4H), 3.24 (d, *J* = 14.3 Hz, 1H), 3.11 (d, *J* = 14.0 Hz, 1H), 3.04 – 2.97 (m, 3H), 2.50 (d, *J* = 14.2 Hz, 1H), 2.22 (s, 1H), 1.31 (s, 3H), 1.27 – 1.22 (m, 6H), 1.10-1.08 (m, 2H), 0.93 – 0.87 (m, 2H). ^{13}C NMR (101 MHz, $CDCl_3$) δ 171.6, 171.5, 148.3, 145.1, 139.8, 136.3, 132.6, 129.3, 128.8, 128.5, 127.7, 118.9, 110.5, 62.0 (2C), 61.8, 57.6, 46.2, 44.6, 40.7, 32.3, 28.8, 14.1, 14.0, 5.1, 4.9. HRMS ESI (m/z): [M+Na]$^+$ calcd. for $C_{30}H_{33}NO_6SNa$: 558.1921, found: 558.1926.

1 mmol-scale experiment:

In a 10 mL oven-dried Schlenk tube, 10 mL DMSO was added to a mixture of $NiCl_2$ (13.0 mg, 0.1 mmol, 10 mol%), dmbpy (18.0 mg, 0.1 mmol, 10 mol%), sodium methanesulfinate (204.2 mg, 2 mmol, 2.0 equiv), 4-iodobenzonitrile (458.0 mg, 2 mmol, 2.0 equiv) and 1,6-enyne **1a** (328.4 mg, 1 mmol, 1.0 equiv) under N_2

atmosphere. The tube was sealed with a Teflon lined cap and the reaction was stirred and irradiated with 6 W purple LED lamps (395-415 nm, with cooling fan to keep the reaction temperature near 25 °C) for 24 h. The reaction mixture was quenched with water and extracted with EtOAc (50 mL x 2). The combined organic phase was washed with brine (100 mL), dried over anhydrous Na_2SO_4, concentrated in vacuo. The residue as purified by flash column chromatography (PE/EA = 2:1) to give the product (397.2 mg, 77% yield).

Radical trap experiments

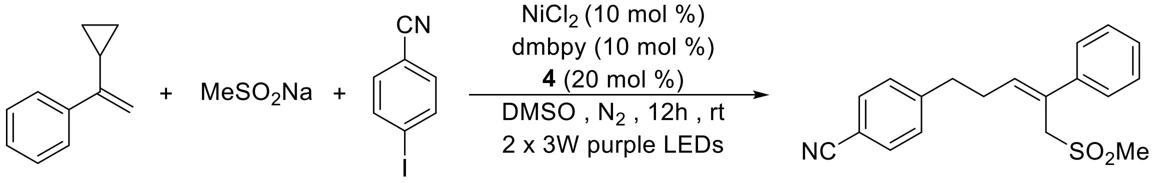

1aa **2a** **3a** **50**

In a 10 mL oven-dried Schlenk tube, 2.0 mL DMSO was added to a mixture of $NiCl_2$ (2.6 mg, 0.02 mmol, 10 mol%), dmbpy (3.7 mg, 0.02 mmol, 10 mol%), sodium methanesulfinate (40.8 mg, 0.4 mmol, 2.0 equiv), 4-iodobenzonitrile (91.6 mg, 0.4 mmol, 2.0 equiv), **1aa**[6] (28.8, 0.2 mmol, 1.0 equiv) and **4** (20.4 mg, 0.04 mmol, 20 mol%) under N_2 atmosphere. The tube was sealed with a Teflon lined cap and the reaction was stirred and irradiated with 6 W purple LED lamps (395-415 nm, with cooling fan to keep the reaction temperature near 25 °C) for 12 h. The reaction mixture was quenched with water and extracted with EtOAc (10 mL x 2). The combined organic phase was washed with brine (20 mL), dried over anhydrous Na_2SO_4, concentrated in vacuo. The residue as purified by flash column chromatography (PE/EA = 2:1) to give the radical trapping product **50** and 4-(methylsulfonyl)benzonitrile as a colorless oil unseparated mixture (19.6 mg, 30% yield). The NMR data of 4-(methylsulfonyl)benzonitrile (^{1}H NMR δ 8.09 (d, J = 8.6 Hz, 2H, 7.89 (d, J = 8.6 Hz, 2H), 3.10 (s, 3H). ^{13}C NMR δ 144.5, 133.2, 128.2, 117.7, 117.0, 44.3) matched the previous report.[7]

^{1}H NMR (800 MHz, $CDCl_3$) δ 7.61 – 7.58 (m, 2H), 7.39 – 7.30 (m, 7H), 6.09 (t, J = 7.4 Hz, 1H), 4.17 (s, 2H), 2.90 (t, J = 7.7 Hz, 2H), 2.69 (q, J = 7.6 Hz, 2H), 2.52 (s, 3H). ^{13}C NMR (201 MHz, $CDCl_3$) δ 146.8, 140.8, 137.3, 132.3, 129.4, 129.1, 128.9, 128.1, 126.5, 119.0, 110.1, 56.5, 41.4, 35.3, 31.1. HRMS ESI (m/z): $[M+Na]^+$ calcd. for $C_{19}H_{19}NO_2SNa$: 348.1029, found: 348.1038.

Light on/off experiment

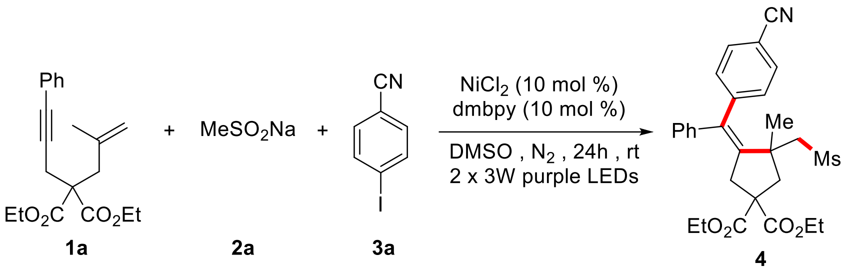

In a 10 mL oven-dried Schlenk tube, 2.0 mL DMSO and acetophenone (6.0 mg, 0.05 mmol, 0.5 equiv., as internal standard) was added to a mixture of $NiCl_2$ (1.3 mg, 0.01 mmol, 10 mol%), dmbpy (1.8 mg, 0.01 mmol, 10 mol%), sodium methanesulfinate (20.4 mg, 0.2 mmol, 2.0 equiv), 4-iodobenzonitrile (45.8 mg, 0.2 mmol, 2.0 equiv) and 1,6-enyne **1a** (32.8 mg, 0.1 mmol, 1.0 equiv) under N_2 atmosphere. The tube was sealed with a Teflon lined cap and the reaction was stirred and irradiated with 2 x 3 W purple LED lamps (395-415 nm, with cooling fan to keep the reaction temperature near 25 °C). 0.1 mL of the reaction mixture was taken out every four hours, and the yield of **4** was detected by crude HPLC.

Table S1. The yield of **4** with light on/off at different time intervals.

Time (h)	0	4	8	12	16	20	24
Yield (%)	0	4.6	5.0	13.7	14.4	29.7	30.0

Time-dependent experiment

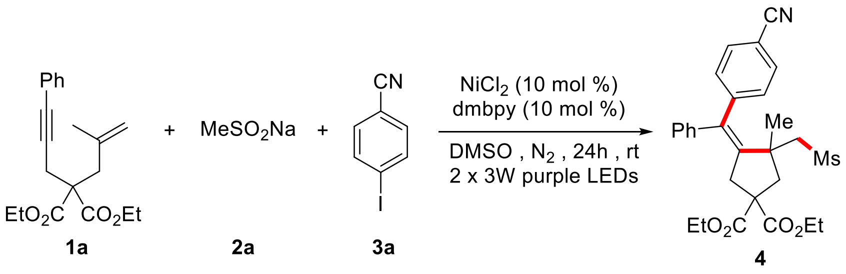

In a 25 mL oven-dried Schlenk tube, 6.0 mL DMSO and acetophenone (18.0 mg, 0.15 mmol, 0.5 equiv., as internal standard) was added to a mixture of $NiCl_2$ (3.9 mg, 0.03 mmol, 10 mol%), dmbpy (5.4 mg, 0.03 mmol, 10 mol%), sodium methanesulfinate (61.2 mg, 0.6 mmol, 2.0 equiv), 4-iodobenzonitrile (137.4 mg, 0.6 mmol, 2.0 equiv) and 1,6-enyne **1a** (98.4 mg, 0.3 mmol, 1.0 equiv) under N_2 atmosphere. The tube was sealed with a Teflon lined cap and the reaction was stirred and irradiated with 2 x 3 W purple LED lamps (395-415 nm, with cooling fan to keep the reaction temperature near 25 °C). 0.1 mL of the reaction mixture was taken out at each sampling time, and the yield of **4** was detected by crude HPLC.

Table S2. The yield of **4** with light on/off at different time intervals.

Time (h)	0	1	2	4	6	8	10
Yield (%)	0	0.6	1.7	4.5	7.9	12.5	17.9
Time (h)	12	14	16	18	20	22	24
Yield (%)	23.6	31.7	46.1	60.3	70.6	74.4	79.1

Computational Details

All density functional theory (DFT) and time-dependent (TD)-DFT calculations were carried out using Gaussian 16 software package.[8] The geometry optimizations were carried out using the B3LYP[9,10] functional and def2-SVP[11] basis set, including Grimme's D3 (BJ-damping) dispersion corrections,[12] with the SMD[13] implicit solvation method (n,n-dimethylformamide as solvent.) The vibrational frequencies were computed at the same level of theory as for the geometry optimizations to evaluate the zero-point vibrational energy (ZPVE) and thermal corrections at 298 K, as well as to identify optimized stationary points as minima or transition states. The simulated UV-Vis spectrums were obtained by performing TD-DFT calculations[14,15] at the same level of theory as for the geometry optimizations. The interaction region indicator (IRI) analysis[16] was employed to unveil the isosurfaces of both chemical bonds and weak interaction regions. The Mayer bond order, spin population analysis and IRI analysis were performed by using the Multiwfn 3.8(dev) program.[17] The three-dimensional structures for IRI analysis and contour map of spin density generated using VMD software.[18]

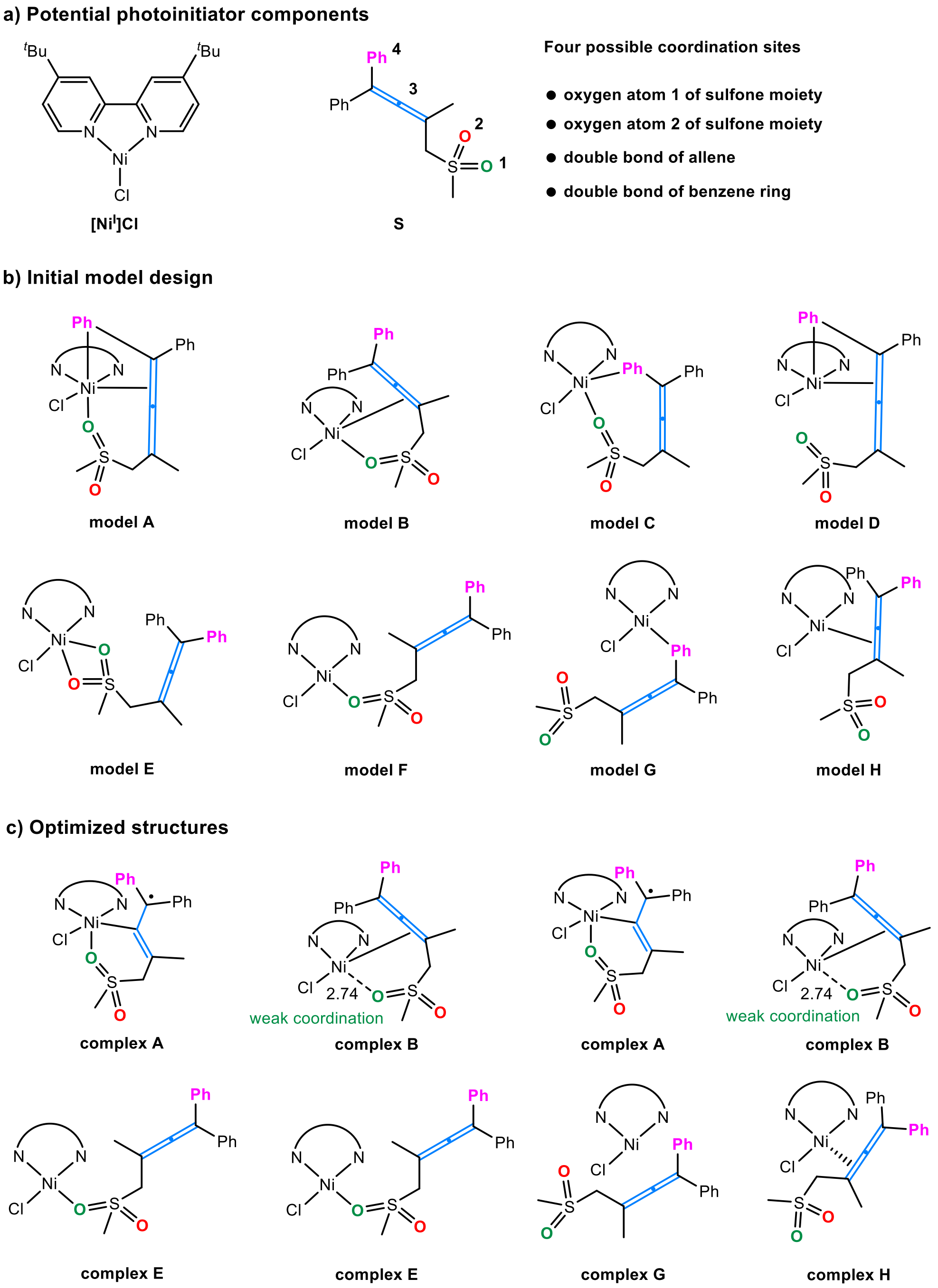

Figure S1. a) Potential photoinitiator components; b) coordination mode design; c) corresponding optimized structures.

Detailed mechanistic studies in our previous report[19] on visible-light-induced, autopromoted nickel-catalyzed three-component arylsulfonation of 1,3-enynes suggest that both [Ni^I]Cl and sulfonyl allene are crucial to achieve the visible-light-induced, nickel-catalyzed arylsulfonation. In other words, the photoactive species may be the coordination compound of [Ni^I]Cl and sulfonyl allene. As depicted by Figure S1a, there are four potential coordination sites in sulfonyl allene **S**, leading to various possible coordination conformations. To verify which conformation plays a substantial role in the light absorption as a virtual photoinitiator, we performed DFT

and TD-DFT calculations for various possible coordination conformations of [Ni^{I}]Cl and sulfonyl allene. Firstly, we designed eight possible coordination models (Figure S1b): model **A** (coordinate with three coordination sites), model **B**-**E** (coordinate with two coordination sites), model **F**-**H** (coordinate with one coordination site). Subsequently, the above models were first subjected to a constricted optimization, followed by a free optimization operation based on the structure of the preceding constricted optimization. Five corresponding optimized structures are shown in Figure S1c. Complex **A** is the optimized structure of model **A** and **C**. Complex **B** is the optimized structure of model **B** and **D**. Complex **E** is the optimized structure of model **E** and **F**. Complex **G** is the optimized structure of model **G**. Complex **H** is the optimized structure of model **H**. Finally, UV-Vis spectrum simulation was carried out with TD-DFT. The maximum absorption wavelengths of the aforementioned complexes **A**, **B**, **E**, **G**, **H** are 400, 372, 472, 456, 358nm, respectively. Only the maximum absorption of complexes A falls in the purple light (395–415 nm). So we speculate that complex **A** is the virtual photoinitiator in the reaction of visible-light-induced, autopromoted nickel-catalyzed three-component arylsulfonation of 1,3-enynes.

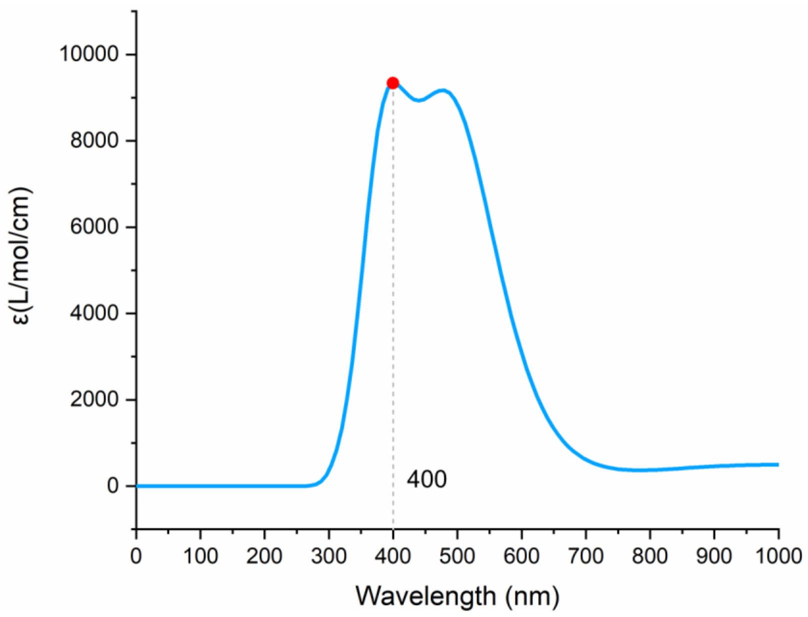

Figure S2. Simulated UV-Vis spectrum of complex **A**.

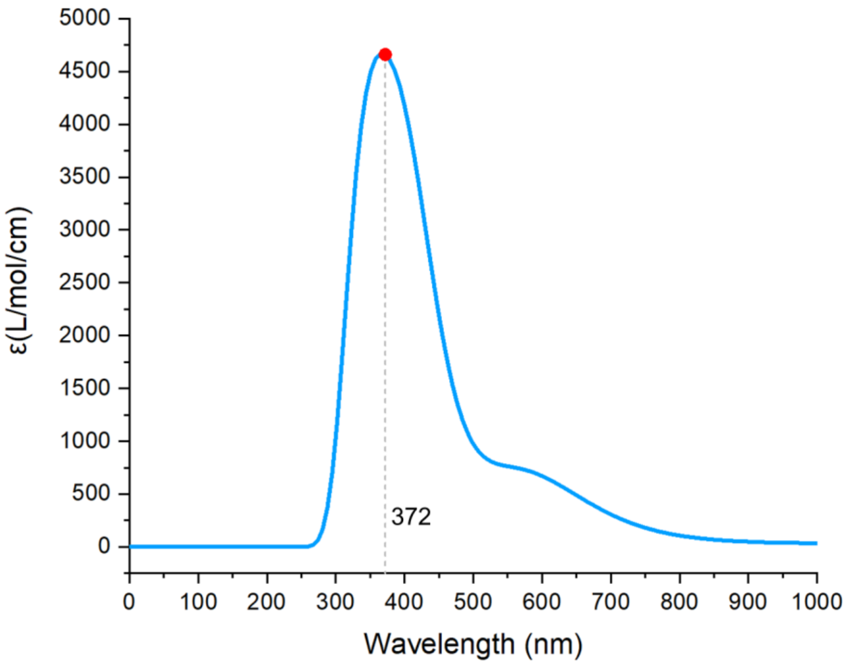

Figure S3. Simulated UV-Vis spectrum of complex **B**.

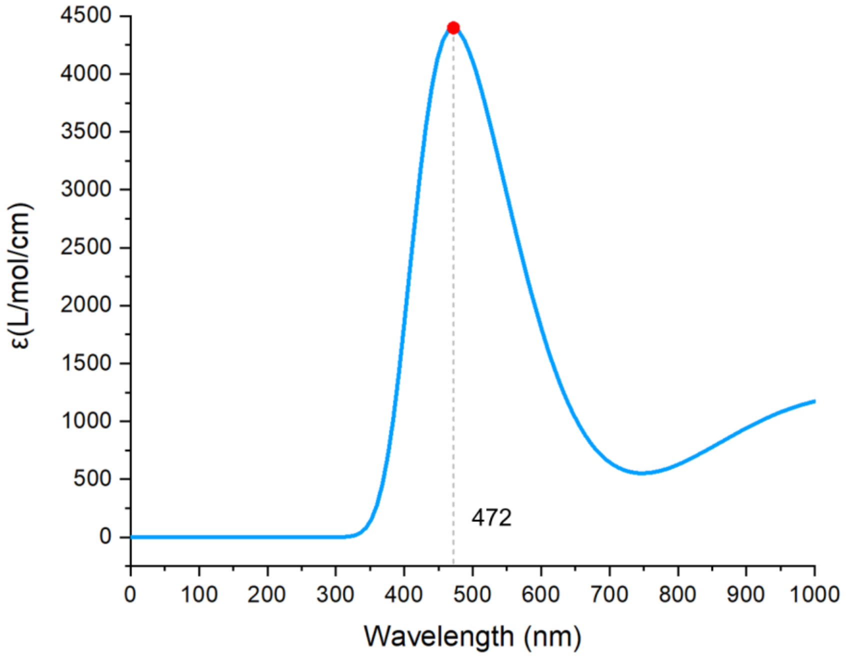

Figure S4. Simulated UV-Vis spectrum of complex **E**.

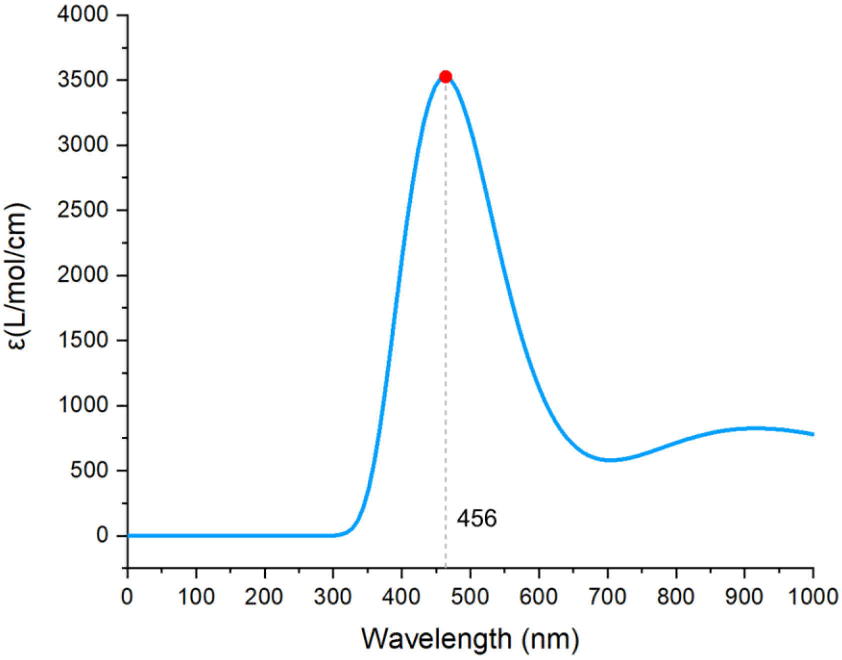

Figure S5. Simulated UV-Vis spectrum of complex **G**.

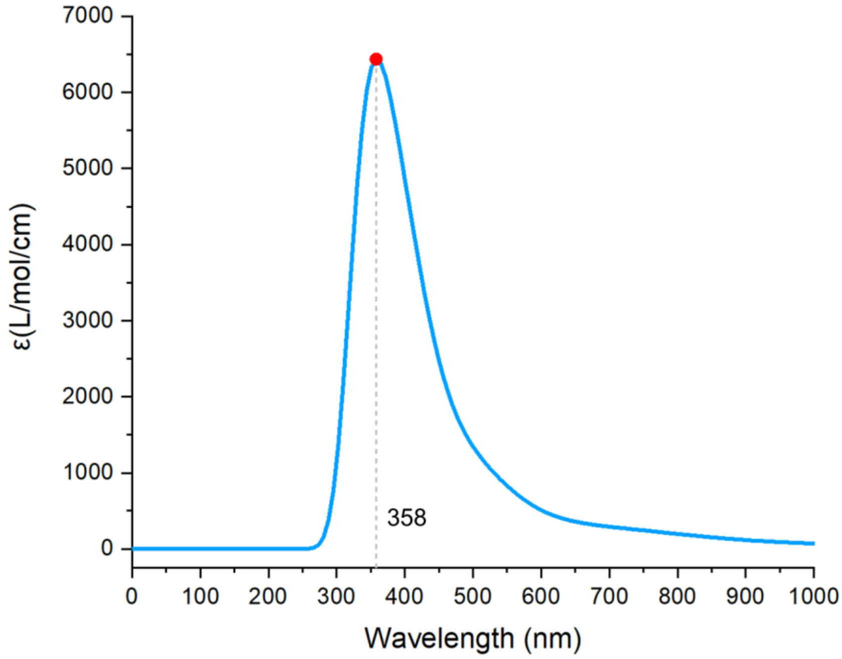

Figure S6. Simulated UV-Vis spectrum of complex **H**.

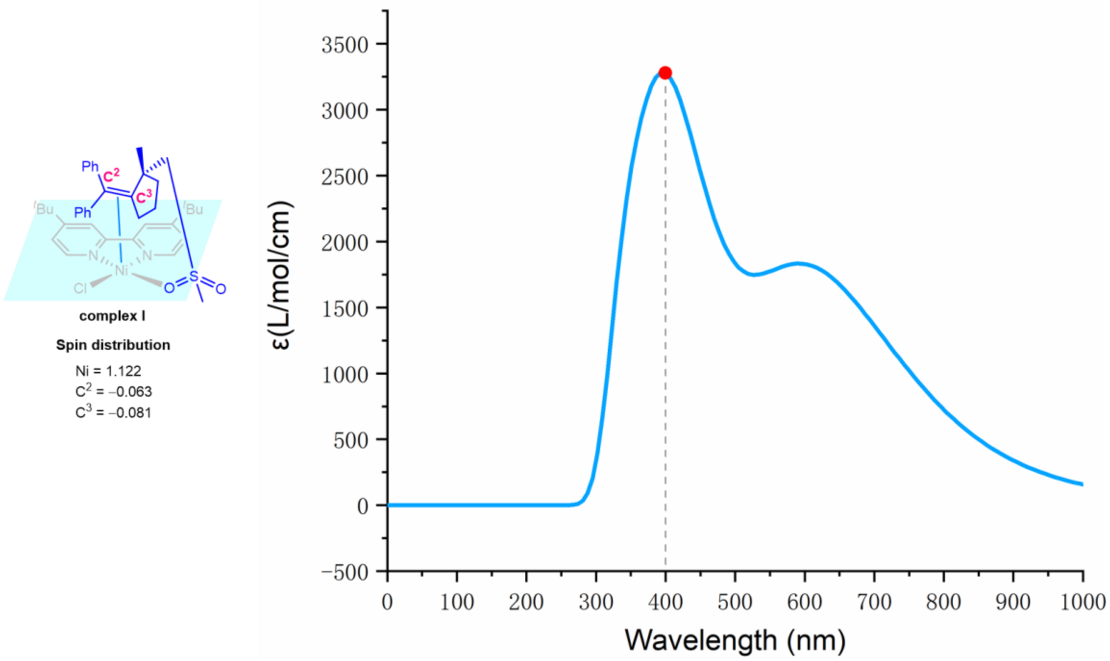

Figure S7. Structure and simulated UV-Vis spectrum of complex I.

Cartesian coordinates of the calculated species

complex A

Imaginary Frequency: no

Electronic Energy (EE): -4022.490396

Thermal Correction to Enthalpy: 0.744304

Thermal Correction to Free Energy: 0.619229

EE + Thermal Enthalpy Correction: -4021.746092

EE + Thermal Free Energy Correction: -4021.871167

0 2

C	1.52967500	1.15126000	0.23194600
C	-0.58336600	2.05922100	0.47000500
C	-0.08901100	3.36109400	0.41711500
C	1.28819800	3.57294100	0.27149500
C	2.09391100	2.42711000	0.17688000
C	2.32873600	-0.09625300	0.10316700
C	2.28015100	-2.40699300	0.11518700
C	3.64578400	-2.48851600	-0.15777500
C	4.39333800	-1.31320100	-0.30873700
C	3.69771300	-0.10096000	-0.17045300
H	-1.65357400	1.87195700	0.57180600
H	-0.79313600	4.18805700	0.48983600
H	3.17098000	2.53244600	0.06087300
H	1.65692100	-3.29796500	0.23345800
H	4.10138300	-3.47300900	-0.25176600
H	4.22792500	0.84207100	-0.28784100

N	1.64000100	-1.24561200	0.24240200
N	0.19963900	0.98255500	0.38631400
C	1.92363800	4.96452300	0.21461600
C	2.89890700	5.11245400	1.40090500
H	3.70404100	4.36290100	1.36497400
H	3.36618000	6.10997500	1.38041300
H	2.37129500	5.00138000	2.36161300
C	0.87173400	6.07897800	0.29740900
H	1.37041200	7.05906500	0.24704500
H	0.15347500	6.02612900	-0.53554600
H	0.30710500	6.04042100	1.24188600
C	2.69678500	5.11035900	-1.11228000
H	2.02377600	4.99100900	-1.97631700
H	3.15449600	6.11071100	-1.17112500
H	3.50355800	4.36723300	-1.20210500
C	5.89316900	-1.30192200	-0.61646400
C	6.63012900	-0.56106700	0.51833800
H	7.71290900	-0.54514100	0.31541300
H	6.29113600	0.48151900	0.61521100
H	6.47093900	-1.06332300	1.48588800
C	6.12242500	-0.56254300	-1.95083200
H	7.19755000	-0.54542500	-2.19090300
H	5.59550700	-1.06664500	-2.77669300
H	5.77127400	0.47958900	-1.90732000
C	6.46605000	-2.72071400	-0.73346800
H	6.34576200	-3.28909400	0.20187900
H	5.98771400	-3.28973800	-1.54572800
H	7.54315000	-2.66534800	-0.95416500
Ni	-0.50761200	-1.01957900	0.61274800
Cl	-0.87435900	-3.37567600	0.67474100
C	-2.66370100	-0.48901300	-1.07109500
C	-2.47402300	-0.54265300	0.31020100
C	-3.34028400	-0.37088800	1.35046000
C	-1.83078600	-1.38548500	-1.94244000
C	-0.54922700	-0.98683200	-2.35929100
C	-2.29621200	-2.65170900	-2.33064600
C	0.26359600	-1.84756600	-3.10624800
H	-0.18694800	0.00888300	-2.09646500
C	-1.49585200	-3.50531800	-3.09064700
H	-3.28924600	-2.97372800	-2.00820900
C	-0.20740000	-3.11042300	-3.47147300
H	1.26691400	-1.52750500	-3.39953800
H	-1.86991000	-4.49285300	-3.37395700
H	0.42476700	-3.78633700	-4.05320700
C	-3.48308600	0.52605500	-1.73689100

C	-3.64010300	0.52047900	-3.14792800
C	-4.13264000	1.57039300	-1.02735500
C	-4.41765200	1.47725300	-3.79974100
H	-3.14814900	-0.25499100	-3.73700900
C	-4.90084400	2.52790100	-1.68401500
H	-4.01141400	1.63201100	0.05383400
C	-5.05832600	2.49024800	-3.07672900
H	-4.52207400	1.43230800	-4.88752600
H	-5.38068100	3.32019800	-1.10237100
H	-5.66408700	3.24202500	-3.58858700
C	-4.81053800	-0.70643700	1.26731700
H	-5.08297900	-1.45960000	2.02950900
H	-5.44834600	0.17641600	1.45838800
H	-5.07310700	-1.10897800	0.27927400
C	-2.89183900	0.15786500	2.68381800
H	-2.38227600	1.13556400	2.62878600
H	-3.71871000	0.24784400	3.40269500
S	-1.64589400	-0.81038100	3.58112700
O	-1.50129800	-0.25211200	4.93661900
O	-0.39363400	-0.82612400	2.75250800
C	-2.27435400	-2.47496800	3.67676900
H	-3.24412300	-2.44459000	4.19143300
H	-2.32977900	-2.88177600	2.65707100
H	-1.53527300	-3.03361800	4.26878000

complex B

Imaginary Frequency: no

Electronic Energy (EE): -4022.505692

Thermal Correction to Enthalpy: 0.746019

Thermal Correction to Free Energy: 0.622083

EE + Thermal Enthalpy Correction: -4021.759673

EE + Thermal Free Energy Correction: -4021.883609

0 2

C	-1.33677200	0.41650600	-1.13791000
C	0.59678300	1.37017400	-1.95796100
C	-0.07354400	2.53330800	-2.33416900
C	-1.45206800	2.64366700	-2.10641300
C	-2.07587900	1.54479200	-1.49920500
C	-1.93993700	-0.75290900	-0.45174500
C	-1.53756800	-2.78050100	0.58304900
C	-2.88441400	-2.94310600	0.90146600
C	-3.81233400	-1.96707000	0.51392100
C	-3.30506600	-0.85980100	-0.18178900
H	1.66159900	1.25302400	-2.15623600

H	0.49635300	3.33360800	-2.80335100
H	-3.14226800	1.57615700	-1.28561200
H	-0.79114900	-3.53317700	0.84558400
H	-3.18579800	-3.83564800	1.44728300
H	-3.97818000	-0.07015600	-0.50919500
N	-1.07585200	-1.71433800	-0.07242100
N	-0.01190100	0.33988300	-1.37114000
C	-2.27125900	3.88380800	-2.47386400
C	-3.39208100	3.47314000	-3.45048400
H	-4.07146000	2.73040500	-3.00540600
H	-3.99249400	4.35471400	-3.72665900
H	-2.97281900	3.04224000	-4.37372400
C	-1.40888300	4.96542900	-3.13806700
H	-2.03632800	5.83488600	-3.38775000
H	-0.60513900	5.31470500	-2.47143800
H	-0.94929300	4.60585600	-4.07184200
C	-2.89447800	4.46607800	-1.18825000
H	-2.11306800	4.76035900	-0.46990900
H	-3.48942000	5.36102900	-1.43123300
H	-3.56073400	3.74348700	-0.69376200
C	-5.30916600	-2.05694700	0.82457200
C	-6.09977200	-2.03407700	-0.49953100
H	-7.18002100	-2.10199000	-0.29366300
H	-5.92437200	-1.10726000	-1.06652300
H	-5.81981200	-2.88495000	-1.14096400
C	-5.71297300	-0.84381700	1.68820600
H	-6.78937300	-0.88974100	1.91890300
H	-5.16007700	-0.83601100	2.64050100
H	-5.52044800	0.10891300	1.17263500
C	-5.66028400	-3.34179400	1.58684500
H	-5.40846200	-4.24345100	1.00711700
H	-5.13865800	-3.39863900	2.55490400
H	-6.74216600	-3.36605700	1.78912300
Ni	0.97041000	-1.43446400	-0.49734500
Cl	1.33973600	-3.77645200	-0.78772600
C	1.56882500	0.53487600	1.74850100
C	1.96946500	-0.34923900	0.82400000
C	3.05296200	-0.98899700	0.25023200
C	0.11555100	0.78820000	1.99675200
C	-0.46871100	2.01281300	1.63918200
C	-0.67945200	-0.17989400	2.62867400
C	-1.81935500	2.25945500	1.89683700
H	0.14045500	2.77396700	1.14568600
C	-2.02784800	0.06739500	2.89550700
H	-0.23088100	-1.13526000	2.90902300

C	-2.60280400	1.28779900	2.52783700
H	-2.26322900	3.21121400	1.59654000
H	-2.63422400	-0.69972200	3.38250100
H	-3.65964400	1.47888300	2.72795000
C	2.53812200	1.28273500	2.60265900
C	3.90889200	1.36937500	2.28385300
C	2.10082000	1.92081700	3.78150500
C	4.79984800	2.05771900	3.10673400
H	4.28375600	0.90242600	1.37498900
C	2.99448200	2.60802700	4.60708000
H	1.04796900	1.87185800	4.06200300
C	4.34985300	2.68187900	4.27605900
H	5.85571600	2.11137700	2.82865000
H	2.62494600	3.08806800	5.51711600
H	5.04906400	3.22185200	4.91933700
C	3.68021200	-2.16592500	0.96418100
H	2.95657300	-2.65556600	1.62770800
H	4.05933600	-2.92781100	0.27012300
H	4.53129700	-1.80558200	1.57141500
C	3.86325000	-0.32760800	-0.82694500
H	3.58454400	0.72431800	-0.98511700
H	4.94410700	-0.37426000	-0.61745500
S	3.70536200	-0.99987700	-2.52321300
O	4.61839700	-0.20878900	-3.37286200
O	2.26536000	-1.02195300	-2.87706700
C	4.27691000	-2.69182200	-2.47381200
H	5.29184200	-2.71879700	-2.05566000
H	3.55059000	-3.27889600	-1.89138400
H	4.28486200	-3.01745700	-3.52447700

complex E

Imaginary Frequency: no

Electronic Energy (EE): -4022.497421

Thermal Correction to Enthalpy: 0.744543

Thermal Correction to Free Energy: 0.619208

EE + Thermal Enthalpy Correction: -4021.752878

EE + Thermal Free Energy Correction: -4021.878213

0 2

C	-2.15797800	0.89310200	-0.15400800
C	0.08427900	0.77589300	-0.72249700
C	0.23102300	2.15572500	-0.62861200
C	-0.86961800	2.95396300	-0.27844000
C	-2.08052700	2.28291600	-0.04692300
C	-3.38731300	0.10628000	0.08377600

C	-4.27058000	-2.03321500	0.18661800
C	-5.54326900	-1.54505600	0.47090300
C	-5.75460500	-0.16037100	0.57440500
C	-4.63450800	0.66141900	0.37557600
H	0.92327300	0.14014100	-0.99967200
H	1.20943600	2.58516100	-0.83243000
H	-2.97273700	2.84596600	0.22472800
H	-4.06870200	-3.10564500	0.12285300
H	-6.35378900	-2.25834600	0.61300900
H	-4.73296200	1.74405500	0.44639200
N	-3.21386500	-1.23566900	-0.00821100
N	-1.07469200	0.14468900	-0.48672400
C	-0.79034600	4.47728000	-0.14518100
C	-1.77804500	5.12237300	-1.13865700
H	-2.81613100	4.81356000	-0.94222500
H	-1.73334400	6.22047800	-1.05775700
H	-1.53047700	4.84603200	-2.17612700
C	0.62161900	5.00186500	-0.44224400
H	0.63872200	6.09754900	-0.33529300
H	1.36703600	4.58571100	0.25253100
H	0.94073700	4.76000300	-1.46742400
C	-1.17404400	4.87556300	1.29472500
H	-0.48628300	4.42094100	2.02569200
H	-1.12188000	5.97011200	1.41151100
H	-2.19750000	4.55927300	1.54767200
C	-7.11821700	0.46247300	0.88953400
C	-7.52063500	1.40292200	-0.26512000
H	-8.50294500	1.85692900	-0.05740000
H	-6.79538300	2.21986500	-0.39912000
H	-7.59331200	0.85154900	-1.21624400
C	-7.00822900	1.27245300	2.19776700
H	-7.98049100	1.73007600	2.44181500
H	-6.71439600	0.62485100	3.03931000
H	-6.26700900	2.08197700	2.11583600
C	-8.21021100	-0.60251400	1.05978100
H	-8.34489300	-1.20053600	0.14503000
H	-7.98609800	-1.29015400	1.89005900
H	-9.17081200	-0.11289600	1.28266200
Ni	-1.34775000	-1.81375100	-0.41115700
Cl	-1.41073100	-4.00705200	0.35717500
C	4.00284600	0.00037900	0.44749000
C	3.06294000	-0.91171200	0.63729900
C	2.09742500	-1.75554600	0.92744800
C	3.84400800	1.02419600	-0.63051100
C	3.36848000	0.66323900	-1.90243300

C	4.11939200	2.37897300	-0.36828100
C	3.16563000	1.63646300	-2.88462000
H	3.16326600	-0.38750800	-2.11565500
C	3.91601800	3.34839000	-1.35241900
H	4.48363200	2.67635900	0.61725200
C	3.43677100	2.98122300	-2.61489300
H	2.79329200	1.33945100	-3.86850200
H	4.12387600	4.39774400	-1.12914700
H	3.27633600	3.74117800	-3.38363200
C	5.21100100	0.02959900	1.32435200
C	6.44322700	0.50142700	0.83765300
C	5.14816400	-0.43996500	2.64936900
C	7.57914700	0.49845100	1.65125900
H	6.51630500	0.86502700	-0.18892000
C	6.28294300	-0.44177600	3.46111800
H	4.19439100	-0.79762400	3.04431500
C	7.50475100	0.02818100	2.96575600
H	8.52901400	0.86436500	1.25300000
H	6.21194000	-0.80521200	4.48953900
H	8.39285700	0.03022400	3.60262800
C	1.00792200	-1.39797800	1.91124300
H	0.02313300	-1.44565800	1.41115200
H	0.98487400	-2.12690800	2.73896800
H	1.14914600	-0.38921000	2.32205600
C	1.99337400	-3.11968300	0.28077600
H	2.93143400	-3.69204100	0.31253100
H	1.16690500	-3.70655800	0.70765100
S	1.59918500	-2.98292400	-1.48736900
O	2.80971900	-2.59066900	-2.23495100
O	0.40659000	-2.09504600	-1.64473800
C	1.12284300	-4.63061400	-1.97262700
H	1.96213000	-5.31106000	-1.77492000
H	0.22139200	-4.89086700	-1.39910400
H	0.91380000	-4.57199800	-3.05050900

complex G

Imaginary Frequency: no

Electronic Energy (EE): -4022.497267

Thermal Correction to Enthalpy: 0.744840

Thermal Correction to Free Energy: 0.618197

EE + Thermal Enthalpy Correction: -4021.752426

EE + Thermal Free Energy Correction: -4021.879070

0 2

C	-2.03699600	-0.18539500	-0.83941200

C	-2.09663800	2.10272900	-1.19974100
C	-3.44417400	2.16750900	-0.85870500
C	-4.13229000	0.99968100	-0.49536300
C	-3.38991800	-0.19029700	-0.50654800
C	-1.17512600	-1.38562600	-0.79630300
C	1.00217200	-2.15109700	-1.01147000
C	0.62600700	-3.44888500	-0.67156200
C	-0.71684800	-3.73681400	-0.38894200
C	-1.61927900	-2.66359000	-0.46063000
H	-1.54940900	3.00364700	-1.48113600
H	-3.93588800	3.13870100	-0.88016300
H	-3.86367500	-1.13008100	-0.22697700
H	2.04558700	-1.89872400	-1.20208800
H	1.39741500	-4.21588000	-0.62538400
H	-2.67384700	-2.82198900	-0.24148800
N	0.12801100	-1.14310000	-1.07313100
N	-1.39326300	0.96090900	-1.18516600
C	-5.60476600	0.98217300	-0.07743300
C	-6.38598400	0.03700800	-1.01268900
H	-6.00206900	-0.99346000	-0.96728100
H	-7.44862400	0.01219900	-0.72243200
H	-6.32528200	0.37803800	-2.05858700
C	-6.23665700	2.37893600	-0.14655900
H	-7.29268700	2.32173700	0.15940200
H	-5.73270400	3.08989400	0.52647500
H	-6.20608200	2.79124600	-1.16718400
C	-5.69975500	0.46588800	1.37379400
H	-5.14196300	1.12060200	2.06217800
H	-6.75222900	0.44580700	1.69950400
H	-5.29871300	-0.55433200	1.47056900
C	-1.21325800	-5.13025800	0.00802500
C	-2.30850100	-5.57803500	-0.98086700
H	-2.67377900	-6.58162100	-0.70980900
H	-3.17130300	-4.89485200	-0.97428900
H	-1.91694500	-5.62340700	-2.00975200
C	-1.80170600	-5.06107000	1.43274600
H	-2.16868900	-6.05462700	1.73684200
H	-1.03790800	-4.74214000	2.15894900
H	-2.64591500	-4.35783700	1.49318500
C	-0.08207200	-6.16733000	-0.00639400
H	0.36972100	-6.26581900	-1.00581000
H	0.71597600	-5.91282100	0.70819600
H	-0.48176400	-7.15287100	0.27837900
Ni	0.49466800	0.72288100	-1.60376100
Cl	2.35110000	0.98114300	-2.84025800

C	1.15944400	1.71728700	1.30745200
C	2.47687700	1.60557900	1.24144500
C	3.78817000	1.54024100	1.19568900
C	0.31826700	0.59702400	1.82621200
C	0.92031000	-0.64197300	2.13186500
C	-1.06611700	0.72365600	2.02621300
C	0.16868600	-1.69810400	2.63978500
H	1.98283800	-0.78553800	1.92535600
C	-1.81707700	-0.33408500	2.54835200
H	-1.57994000	1.64362000	1.75448600
C	-1.20660400	-1.54798200	2.86358100
H	0.65733800	-2.65216900	2.85321300
H	-2.89265100	-0.20582600	2.68871800
H	-1.79527800	-2.37713600	3.26218000
C	0.56575500	3.01536200	0.85090000
C	0.92107200	3.52657600	-0.41023100
C	-0.30993400	3.76354500	1.65490500
C	0.39608500	4.73826300	-0.86471500
H	1.60421000	2.96061300	-1.04683600
C	-0.84023700	4.97316700	1.19638000
H	-0.56901500	3.40888500	2.65364800
C	-0.49511400	5.46284000	-0.06705200
H	0.67839100	5.11078500	-1.85275600
H	-1.52196500	5.53940600	1.83613400
H	-0.91401600	6.40663300	-0.42488500
C	4.67635800	1.87919000	2.36992100
H	4.09146200	2.17602200	3.25133300
H	5.29865500	1.00680500	2.62716500
H	5.36407500	2.70048200	2.10503900
C	4.46224500	1.07099900	-0.06508000
H	3.79861000	1.12849500	-0.94103400
H	5.40129200	1.61079900	-0.26111200
S	4.93278100	-0.68373600	0.05171500
O	3.73627400	-1.47153300	0.42539900
O	6.13974100	-0.81783300	0.89464900
C	5.35873400	-1.09592900	-1.63911700
H	4.49593800	-0.86942400	-2.28127800
H	6.24028600	-0.51011000	-1.93280000
H	5.58849300	-2.17092600	-1.63863600

complex H

Imaginary Frequency: no

Electronic Energy (EE): -4022.500137

Thermal Correction to Enthalpy: 0.746017

Thermal Correction to Free Energy: 0.617410

EE + Thermal Enthalpy Correction: -4021.754120

EE + Thermal Free Energy Correction: -4021.882727

0 2

C	-2.24632600	0.98661100	-0.00330500
C	-0.46601000	2.46287400	0.10710900
C	-1.21330100	3.49101800	-0.46532100
C	-2.54376700	3.25573000	-0.83560900
C	-3.04826200	1.96892700	-0.58821300
C	-2.73919000	-0.38681000	0.29628400
C	-2.23912000	-2.43726200	1.24954800
C	-3.49855300	-2.94862900	0.93832400
C	-4.42730900	-2.13946800	0.26939200
C	-4.01776800	-0.83343800	-0.04564000
H	0.57510600	2.62336800	0.39231300
H	-0.73734000	4.45876900	-0.61354700
H	-4.07798400	1.73409800	-0.85094400
H	-1.49232500	-3.03079400	1.78449100
H	-3.73297200	-3.97244300	1.22521300
H	-4.70181900	-0.16430600	-0.56437300
N	-1.86996300	-1.19637200	0.93376900
N	-0.96312600	1.24528500	0.33031700
C	-3.43647800	4.31718900	-1.48377100
C	-3.86610400	3.81807000	-2.87898700
H	-4.43570700	2.87816800	-2.82025200
H	-4.50722400	4.57025800	-3.36584400
H	-2.98950500	3.64680300	-3.52392200
C	-2.70916200	5.65936000	-1.64128600
H	-3.38693900	6.39221200	-2.10539900
H	-2.38997400	6.06806100	-0.66994000
H	-1.82141400	5.57134600	-2.28658700
C	-4.68539600	4.53000800	-0.60393000
H	-4.40425000	4.87240900	0.40476800
H	-5.33611200	5.29554000	-1.05582200
H	-5.27625300	3.60753000	-0.49937100
C	-5.83103900	-2.61450900	-0.11676500
C	-5.98896300	-2.51204300	-1.64778000
H	-6.99320900	-2.85346800	-1.94573200
H	-5.86671800	-1.47794800	-2.00401300
H	-5.24669800	-3.14153300	-2.16413300
C	-6.87238600	-1.70842600	0.57206100
H	-7.89008900	-2.03721400	0.30743000
H	-6.77211700	-1.75482600	1.66820400
H	-6.76854600	-0.65695500	0.26401700
C	-6.08251800	-4.06724700	0.30923200

H	-5.37595500	-4.76260200	-0.17003600
H	-6.00330900	-4.19359900	1.40015500
H	-7.09919400	-4.36693500	0.01177600
Ni	0.17012700	-0.45745300	1.12891900
Cl	0.75355600	-1.82489200	3.02328800
C	2.88191300	0.67038200	0.25313300
C	1.89783100	-0.23080200	0.13075300
C	1.23839800	-1.21770300	-0.57291200
C	4.06566400	0.65078900	-0.65384800
C	5.32754000	1.06899100	-0.18984100
C	3.96777300	0.18316800	-1.97609400
C	6.45106000	1.01731100	-1.01826400
H	5.43352600	1.42621800	0.83625400
C	5.08812800	0.13666400	-2.80571200
H	3.00512600	-0.14859200	-2.35984700
C	6.33715300	0.55298000	-2.33241100
H	7.42185200	1.33977300	-0.63247500
H	4.98444700	-0.23164600	-3.82967100
H	7.21494500	0.51556500	-2.98261900
C	2.79682700	1.73099100	1.29872100
C	2.29301100	1.44695000	2.58081400
C	3.13158200	3.06378600	0.99174500
C	2.10671900	2.46766800	3.51653900
H	2.05256000	0.41425200	2.84294200
C	2.94387800	4.08286500	1.92813600
H	3.52053500	3.30749700	0.00096100
C	2.42545300	3.79060500	3.19451200
H	1.71357400	2.22404000	4.50730400
H	3.19726700	5.11295100	1.66401700
H	2.27721500	4.58856200	3.92652300
C	0.50802600	-0.92480500	-1.86764200
H	1.06568900	-1.36861100	-2.70853700
H	-0.49599900	-1.37968800	-1.86261700
H	0.40335100	0.15482100	-2.03752600
C	1.46941400	-2.67321700	-0.25493500
H	1.61529800	-2.84705800	0.82012100
H	0.65463600	-3.30883900	-0.63246500
S	2.99729700	-3.28396600	-1.04742900
O	4.15750100	-2.78355400	-0.28519300
O	2.93466500	-3.02591600	-2.50303500
C	2.90231300	-5.05968000	-0.80037700
H	2.84784100	-5.26954400	0.27642800
H	2.02575500	-5.45307500	-1.33200100
H	3.83028900	-5.46728400	-1.22675600

complex I

Imaginary Frequency: no

Electronic Energy (EE): -4022.490396

Thermal Correction to Enthalpy: 0.744303

Thermal Correction to Free Energy: 0. 619246

EE + Thermal Enthalpy Correction: -4021.746093

EE + Thermal Free Energy Correction: -4021.871150

0 2

C	-2.28550100	0.96403100	-1.22514700
C	-0.49861500	2.16045100	-2.07625600
C	-1.26854100	3.30629100	-2.23250800
C	-2.61841500	3.28835000	-1.84274400
C	-3.12033200	2.08392100	-1.34353300
C	-2.77958200	-0.36190800	-0.77397300
C	-2.24268000	-2.58386700	-0.44433600
C	-3.56619600	-2.92276900	-0.17581100
C	-4.54353900	-1.91804900	-0.17565400
C	-4.12846300	-0.62255500	-0.50745300
H	0.55419700	2.13901200	-2.36406500
H	-0.81895200	4.20944300	-2.65108900
H	-4.16506400	2.02593600	-1.03997500
H	-1.46082900	-3.34448800	-0.47682800
H	-3.83007300	-3.96144400	0.03360500
H	-4.86808800	0.17560200	-0.56430800
N	-1.85128200	-1.33356000	-0.69346200
N	-0.98357000	1.01994900	-1.56596100
Ni	0.24120200	-0.63815200	-0.82669800
Cl	0.66653600	-2.47023800	-2.36675700
C	0.56390800	0.00174700	1.32129600
C	1.24061700	-1.19992400	1.00511600
C	2.78424800	-1.37511400	0.90890700
C	-0.83294300	0.10976900	1.85595500
C	-1.50056800	1.35180400	1.73735200
C	-1.54137900	-0.92305600	2.50727500
C	-2.79791500	1.54359900	2.20708100
H	-0.99414900	2.18204400	1.24606000
C	-2.84421400	-0.73333100	2.97511200
H	-1.08010700	-1.89093800	2.66835100
C	-3.48867800	0.49730000	2.82620200
H	-3.27689600	2.51722600	2.07422600
H	-3.35723200	-1.56374000	3.46775200
H	-4.50916900	0.63959900	3.19014400
C	1.34089700	1.26751500	1.60914800
C	1.74794700	1.47784400	2.94055200

C	1.61457500	2.27380000	0.67480600
C	2.44438600	2.62924300	3.31262700
H	1.50817800	0.72469800	3.69456600
C	2.31064200	3.43042500	1.04162700
H	1.30376200	2.13820400	-0.35402700
C	2.73624700	3.61144800	2.35926500
H	2.75788100	2.76160300	4.35159100
H	2.52068500	4.19215900	0.28616700
H	3.28292400	4.51353700	2.64579100
C	3.36784200	-1.47254900	2.34282600
H	4.42776300	-1.76998200	2.29870900
H	3.30613300	-0.51101900	2.86829500
H	2.83425700	-2.22393100	2.94126000
C	3.65807700	-0.31439400	0.22786400
H	3.62554800	0.65189700	0.74592200
H	4.70746900	-0.64664900	0.23083700
S	3.41471100	0.21845400	-1.48368100
O	4.33207500	1.35714800	-1.67595200
O	1.96712200	0.47966300	-1.74154800
C	3.91077800	-1.09037300	-2.59551100
H	4.84971600	-1.53548200	-2.24140600
H	3.08387100	-1.81164600	-2.67168000
H	4.06599700	-0.57822100	-3.55628800
C	-5.97283300	-2.20540200	0.18056600
H	-6.14063300	-1.99229700	1.25100100
H	-6.66604200	-1.56991200	-0.39074500
H	-6.22860500	-3.26102100	0.00826900
C	-3.47704500	4.51390300	-1.96168600
H	-4.52001400	4.30759400	-1.68258400
H	-3.09579300	5.31670400	-1.30852900
H	-3.45936700	4.90545400	-2.99179900
C	0.74361100	-2.60476900	1.34909300
H	0.71360300	-2.71250700	2.44689800
H	-0.27322200	-2.79425400	1.00104800
C	2.92148000	-2.75264400	0.22766200
H	2.78379600	-2.63954200	-0.85114600
H	3.91500000	-3.19003700	0.41107600
C	1.76038700	-3.60415100	0.76006300
H	1.31285000	-4.17690400	-0.06342100
H	2.09162000	-4.31836800	1.52890900

References

[1] Ota, K.; Lee, S. I. Tang, J. M.; Takachi, M.; Nakai, H.; Morimoto, T.; Sakurai, H.; Kataoka, K.; Chatani, N. *J. Am. Chem. Soc*. **2009,** *131*, 15203–15211.

[2] Zhao, M.-M.; He, W.; Zou, L.-H.; Wang, D.-W.; Sun, T.-Y.; Xia, X.-F. *Org. Chem. Front*. **2021,** *8*, 643-652.

[3] Zheng, L.; Liang, Y.-M. *J. Org. Chem*. **2017,** *82*, 7000–7007.

[4] Shibata, T.; Toshida, N.; Takagi, K. *Org. Lett*. **2002,** *4*, 1619–1621.

[5] Ohmura, T.; Sasaki, I.; Suginome, M. *Org. Lett*. **2019,** *21*, 1649–1653.

[6] Bao, H.-L.; Cao, J. *Org. Lett*. **2021**, *23*, 3184–3189.

[7] Xue, D; Yan, Y.-G. *Org. Lett.* **2024**, *26*, 1370–1375

[8] Frisch, M. J.; Trucks, G. W.; Schlegel, H. B.; Scuseria, G. E.; Robb, M. A.; Cheeseman, J. R.; Scalmani, G.; Barone, V.; Mennucci, B.; Petersson, G. A.; Nakatsuji, H.; Caricato, M.; Li, X.; Hratchian, H. P.; Izmaylov, A. F.; Bloino, J.; Zheng, G.; Sonnenberg, J. L.; Hada, M.; Ehara, M.; Toyota, K.; Fukuda, R.; Hasegawa, J.; Ishida, M.; Nakajima, T.; Honda, Y.; Kitao, O.; Nakai, H.; Vreven, T.; Montgomery, J. A., Jr.; Peralta, J. E.; Ogliaro, F.; Bearpark, M.; Heyd, J. J.; Brothers, E.; Kudin, K. N.; Staroverov, V. N.; Keith, T.; Kobayashi, R.; Normand, J.; Raghavachari, K.; Rendell, A.; Burant, J. C.; Iyengar, S. S.; Tomasi, J.; Cossi, M.; Rega, N.; Millam, J. M.; Klene, M.; Knox, J. E.; Cross, J. B.; Bakken, V.; Adamo, C.; Jaramillo, J.; Gomperts, R.; Stratmann, R. E.; Yazyev, O.; Austin, A. J.; Cammi, R.; Pomelli, C.; Ochterski, J. W.; Martin, R. L.; Morokuma, K.; Zakrzewski, V. G.; Voth, G. A.; Salvador, P.; Dannenberg, J. J.; Dapprich, S.; Daniels, A. D.; Farkas, O.; Foresman, J. B.; Ortiz, J. V.; Cioslowski, J.; Fox, D. J. *Gaussian 16* revision A. 03; Gaussian Inc.: Wallingford, CT, **2016**.

[9] Becke, A. D. *J. Chem. Phys.* **1993**, *98*, 5648-5652.

[10] Lee, C.; Yang, W.; Parr, R. G. *Phys. Rev. B.* **1988**, *37*, 785-789.

[11] Weigend, F.; Ahlrichs, R. *Phys. Chem. Chem. Phys.* **2005**, *7*, 3297–3305.

[12] Grimme, S.; Antony, J.; Ehrlich, S.; Krieg, H. *J. Chem. Phys.* **2010**, *132*, 154104.

[13] Marenich, A. V.; Cramer, C. J.; Truhlar, D. G. *J. Phys. Chem. B* **2009**, *113*, 6378–6396.

[14] Casida, M. E.; Jamorski, C.; Casida, K. C.; Salahub, D. R. *J. Chem. Phys.* **1998**, *108*,4439–4449.

[15] Stratmann, R. E.; Scuseria, G. E.; Frisch, M. J. *J. Chem. Phys.* **1998**, *109*, 8218–8224.

[16] Lu, T.; Chen, F. *J. Comput. Chem.* **2012**, *33*, 580−592.

[17] Lu, T.; Chen, Q. *Chem.-Methods* **2021**, *1*, 231−239.

[18] Humphrey, W.; Dalke, A.; Schulten, K. *J. Mol. Graphics* **1996**, *14*, 33−38.

[19] Hu, D.-D.; Gao, Q.; Dai, J.-C.; Cui, R.; Li, Y.-B.; Li, Y.-M.; Zhou, X.-G.; Bian, K.-J.; Wu, B.-B.; Zhang, K.-F.; Wang, X.-S.; Li, Y. *Sci. China Chem.* **2022**, *65*, 753-761.

NMR spectra of new compounds (^{1}H, ^{13}C and ^{19}F NMR)

1c: ^{1}H NMR (400 Hz, $CDCl_3$)

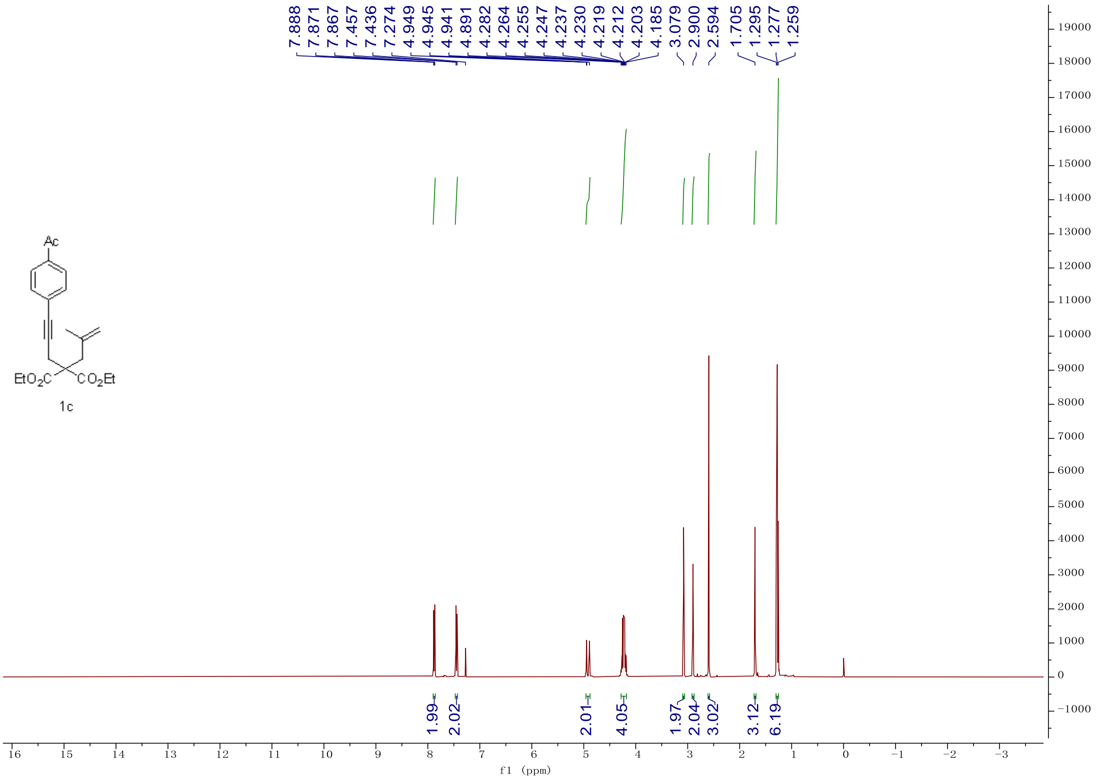

1c: ^{13}C NMR (151 Hz, $CDCl_3$)

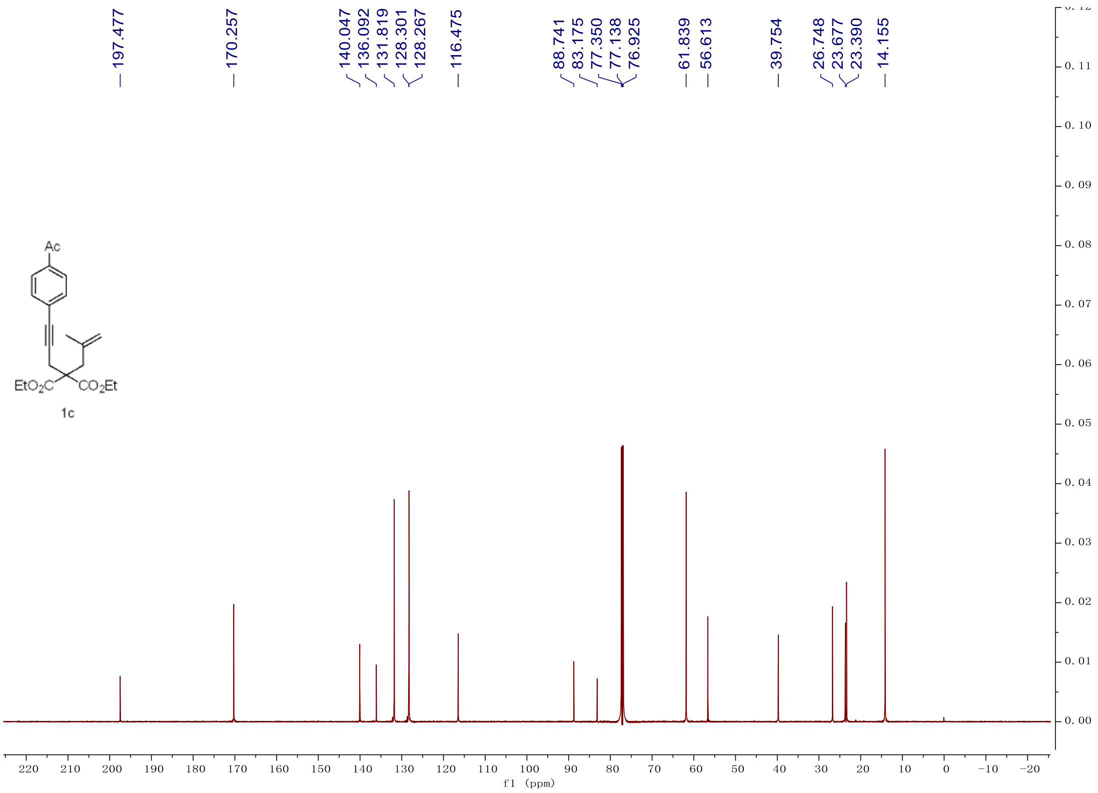

1l: ^{1}H NMR (400 Hz, $CDCl_3$)

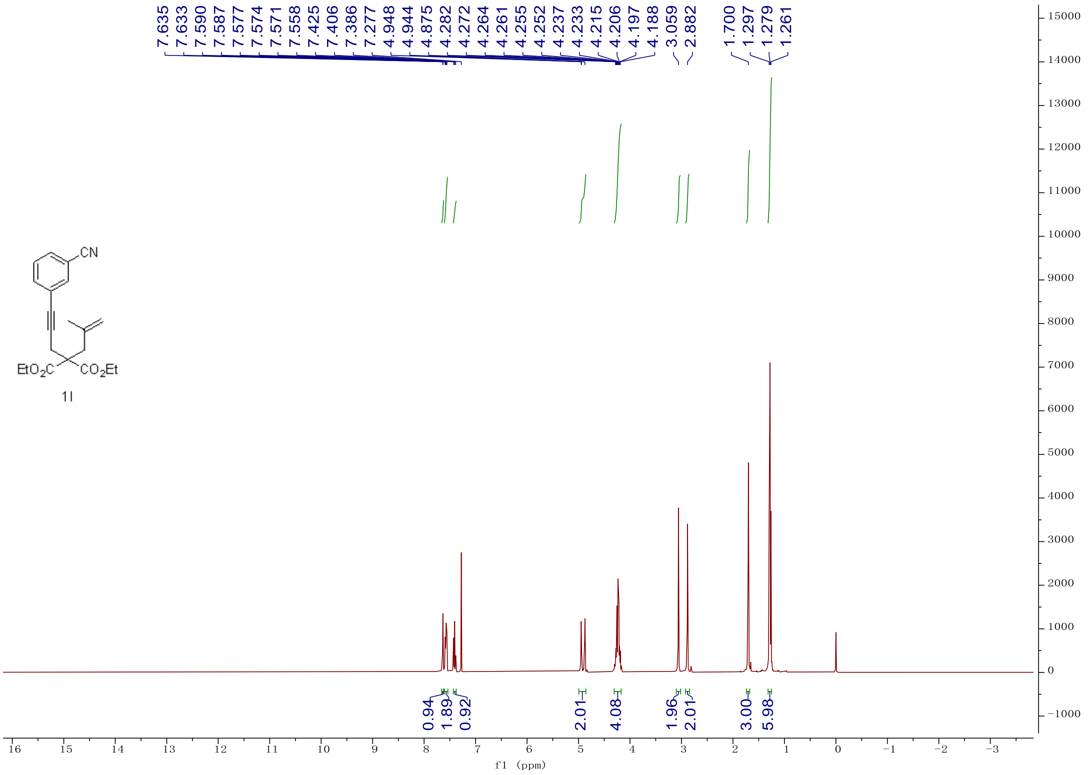

1l: ^{13}C NMR (151 Hz, $CDCl_3$)

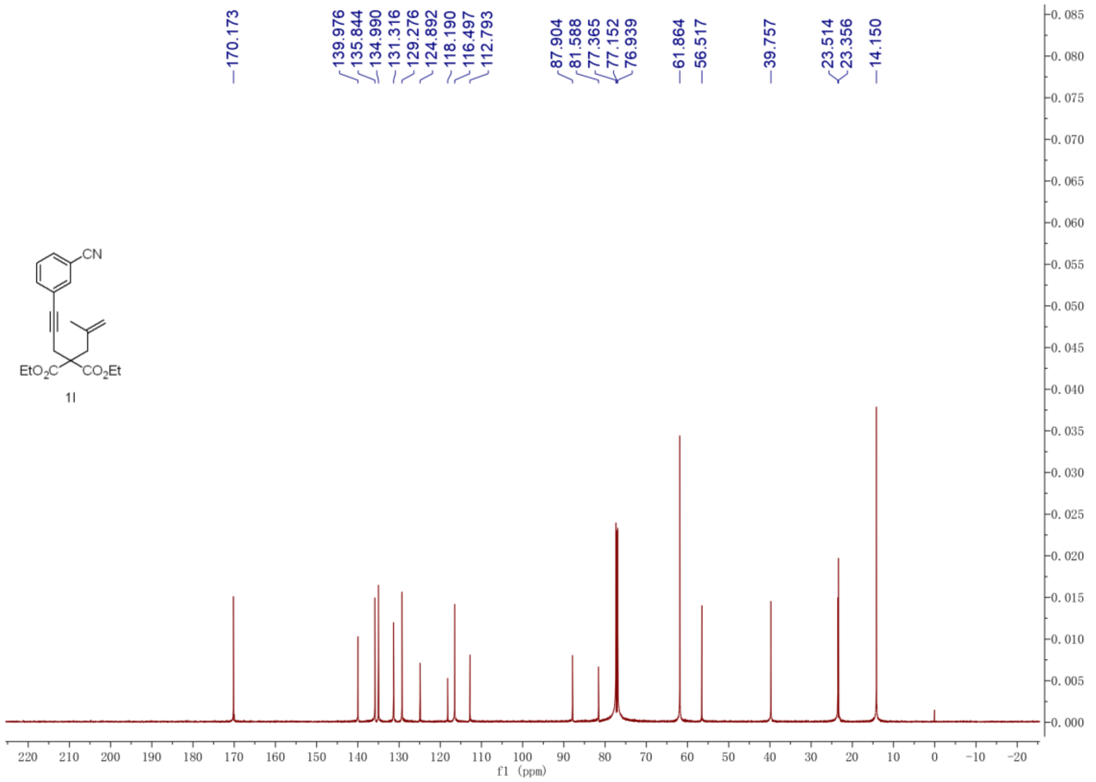

1m: ^{1}H NMR (400 Hz, $CDCl_3$)

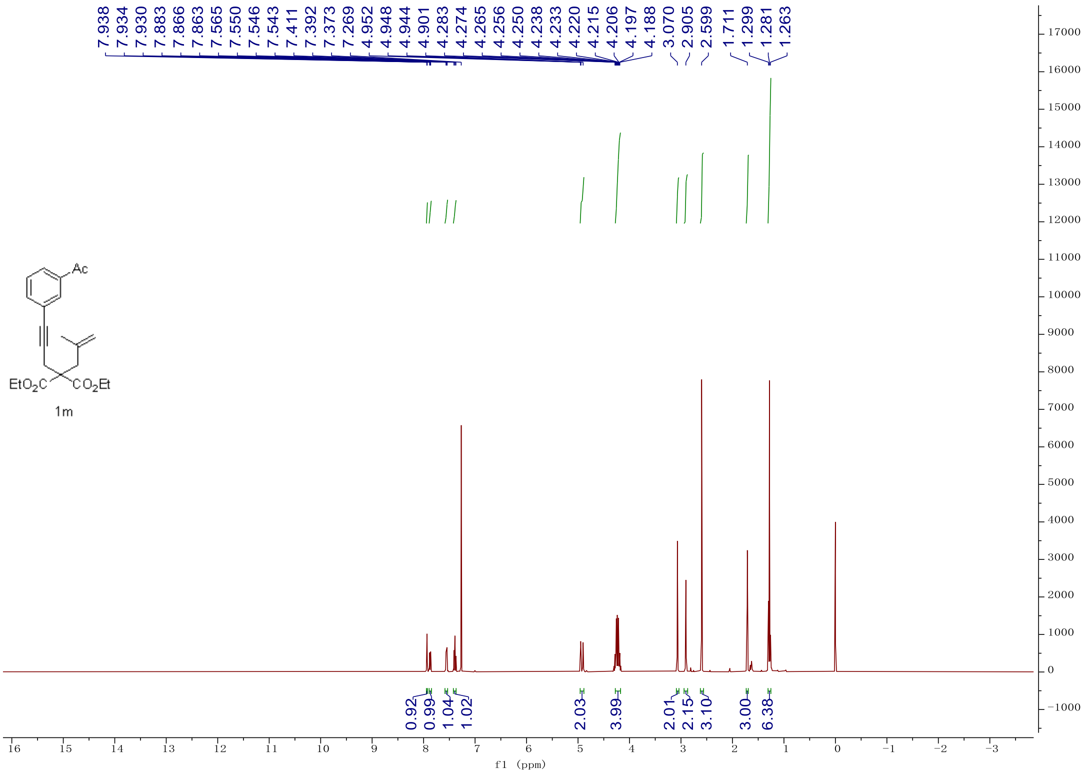

1m: ^{13}C NMR (151 Hz, $CDCl_3$)

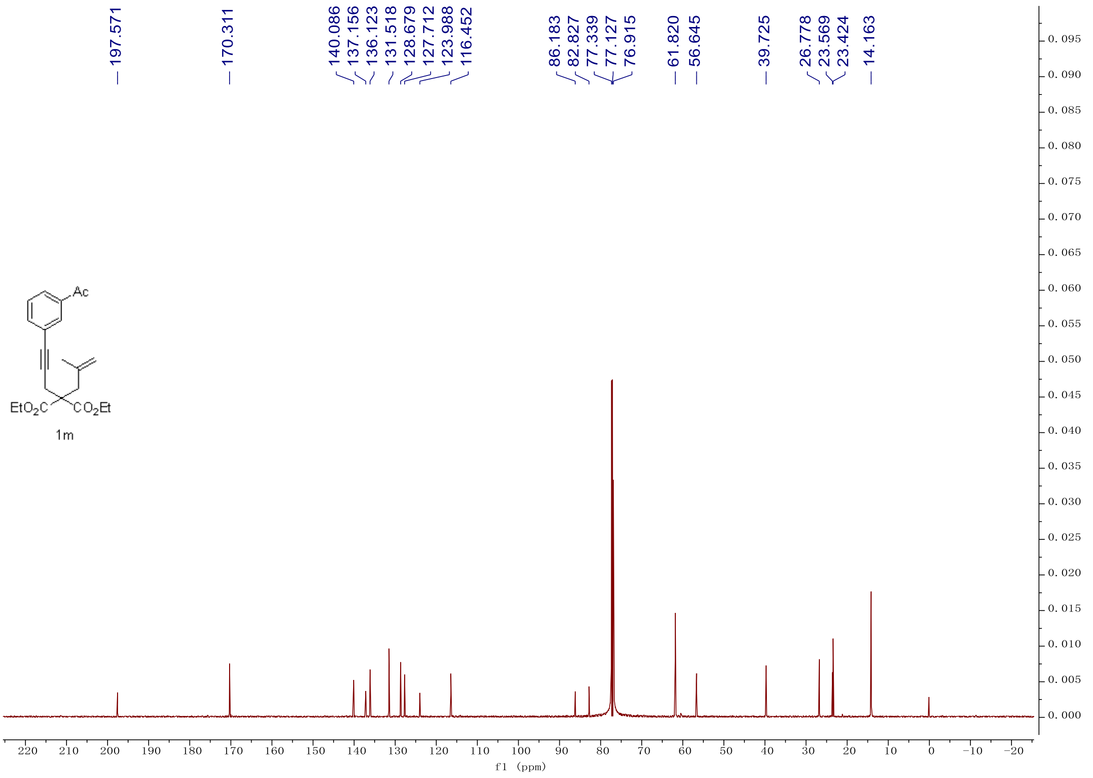

1n: ^{1}H NMR (400 Hz, $CDCl_3$)

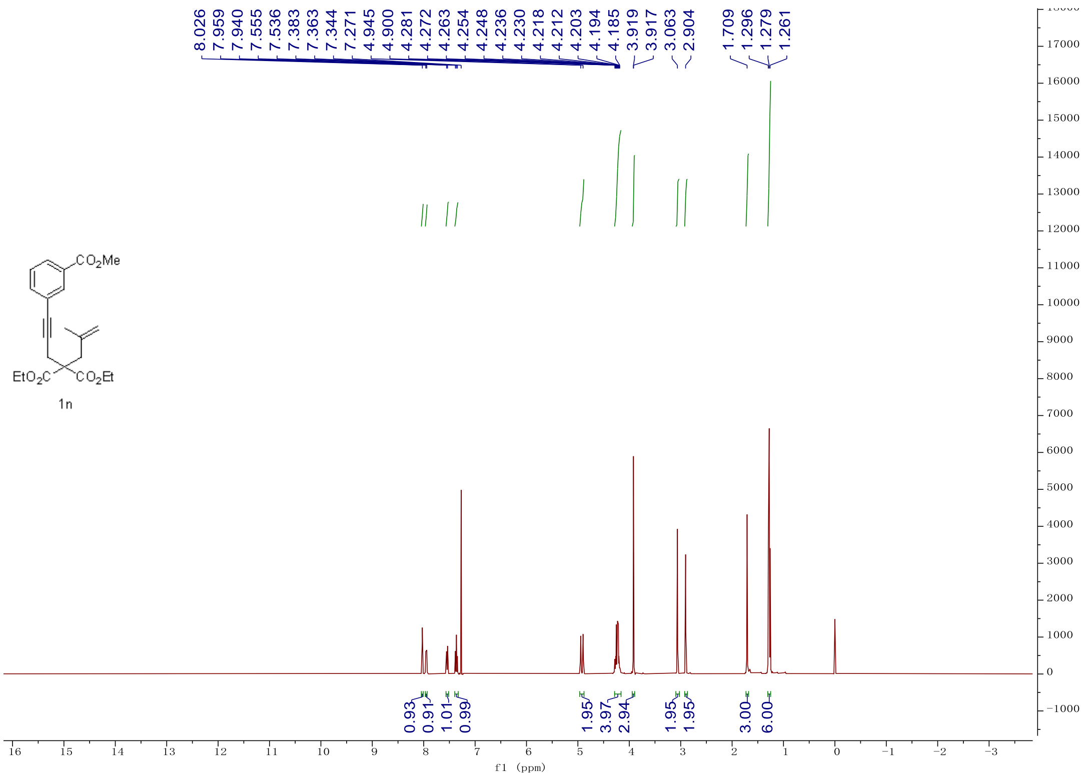

1n: ^{13}C NMR (151 Hz, $CDCl_3$)

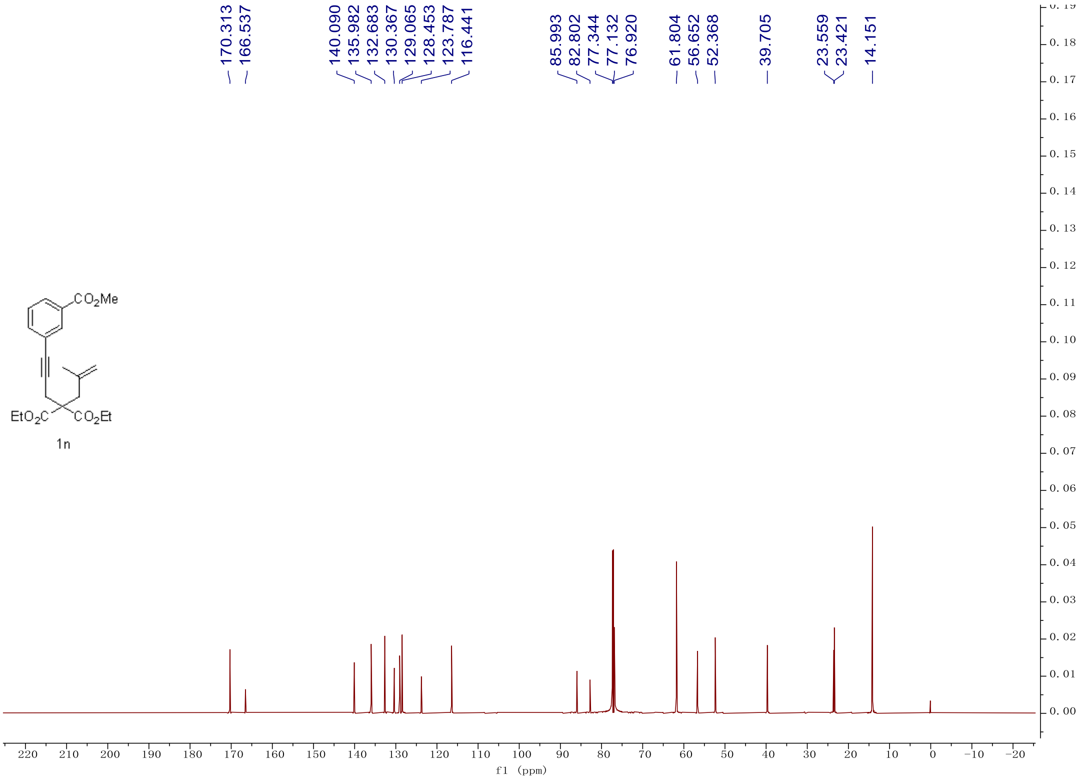

1p: ^{1}H NMR (400 Hz, $CDCl_3$)

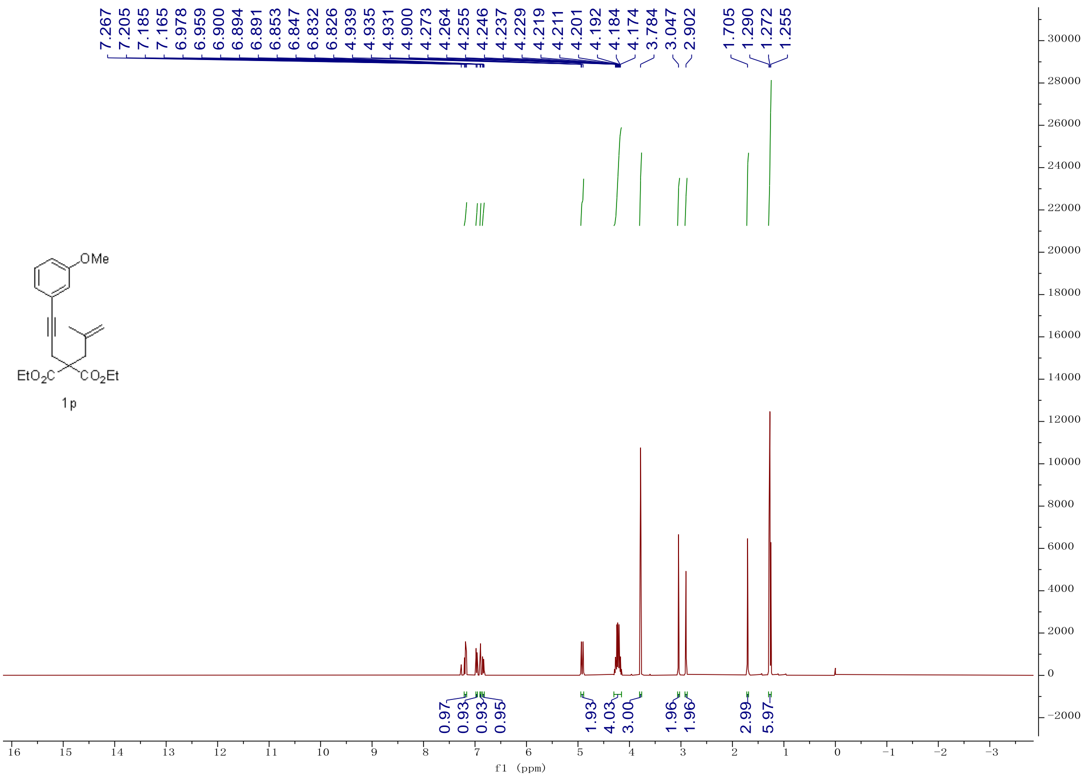

1p: ^{13}C NMR (151 Hz, $CDCl_3$)

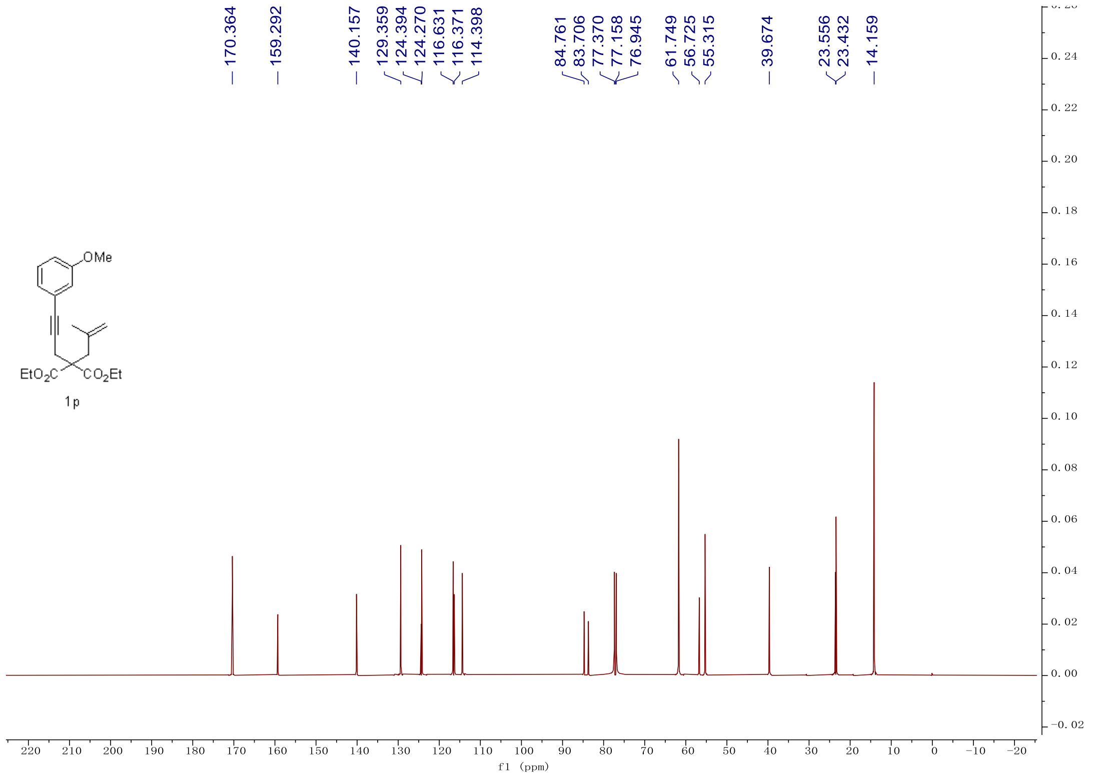

1r: ^{1}H NMR (400 Hz, $CDCl_3$)

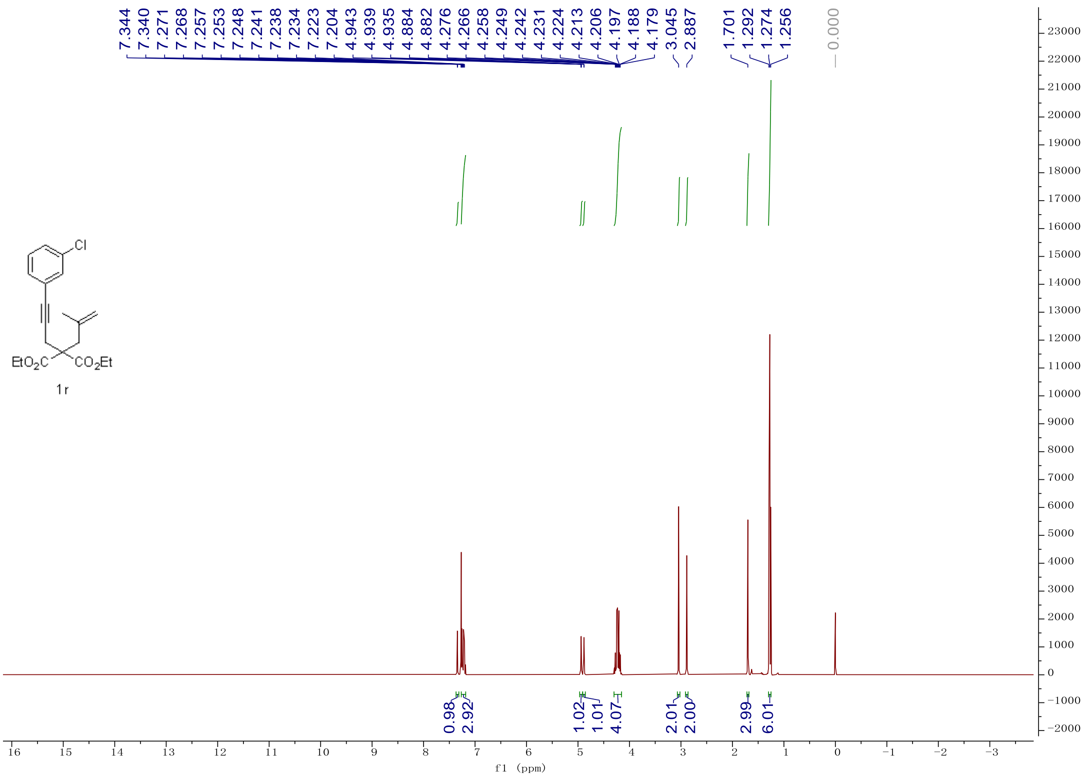

1r: ^{13}C NMR (151 Hz, $CDCl_3$)

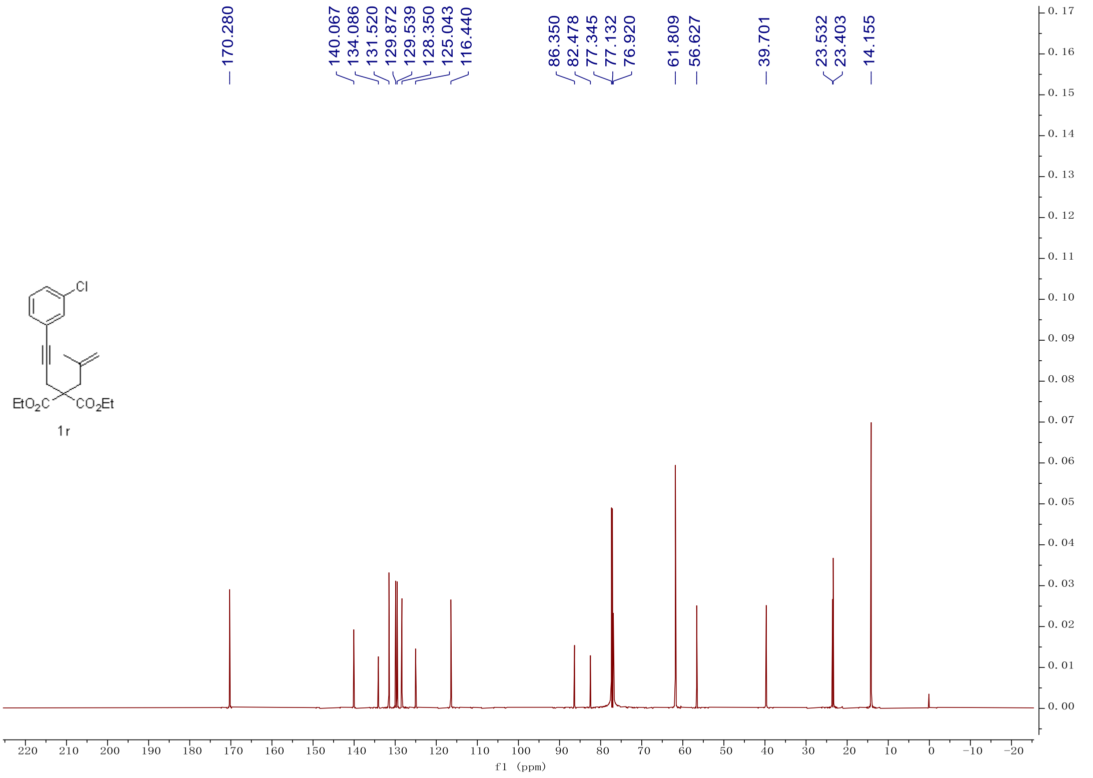

1s: ^{1}H NMR (400 Hz, $CDCl_3$)

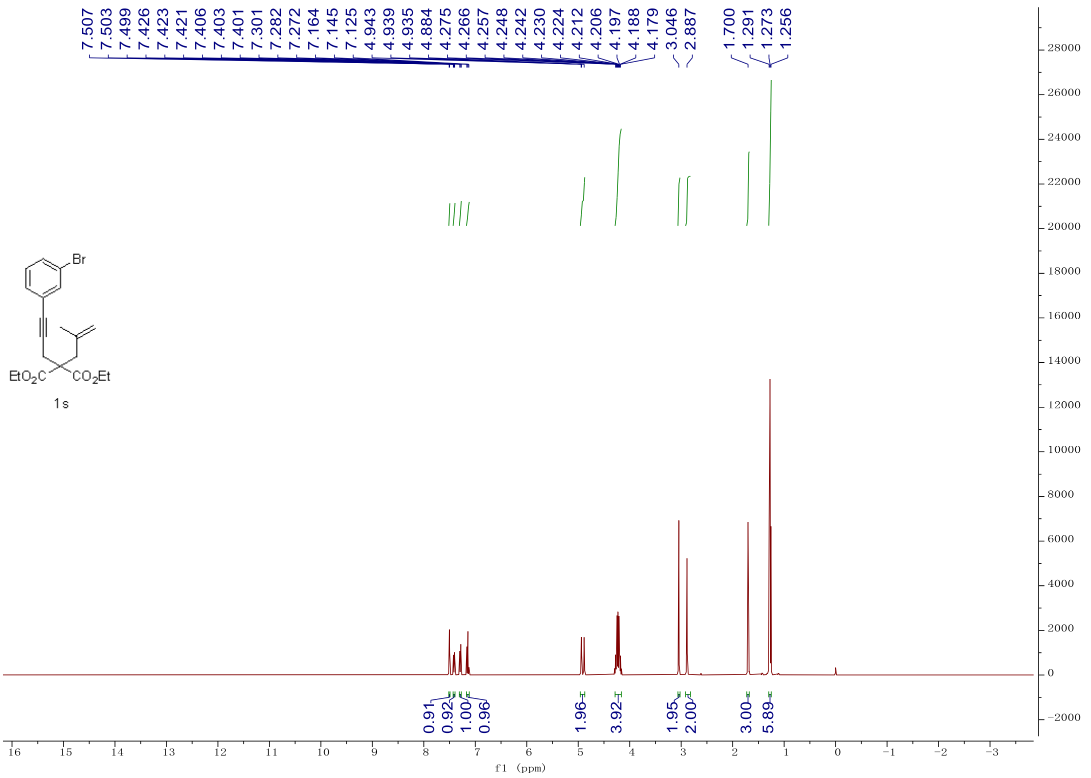

1s: ^{13}C NMR (151 Hz, $CDCl_3$)

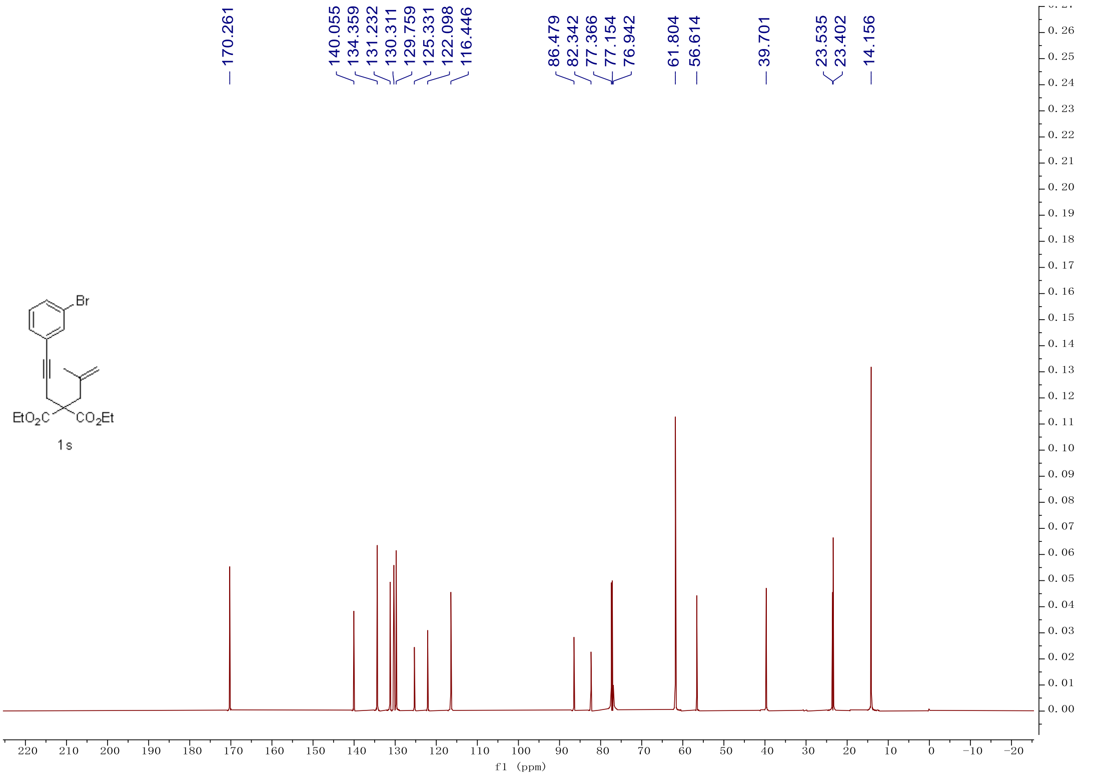

1u: ^{1}H NMR (400 Hz, CDCl$_3$)

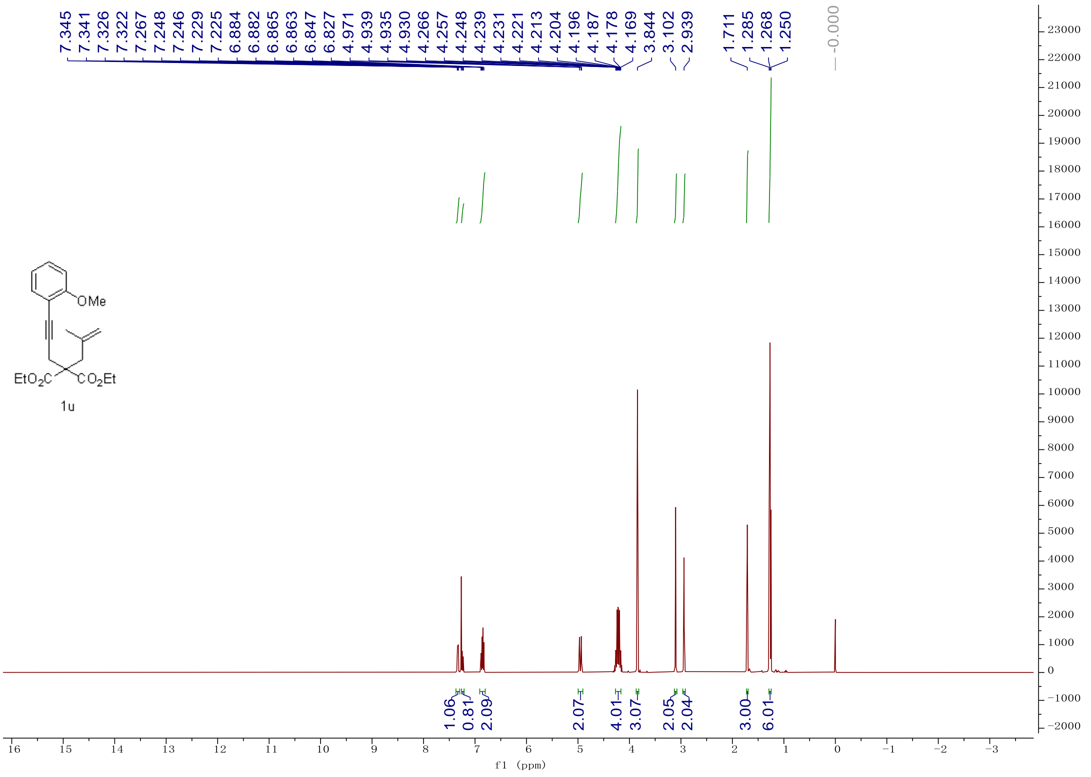

1u: ^{13}C NMR (151 Hz, CDCl$_3$)

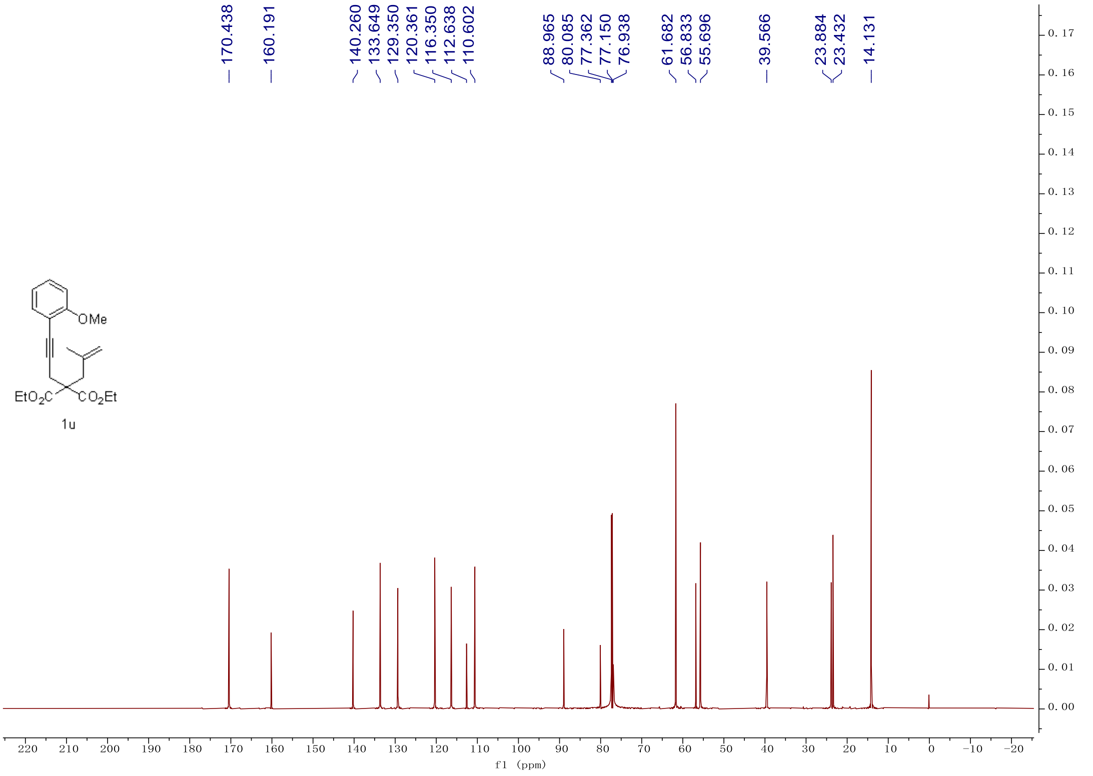

1y: ^{1}H NMR (400 Hz, $CDCl_3$)

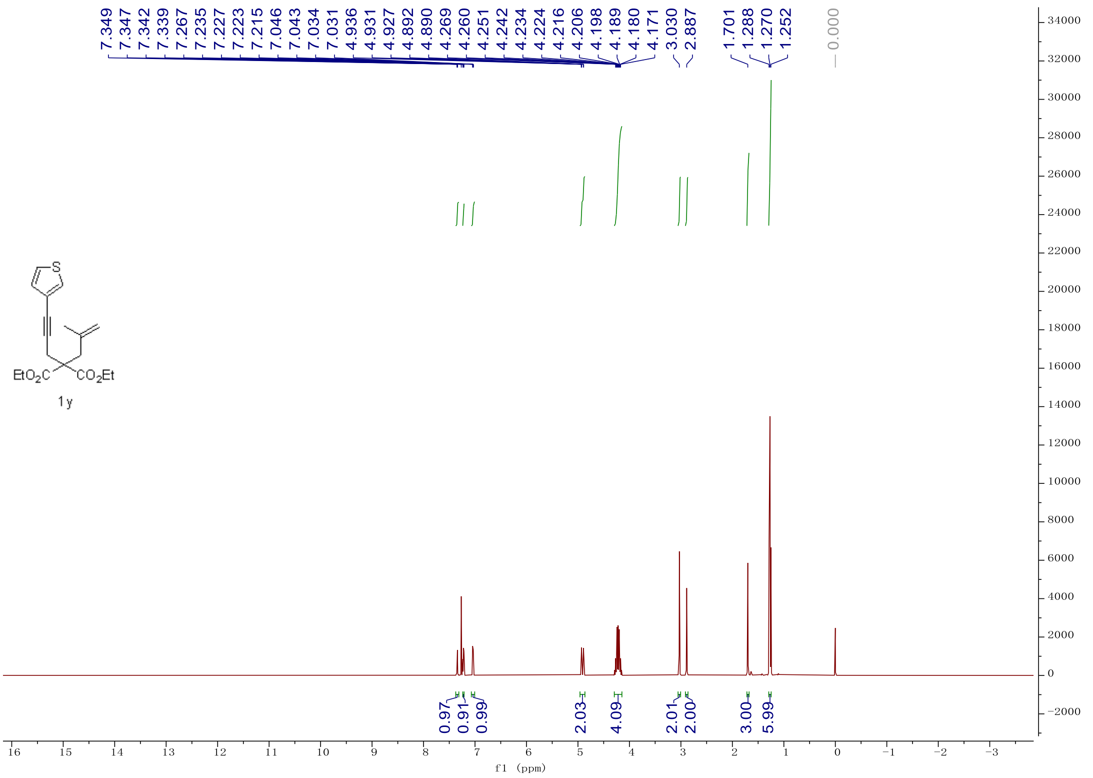

1y: ^{13}C NMR (151 Hz, $CDCl_3$)

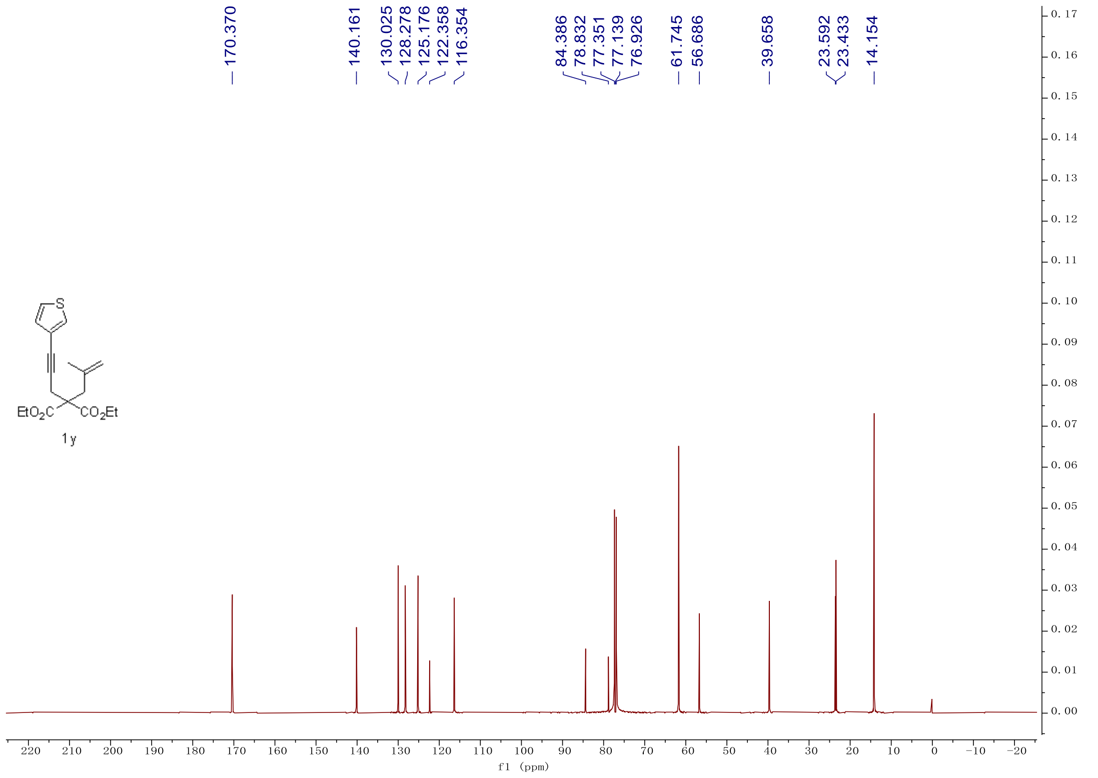

4: ^{1}H NMR (400 Hz, $CDCl_3$)

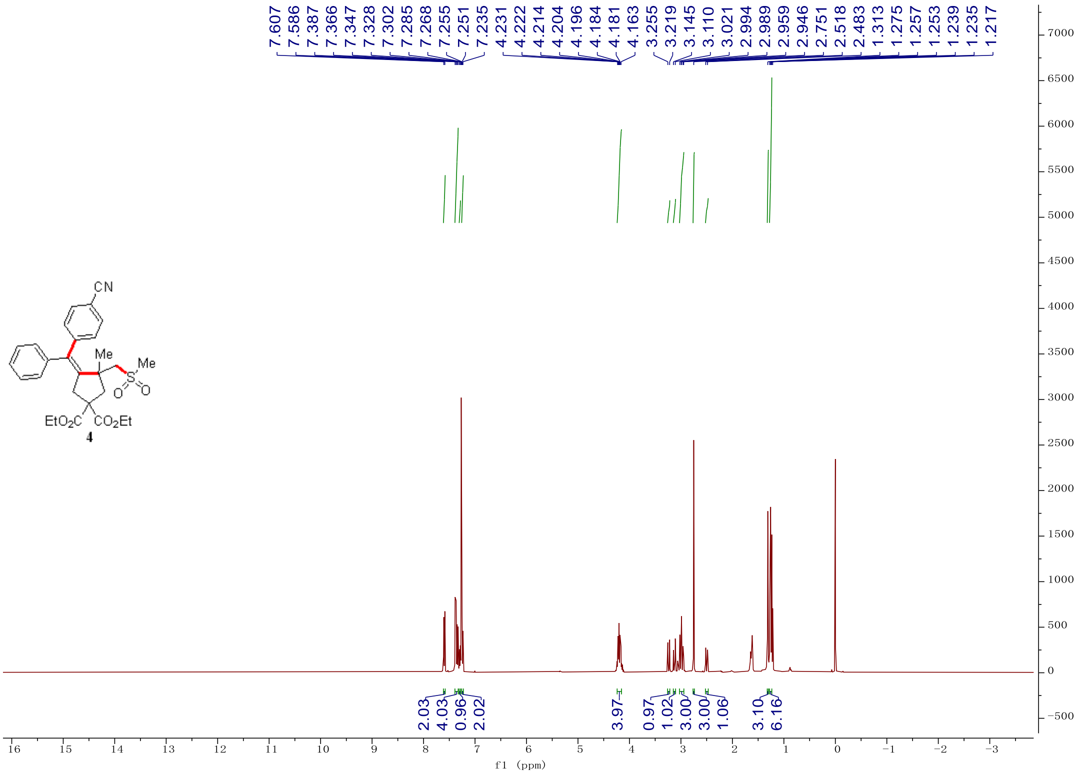

4: ^{13}C NMR (101 Hz, $CDCl_3$)

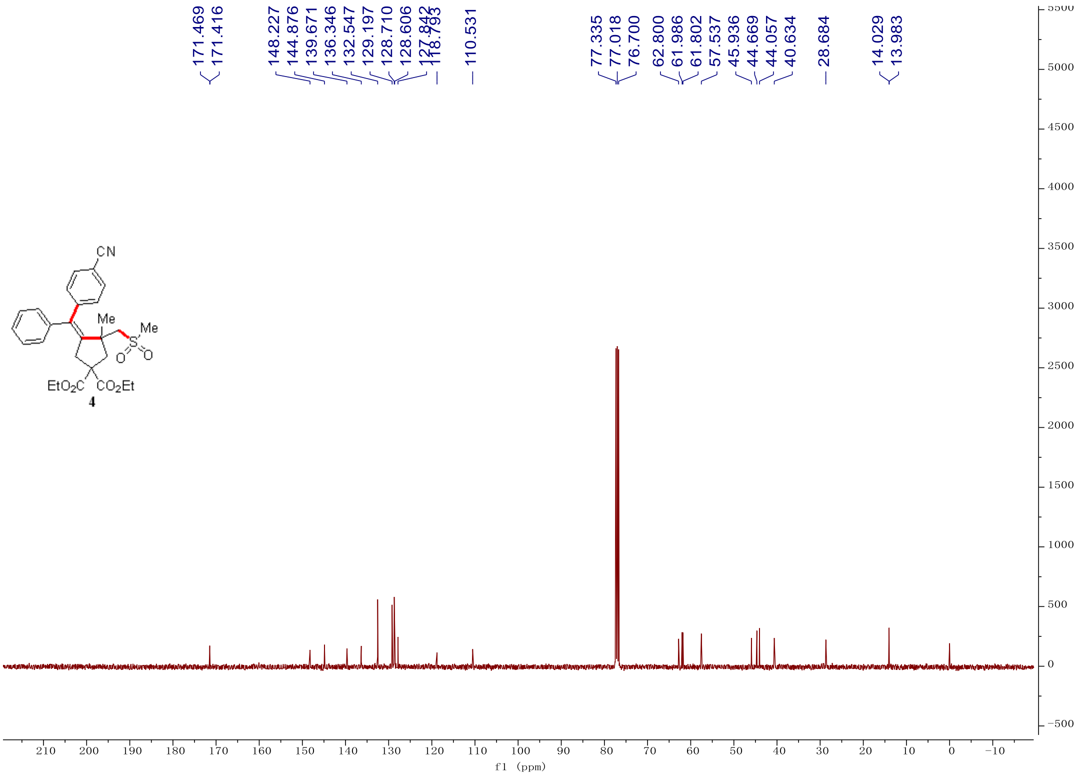

5: ^{1}H NMR (500 Hz, $CDCl_3$)

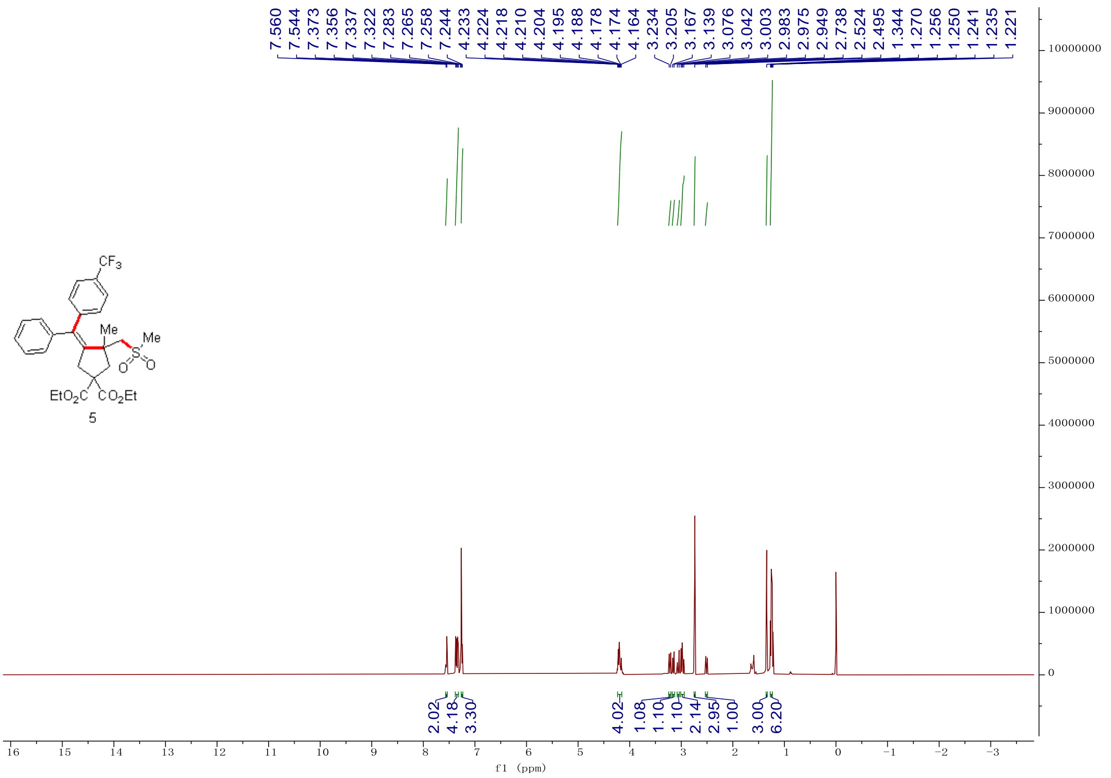

5: ^{13}C NMR (126 Hz, $CDCl_3$)

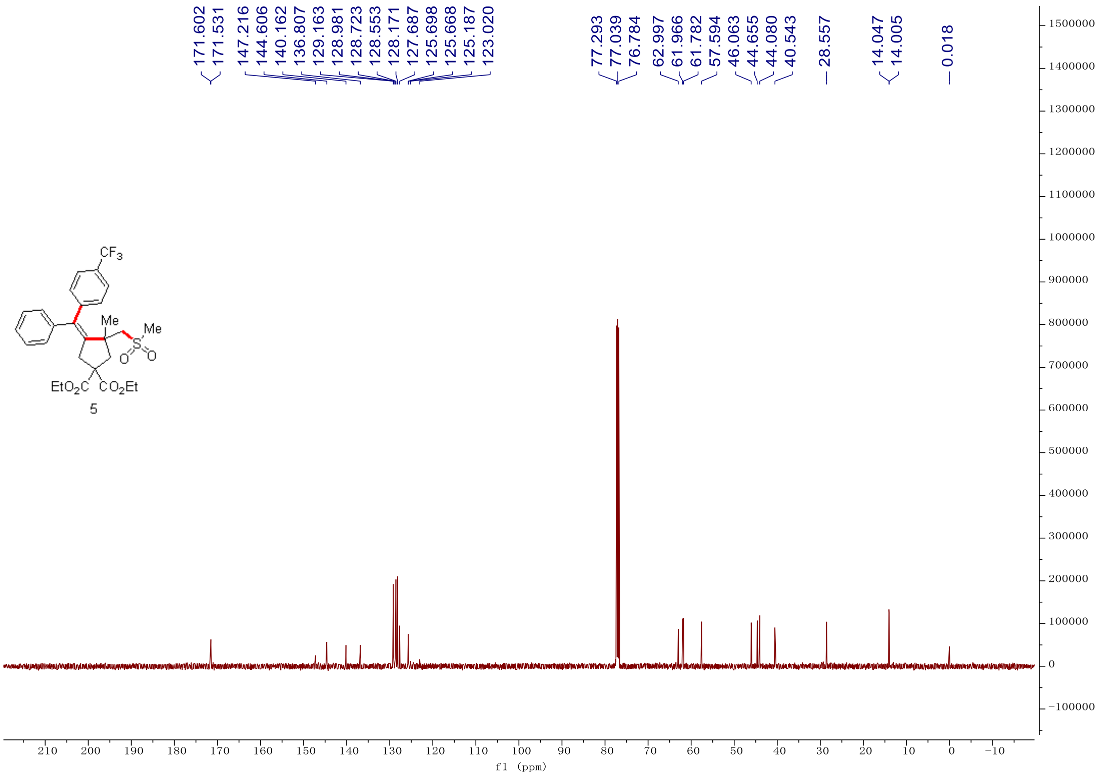

5: ^{19}F NMR (376 Hz, $CDCl_3$)

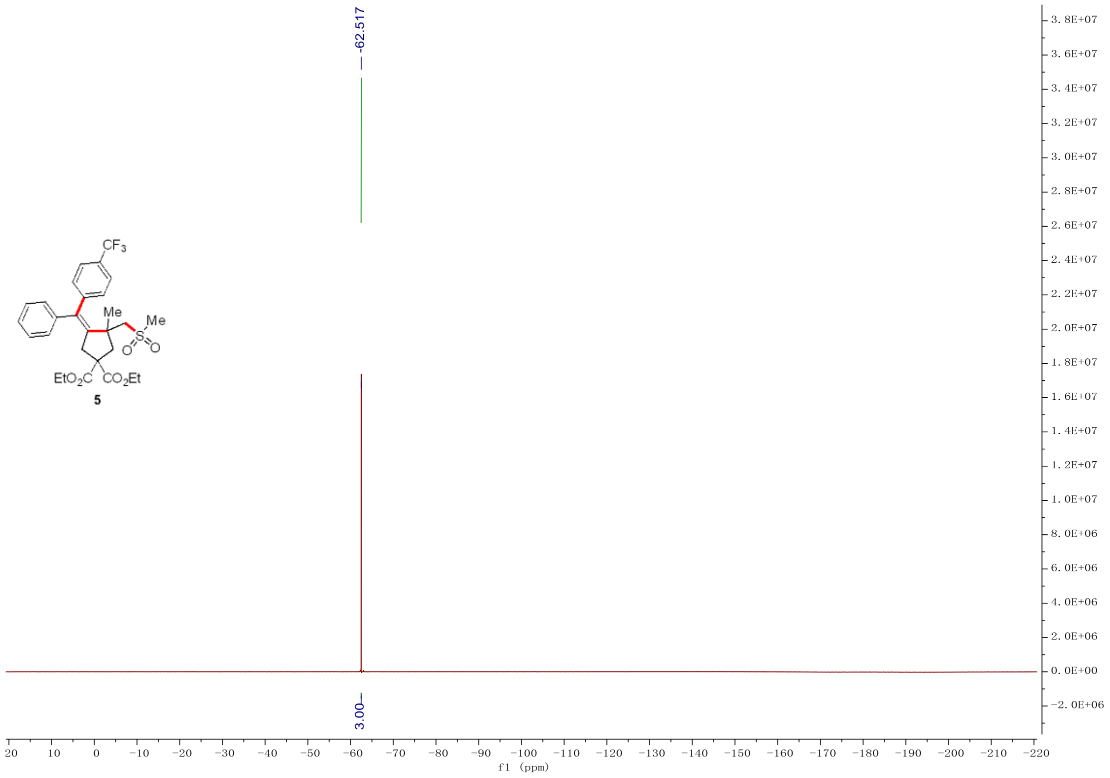

6: ^{1}H NMR (500 Hz, $CDCl_3$)

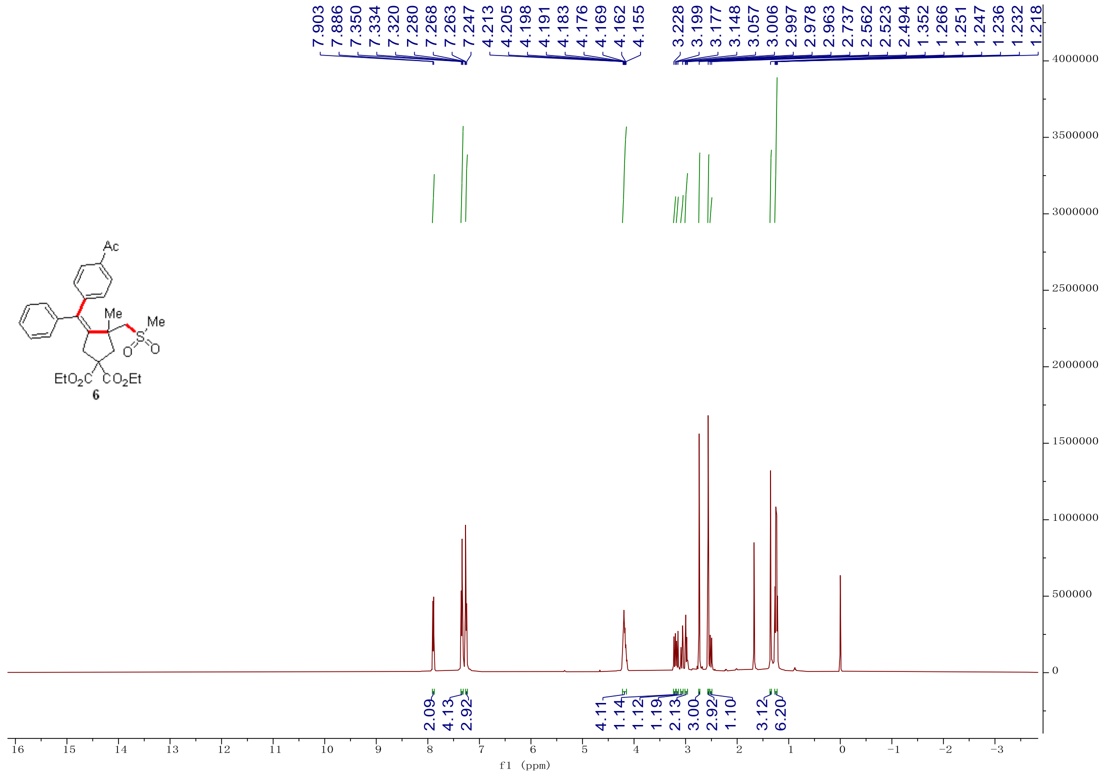

6: ^{13}C NMR (126 Hz, $CDCl_3$)

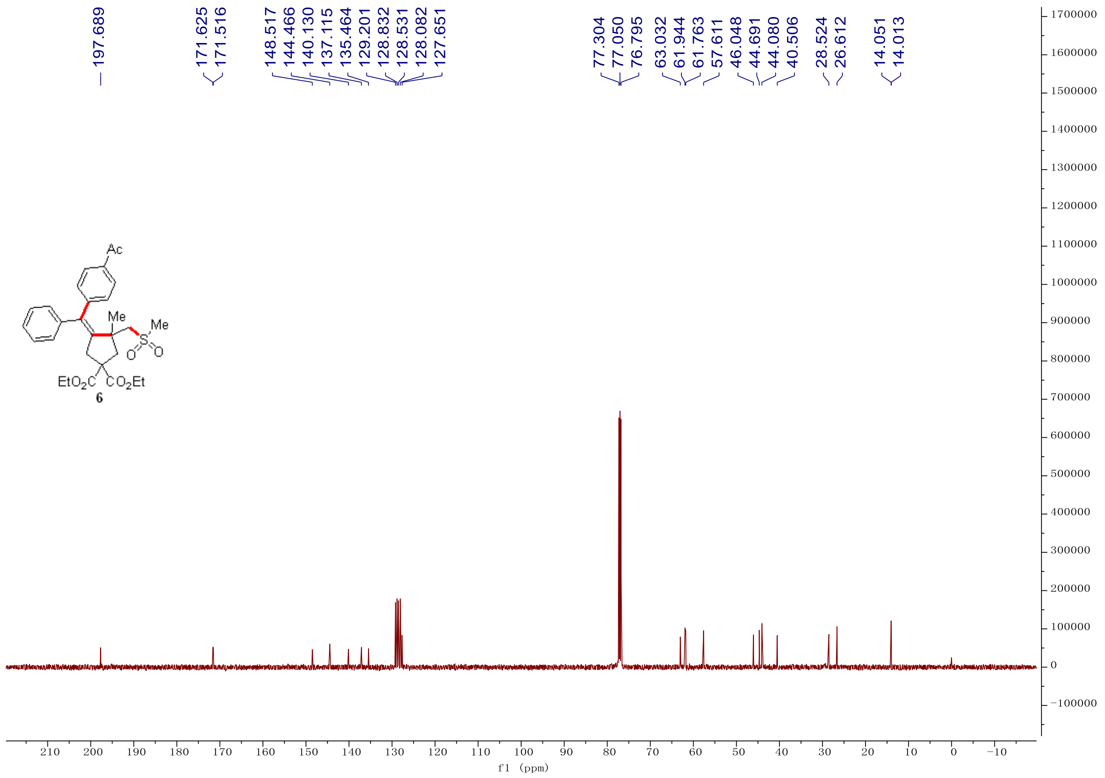

7: ^{1}H NMR (400 Hz, $CDCl_3$)

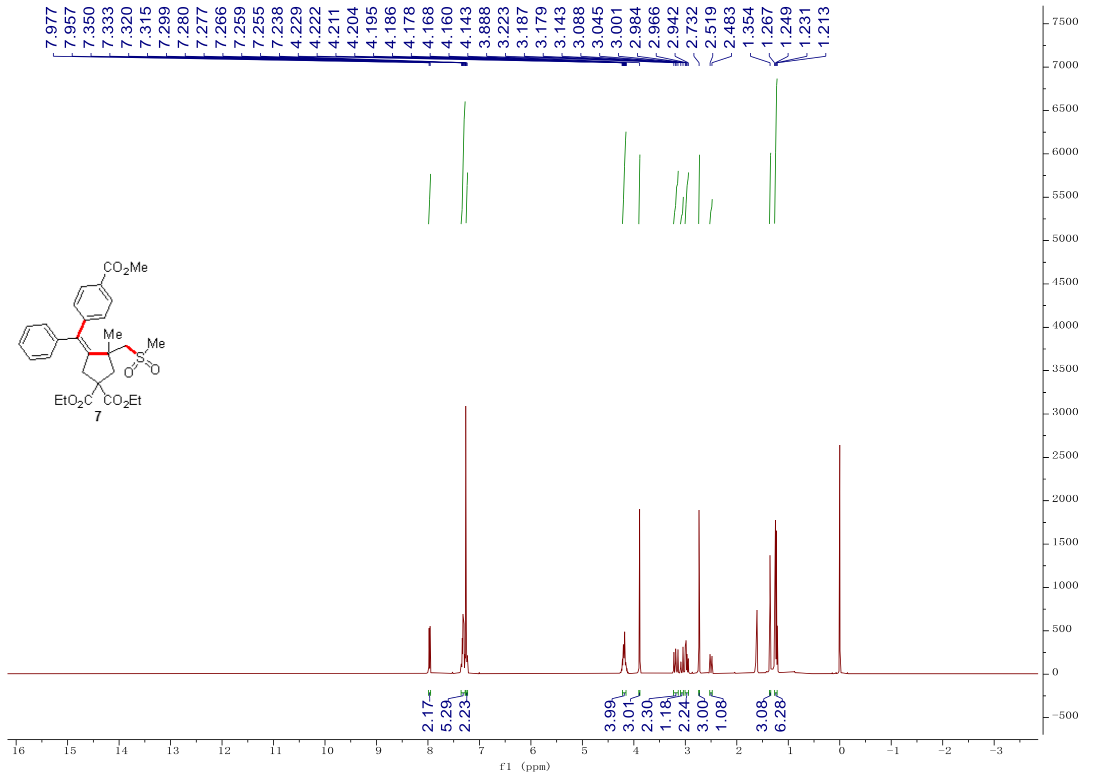

7: ^{13}C NMR (101 Hz, $CDCl_3$)

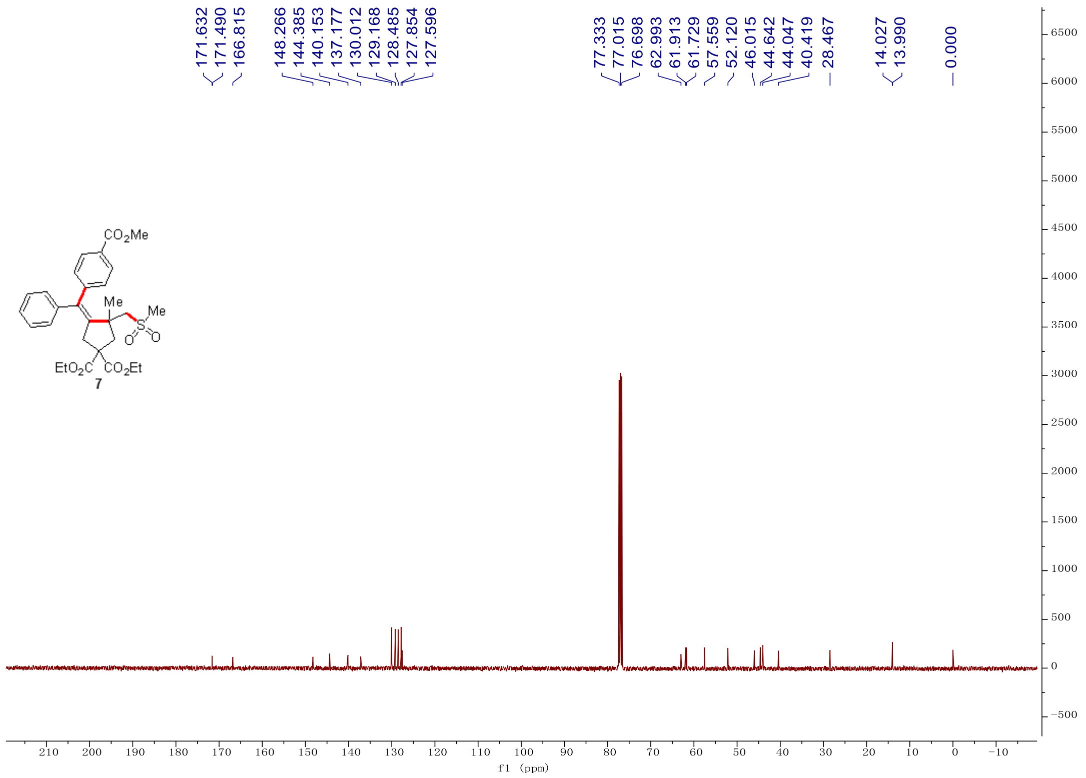

8: ^{1}H NMR (800 Hz, $CDCl_3$)

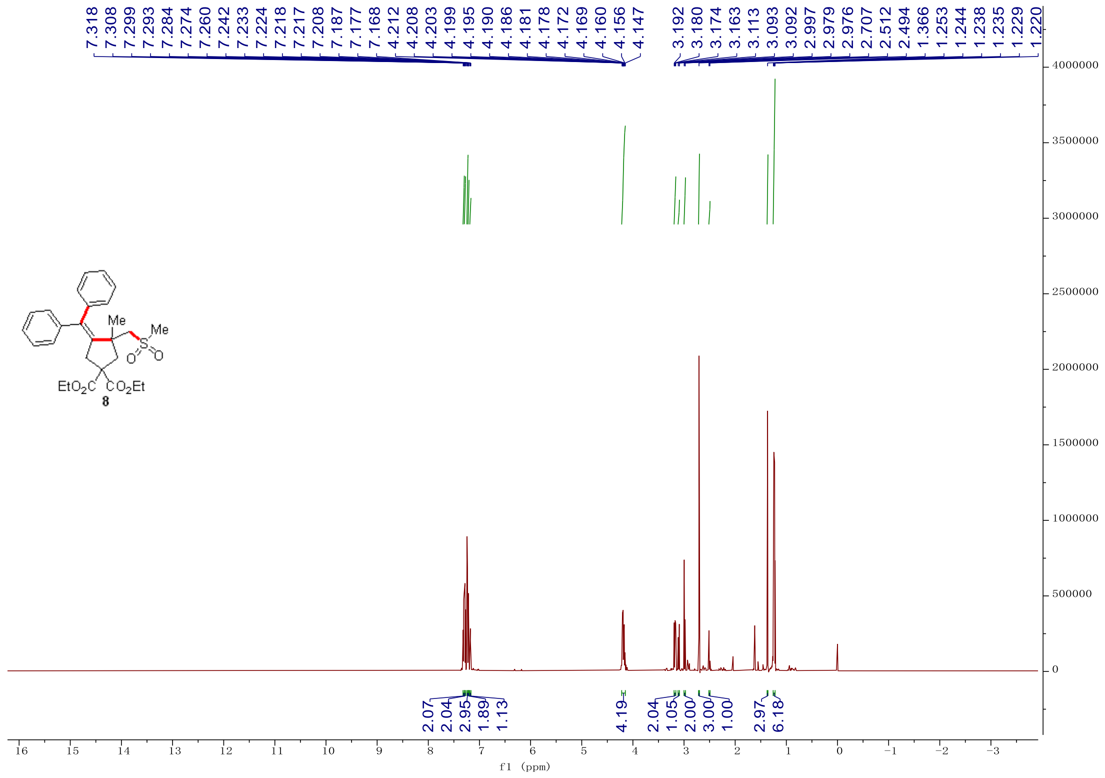

8: ^{13}C NMR (201 Hz, $CDCl_3$)

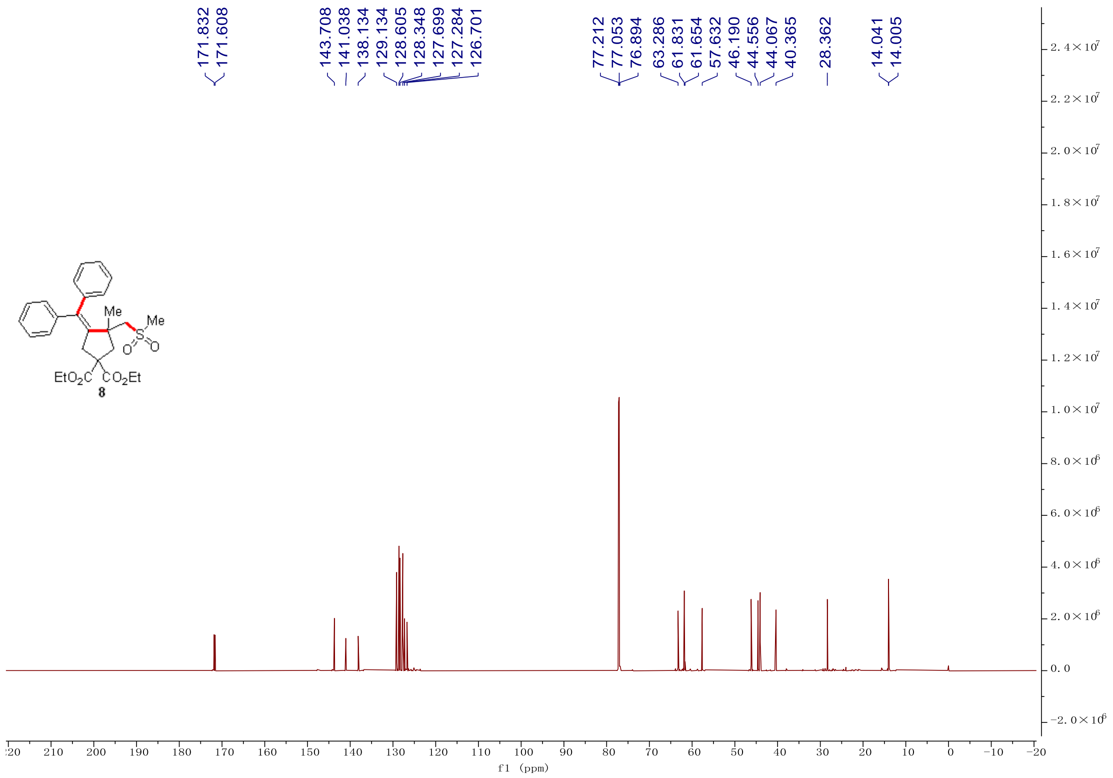

9: ^{1}H NMR (800 Hz, $CDCl_3$)

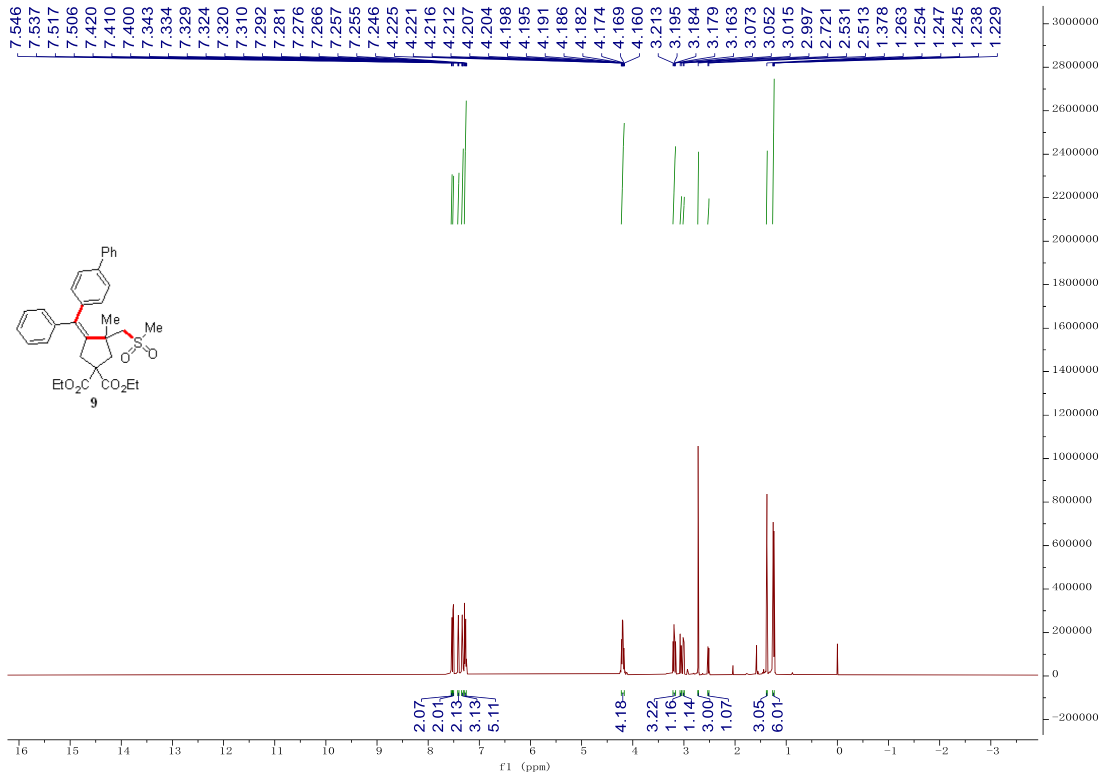

9: ^{13}C NMR (201 Hz, CDCl$_3$)

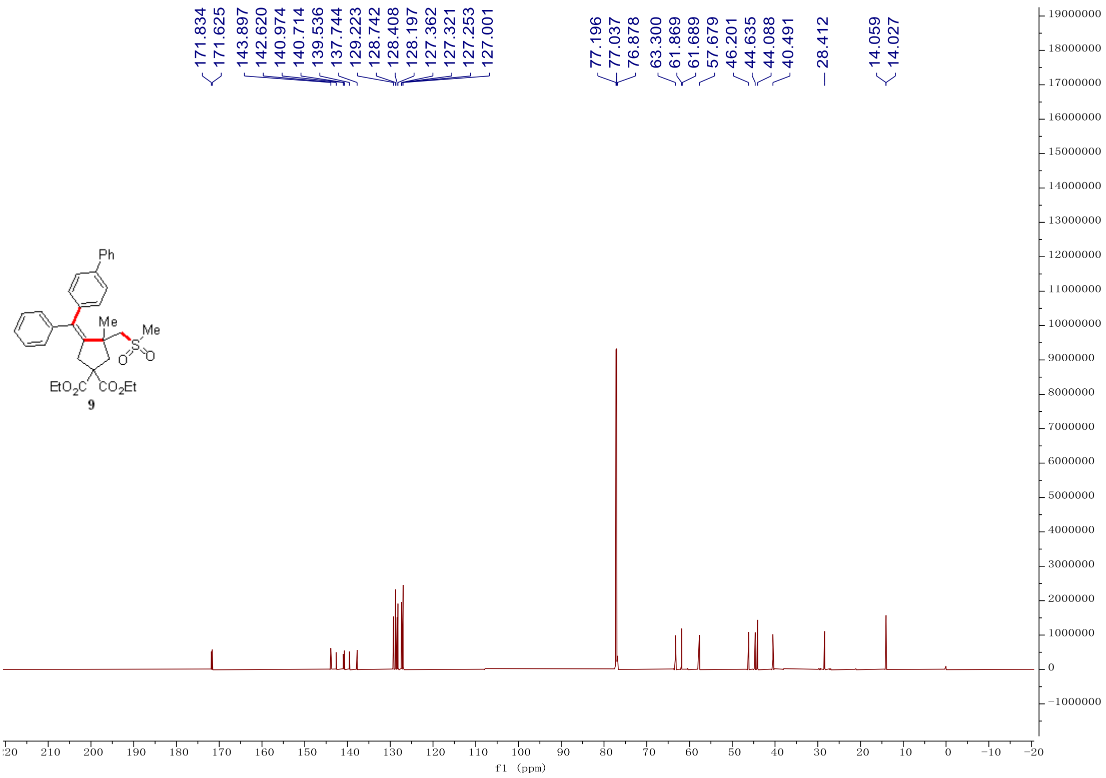

10: ^{1}H NMR (400 Hz, CDCl$_3$)

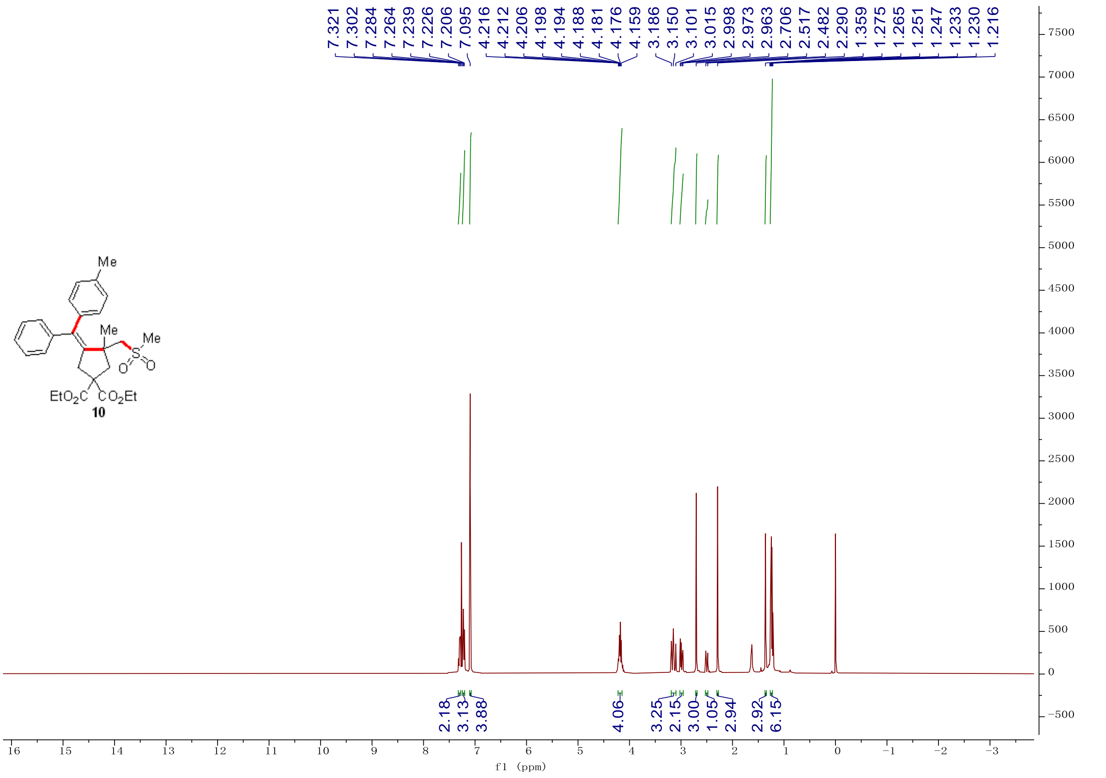

10: ^{13}C NMR (101 Hz, $CDCl_3$)

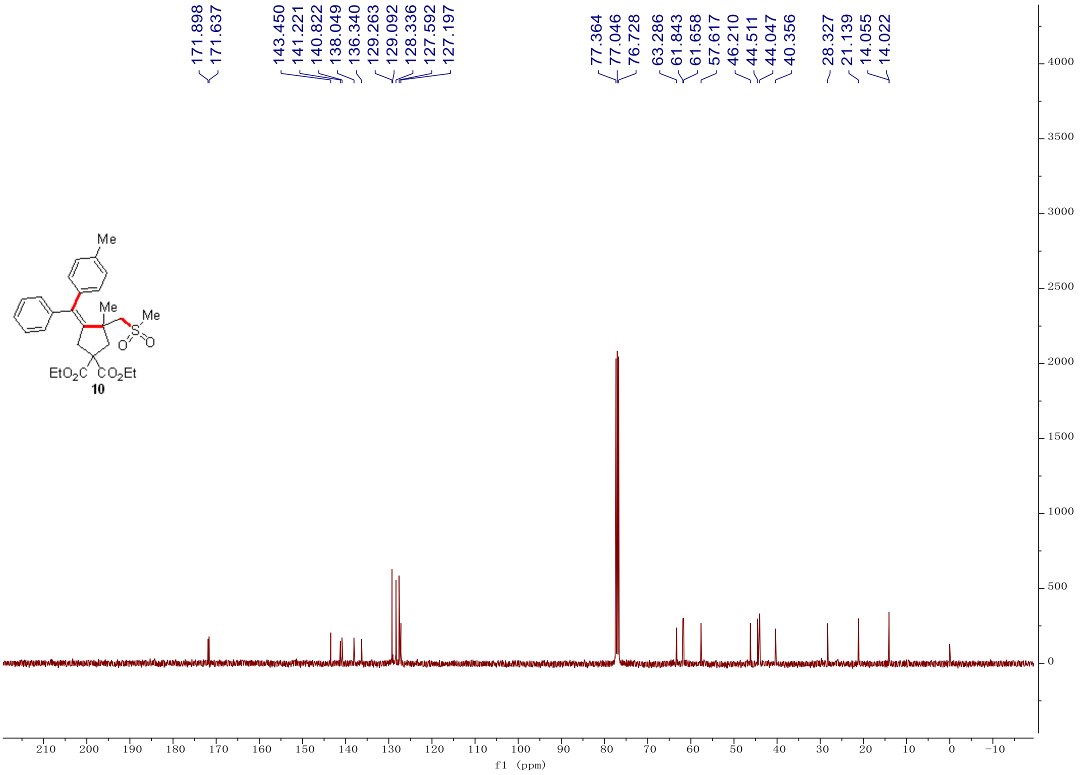

11: ^{1}H NMR (400 Hz, $CDCl_3$)

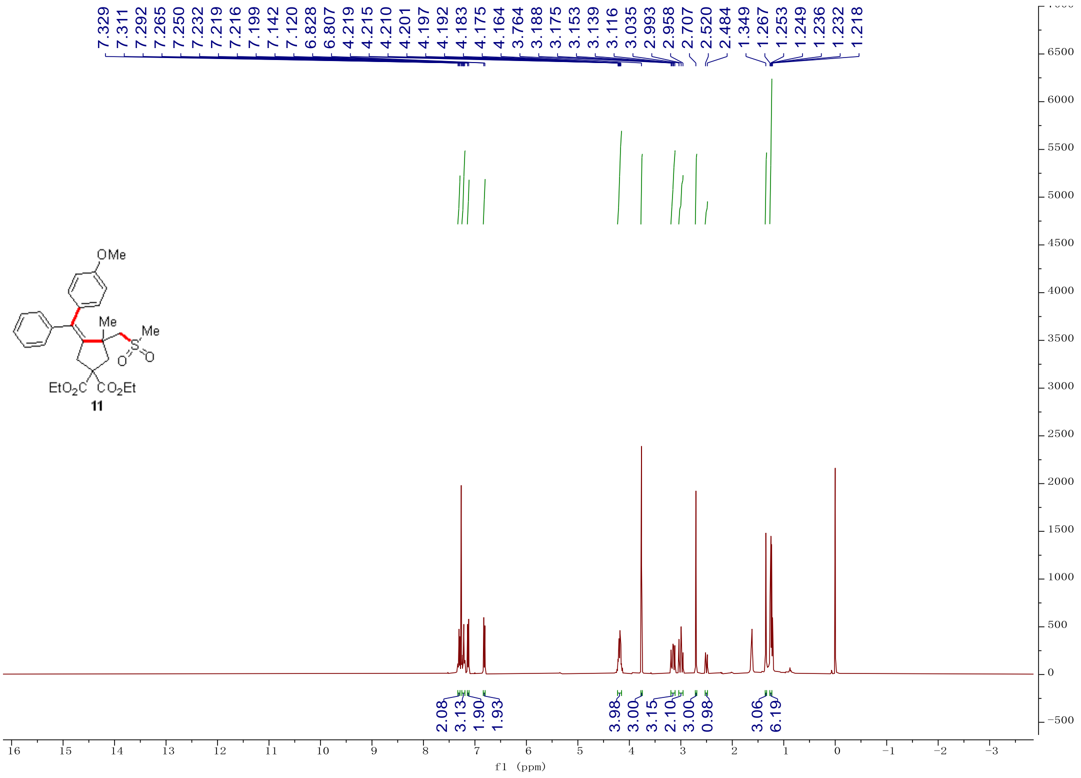

11: ^{13}C NMR (101 Hz, CDCl$_3$)

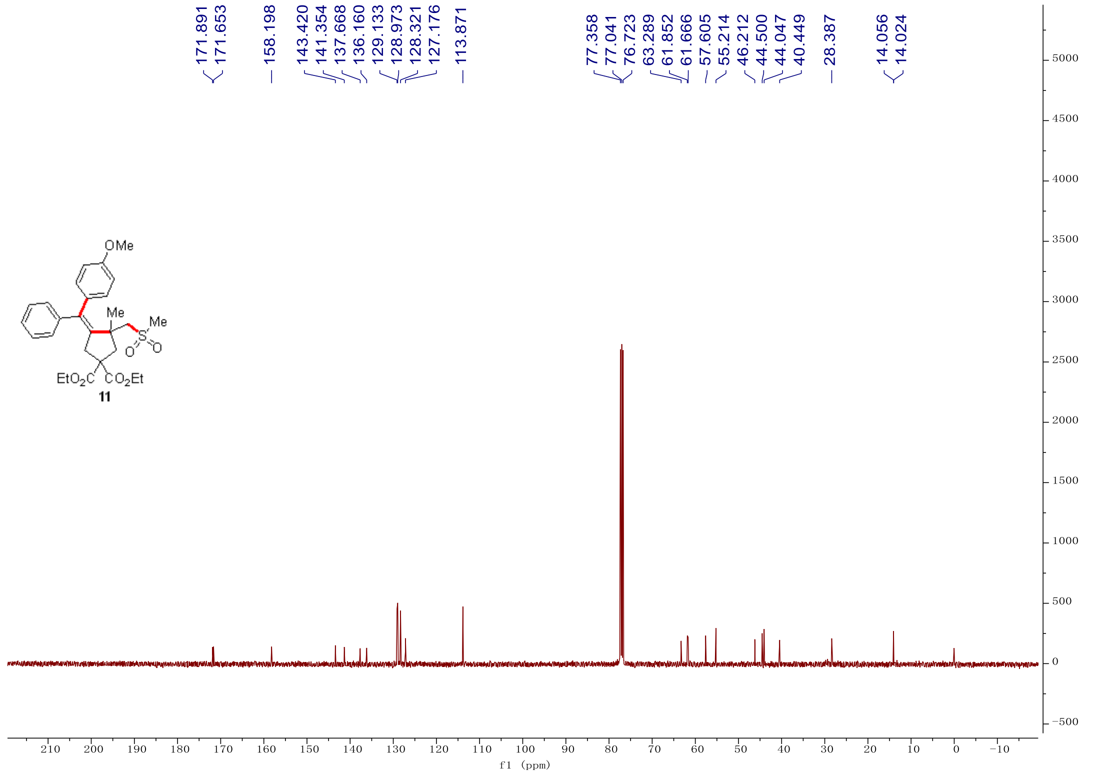

12: ^{1}H NMR (800 Hz, CDCl$_3$)

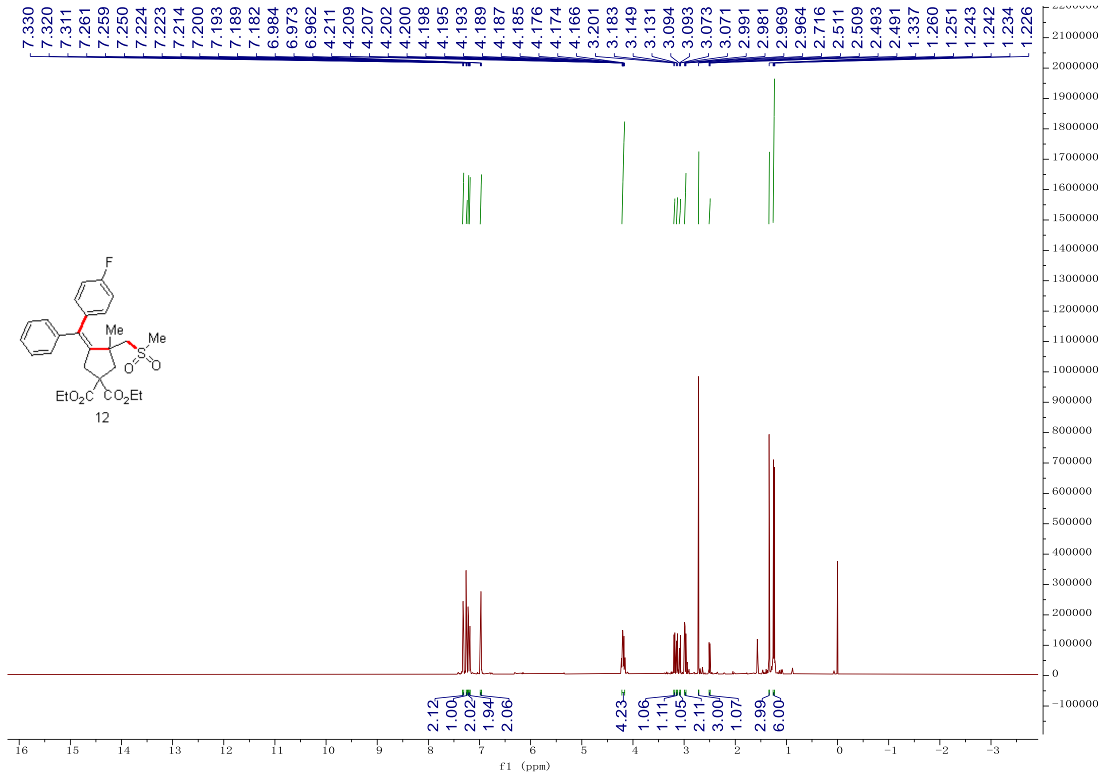

12: ^{13}C NMR (201 Hz, CDCl$_3$)

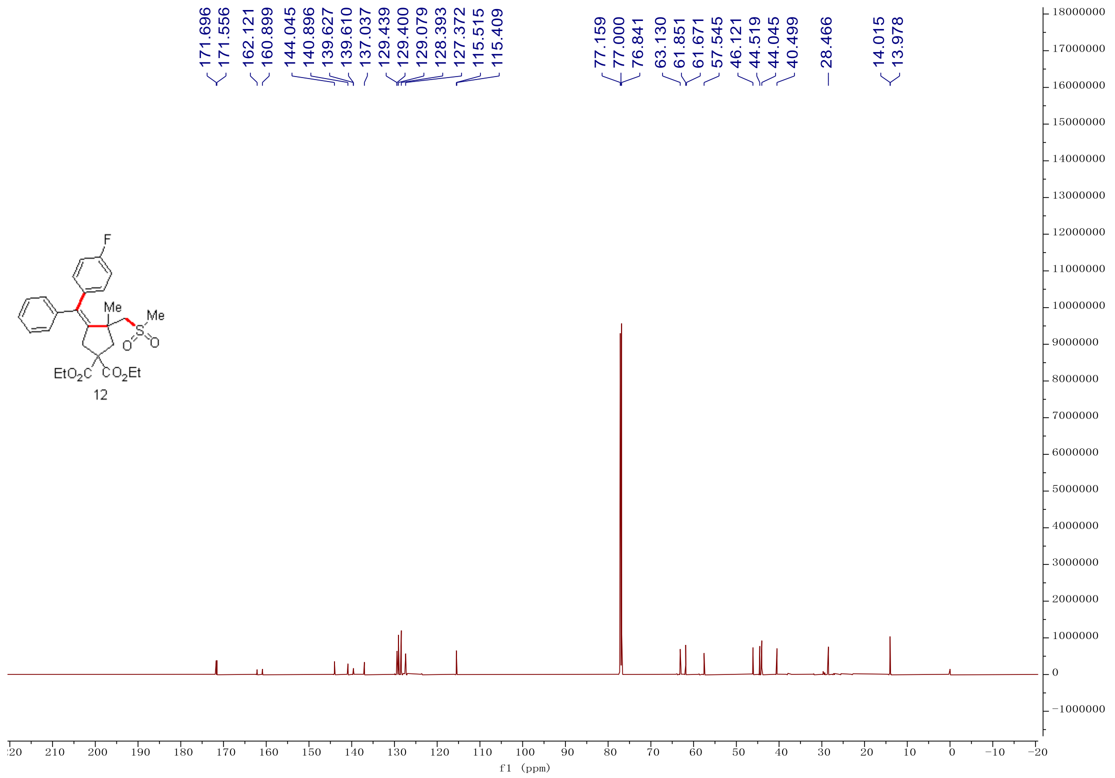

12: ^{19}F NMR (376 Hz, CDCl$_3$)

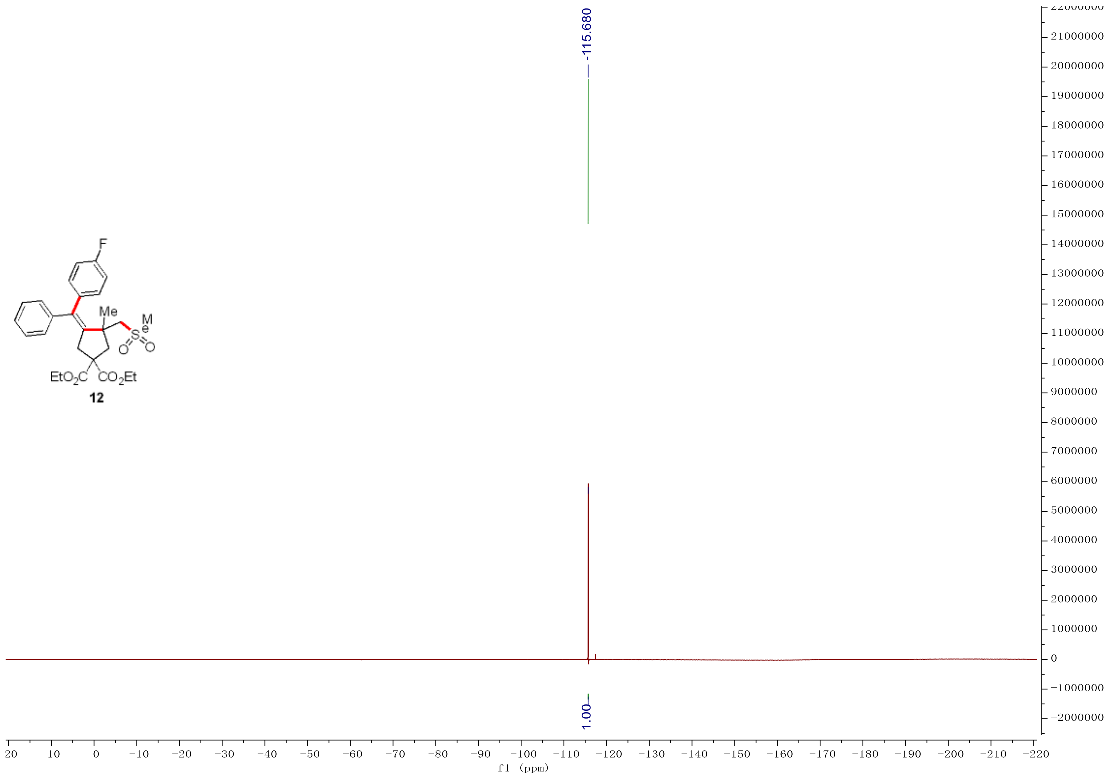

13: ^{1}H NMR (500 Hz, $CDCl_3$)

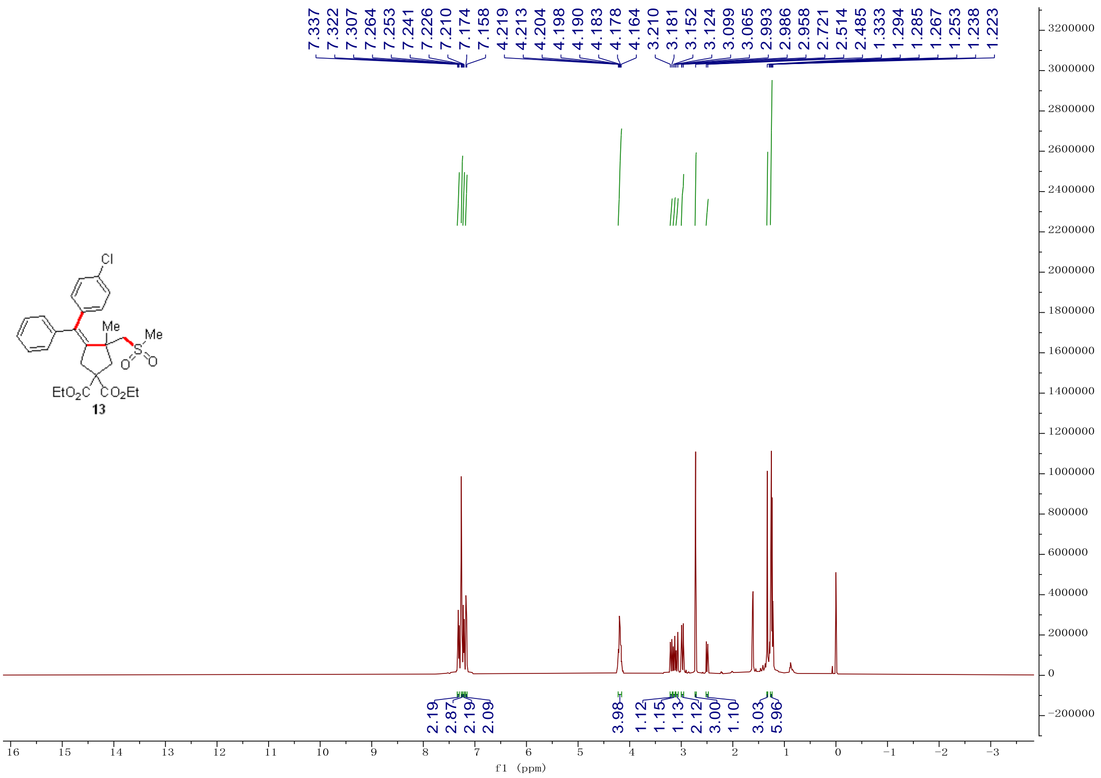

13: ^{13}C NMR (126 Hz, $CDCl_3$)

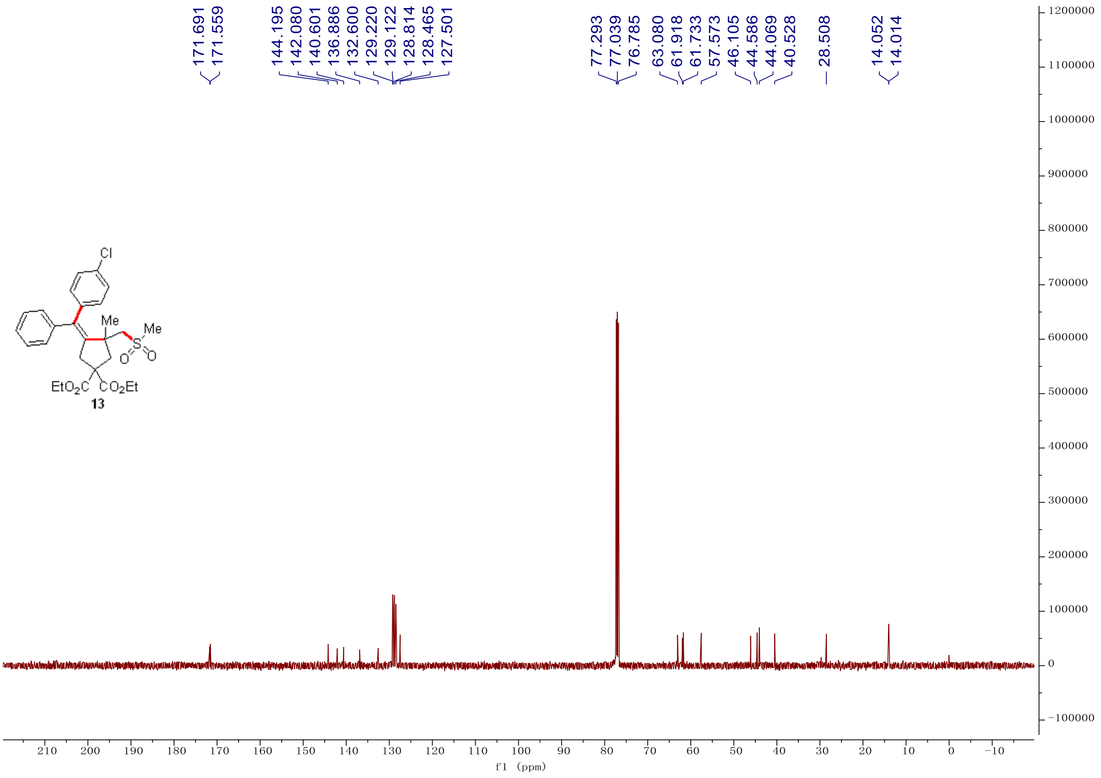

14: ^{1}H NMR (400 Hz, CDCl$_3$)

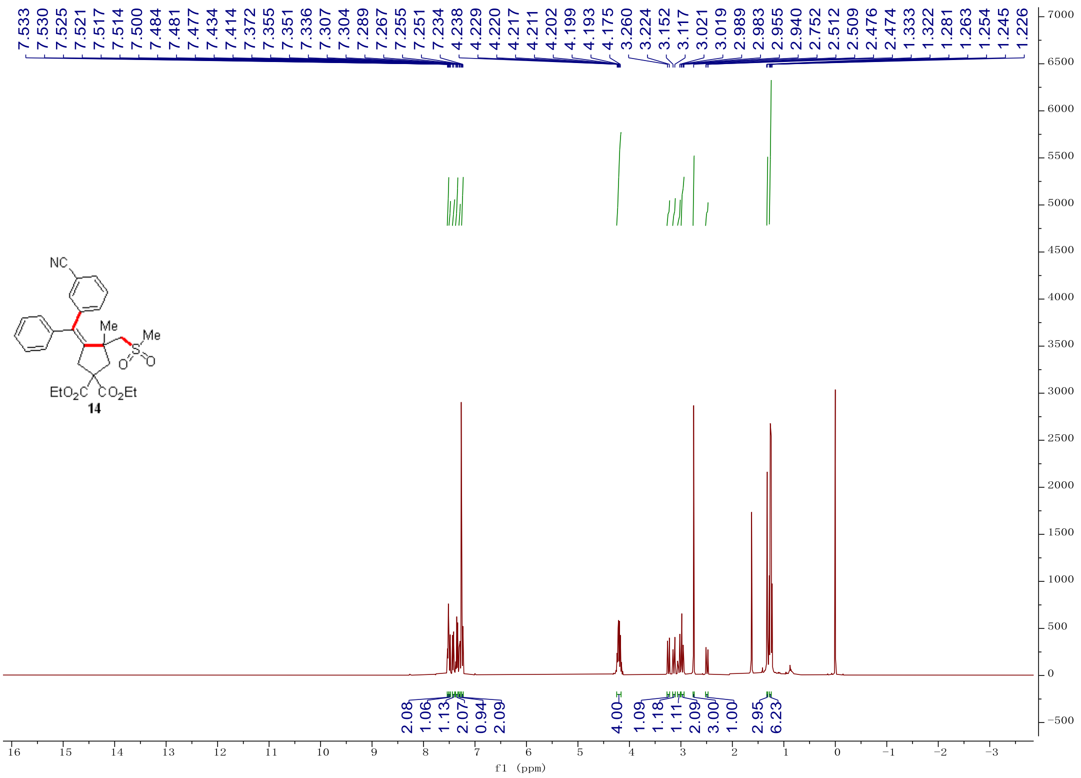

14: ^{13}C NMR (101 Hz, CDCl$_3$)

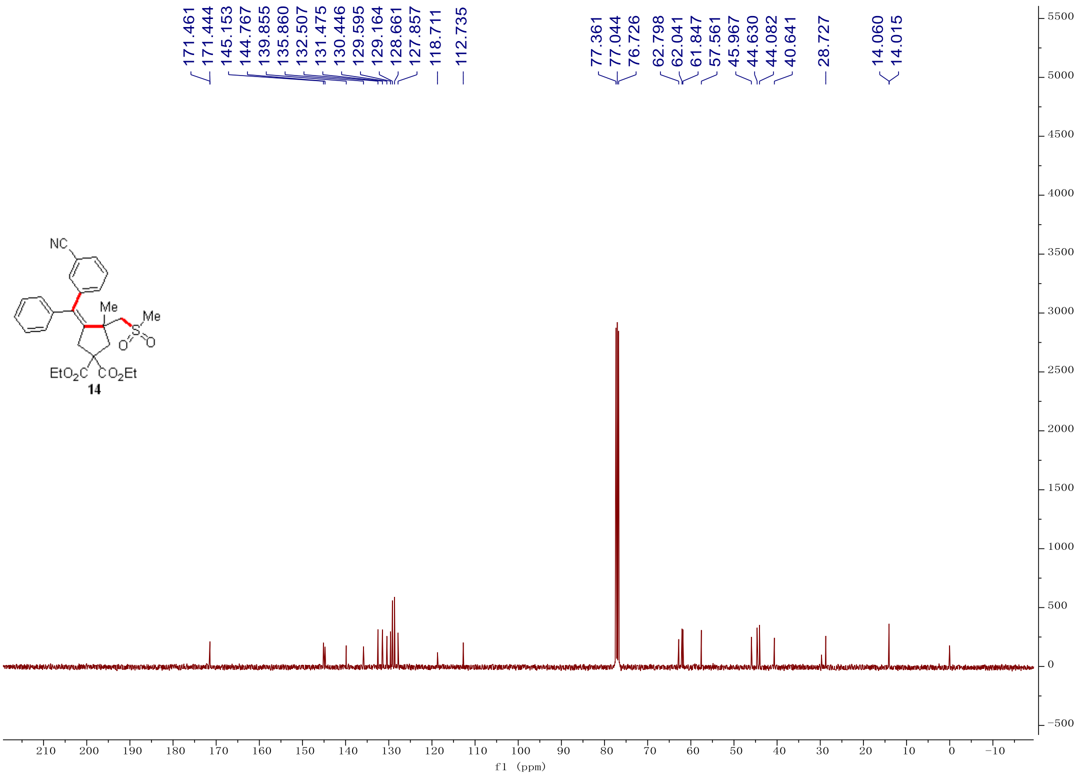

15: ^{1}H NMR (400 Hz, $CDCl_3$)

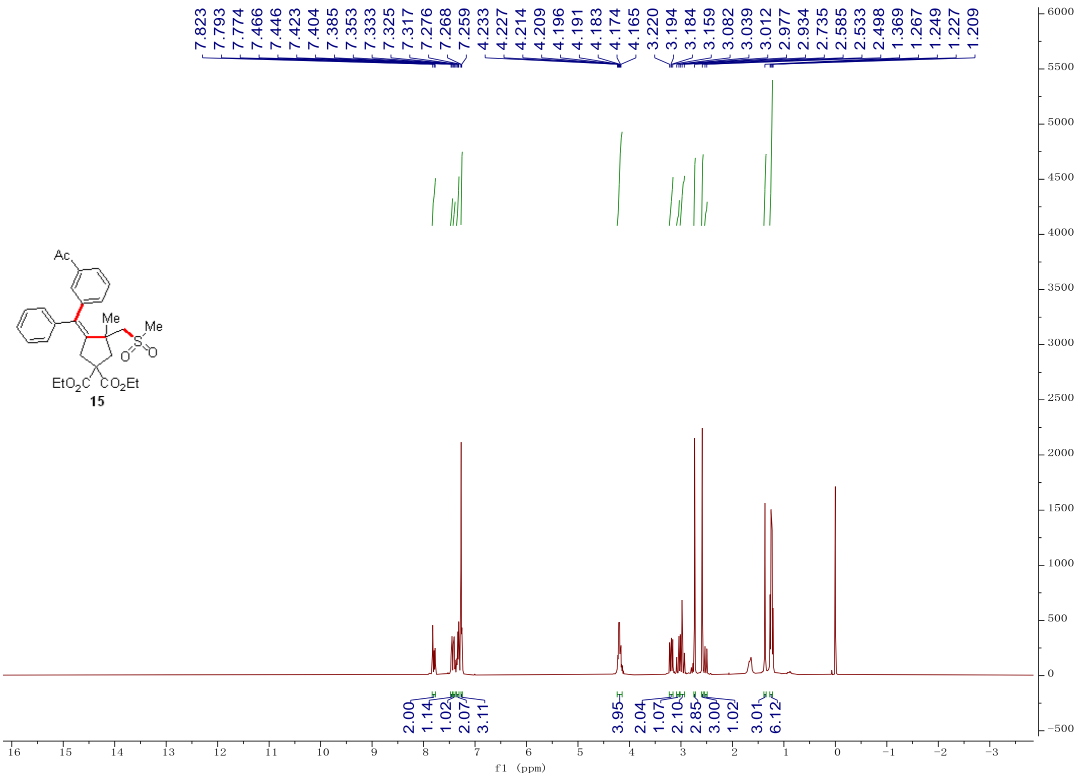

15: ^{13}C NMR (101 Hz, $CDCl_3$)

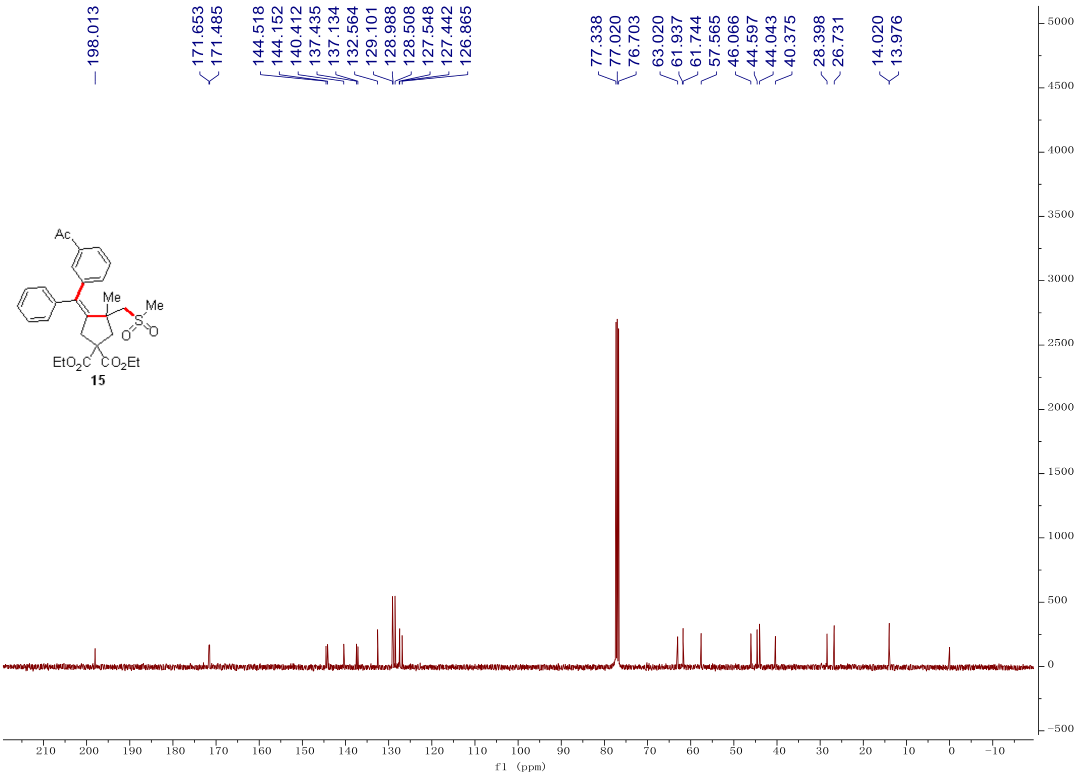

16: ^{1}H NMR (400 Hz, $CDCl_3$)

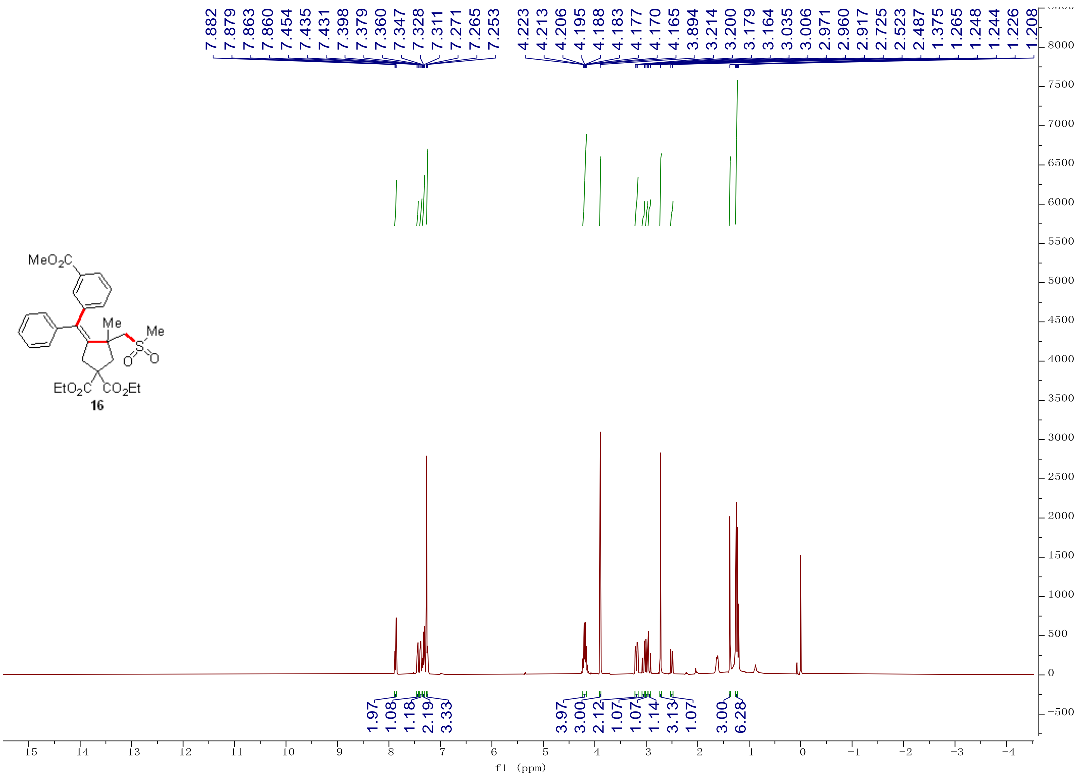

16: ^{13}C NMR (101 Hz, $CDCl_3$)

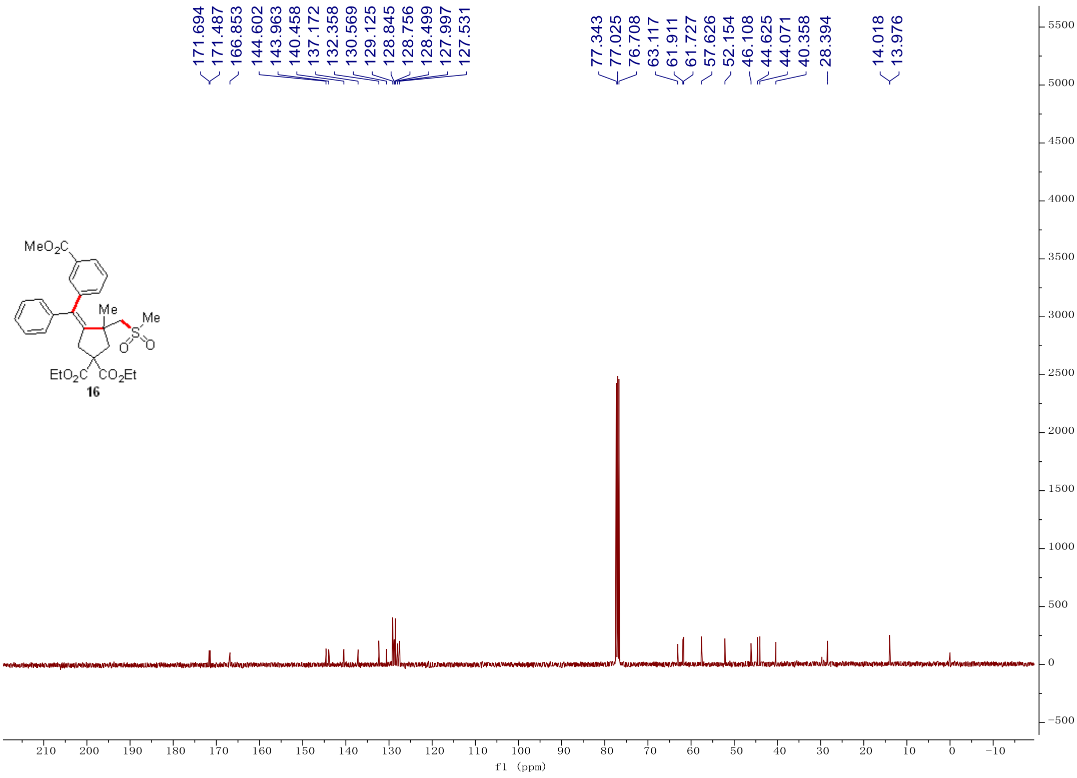

17: ^{1}H NMR (800 Hz, $CDCl_3$)

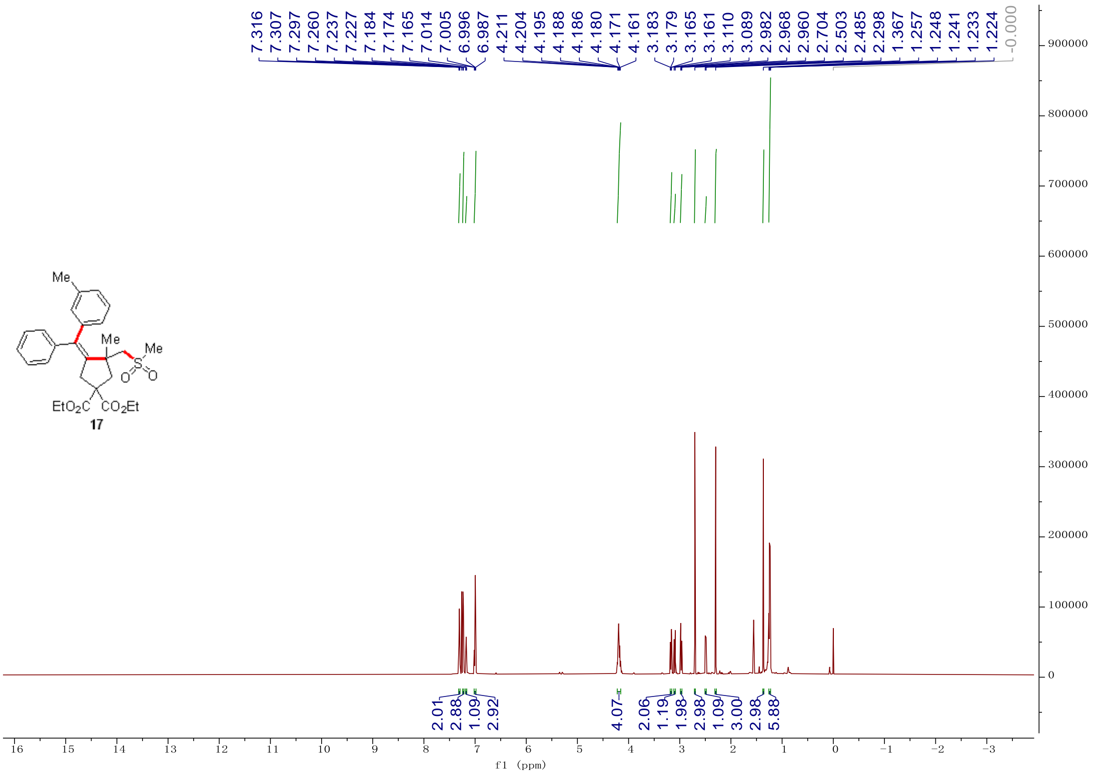

17: ^{13}C NMR (201 Hz, $CDCl_3$)

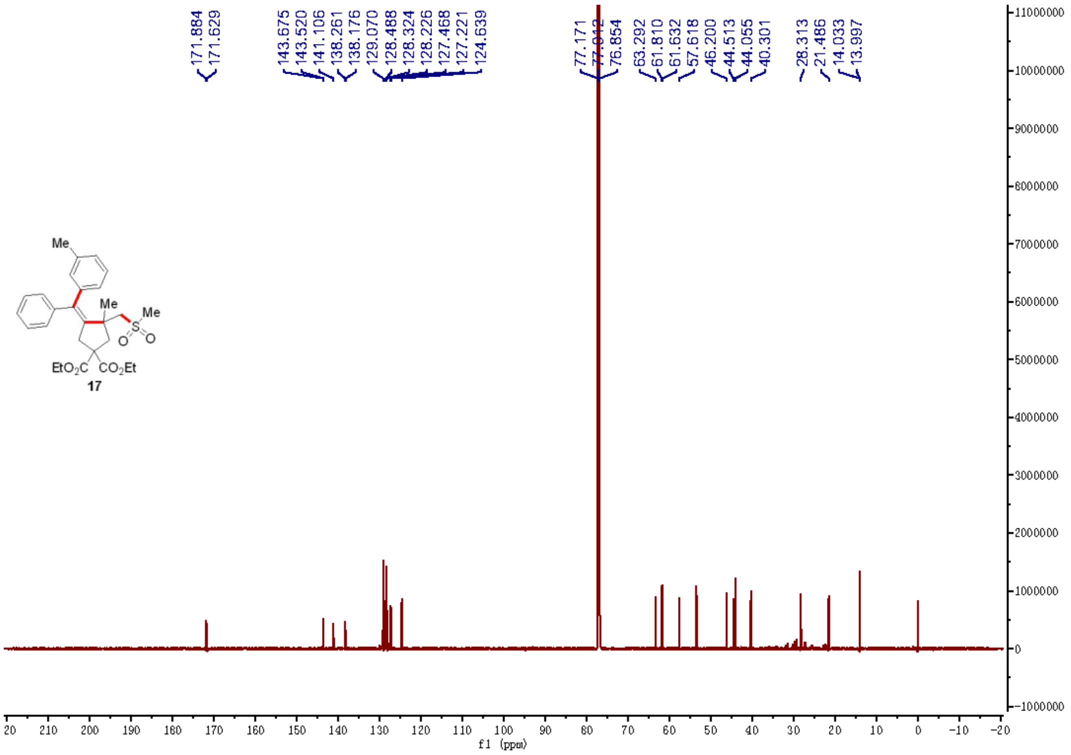

18: ^{1}H NMR (400 Hz, $CDCl_3$)

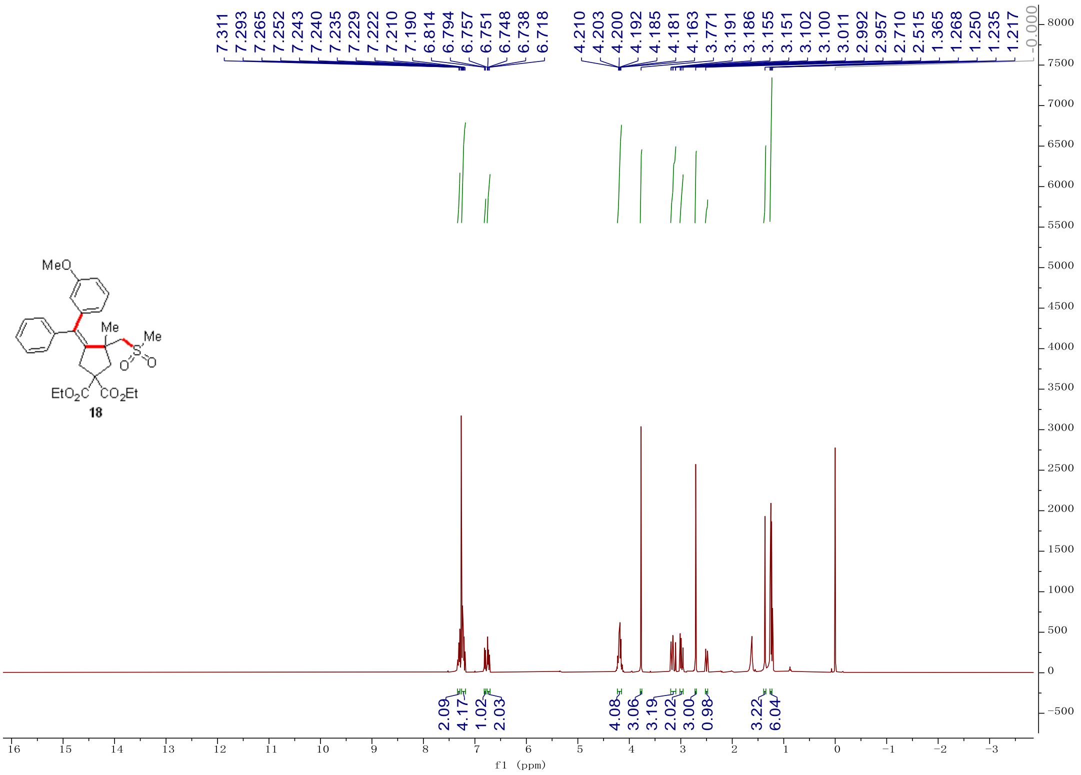

18: ^{13}C NMR (101 Hz, $CDCl_3$)

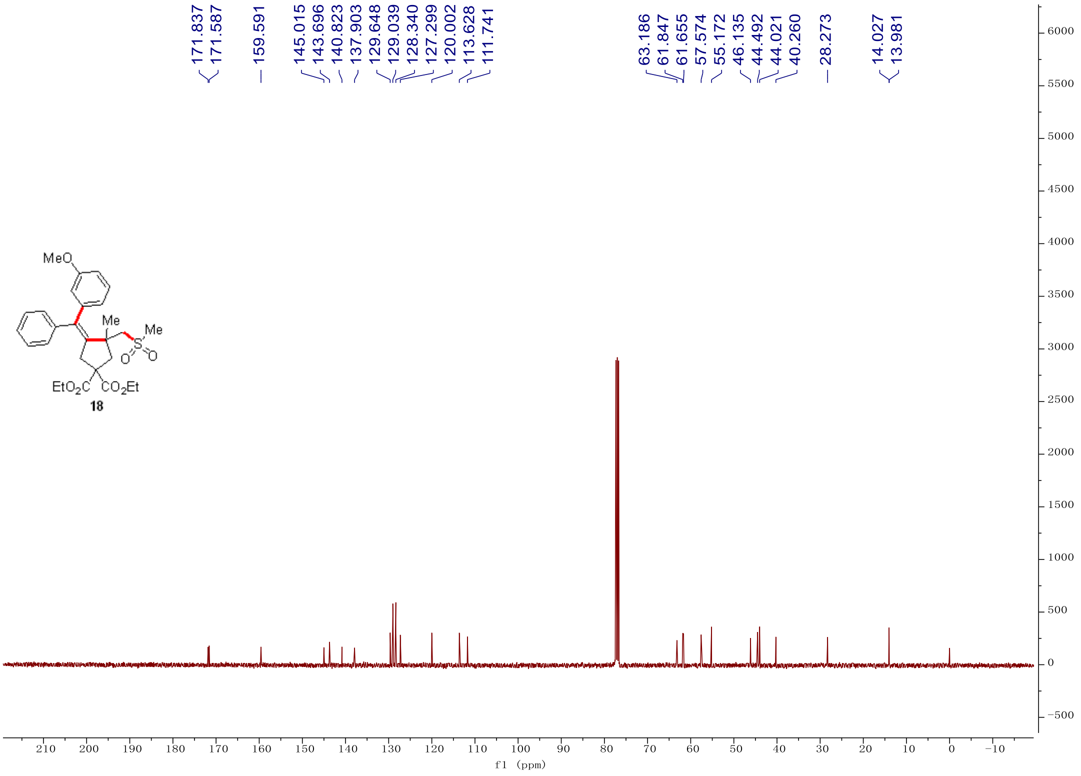

19: ^{1}H NMR (500 Hz, CDCl$_3$)

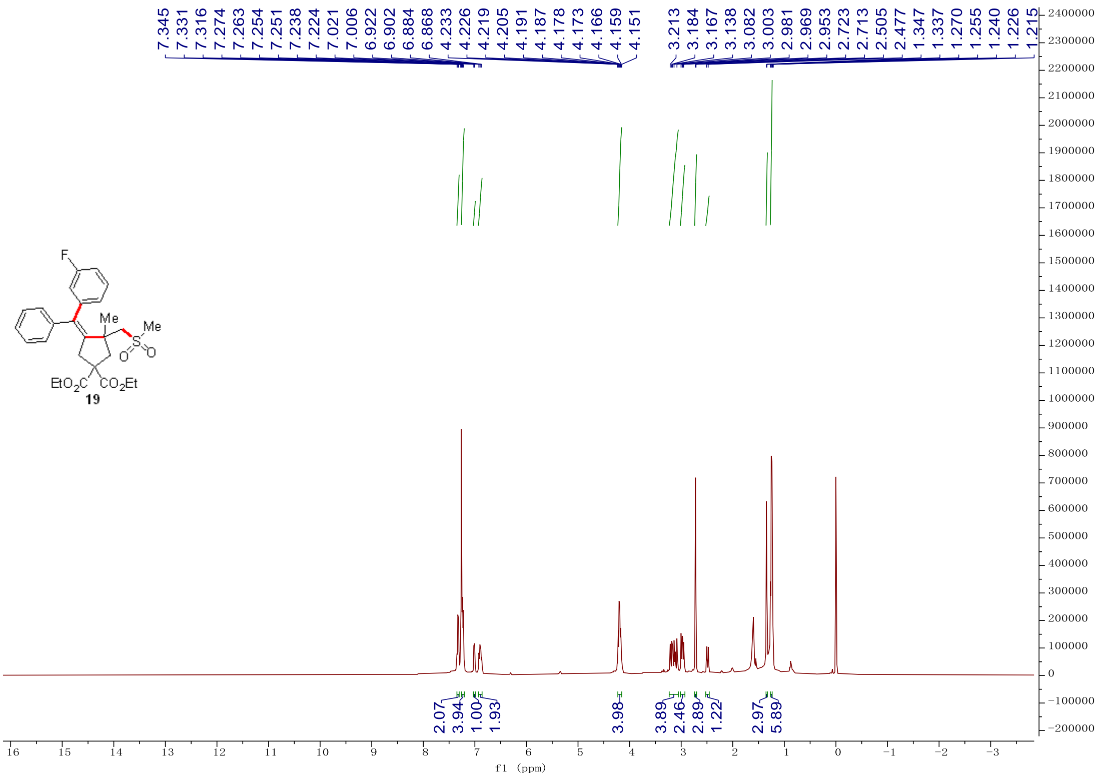

19: ^{13}C NMR (126 Hz, CDCl$_3$)

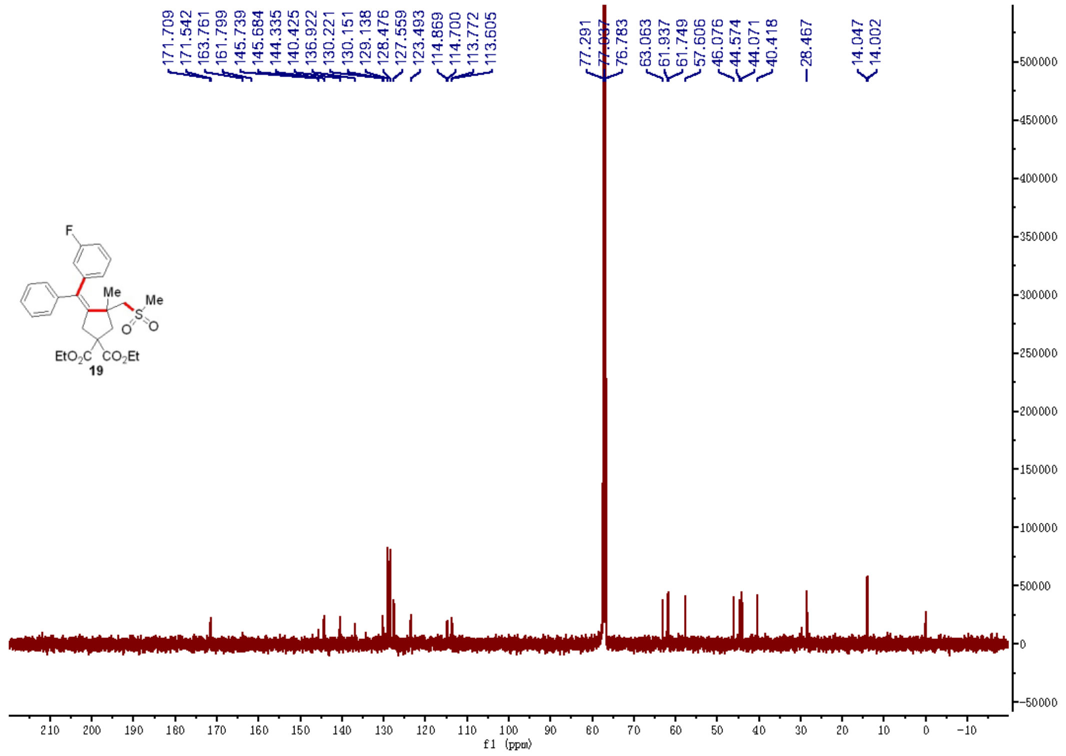

19: ^{19}F NMR (753 Hz, $CDCl_3$)

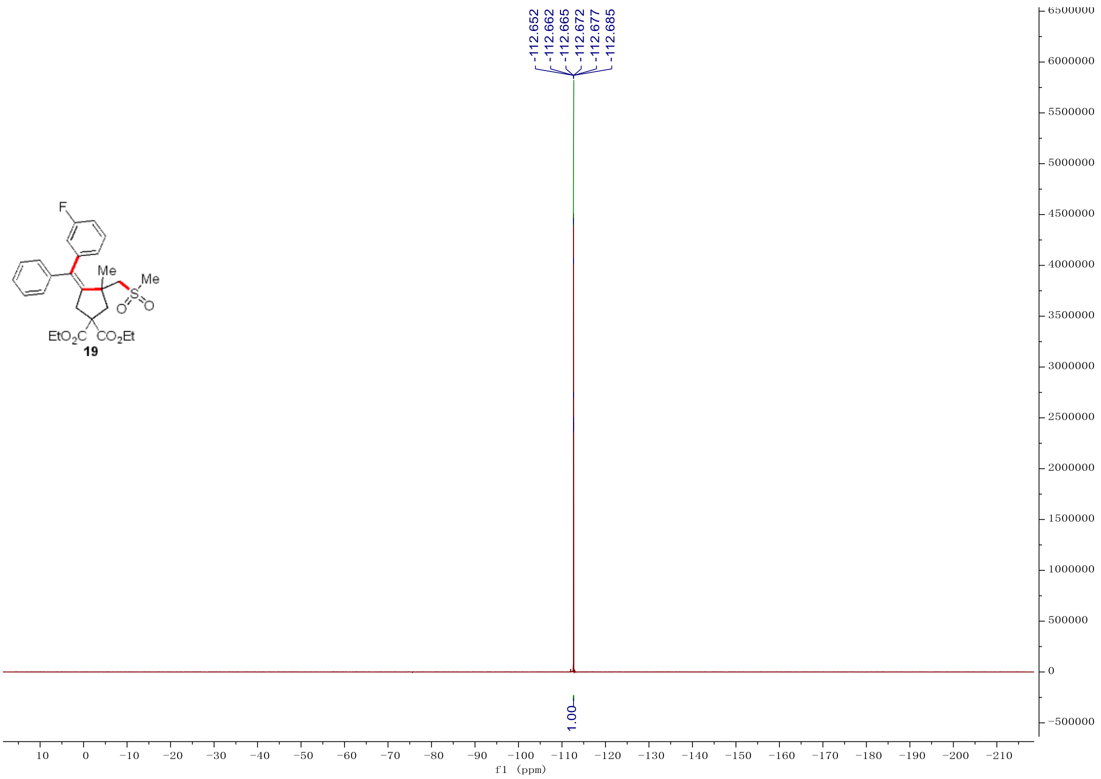

20: ^{1}H NMR (500 Hz, $CDCl_3$)

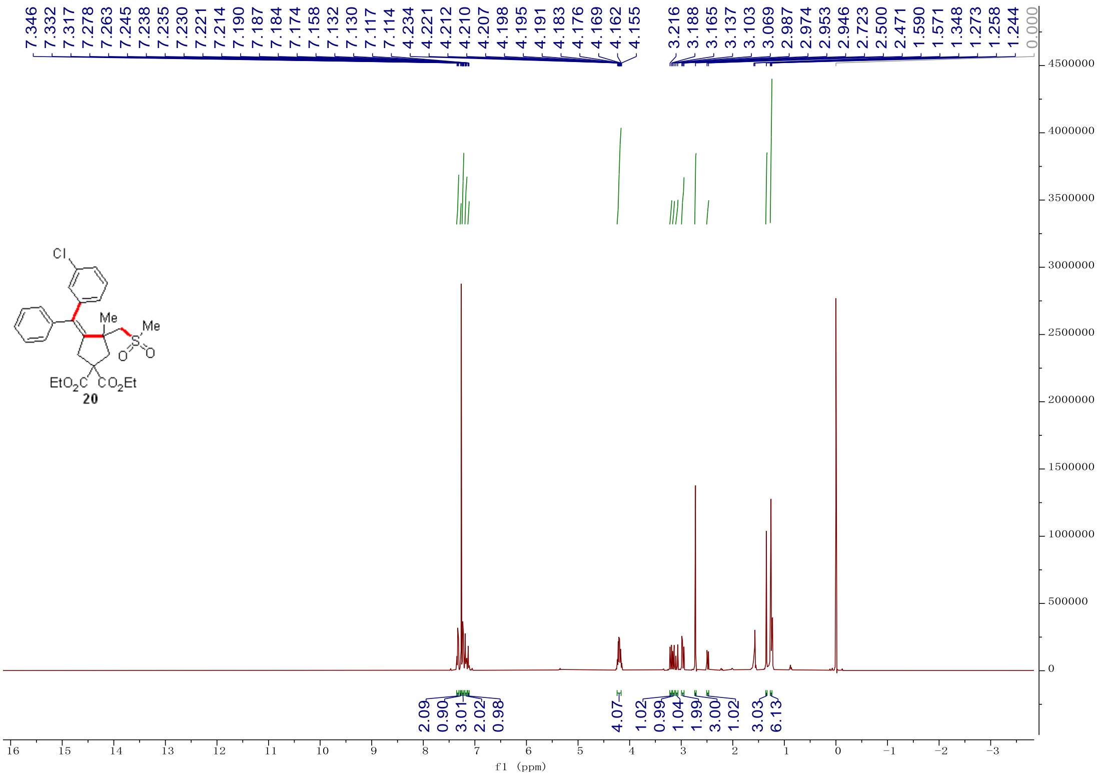

20: ^{13}C NMR (126 Hz, CDCl$_3$)

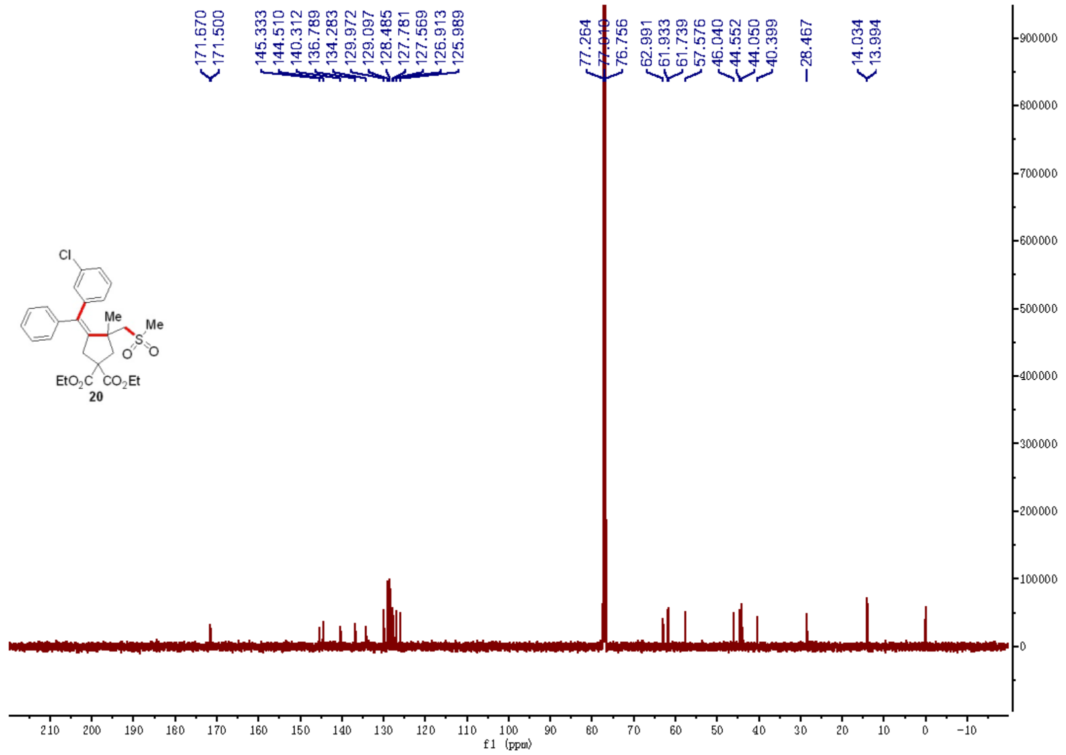

21: ^{1}H NMR (400 Hz, CDCl$_3$)

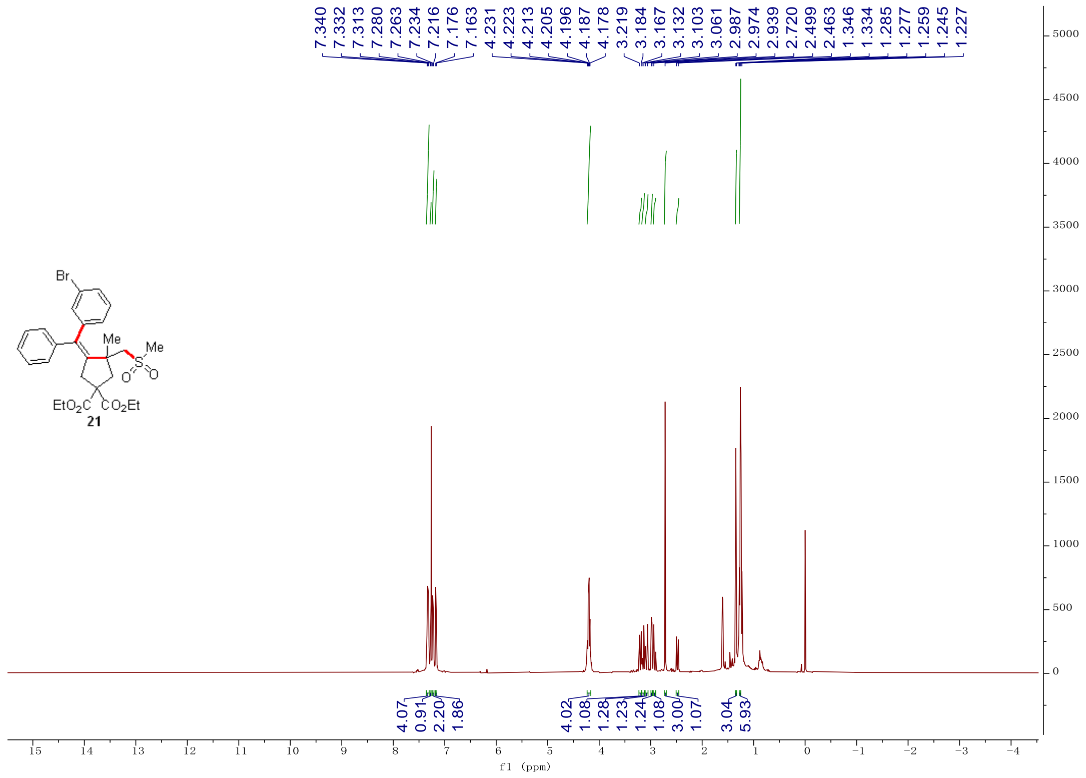

21: ^{13}C NMR (101 Hz, $CDCl_3$)

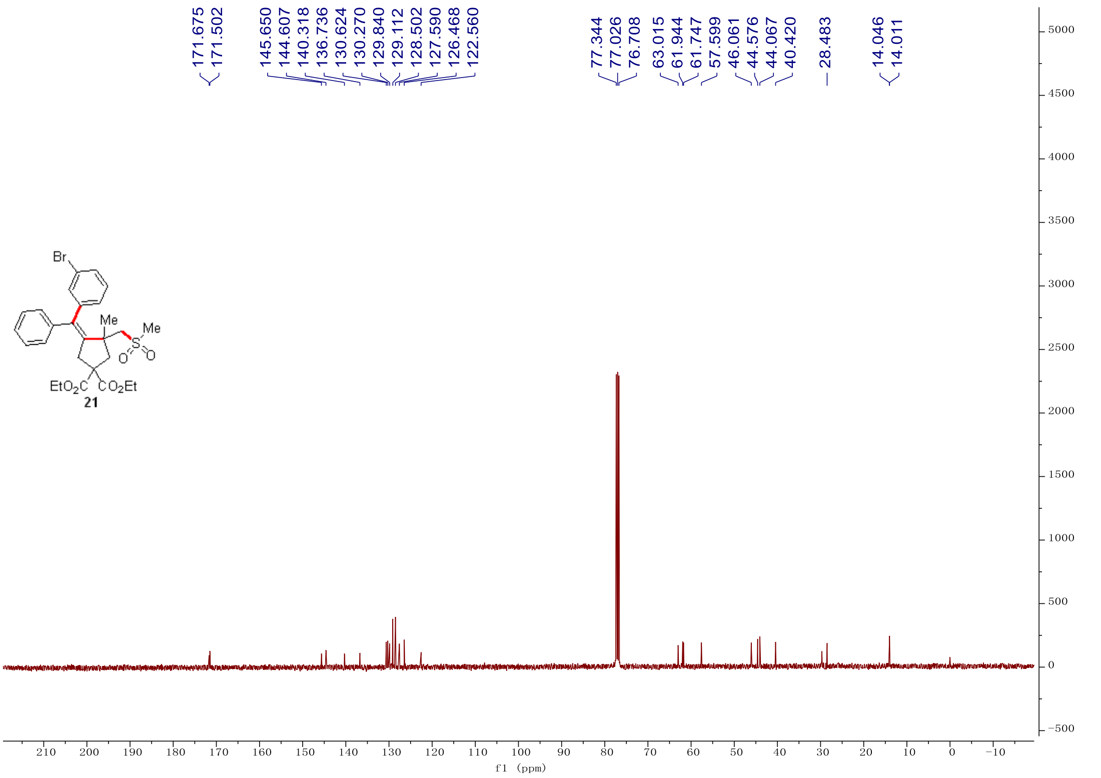

22: ^{1}H NMR (800 Hz, $CDCl_3$)

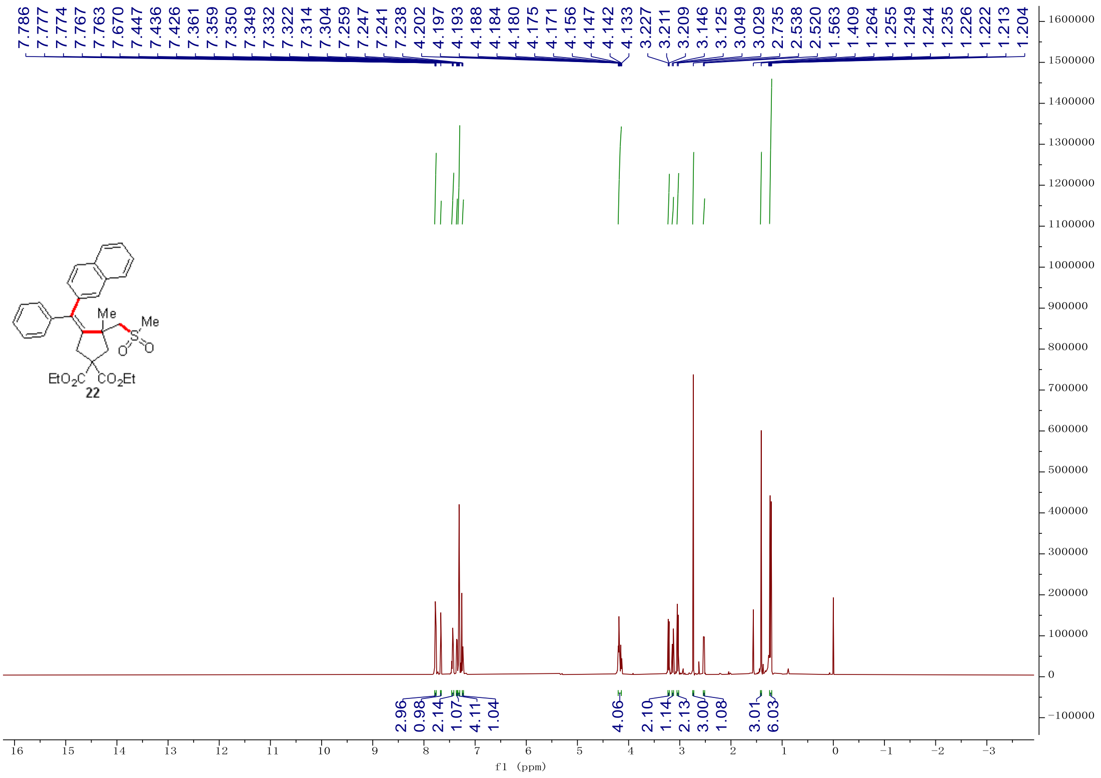

22: ^{13}C NMR (201 Hz, CDCl$_3$)

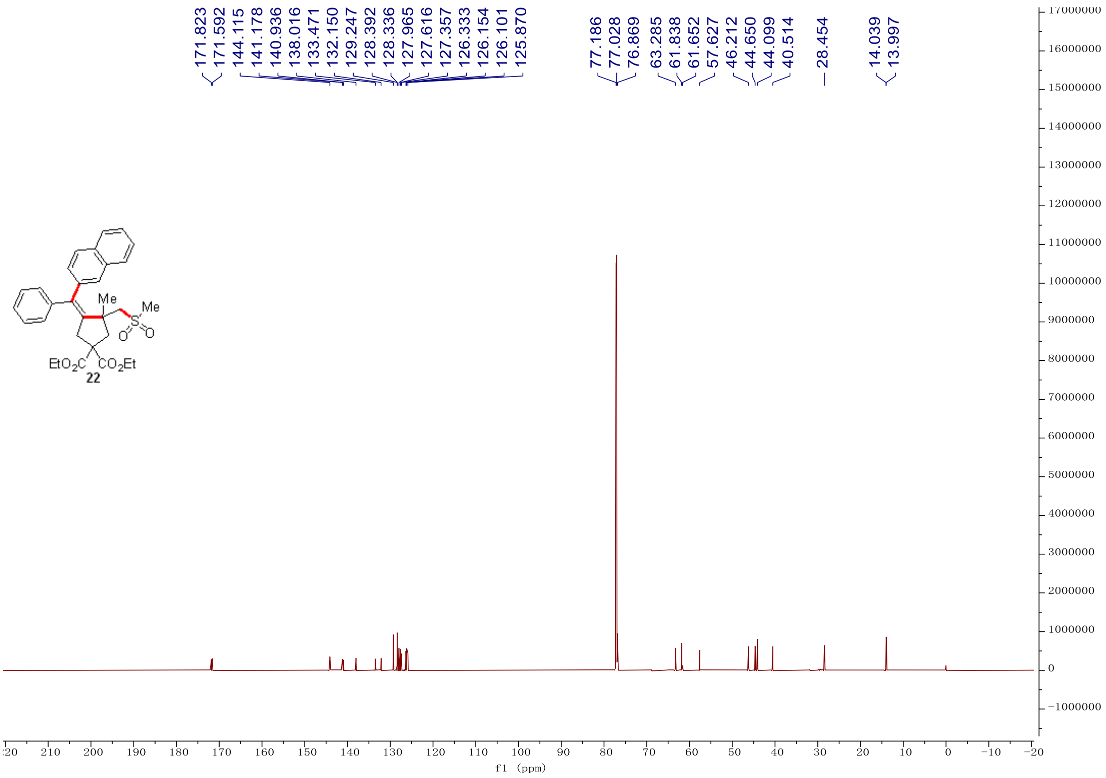

23: ^{1}H NMR (800 Hz, CDCl$_3$)

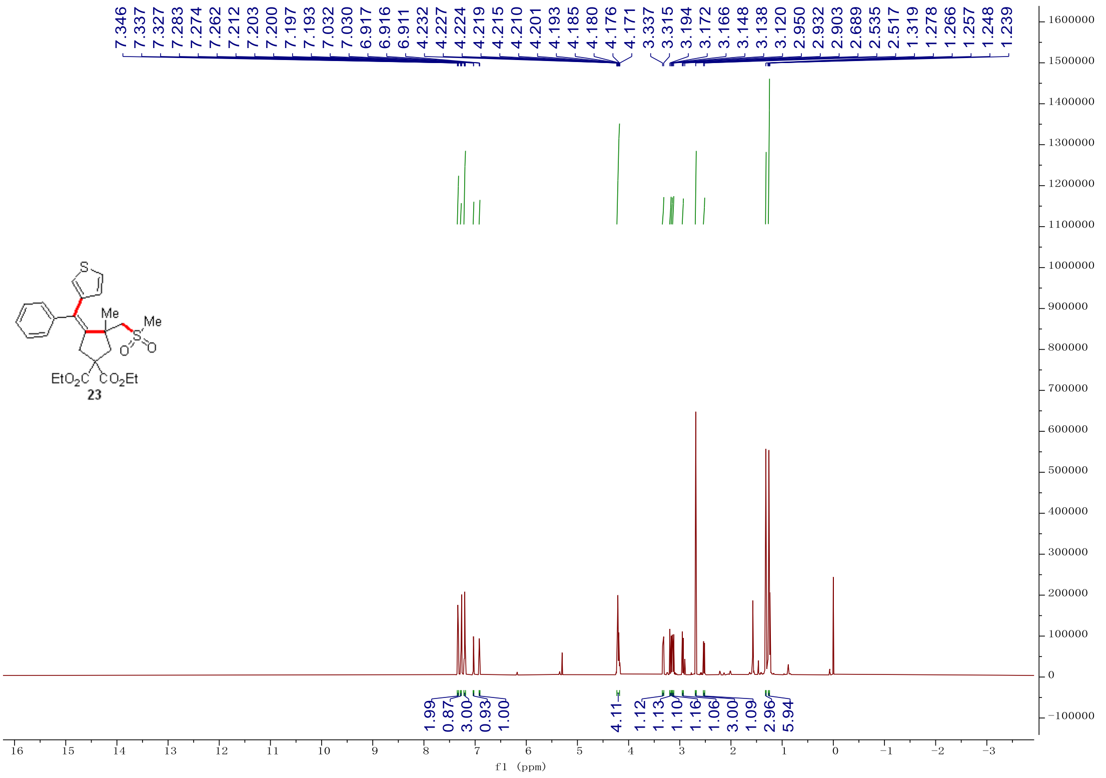

23: ^{13}C NMR (201 Hz, $CDCl_3$)

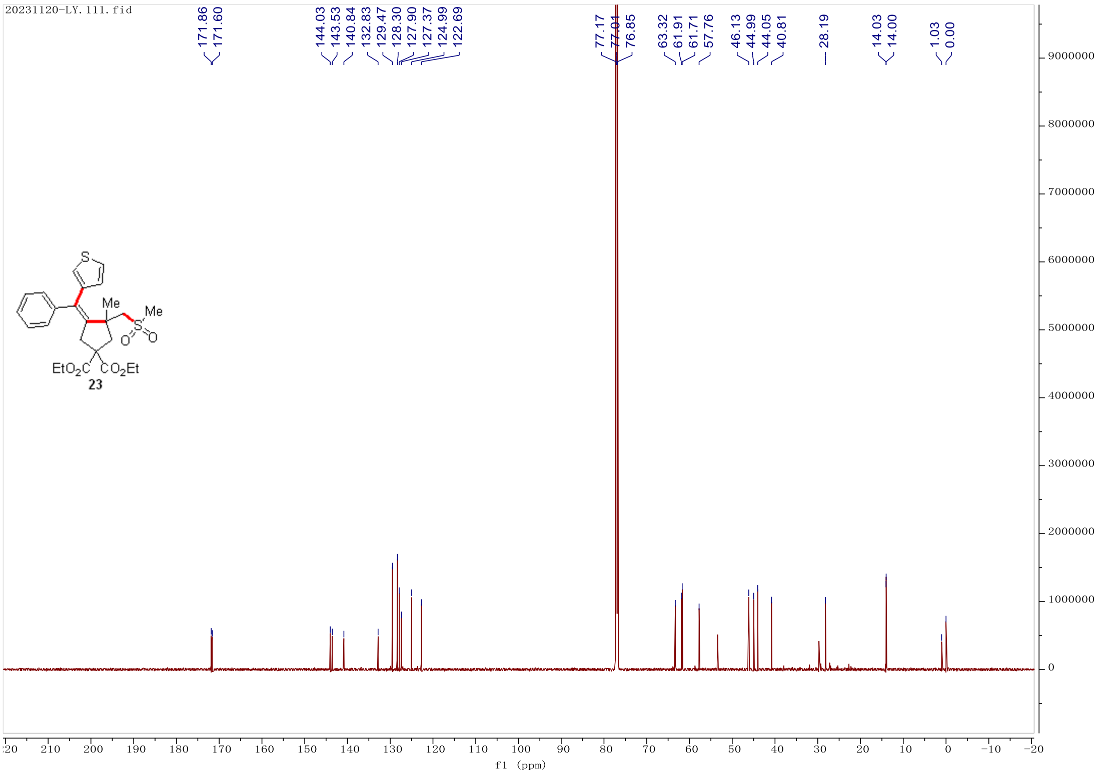

24: ^{1}H NMR (400 Hz, $CDCl_3$)

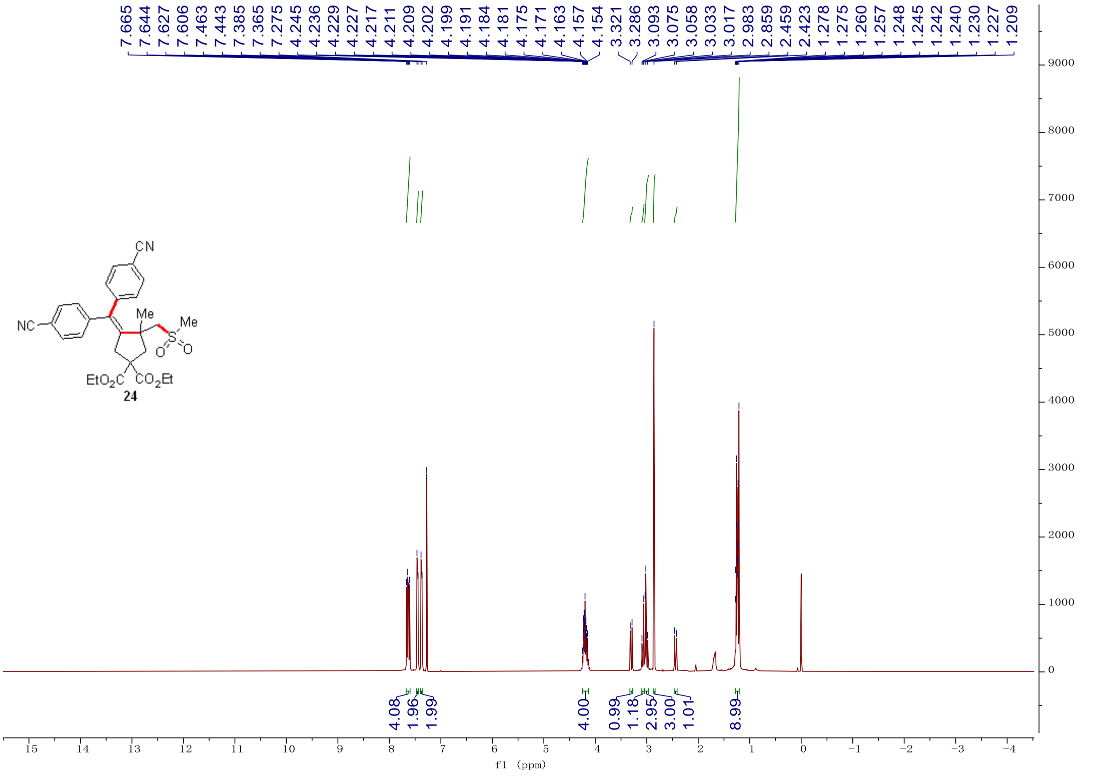

24: ^{13}C NMR (101 Hz, CDCl$_3$)

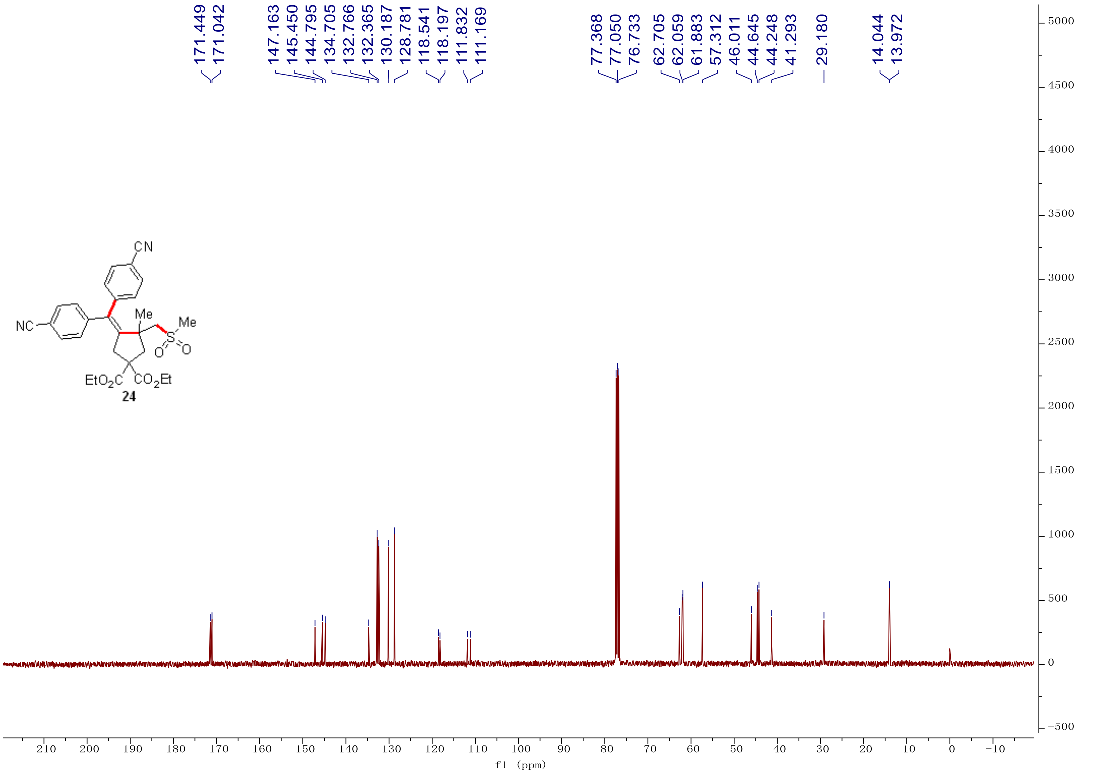

25: ^{1}H NMR (500 Hz, CDCl$_3$)

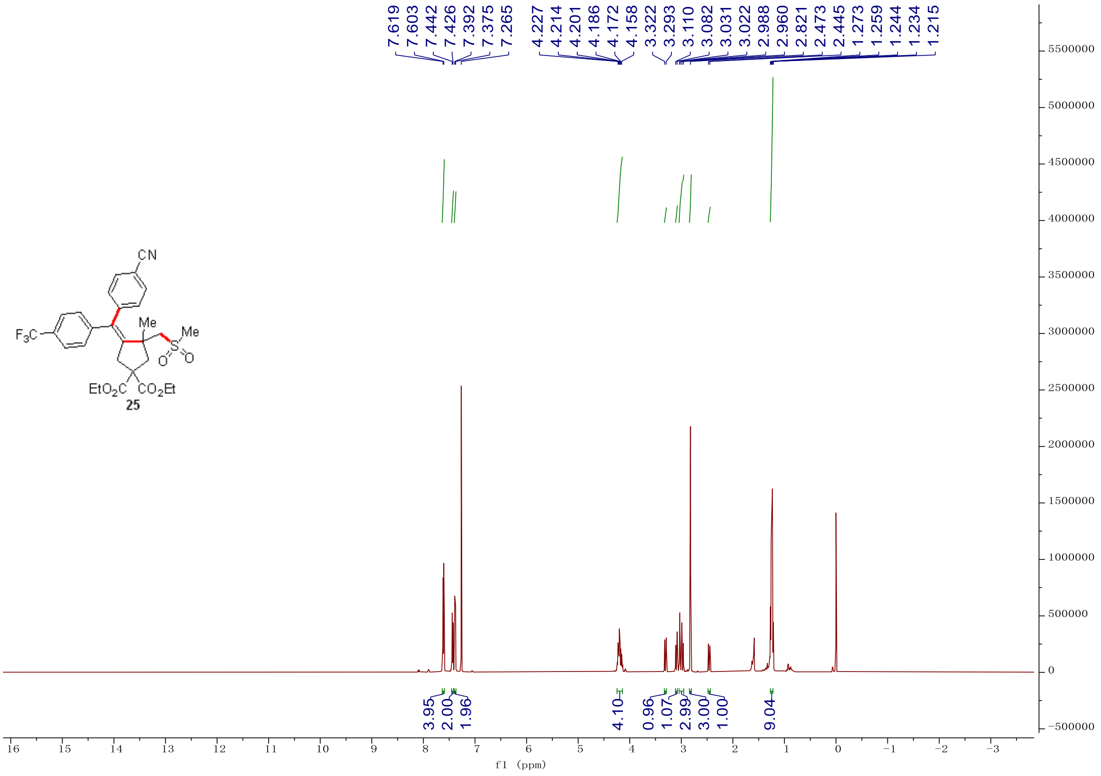

25: ^{13}C NMR (126 Hz, $CDCl_3$)

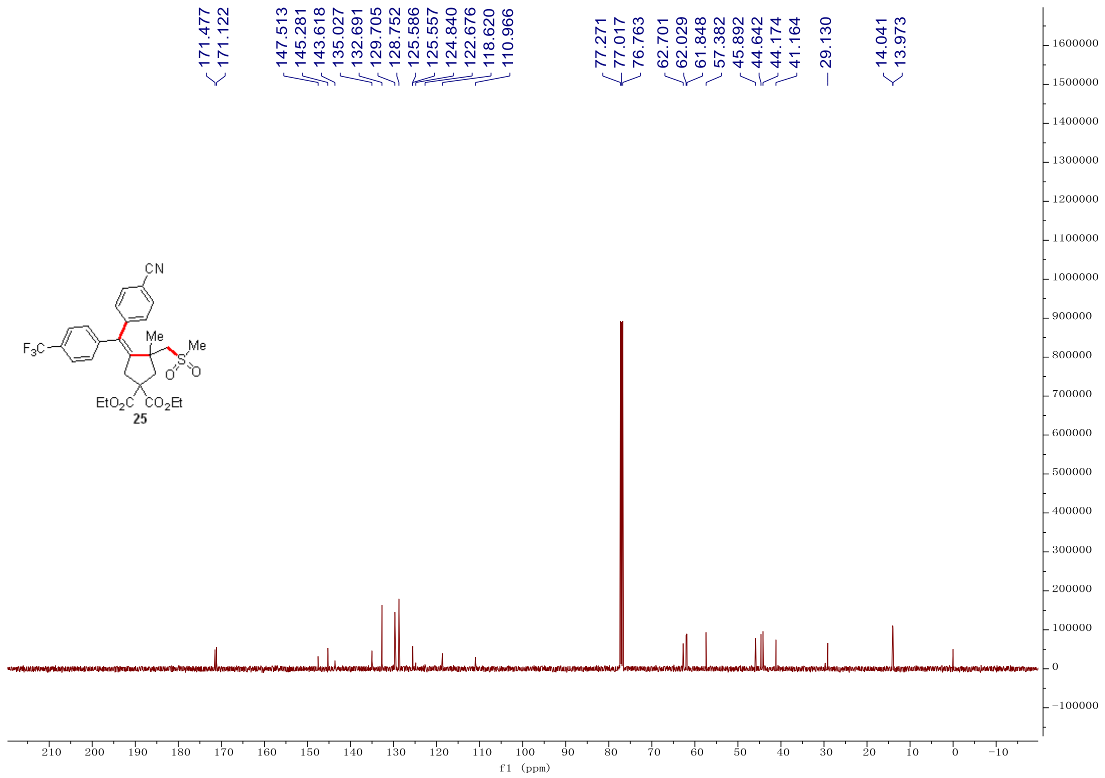

25: ^{19}F NMR (376 Hz, $CDCl_3$)

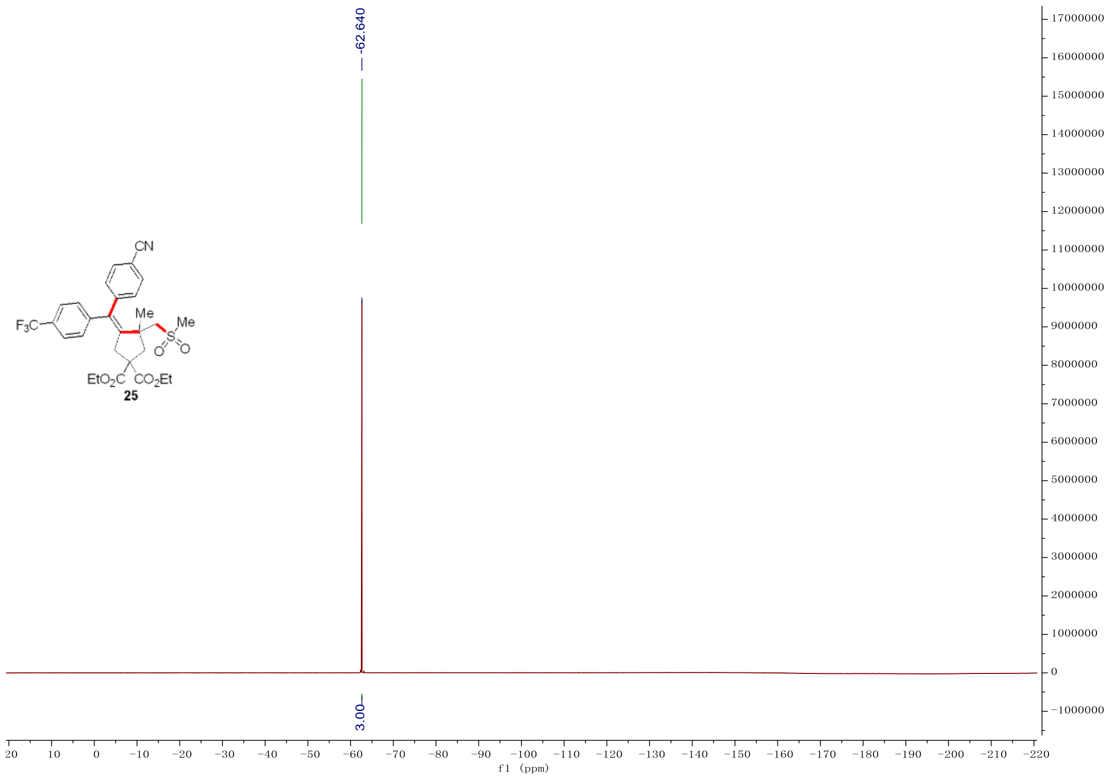

26: ^{1}H NMR (400 Hz, $CDCl_3$)

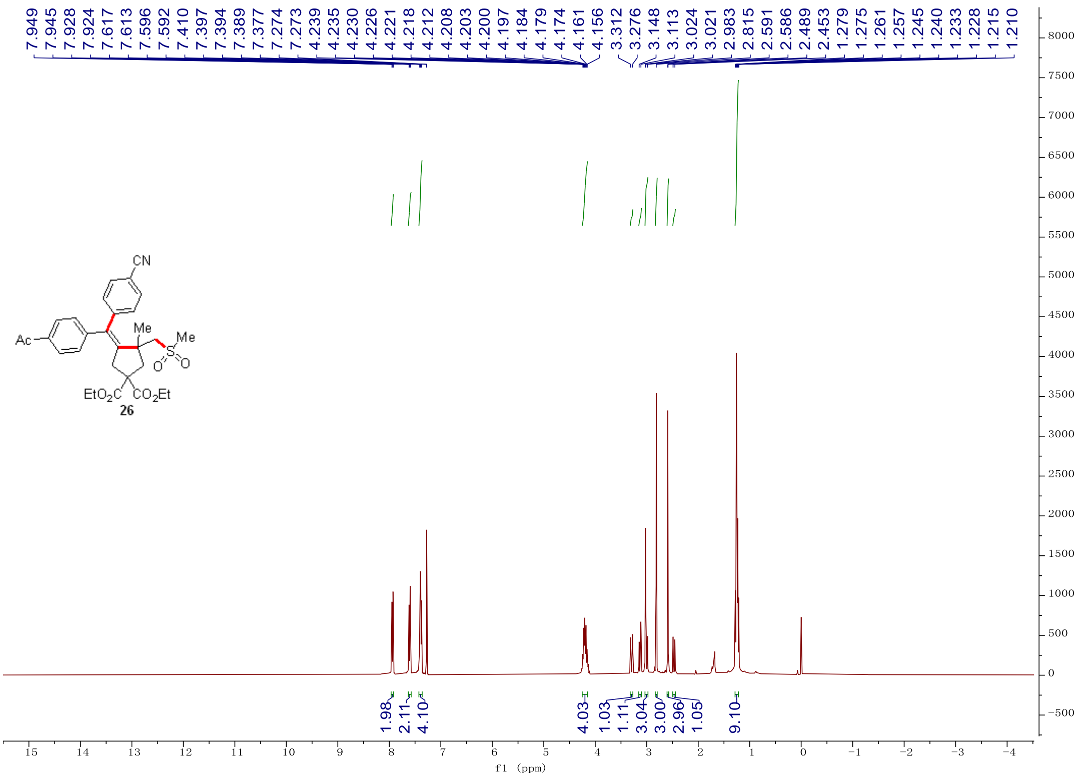

26: ^{13}C NMR (101 Hz, $CDCl_3$)

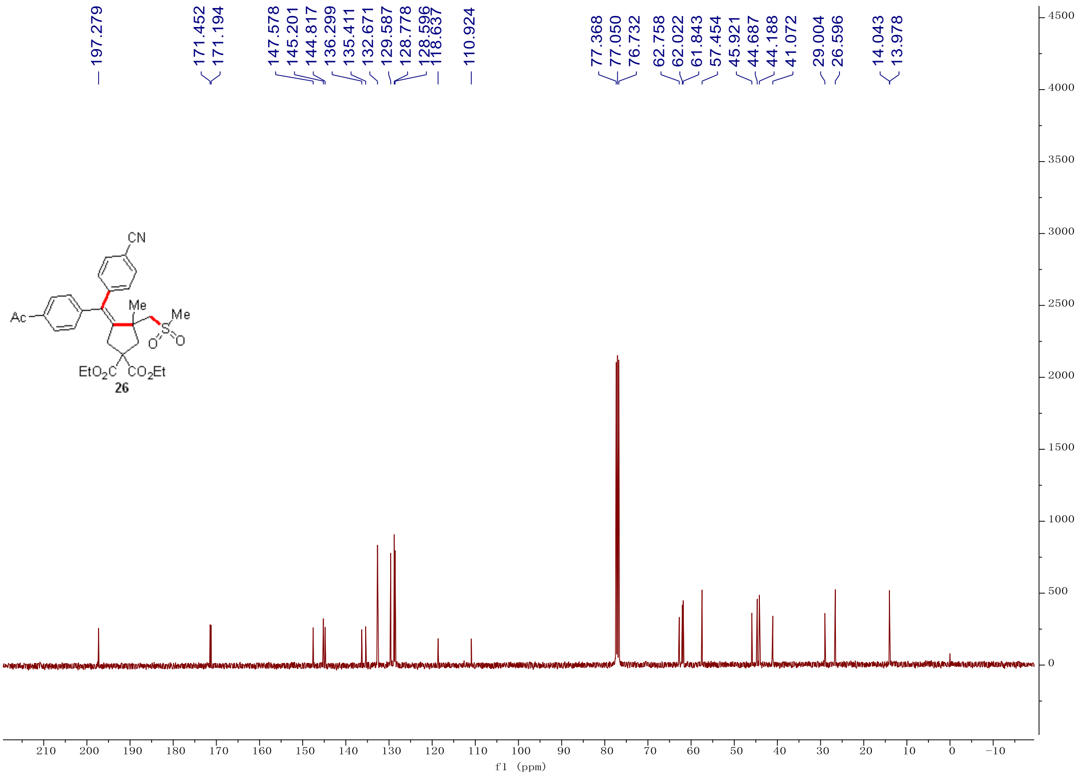

27: ^{1}H NMR (500 Hz, $CDCl_3$)

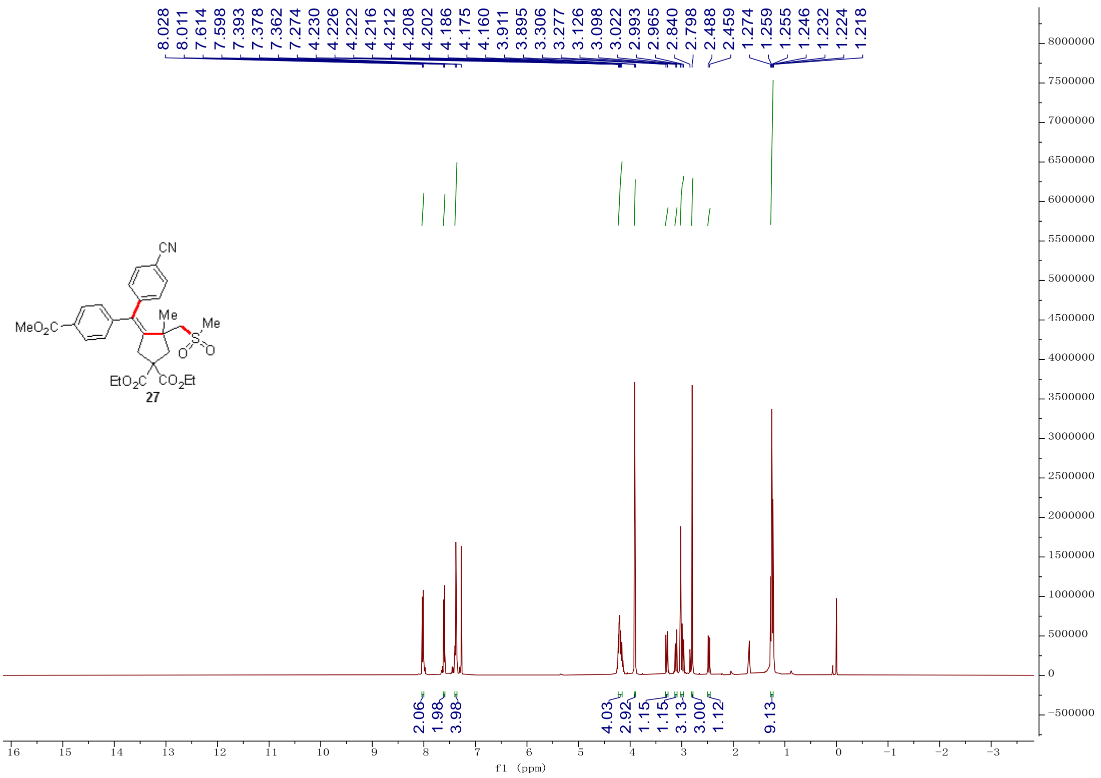

27: ^{13}C NMR (126 Hz, $CDCl_3$)

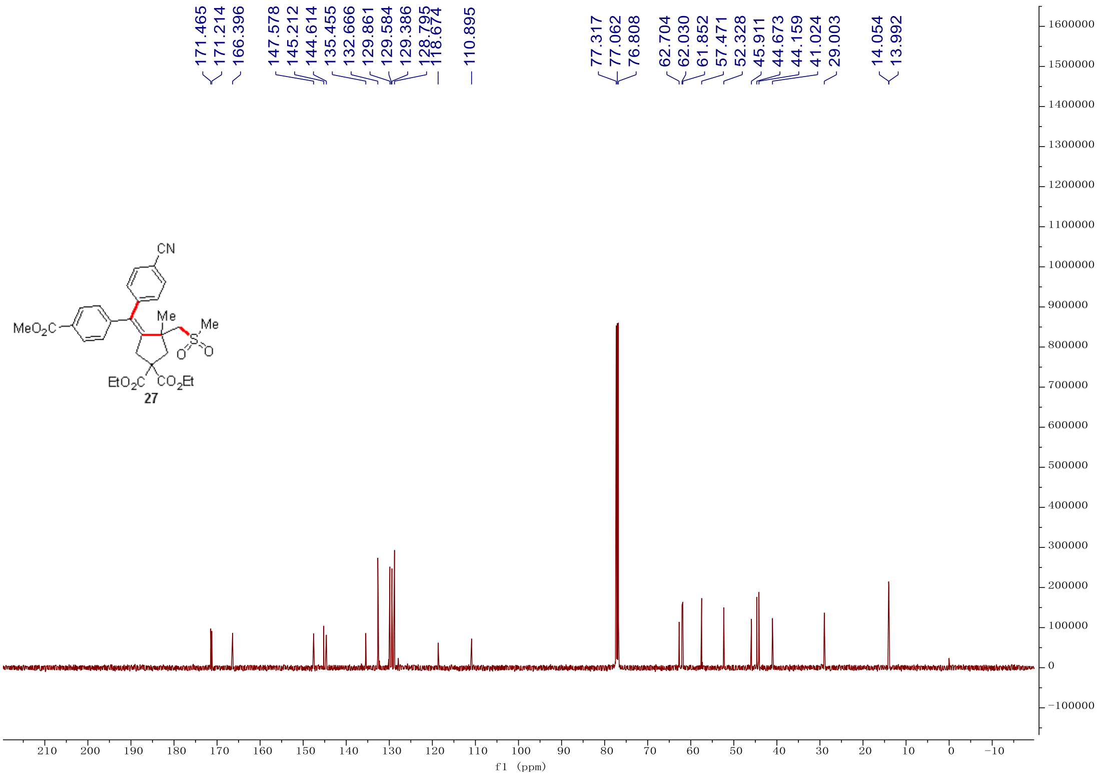

28: ^{1}H NMR (400 Hz, $CDCl_3$)

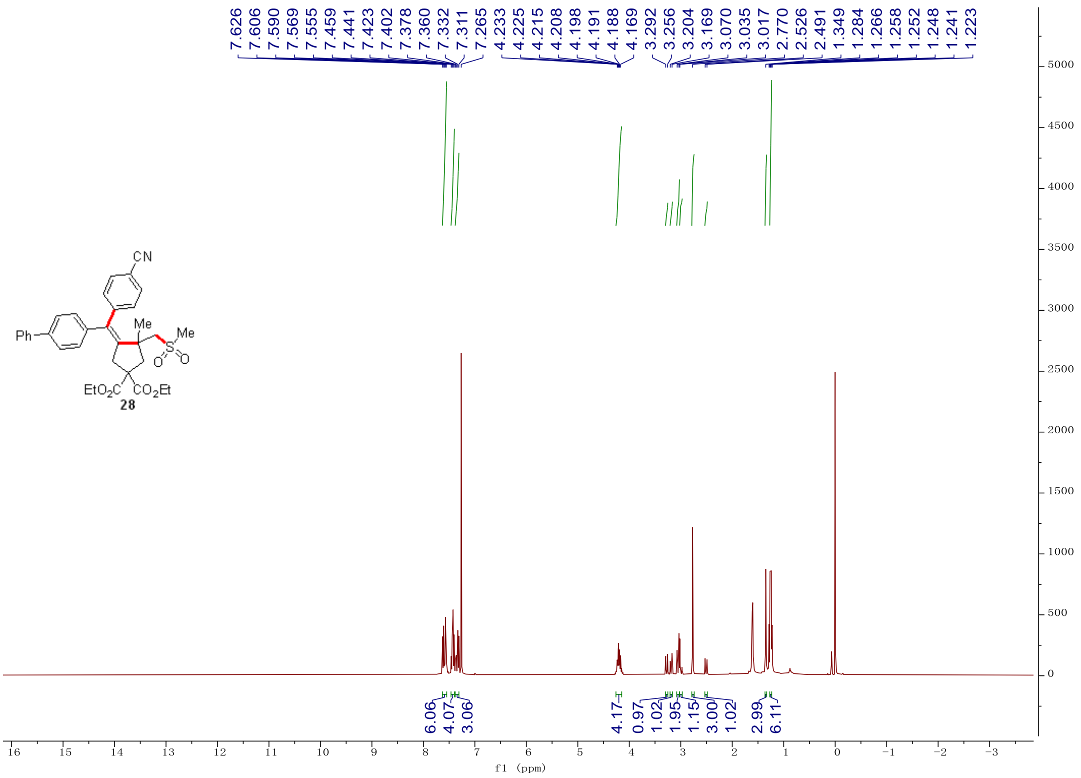

28: ^{13}C NMR (201 Hz, $CDCl_3$)

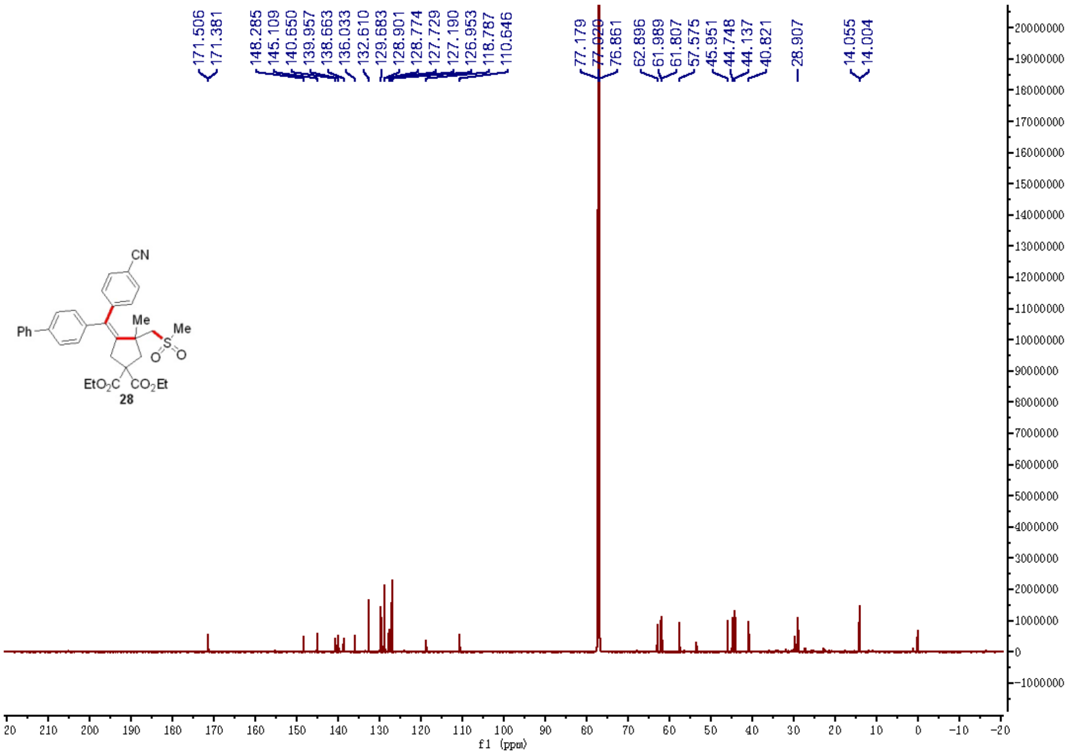

29: ^{1}H NMR (800 Hz, $CDCl_3$)

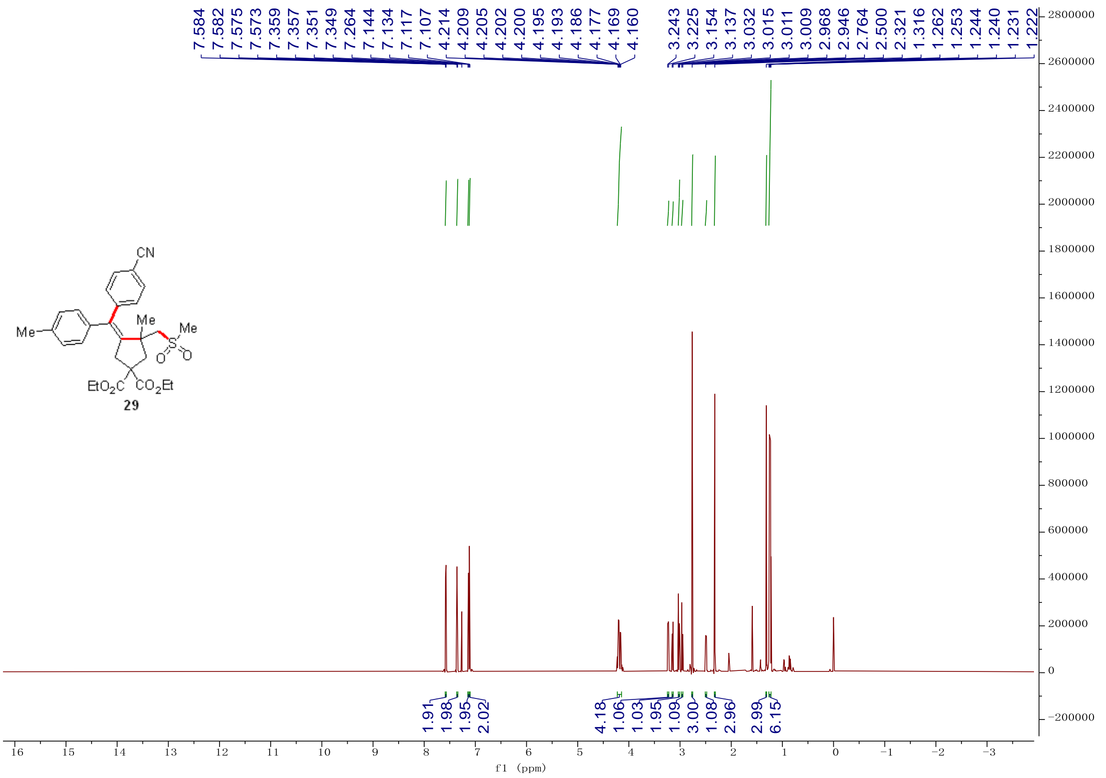

29: ^{13}C NMR (201 Hz, $CDCl_3$)

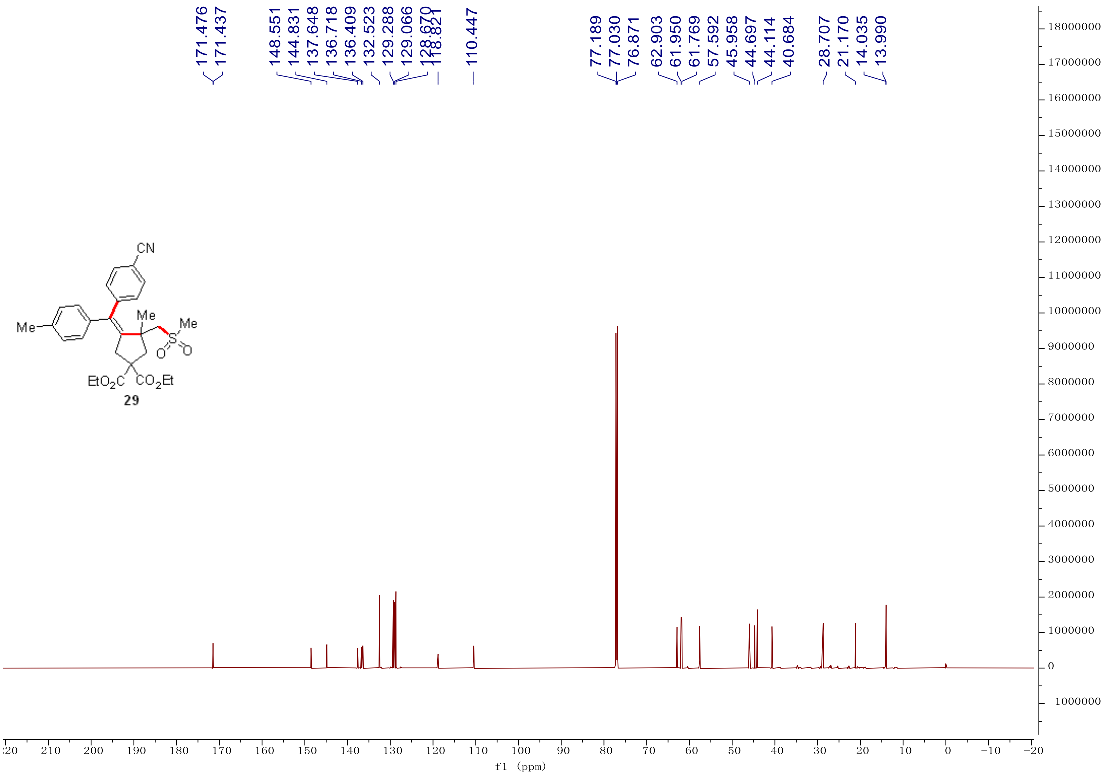

30: ^{1}H NMR (400 Hz, $CDCl_3$)

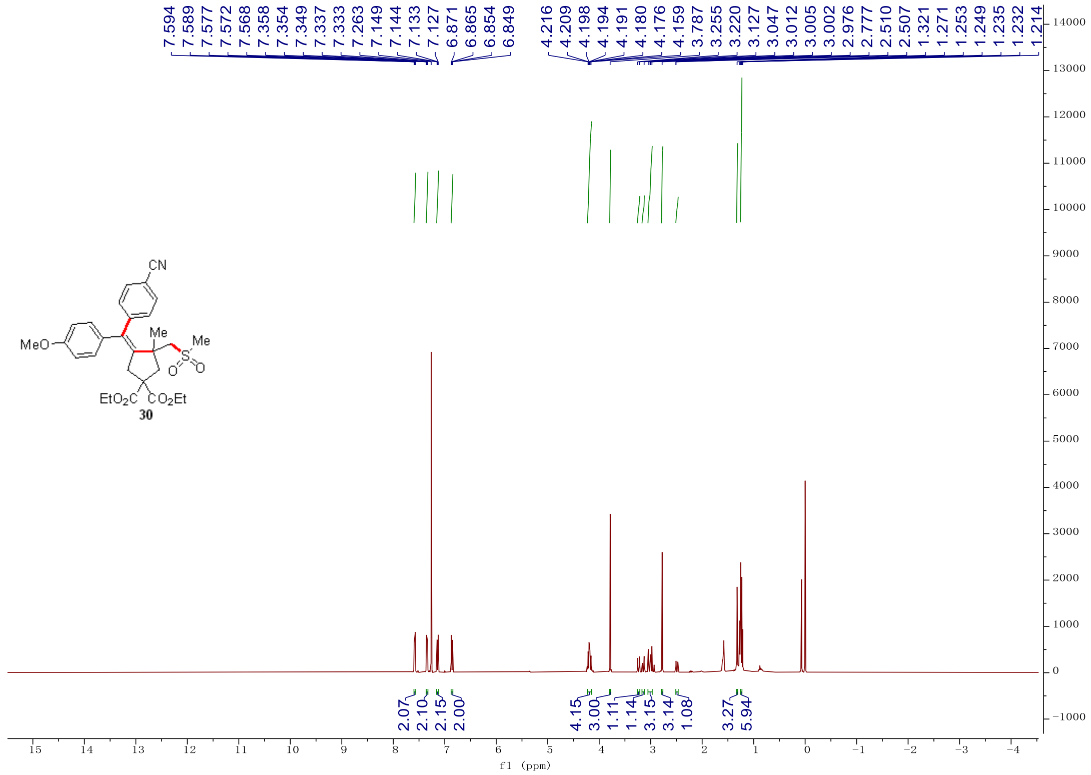

30: ^{13}C NMR (101 Hz, $CDCl_3$)

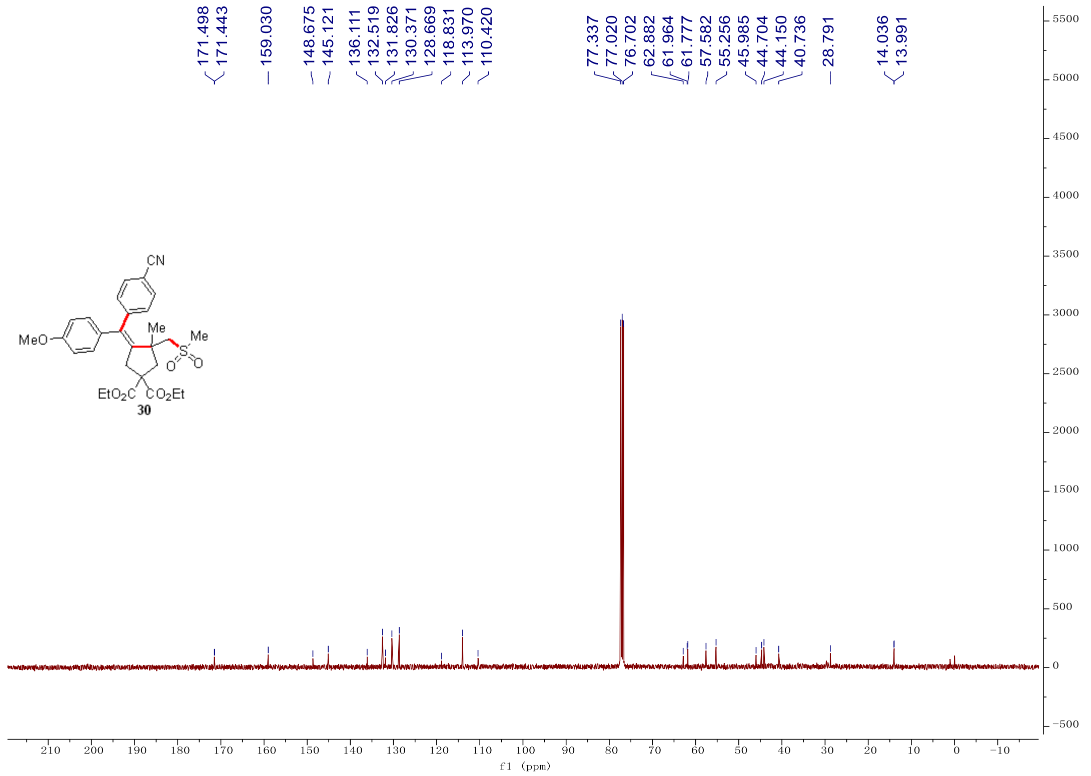

31: ^{1}H NMR (400 Hz, $CDCl_3$)

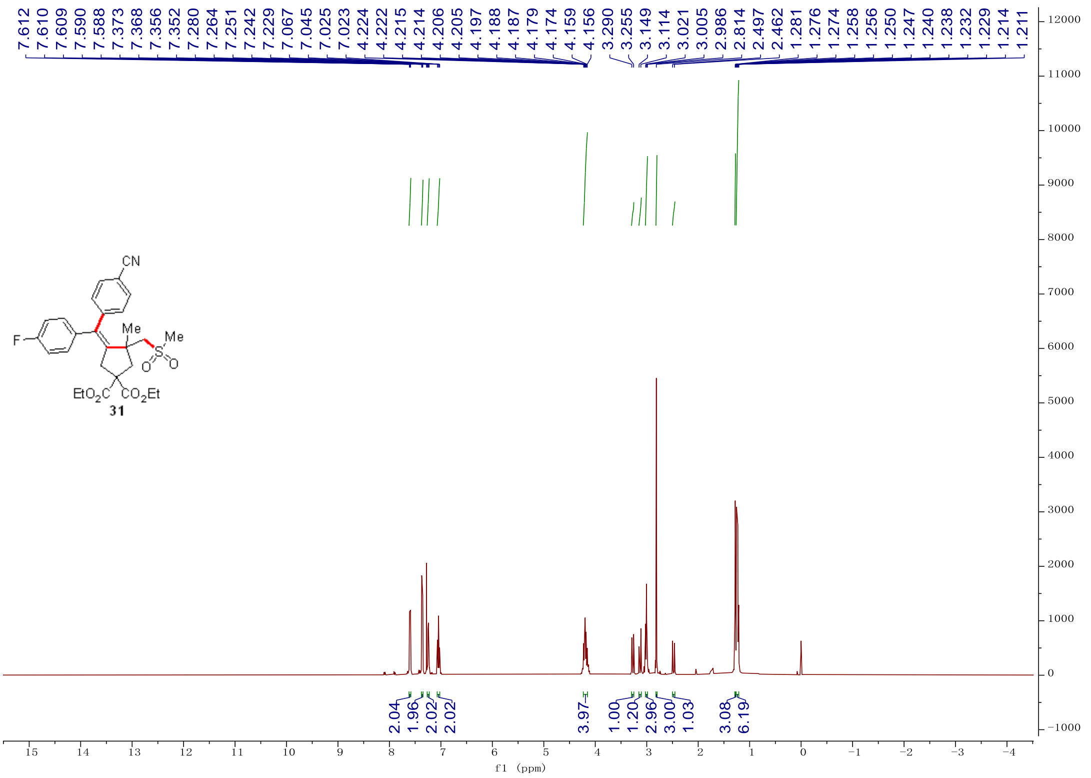

31: ^{13}C NMR (101 Hz, $CDCl_3$)

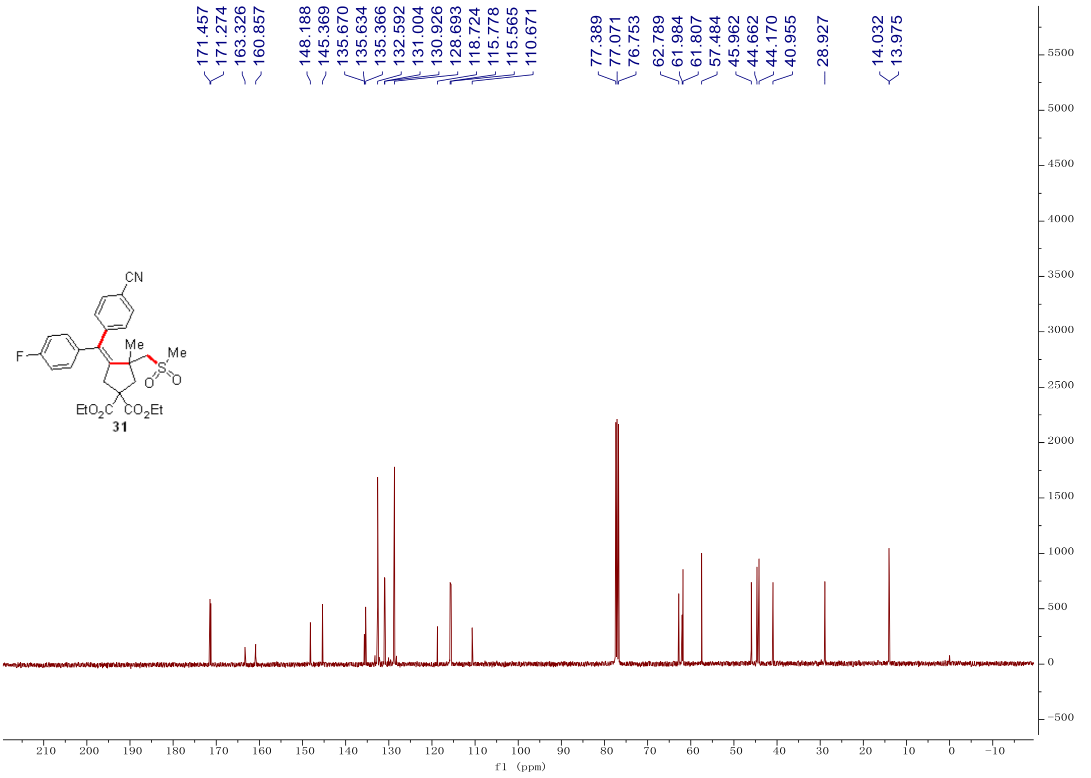

31: ^{19}F NMR (376 Hz, CDCl$_3$)

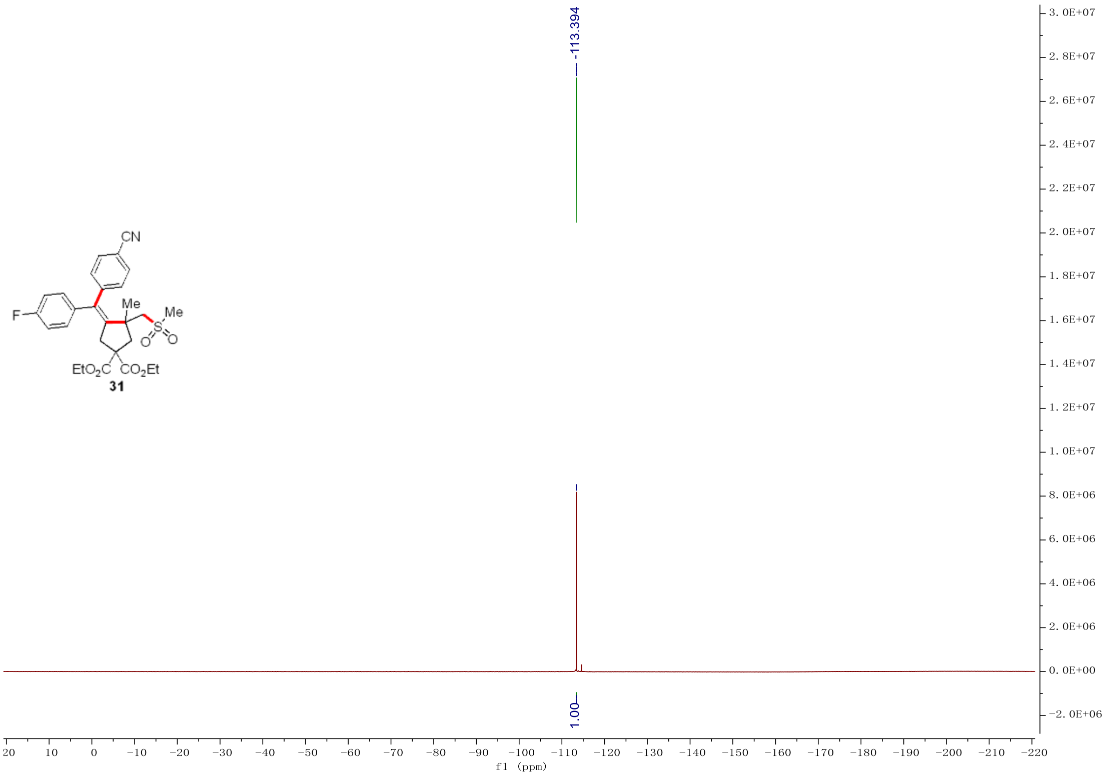

32: ^{1}H NMR (400 Hz, CDCl$_3$)

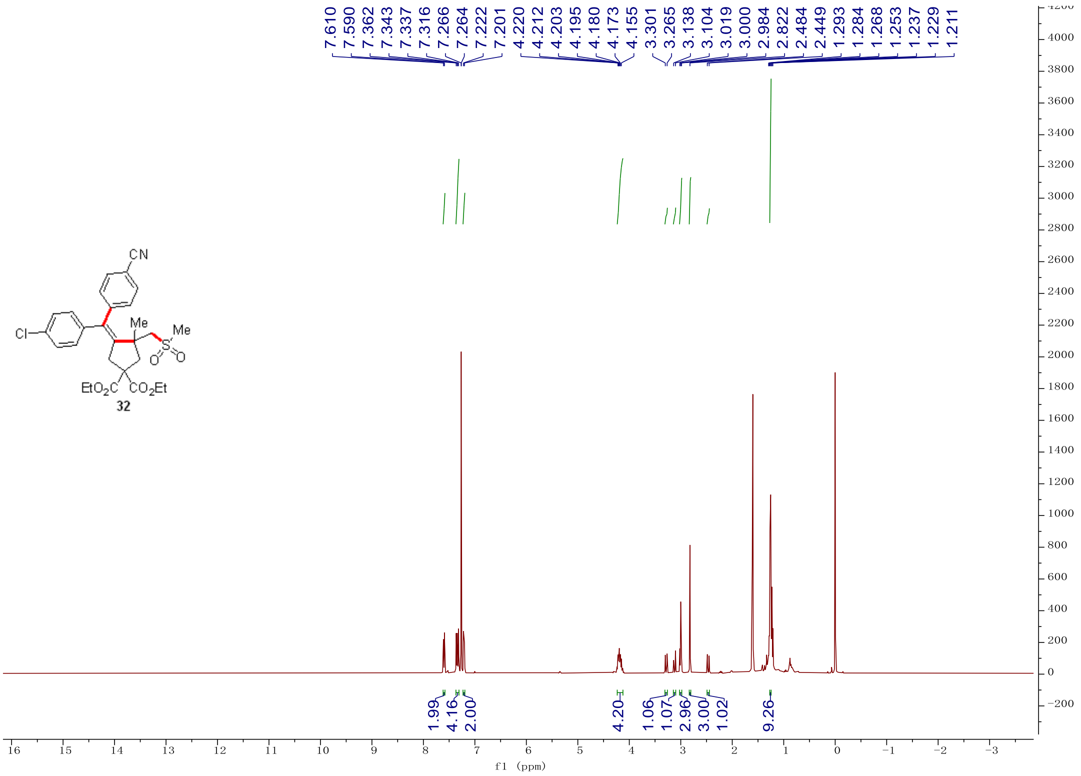

32: ^{13}C NMR (101 Hz, $CDCl_3$)

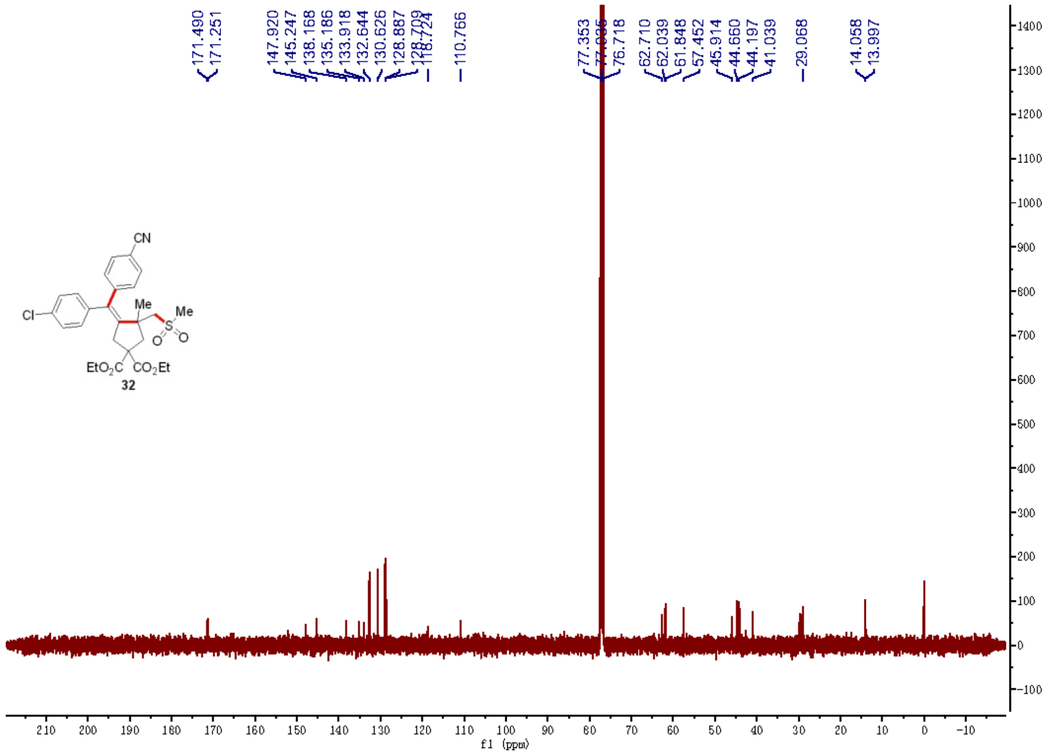

33: ^{1}H NMR (400 Hz, $CDCl_3$)

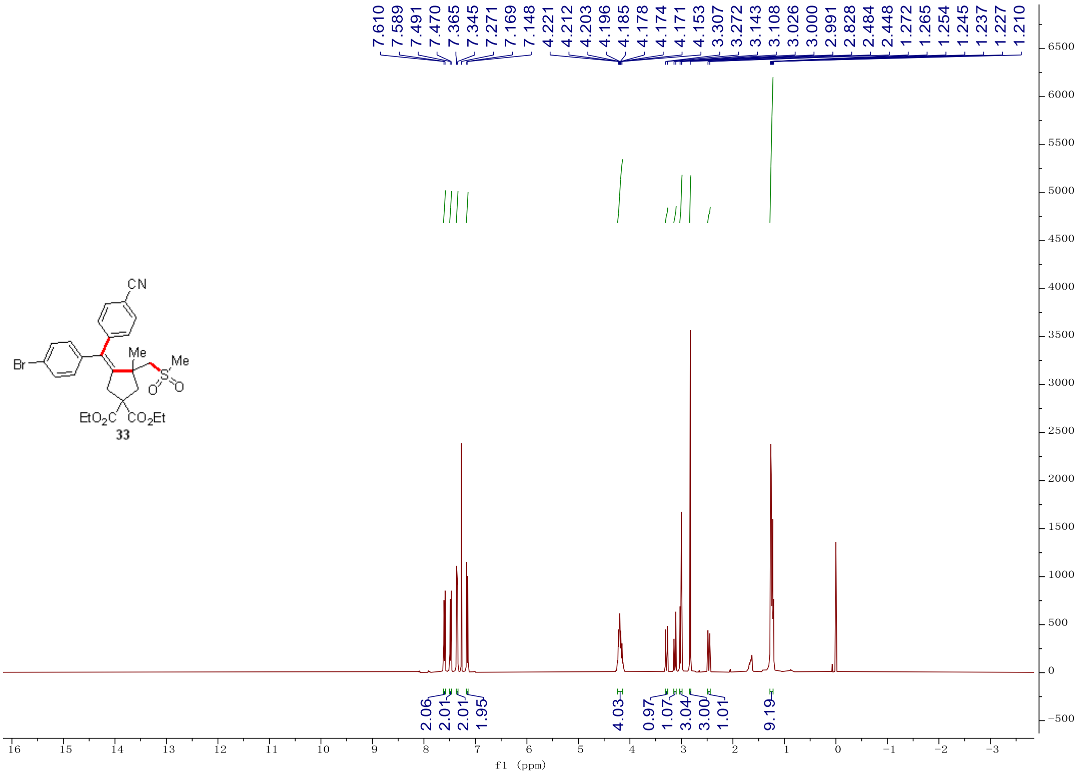

33: ^{13}C NMR (101 Hz, $CDCl_3$)

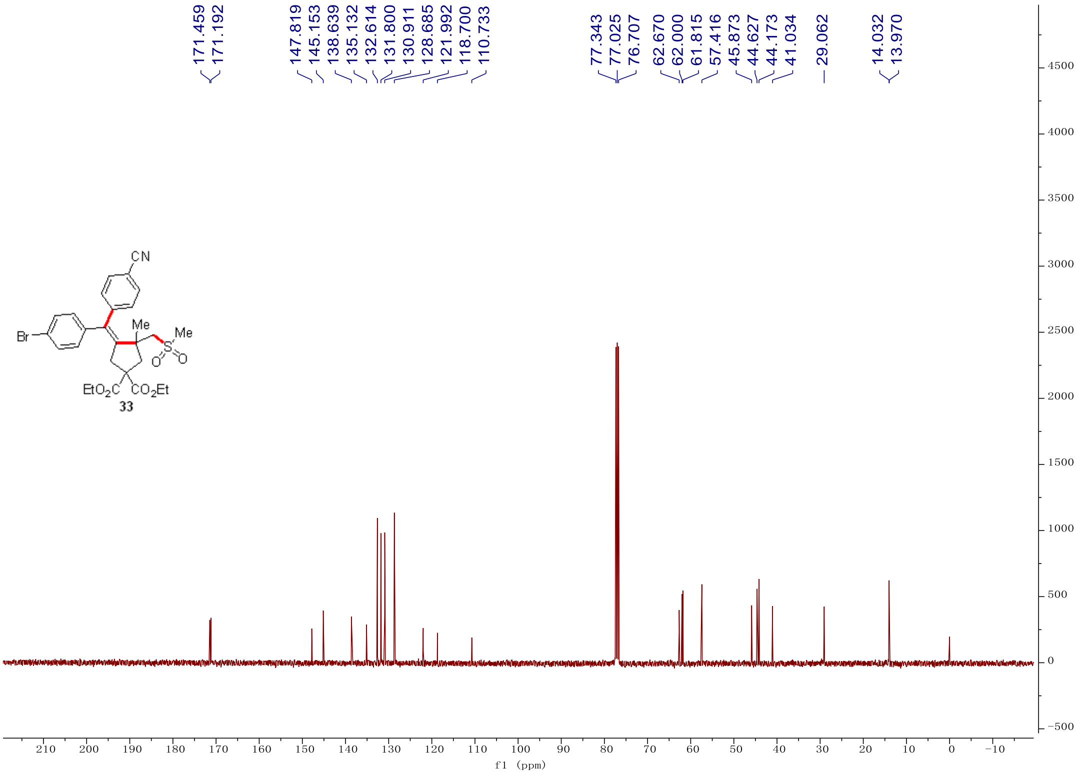

34: ^{1}H NMR (400 Hz, $CDCl_3$)

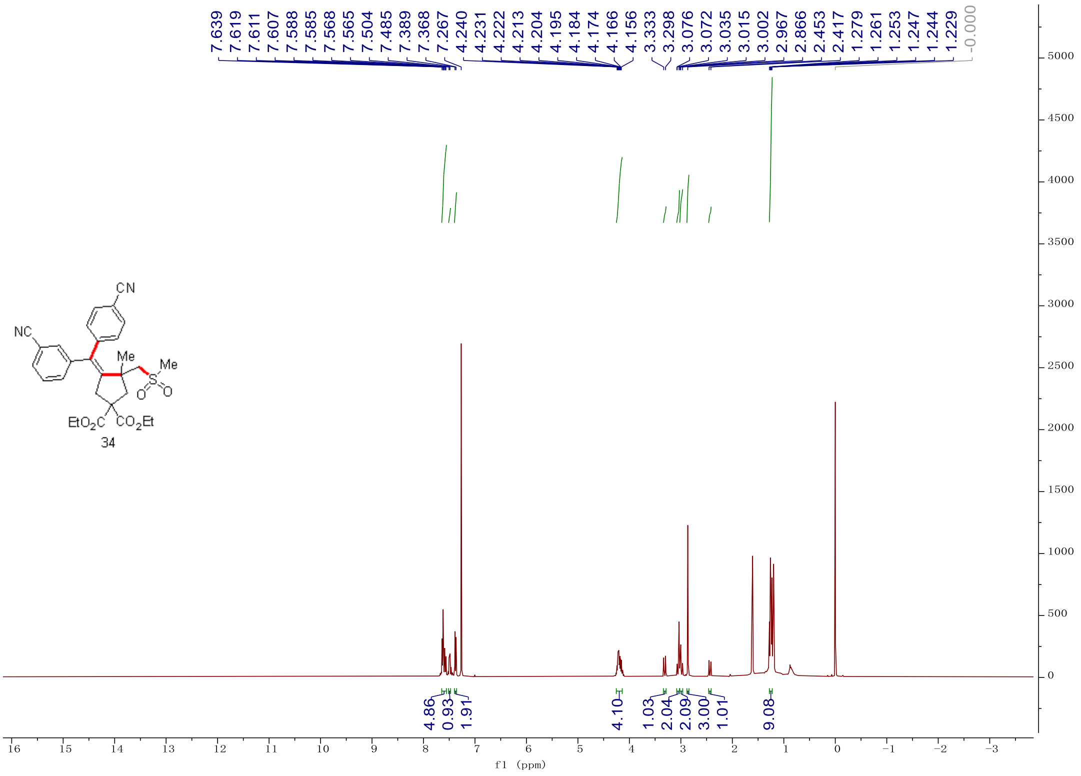

34: ^{13}C NMR (101 Hz, CDCl$_3$)

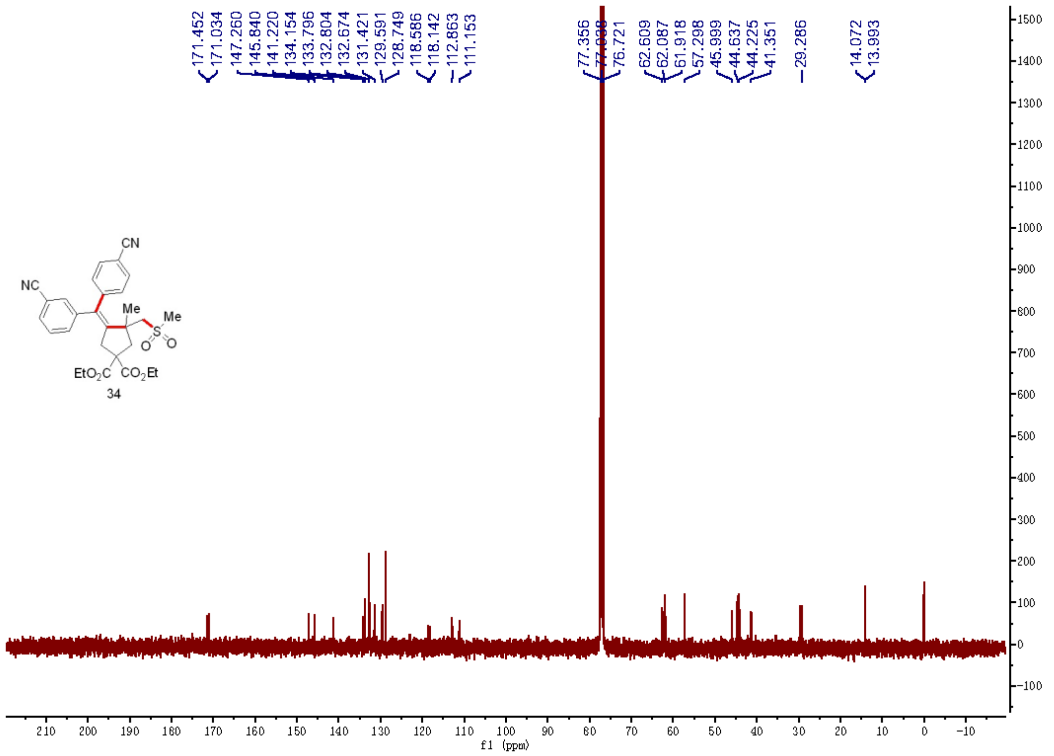

35: ^{1}H NMR (400 Hz, CDCl$_3$)

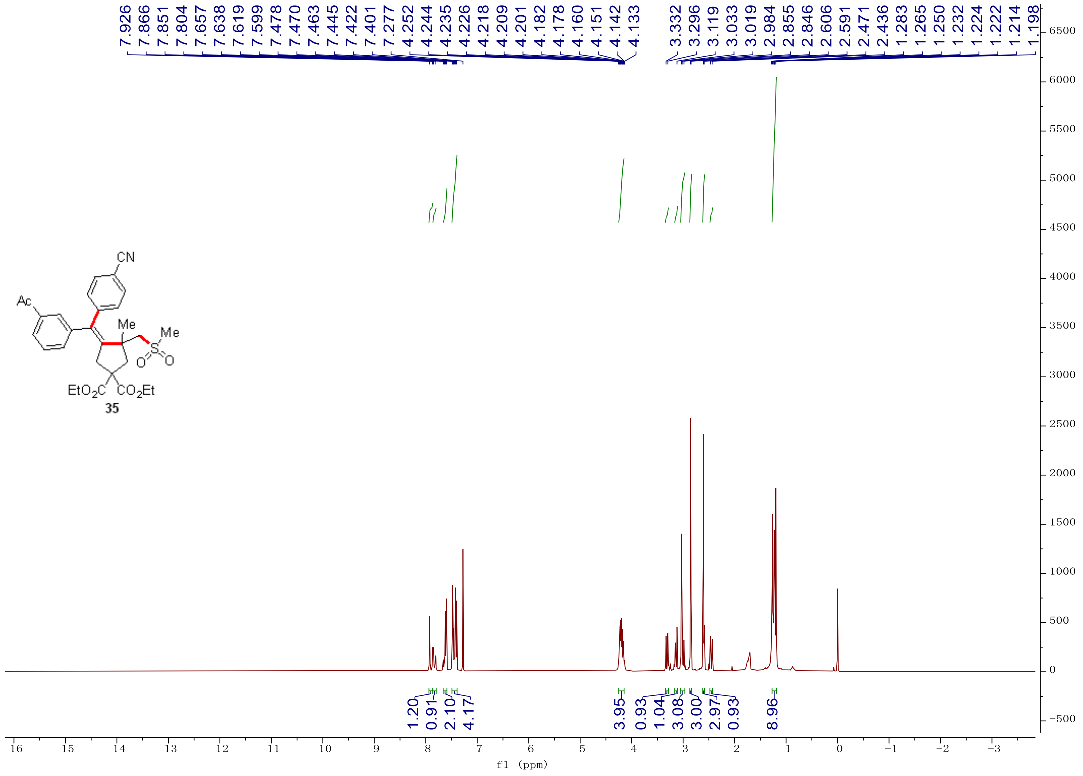

35: ^{13}C NMR (101 Hz, $CDCl_3$)

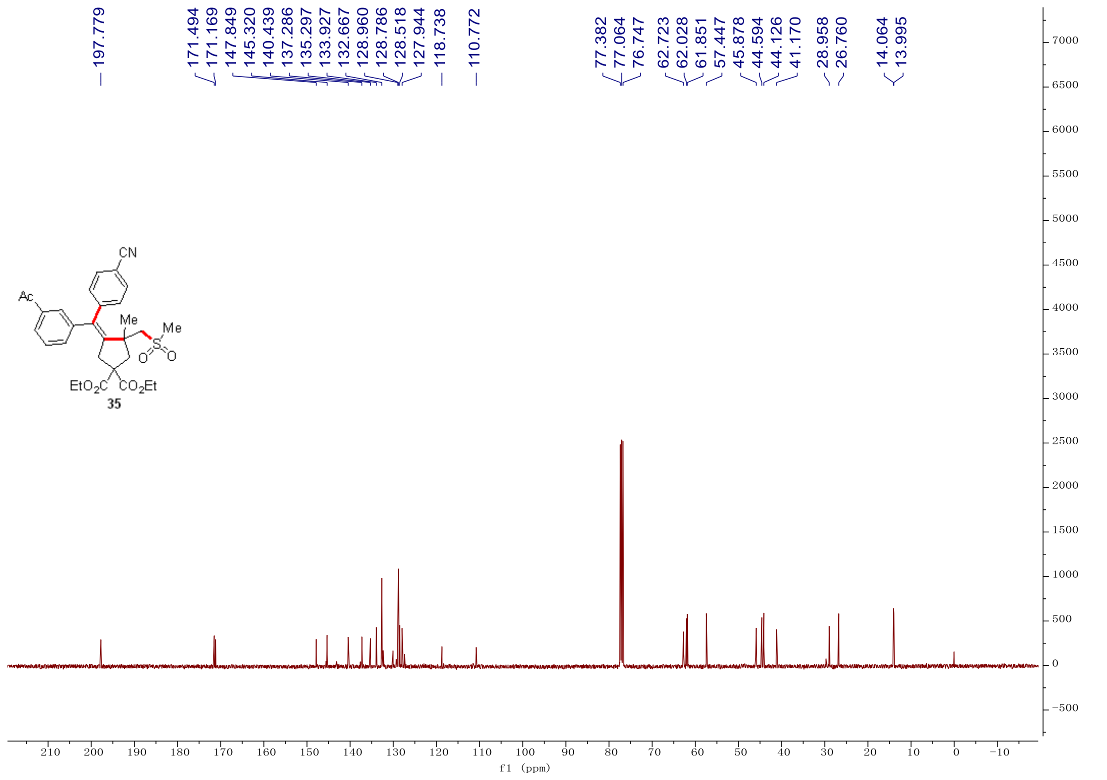

36: ^{1}H NMR (500 Hz, $CDCl_3$)

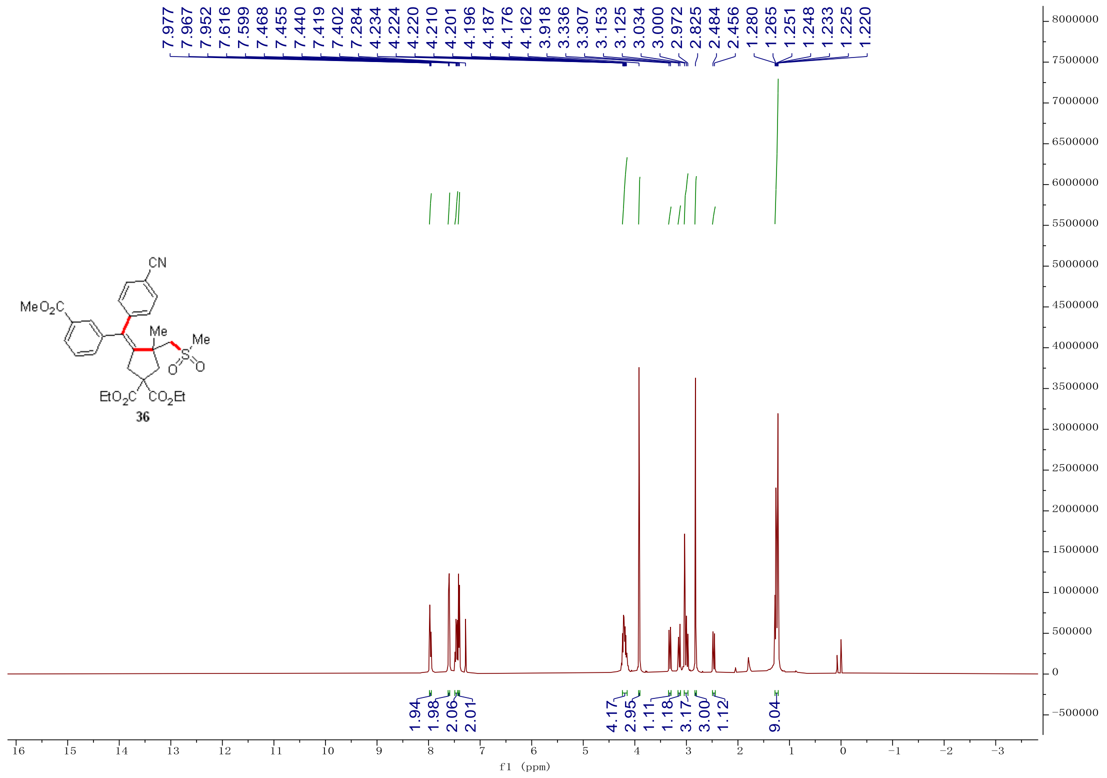

36: ^{13}C NMR (126 Hz, $CDCl_3$)

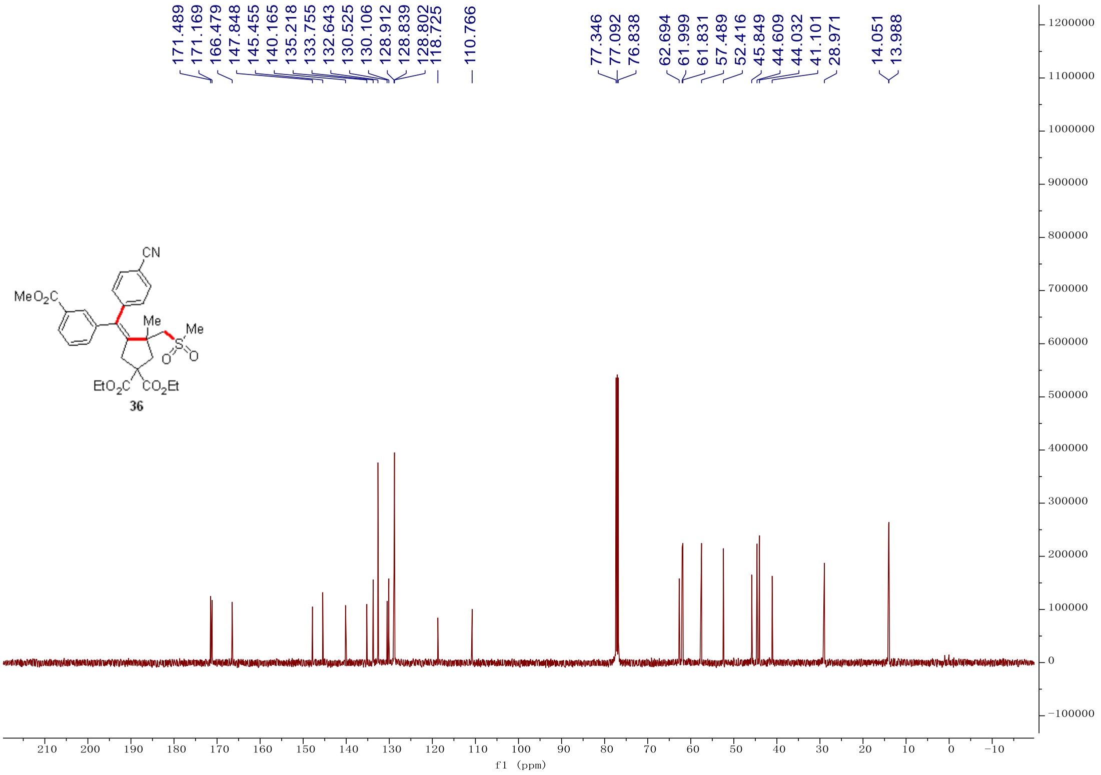

37: ^{1}H NMR (800 Hz, $CDCl_3$)

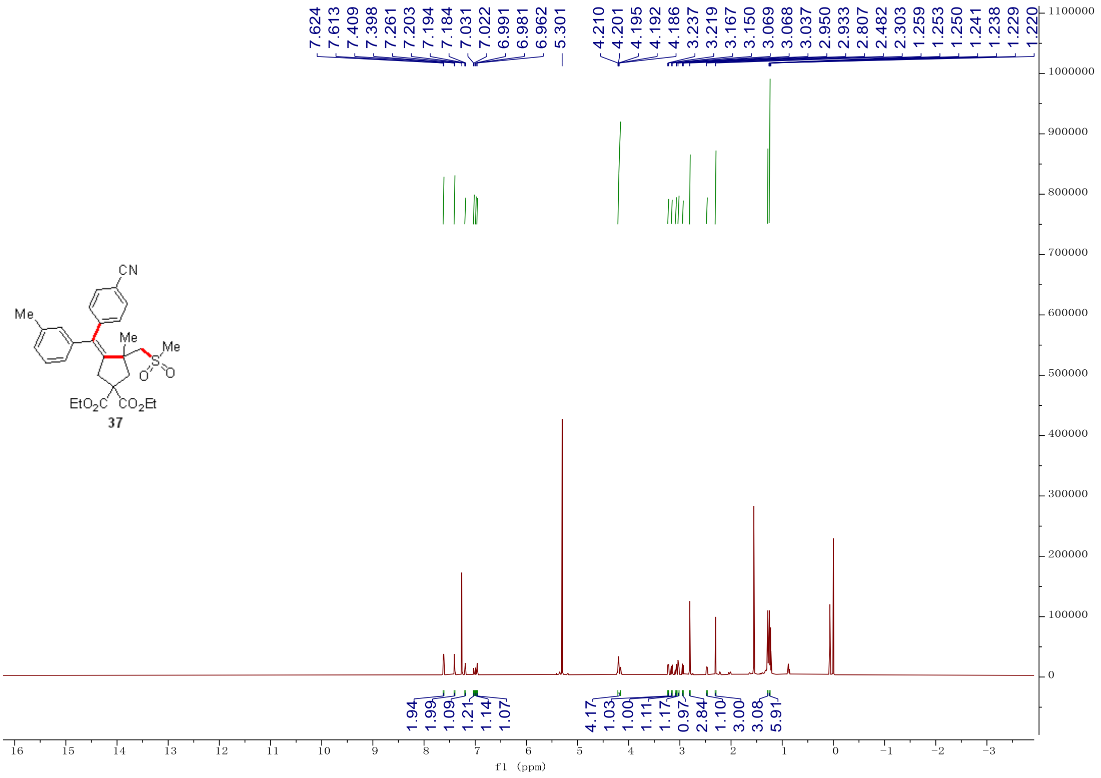

37: ^{13}C NMR (201 Hz, CDCl$_3$)

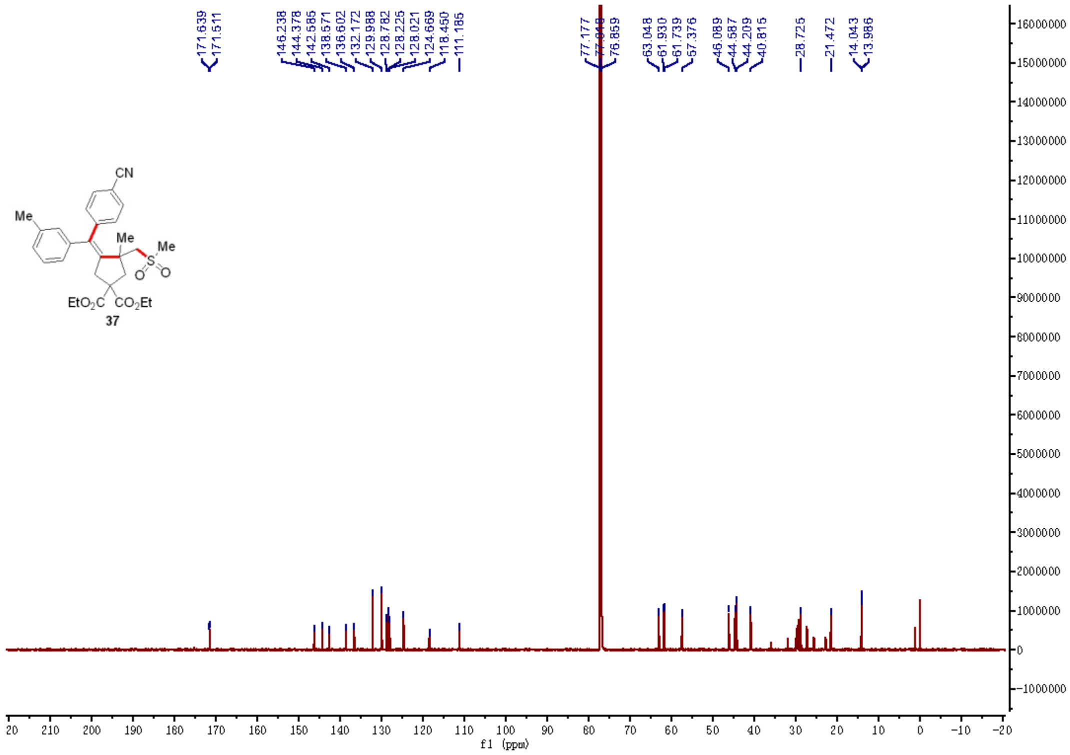

37

38: ^{1}H NMR (400 Hz, CDCl$_3$)

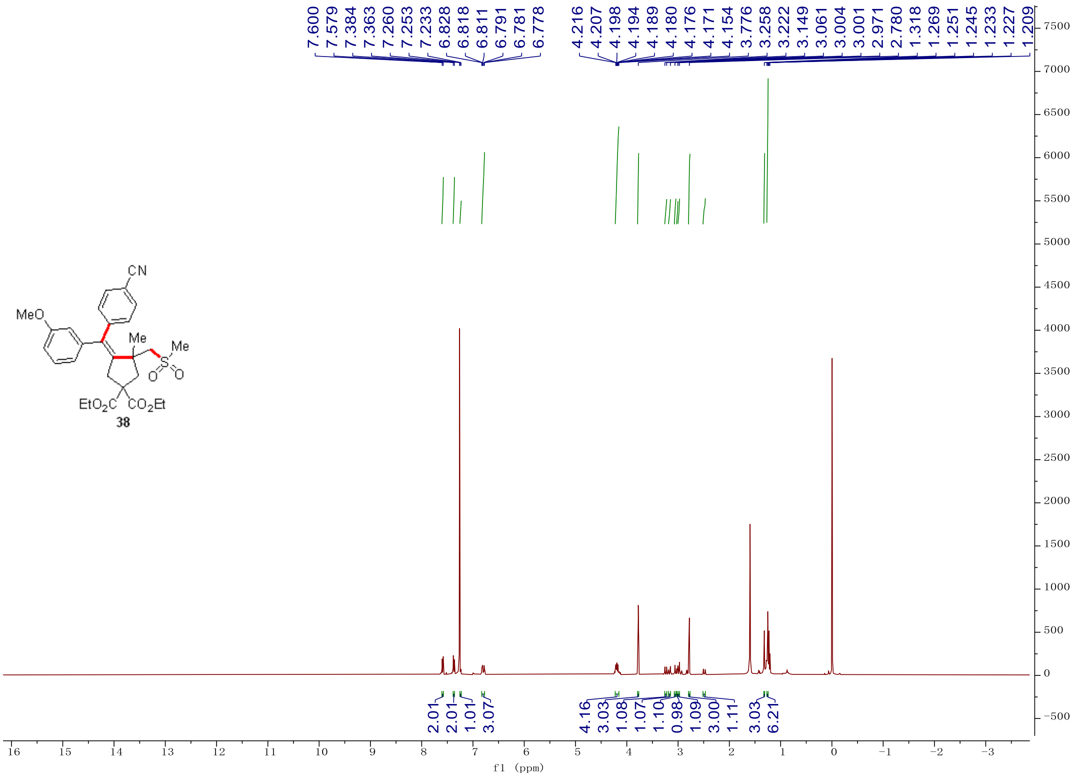

38

38: ^{13}C NMR (201 Hz, $CDCl_3$)

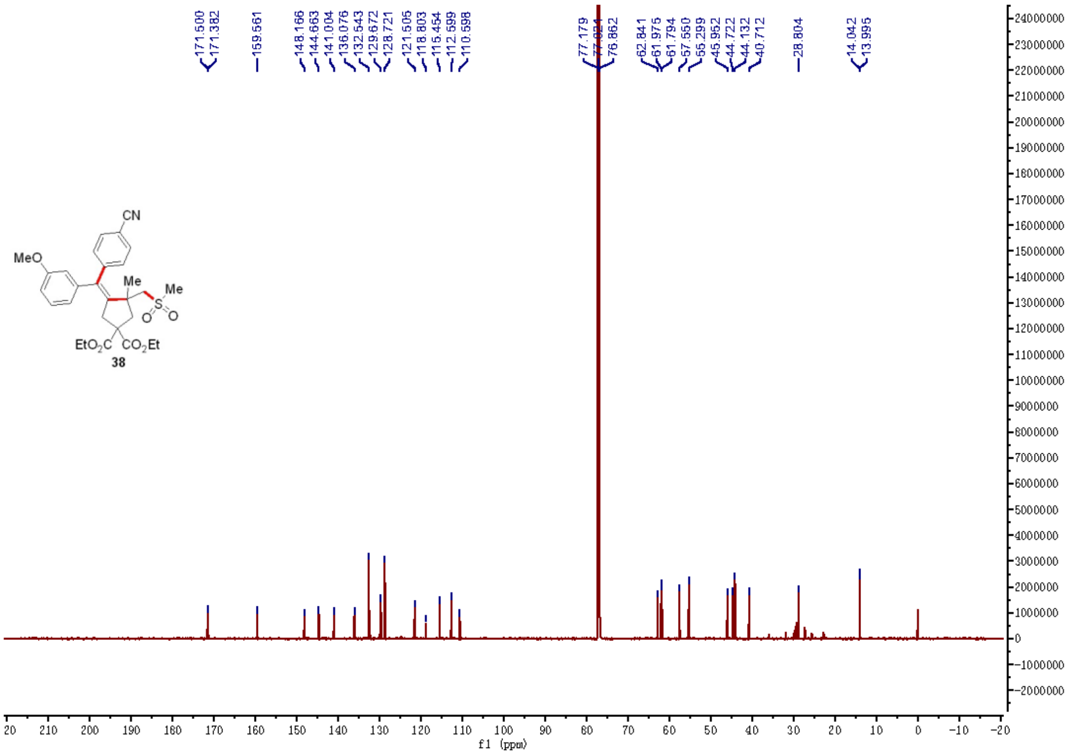

39: ^{1}H NMR (400 Hz, $CDCl_3$)

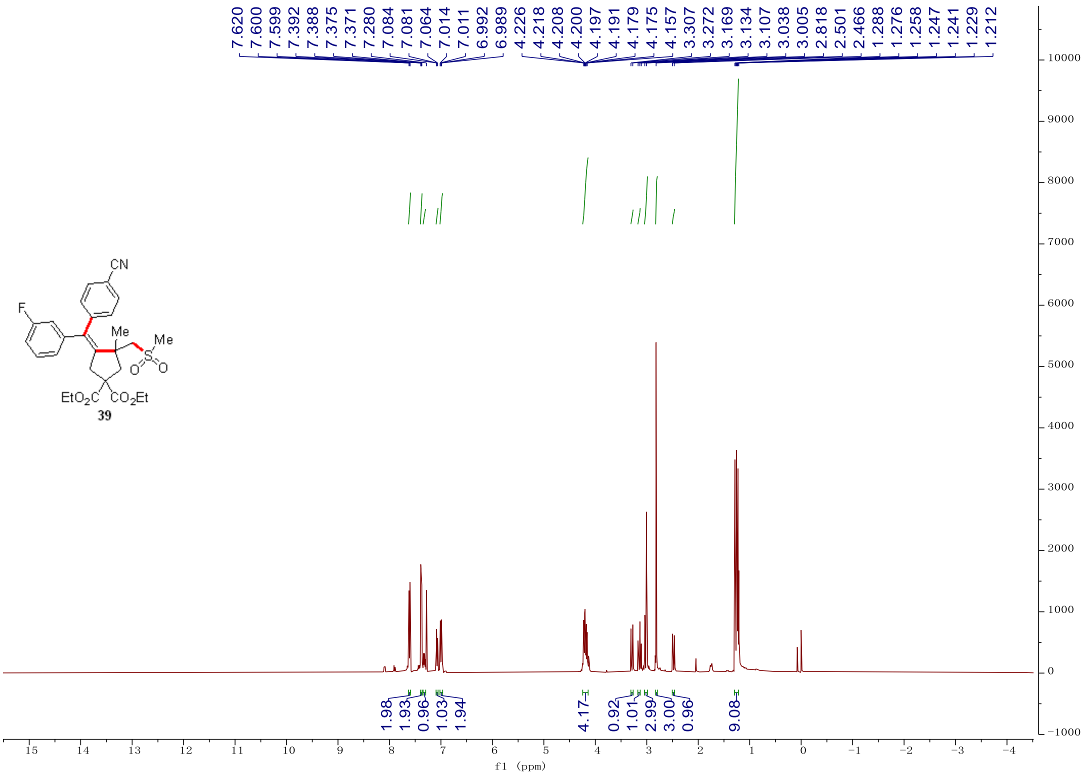

39: ^{13}C NMR (101 Hz, $CDCl_3$)

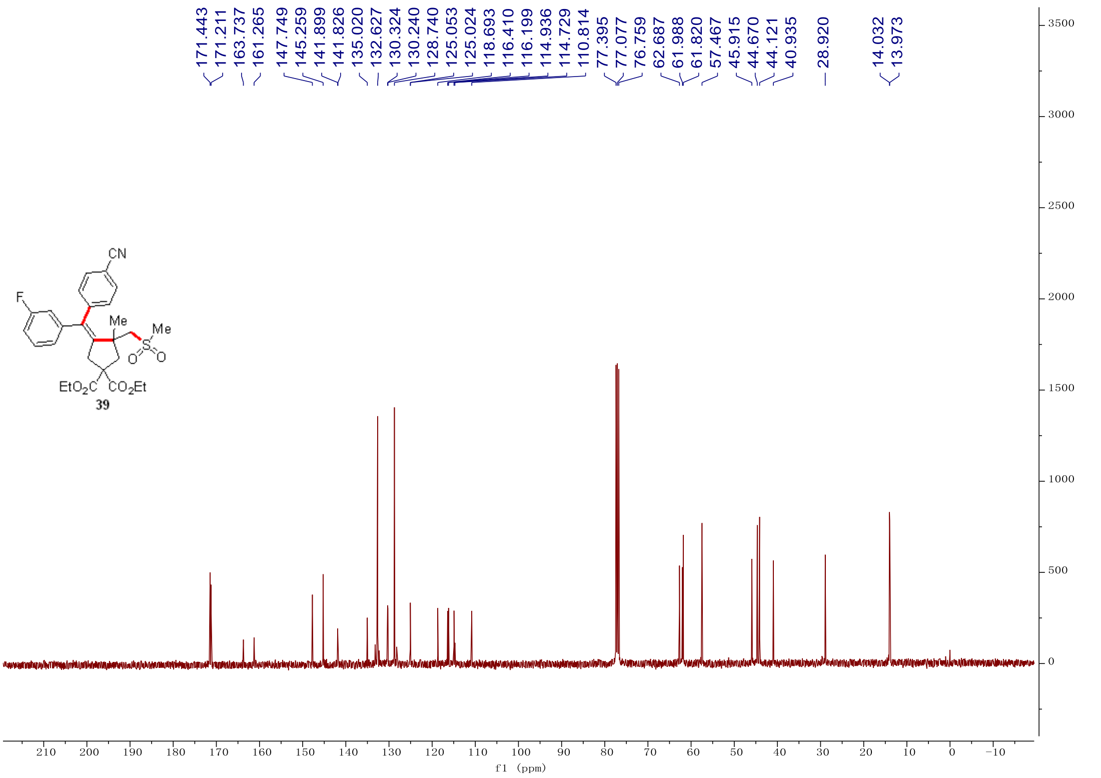

39: ^{19}F NMR (376 Hz, $CDCl_3$)

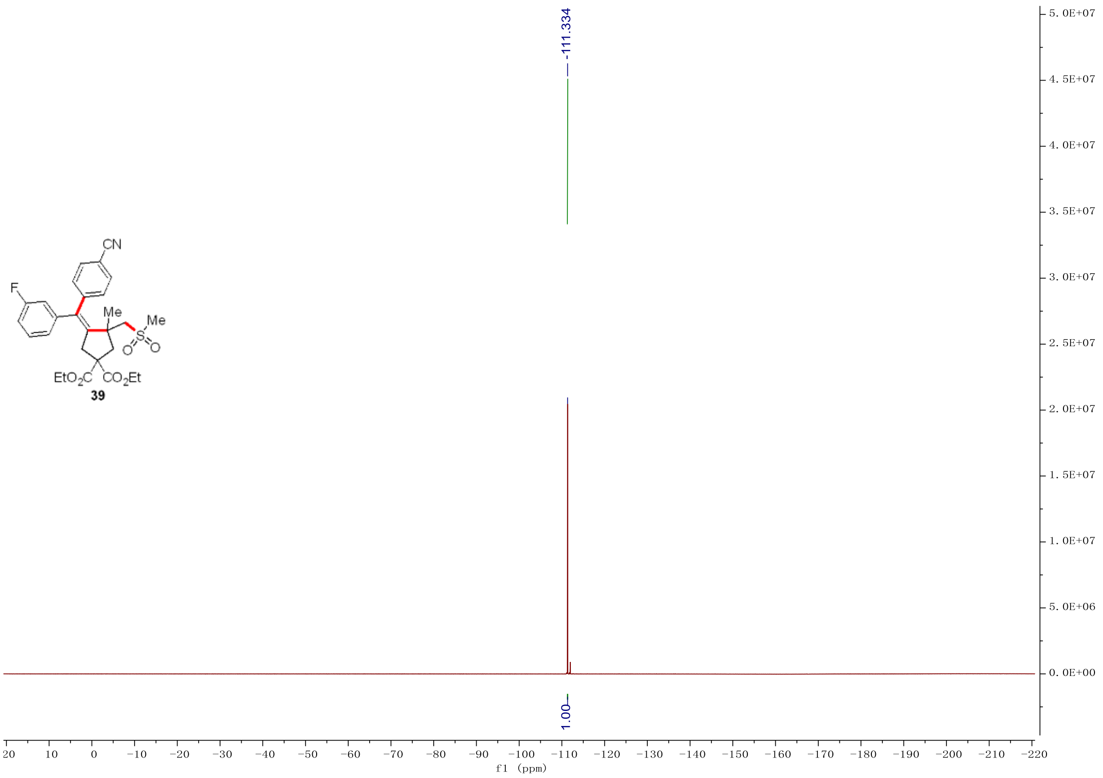

40: ^{1}H NMR (400 Hz, $CDCl_3$)

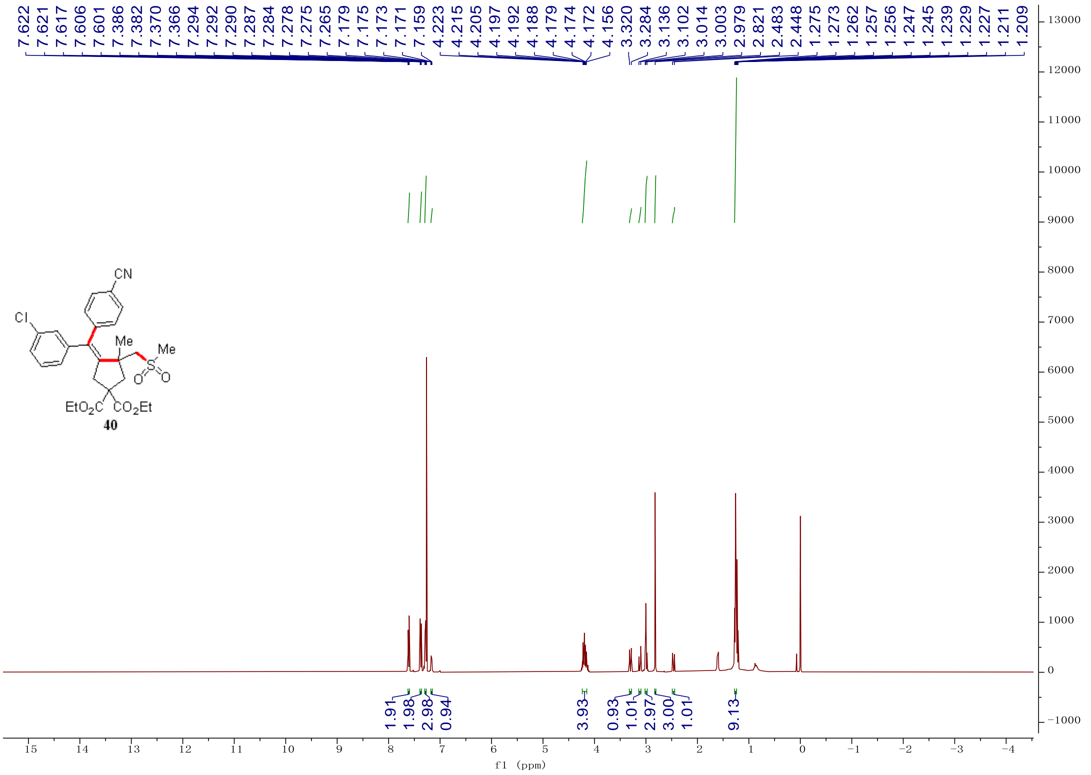

40: ^{13}C NMR (101 Hz, $CDCl_3$)

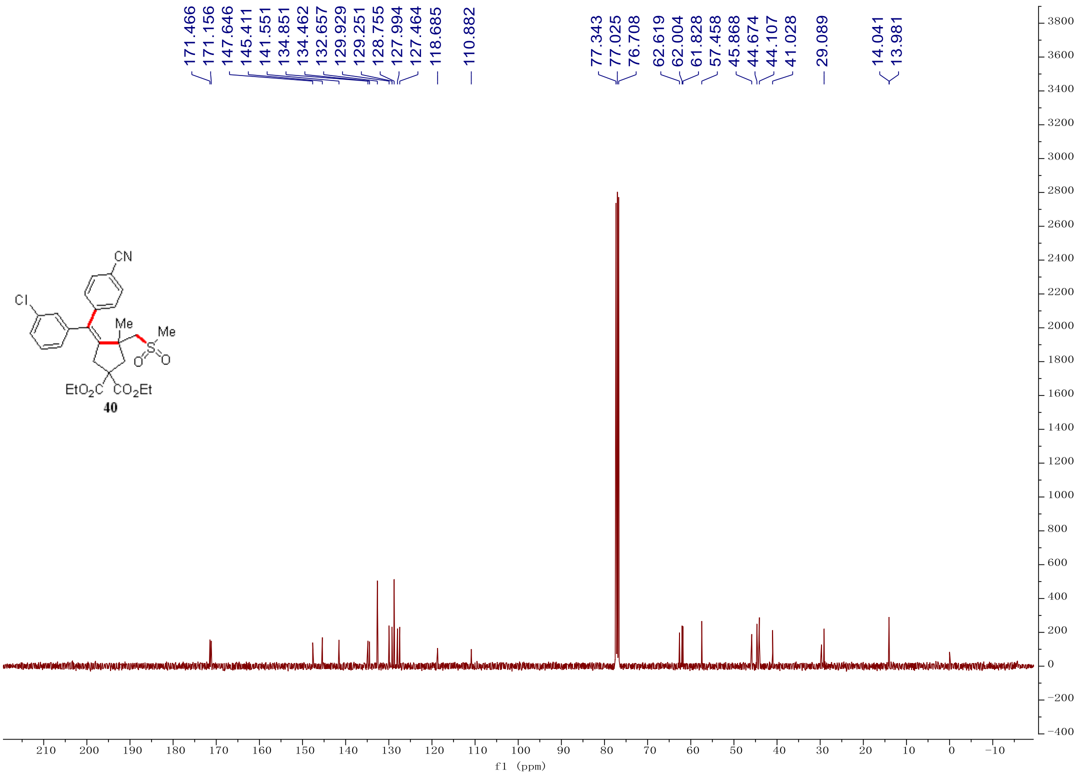

41: ^{1}H NMR (500 Hz, $CDCl_3$)

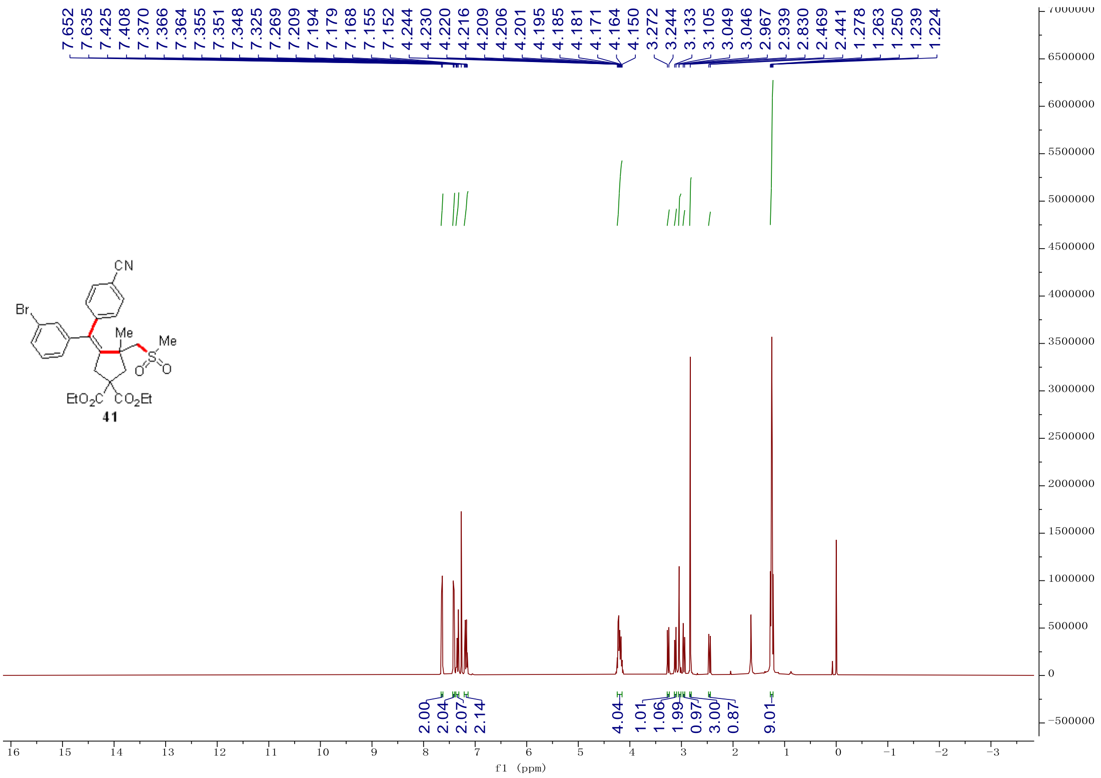

41: ^{13}C NMR (126 Hz, $CDCl_3$)

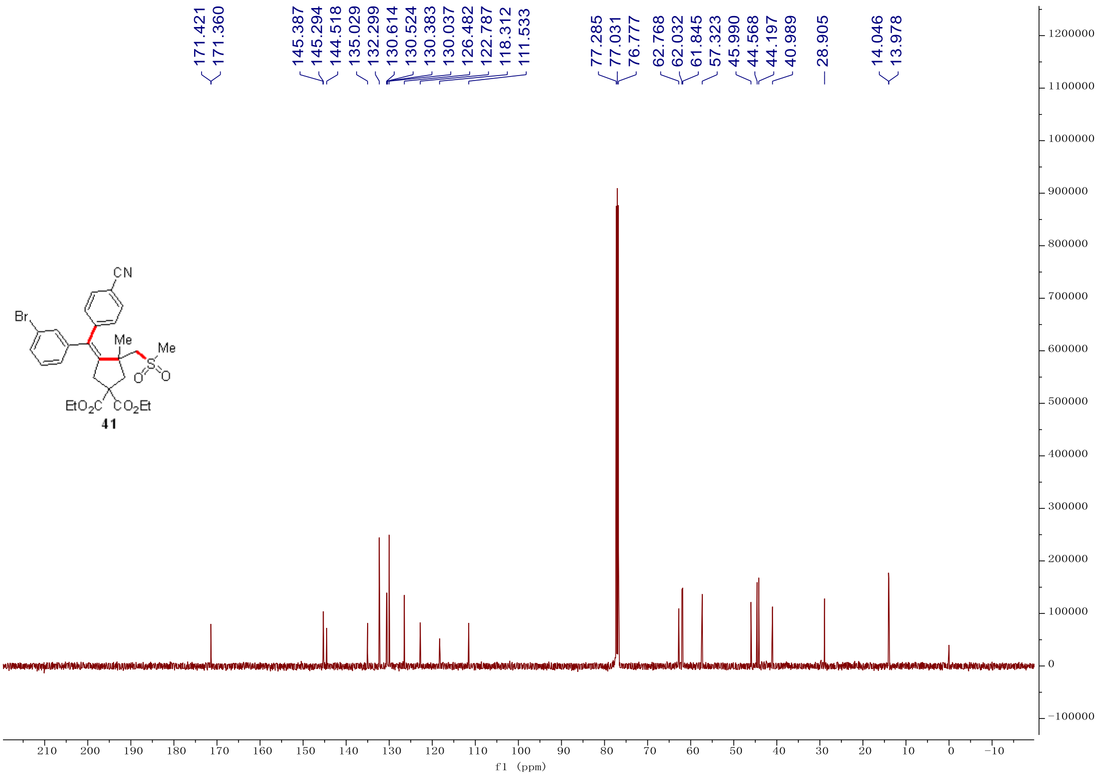

42: ^{1}H NMR (800 Hz, $CDCl_3$)

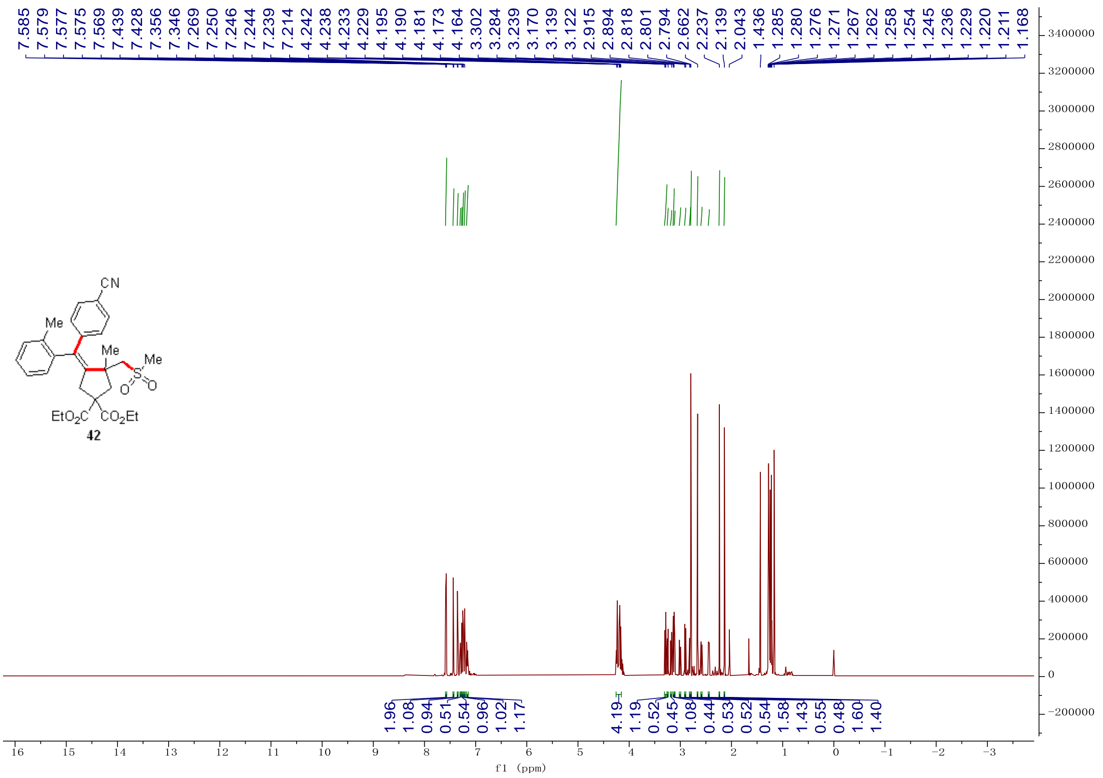

42: ^{13}C NMR (201 Hz, $CDCl_3$)

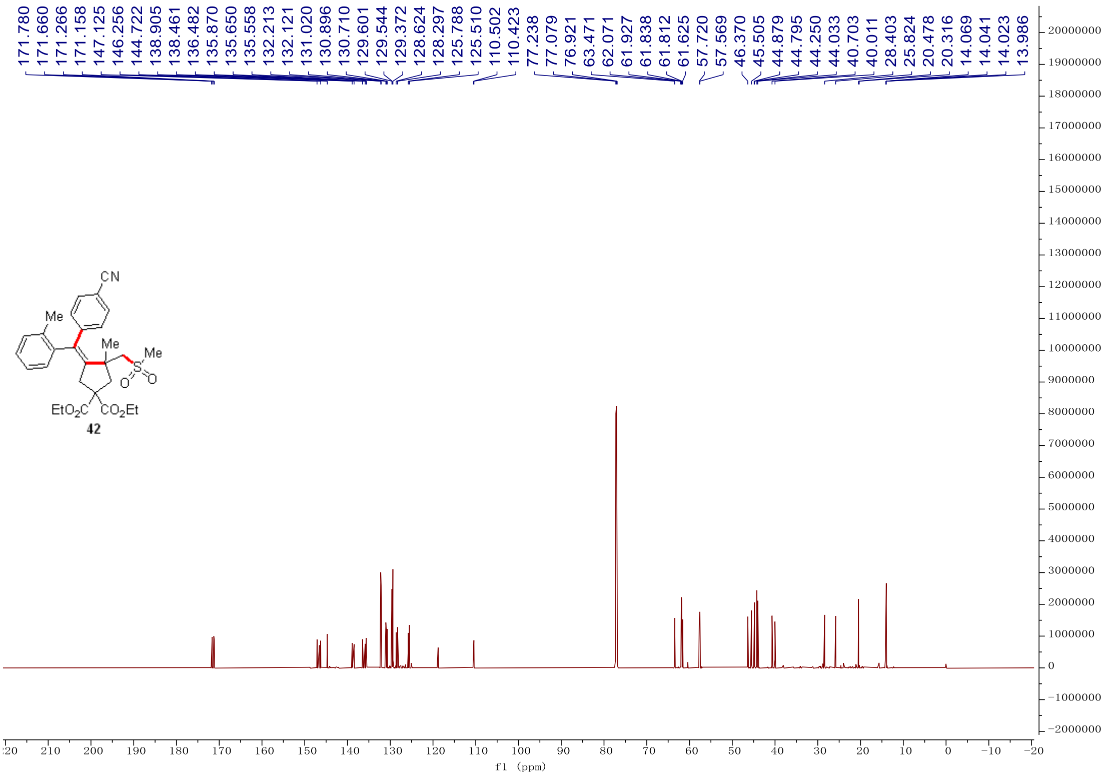

43: ^{1}H NMR (500 Hz, $CDCl_3$)

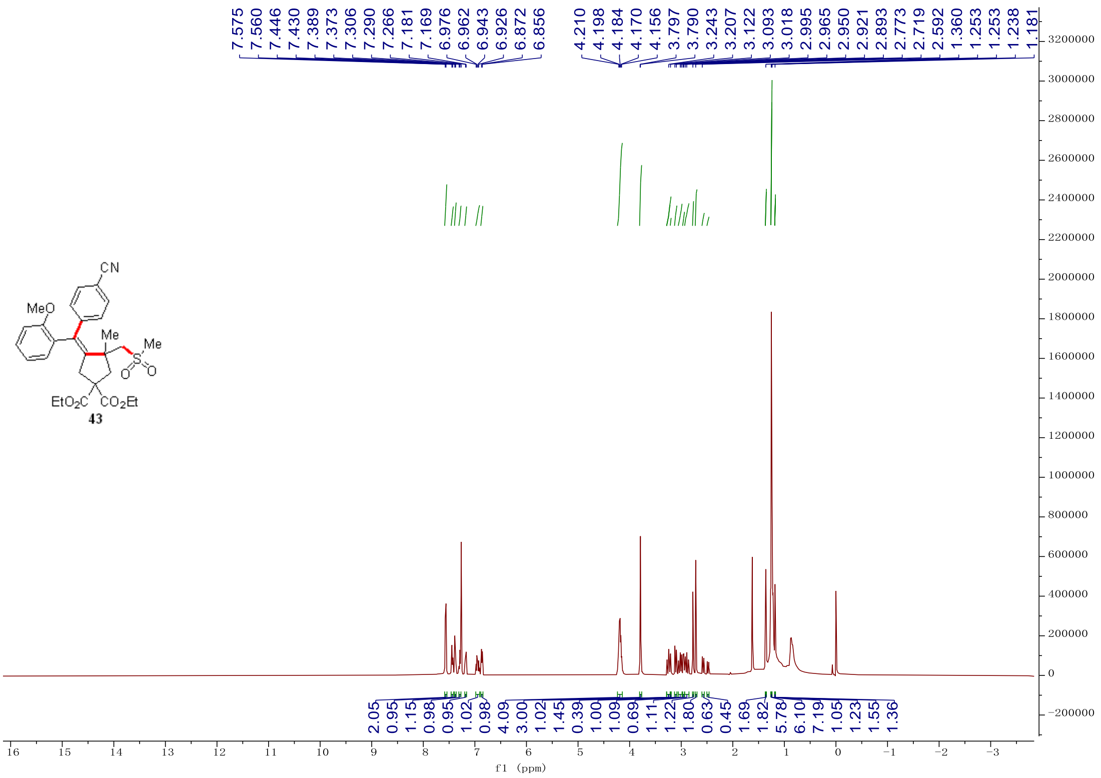

43: ^{13}C NMR (201 Hz, $CDCl_3$)

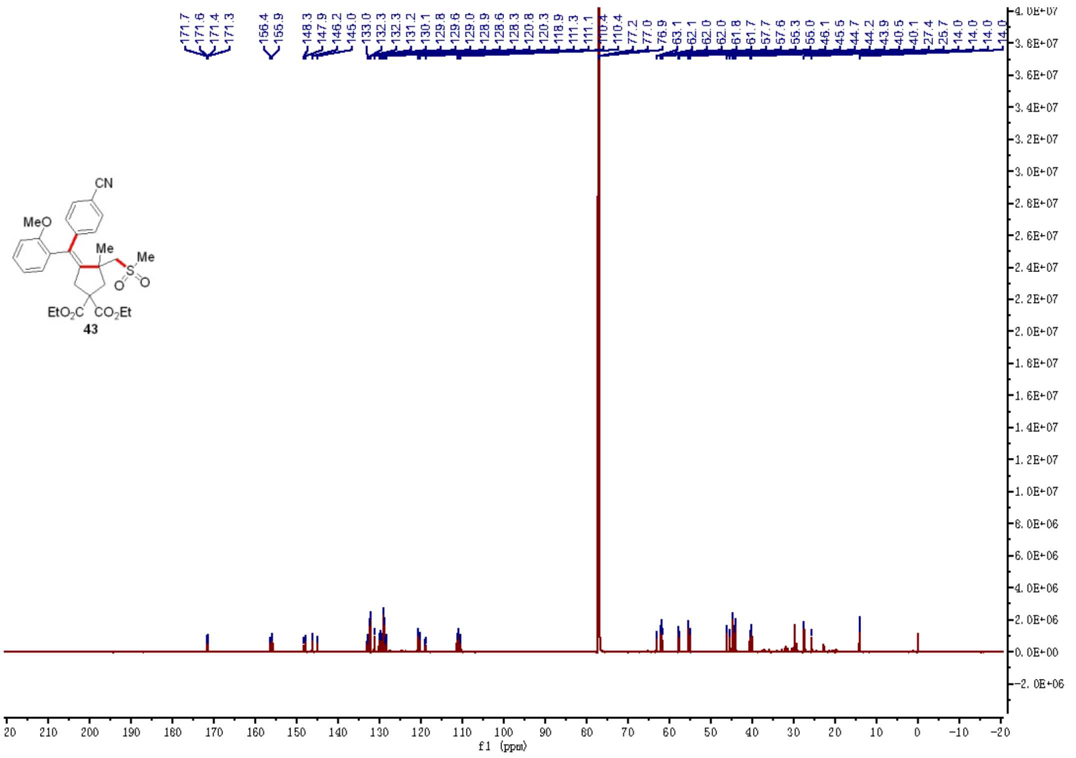

44: ^{1}H NMR (500 Hz, $CDCl_3$)

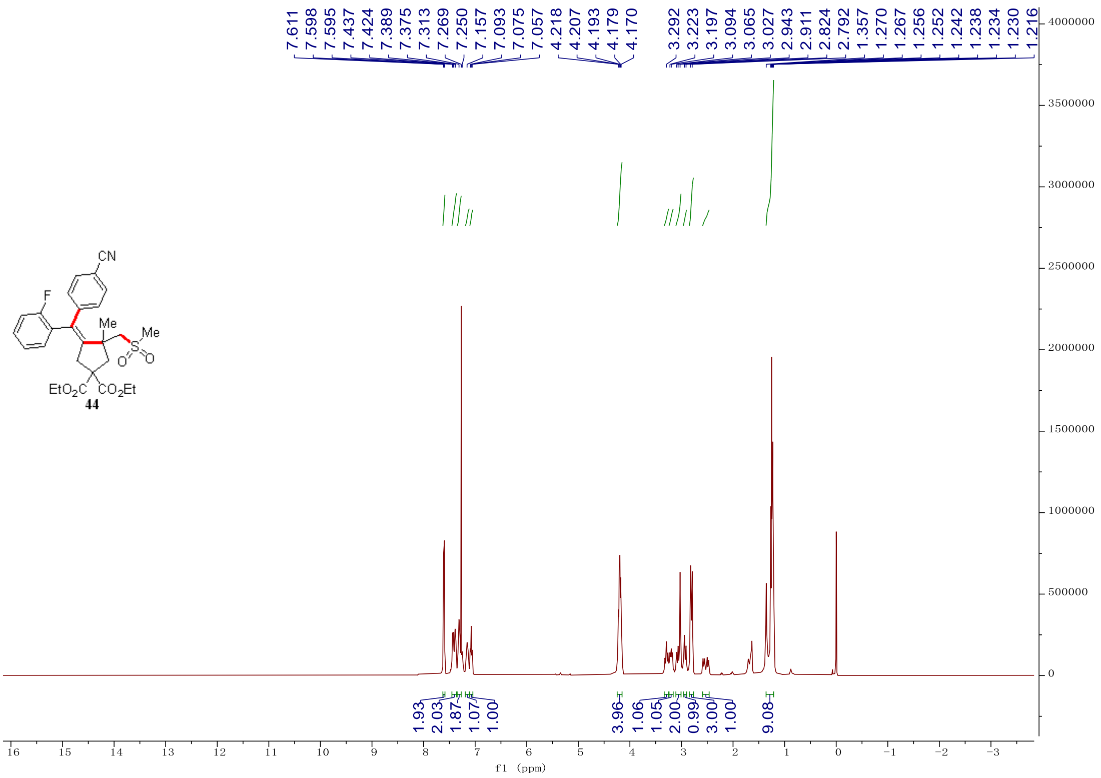

44: ^{13}C NMR (201 Hz, $CDCl_3$)

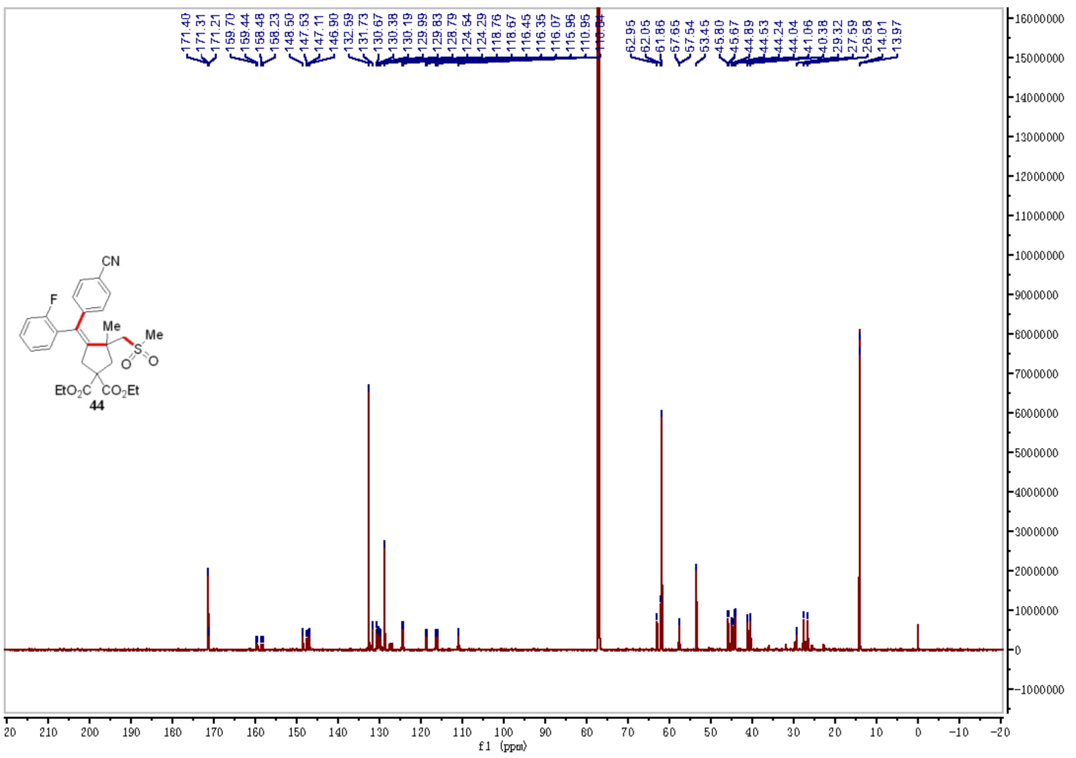

44: ^{19}F NMR (753 Hz, $CDCl_3$)

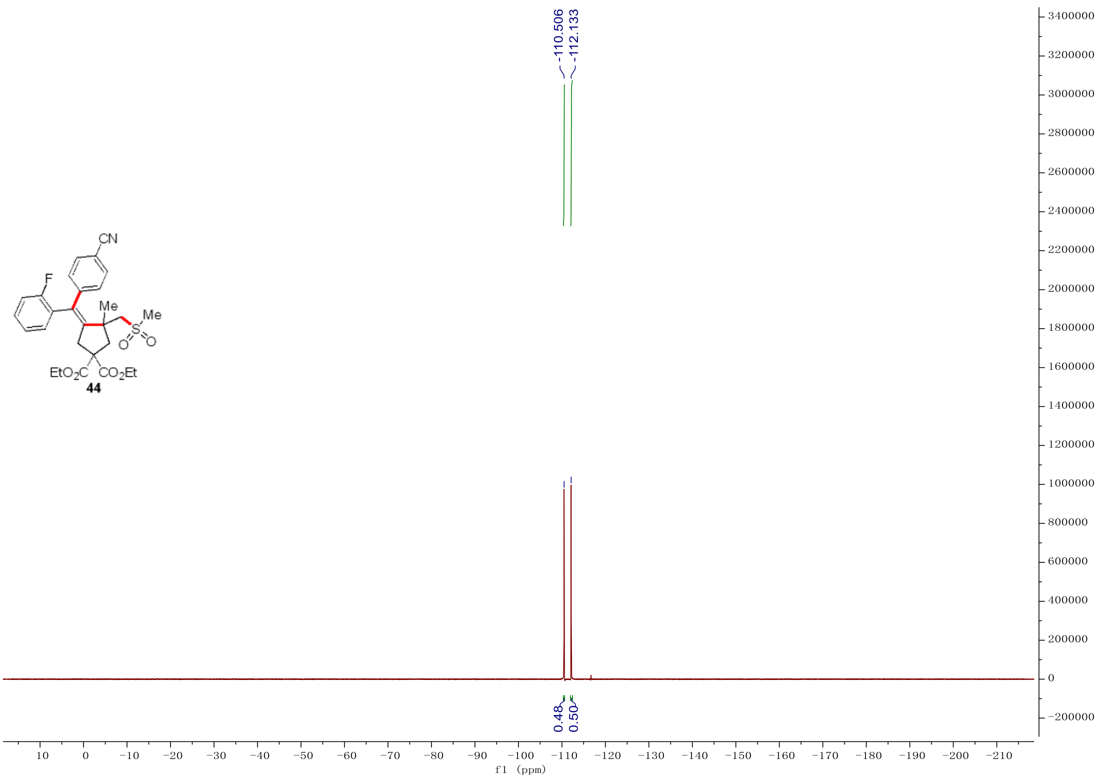

45: ^{1}H NMR (400 Hz, $CDCl_3$)

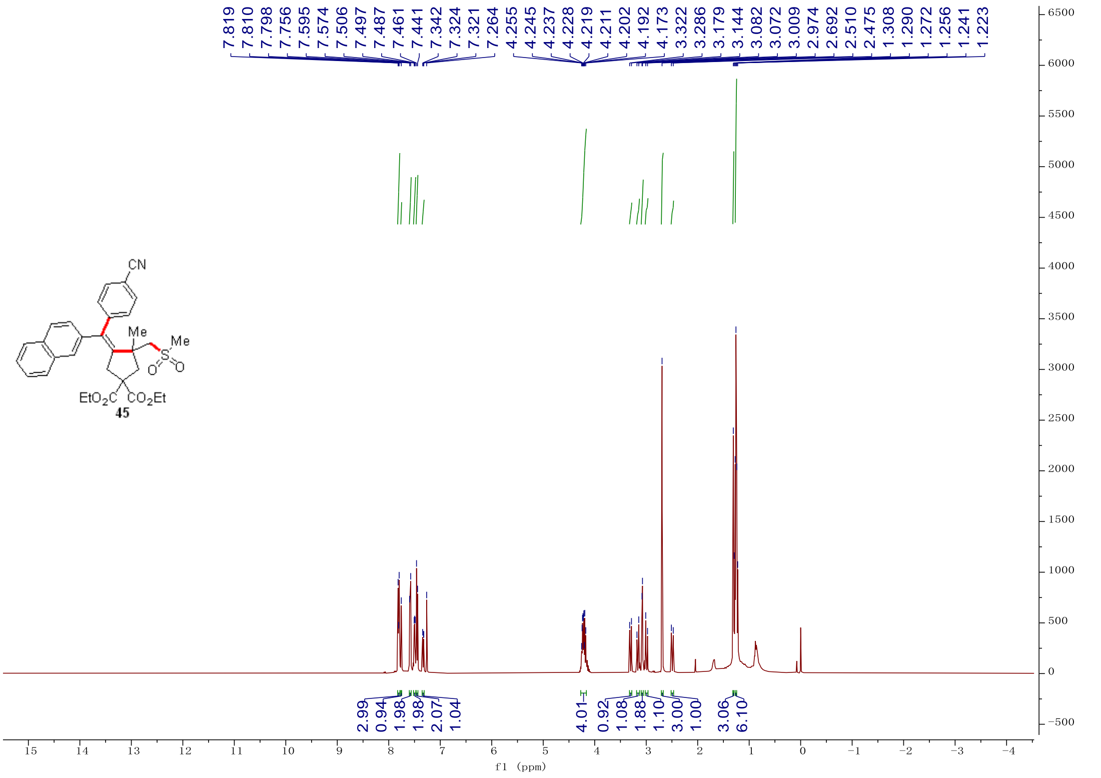

45: ^{13}C NMR (101 Hz, $CDCl_3$)

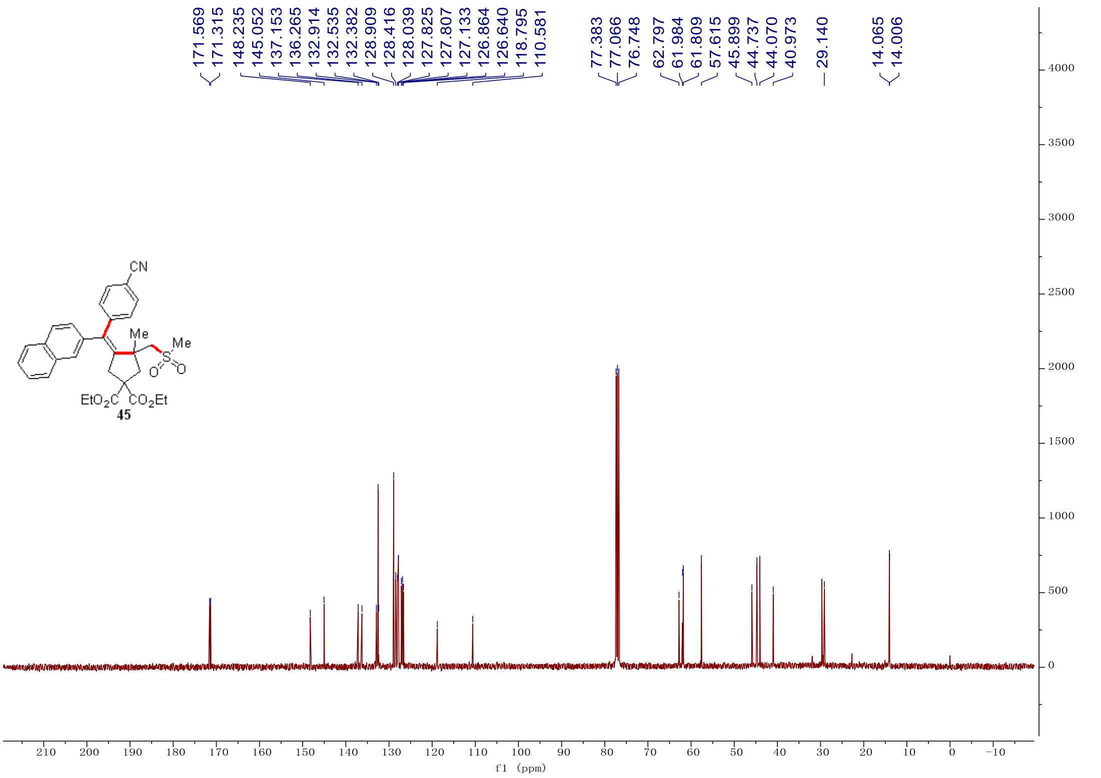

46: ^{1}H NMR (800 Hz, $CDCl_3$)

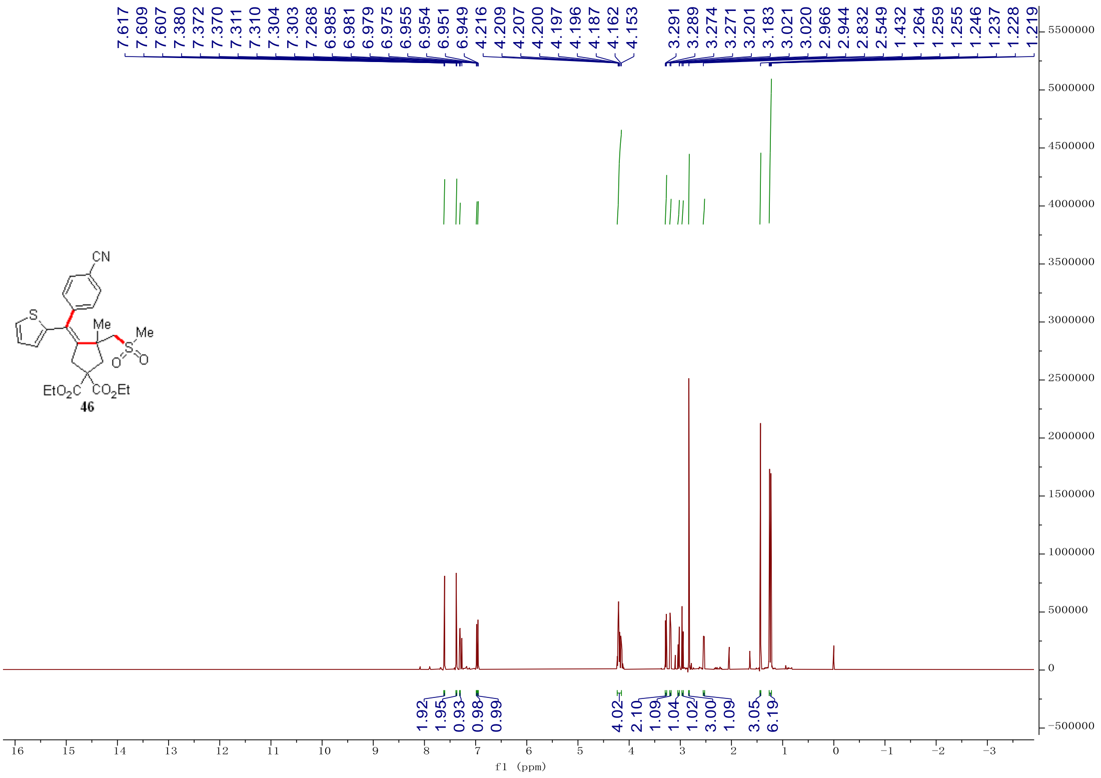

46: ^{13}C NMR (201 Hz, CDCl$_3$)

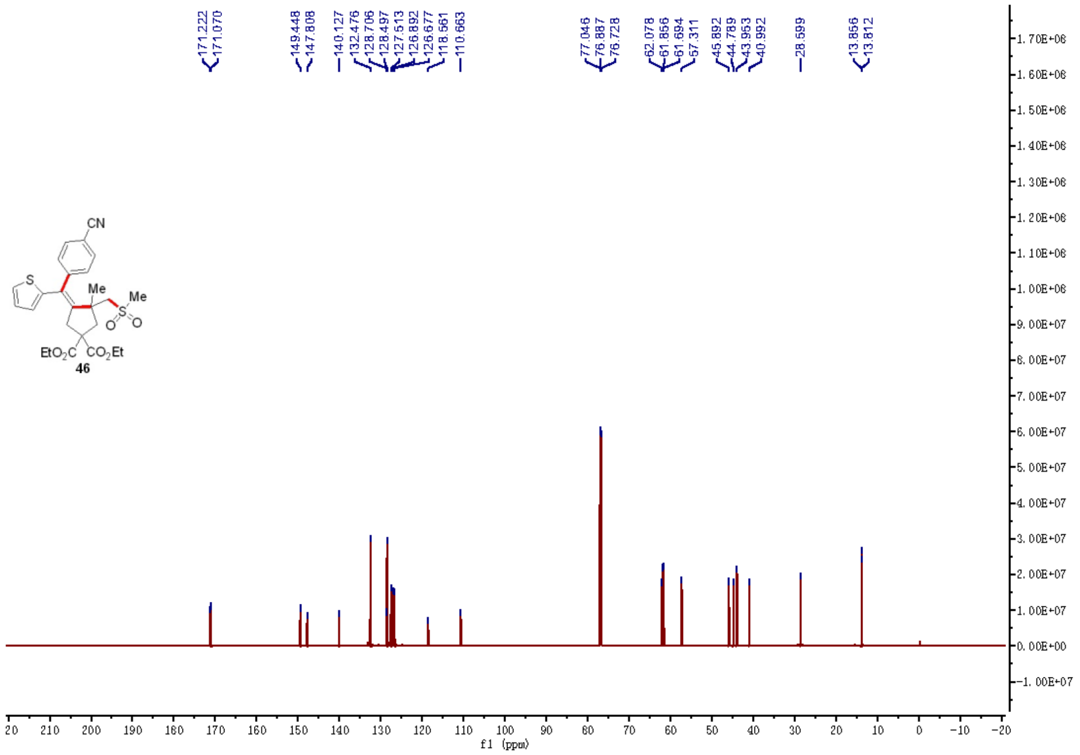

47: ^{1}H NMR (500 Hz, CDCl$_3$)

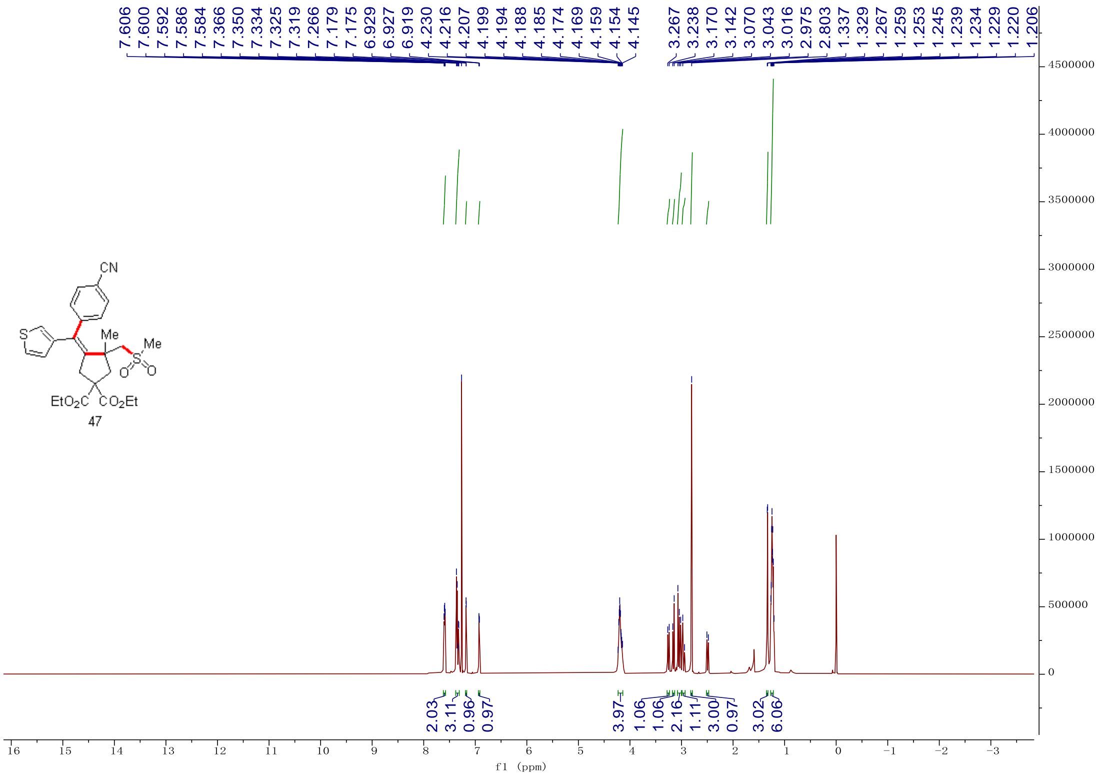

47: ^{13}C NMR (126 Hz, $CDCl_3$)

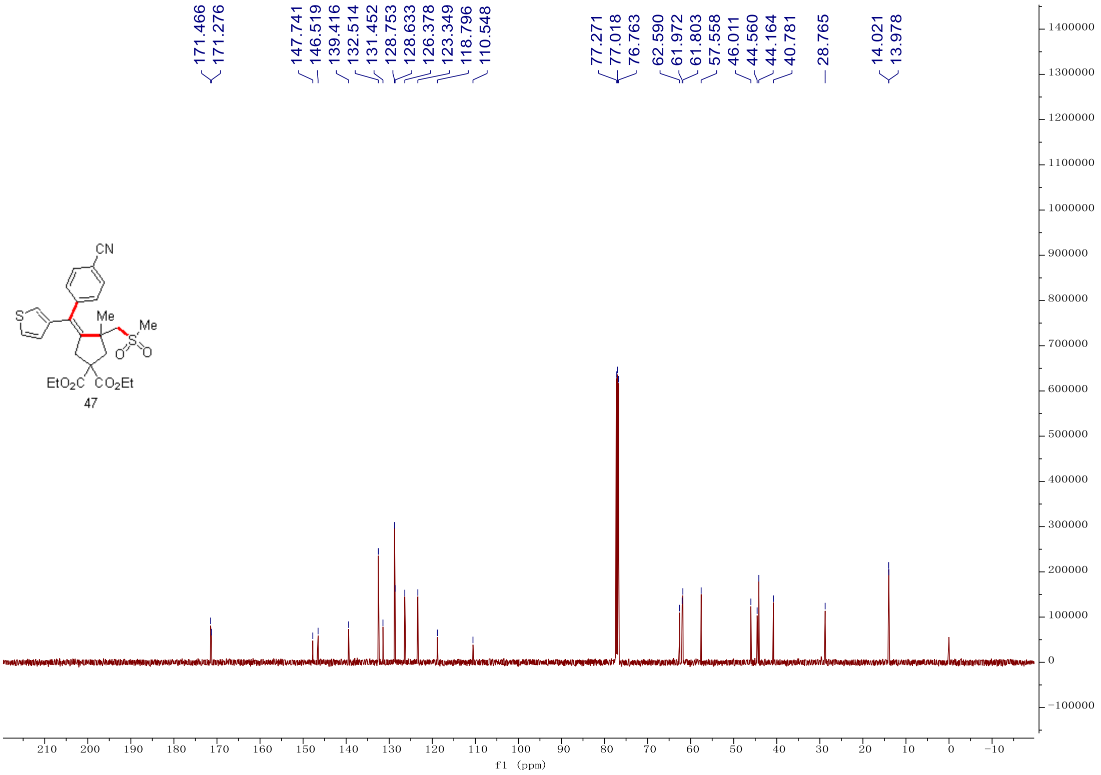

48: ^{1}H NMR (400 Hz, $CDCl_3$)

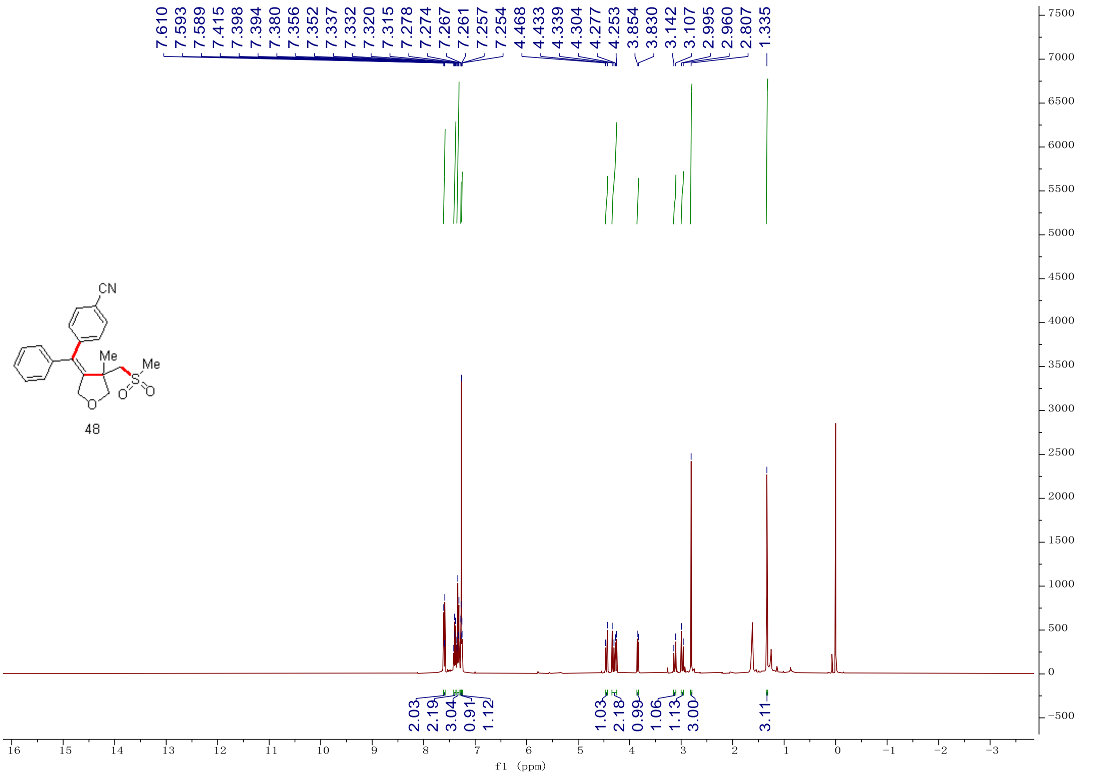

48: ^{13}C NMR (101 Hz, $CDCl_3$)

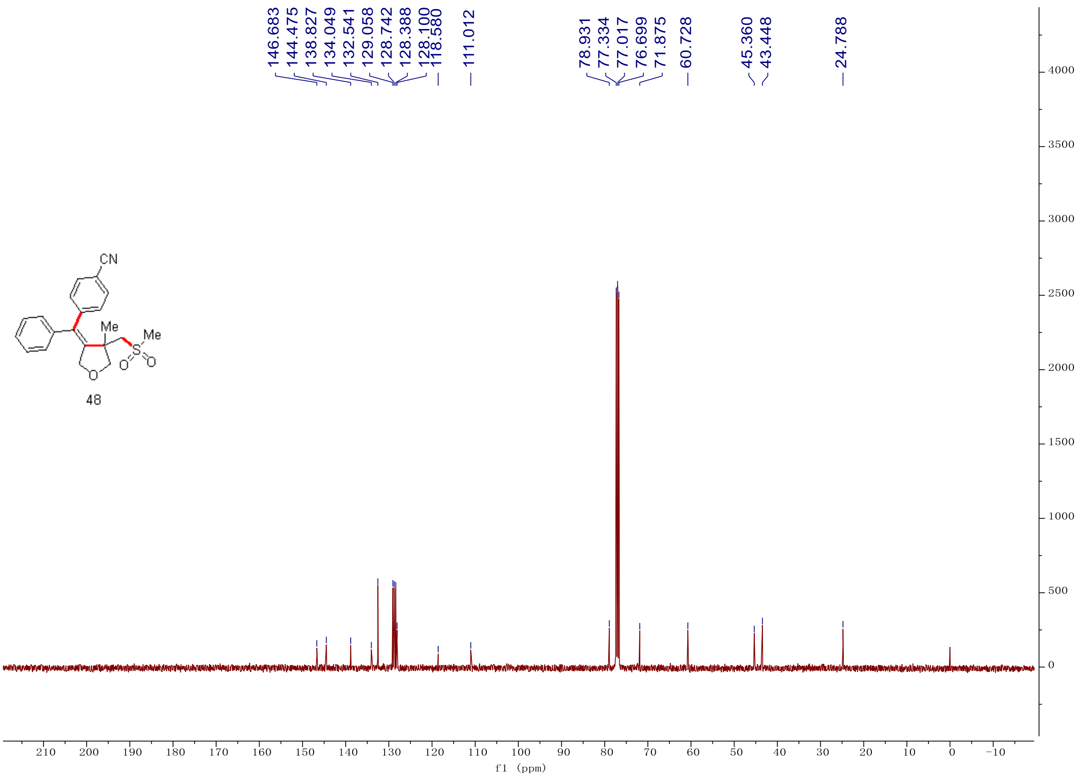

49: ^{1}H NMR (400 Hz, $CDCl_3$)

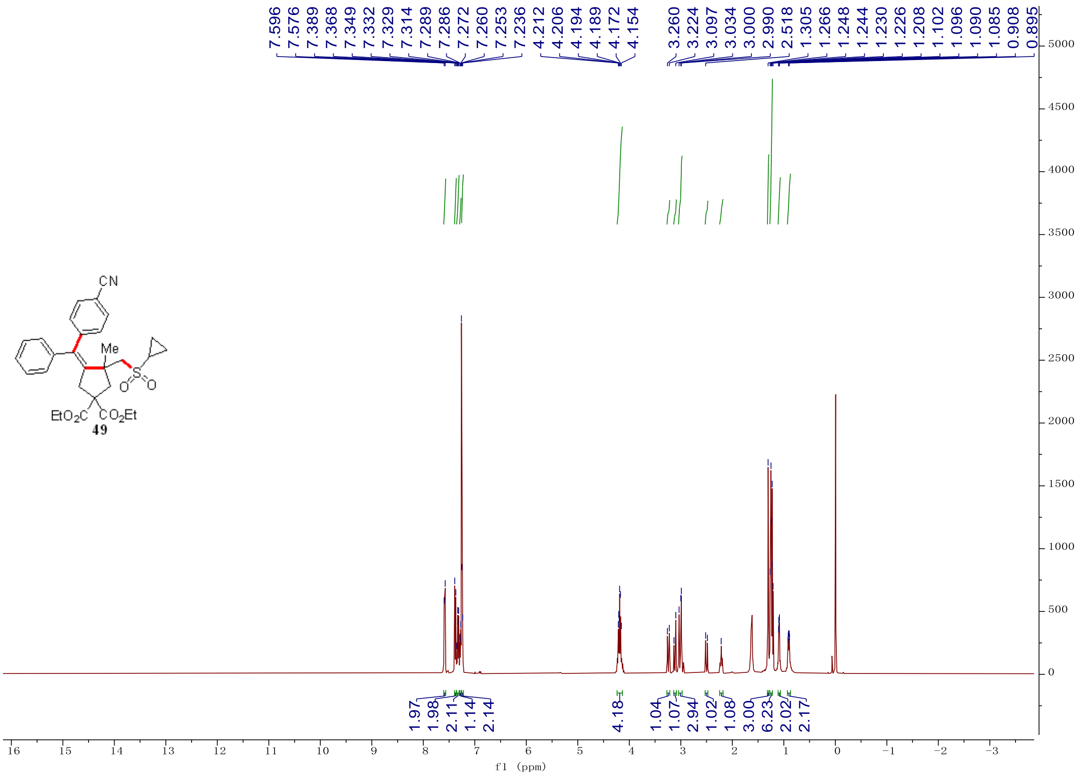

49: ^{13}C NMR (101 Hz, $CDCl_3$)

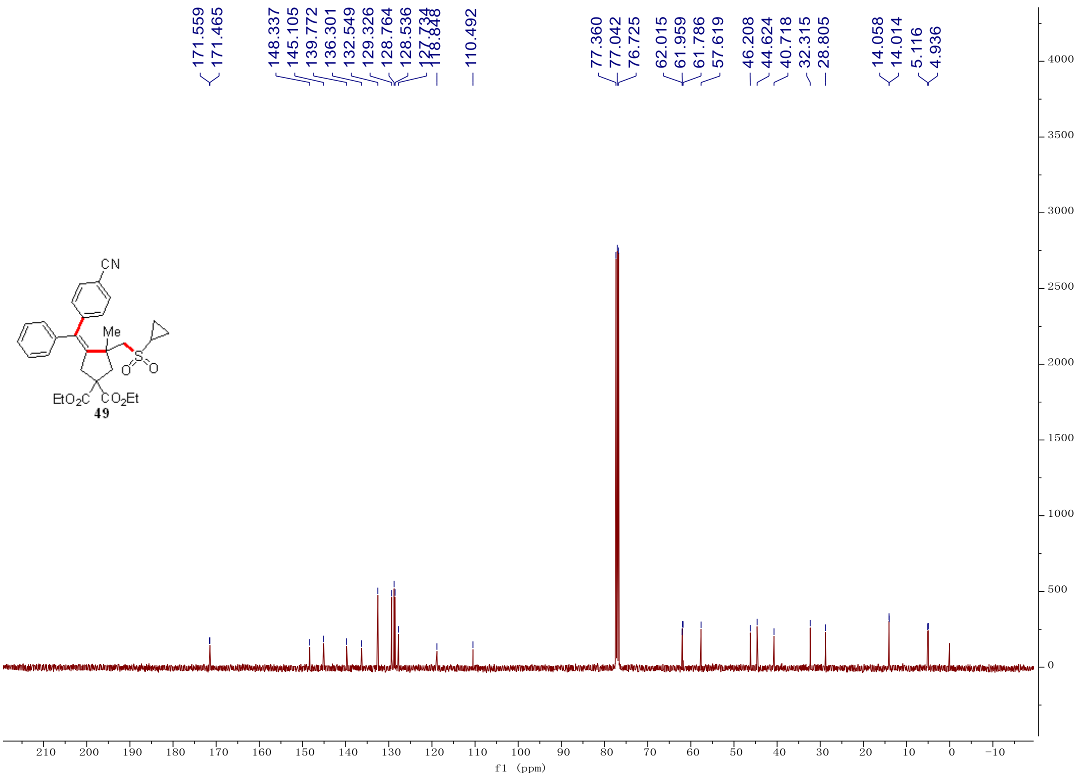

50: ^{1}H NMR (800 Hz, $CDCl_3$)

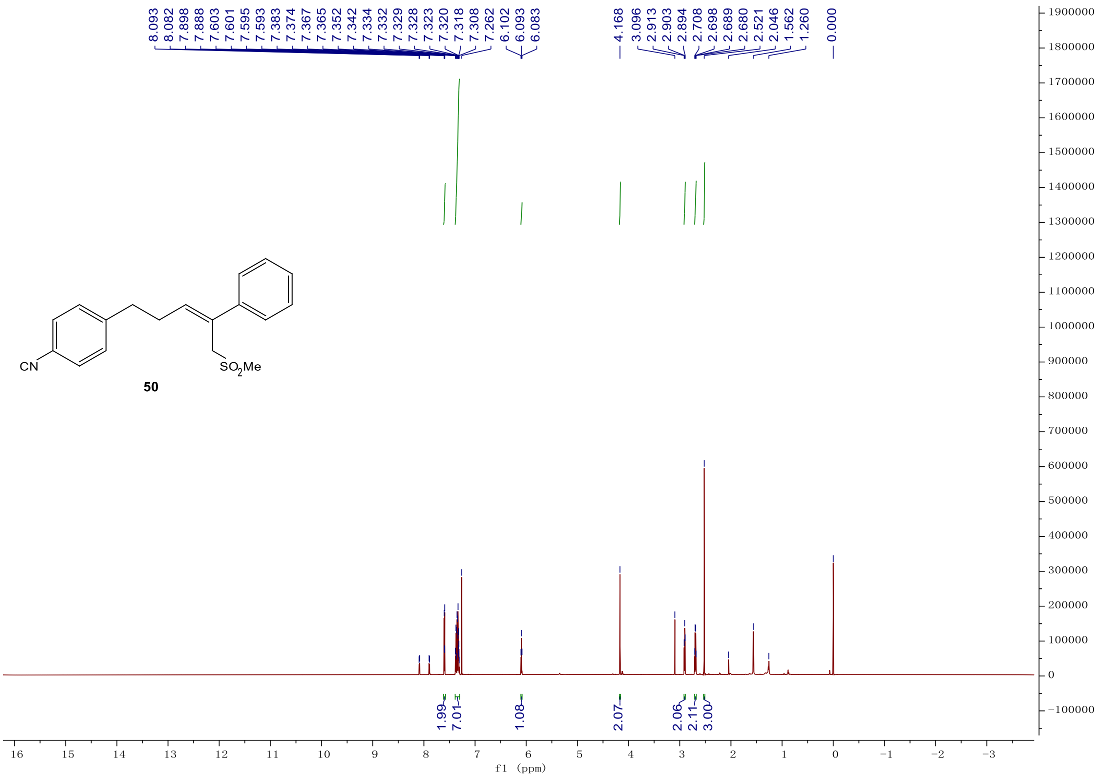

50: ^{13}C NMR (201 Hz, CDCl$_3$)

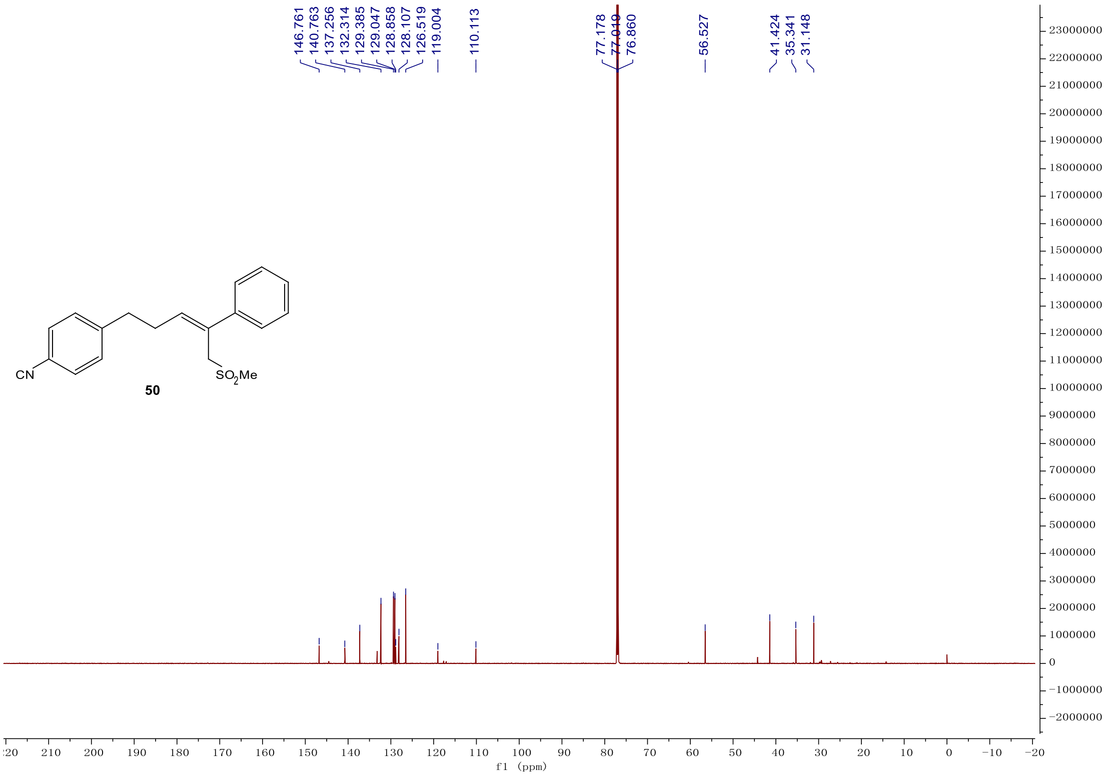

Supplement: Supplementary file 1 — ol4c04222_si_001.pdf [file ol4c04222_si_001.pdf]
